# Genomic characterization of blaKPC transmission via Tn4401a and Tn4401l transposons across Enterobacteriaceae and ESKAPE in Ecuador

**DOI:** 10.1099/mic.0.001772

**Published:** 2026-10-15

**Authors:** Isaías Mejia-Limones, María Benites-Villegas, María Cáceres-Valdiviezo, Derly Andrade-Molina

**Affiliations:** 1 Omics Science Laboratory, Faculty of Health Science, Universidad Espíritu Santo, Samborondón, Ecuador

**Keywords:** *bla*_KPC_, carbapenem resistance, *Enterobacteriaceae*, plasmids, Tn*4401*, whole-genome sequencing

## Abstract

Carbapenem resistance in *Enterobacteriaceae* poses a major threat to public health because of the limited therapeutic options. The dissemination of the *bla*_KPC_ gene, which encodes KPC carbapenemases, typically occurs through Tn*4401* transposons and plasmids that facilitate its transmission. In this study, we analysed 802 genomes of ESKAPE pathogens and other *Enterobacteriaceae* from Ecuador, including both clinical and non-clinical isolates, to determine the prevalence and genetic context of *bla*_KPC-2_ and *bla*_KPC-3_ and their associated mobile genetic elements. Whole-genome analysis revealed several isolates carrying *bla*_KPC_, with *bla*_KPC-3_ restricted to *Klebsiella pneumoniae*. While most isolates harboured the Tn*4401a* transposon, we identified a novel variant, Tn*4401l*, in two *K. pneumoniae* isolates collected between 2020 and 2021. Additionally, two putative plasmids carrying Tn*4401a* were reconstructed: pECU0072020, a mosaic multidrug-resistant plasmid with a unique genetic organization, and pECU0062021, a more compact structure carrying only *bla*_KPC-2_. The findings demonstrate the wide distribution of *bla*_KPC_ within diverse bacterial hosts across multiple regions of Ecuador, the emergence of a novel Tn*4401l* variant and the presence of putative plasmids that could potentially accelerate multidrug resistance dissemination. This study highlights the need for continuous genomic surveillance and coordinated strategies and practices regarding the use of antibiotics to mitigate the risk of outbreaks and interspecies transmission of carbapenem resistance.

**Impact Statement**

To our knowledge, this is the first genomic study characterizing the spread of the *bla*_KPC_ gene, which is responsible for carbapenem resistance, in clinically important bacteria in Ecuador. This study revealed the presence of a novel transposon variant (Tn*4401l*) and previously undescribed plasmids. These mobile elements act as ‘gene vehicles’ facilitating the transfer of resistance genes among bacteria, such as *Klebsiella pneumoniae* and other members of the ESKAPE group to exchange resistance genes, making them more difficult to treat. The identification of these new genetic elements points to new mechanisms for the transmission of resistance, accentuating the pressing necessity for the establishment of national genomic surveillance to anticipate and contain hospital outbreaks. This finding not only provides scientific knowledge about the routes of resistance dissemination but is also of great relevance to clinical microbiology, molecular epidemiology and public health. Ultimately, this study signifies a pivotal advancement in elucidating the dynamics of resistance gene circulation among bacterial species and geographic regions and represents a fundamental step toward the development of more effective control strategies.

## Data Summary

Sequence files for the isolates described in this study can be found in the SRA archive, with project accession numbers PRJEB56918, PRJNA1024947, PRJNA1293794, PRJNA486009, PRJNA514245, PRJNA849201, PRJNA861272, PRJNA946810, PRJNA1338115 and PRJNA956653. A full list of sequences with associated accession numbers can be found in Table S1 (available in Supplementary Material).

## Introduction

Antimicrobial resistance (AMR) is one of the most urgent global health challenges, undermining the effectiveness of contemporary medical interventions and contributing to elevated mortality rates in nosocomial infections. The World Health Organization has ranked carbapenem-resistant *Enterobacteriaceae* and ESKAPE pathogens (*Enterococcus faecium*, *Staphylococcus aureus*, *Klebsiella pneumoniae*, *Acinetobacter baumannii*, *Pseudomonas aeruginosa* and *Enterobacter* spp.) as top-priority threats owing to their limited therapeutic alternatives and recurrent association with nosocomial outbreaks [1].

Among the most concerning resistant determinants are beta-lactamases, particularly extended-spectrum beta-lactamases (ESBLs) and carbapenemase (KPC) genes. ESBLs infections often result in carbapenem use, which promotes the spread of carbapenemase-producing Enterobacterales that typically resist most treatment options currently available [2, 3]. KPC enzymes are Ambler class A *β*-lactamase encoded by the *bla*_KPC_ gene, which is predominantly found in *K. pneumoniae* but also present in other *Enterobacteriaceae*, including members of the ESKAPE group, as well as *Serratia* and *Salmonella* [1, 3, 4]. To date, over 150 *bla*_KPC_ variants have been identified worldwide, with *bla*_KPC-2_ and *bla*_KPC-3_ being the most prevalent [1, 3]. These enzymes confer resistance to a wide spectrum of *β*-lactam antibiotics and have spread globally, facilitated by international travel and dissemination in endemic regions such as the USA, Brazil and Colombia [1, 4, 5]. Antibiotic resistance associated with KPC, especially involving the *bla*_KPC-2_ and *bla*_KPC-3_ variants, is increasing at an exponential rate globally, severely limiting the range of treatment options and posing a growing epidemiological threat [2, 3].

Originally, the dissemination of the *bla*_KPC_ gene was associated with Tn*4401*, a transposon-encoded element of the Tn*3* family which exists as several structural isoforms, which have high transposition without target site specificity, with the gene order of Tn*4401-tnpA*, Tn*4401-tnpR*, IS*Kpn7*, *bla*_KPC_ and IS*Kpn6* [6]. Subsequent studies revealed that *bla*_KPC_ can also be mobilized by mobile genetic elements located on plasmids lacking Tn*4401*, known as non-Tn*4401* elements (NTEs) [1, 3]. These elements encode alternative transposases with higher transposition frequencies, facilitating *bla*_KPC_ mobilization and the spread of resistance. In both contexts, the association of *bla*_KPC_ with mobile elements enables horizontal transmission via plasmid conjugation, thereby enhancing its spread across different bacterial species [1]. Within the Tn*4401* family, the variants most commonly observed are Tn*4401a* and Tn*4401b*. These isoforms differ in that Tn*4401a* contains a 100 bp deletion upstream of *bla*_KPC_ [7]. In addition, the variability of the intervening sequence within its structure, between the IS*Kpn7* and *bla*_KPC_ genes, leads to the presence of different promoters: Tn*4401a* carries P1 and P2, whereas Tn*4401b* carries P1, P3 and P2 [8].

In Ecuador, the first detection of *bla*_KPC-2_ was reported in 2012 using PCR, followed by multiple reports of this variant in clinical isolates [9]. More recently, *bla*_KPC-3_ was identified using whole-genome sequencing from a patient sample in 2021 [10]. Although the number of cases documented in the country is still relatively low, likely due to the limitations in routine molecular surveillance and restricted access to whole-genome sequencing rather than true prevalence, these cases have already posed a significant risk in healthcare settings due to their high virulence, AMR and ability to evade the immune system. To date, the genomic contexts underlying *bla*_KPC_ dissemination in Ecuador have not been systematically studied. Therefore, this work aimed to characterize *bla*_KPC_-carrying isolates from clinical samples and compare them with non-clinical strains lacking this resistance determinant, providing a broader perspective on the local dynamics of carbapenem resistance.

## Methods

### Data collection

Initially, 1,097 publicly available genomes representing 10 bacterial species (*Escherichia coli*, *K. pneumoniae*, *A. baumannii*, *Klebsiella oxytoca*, *Klebsiella variicola*, *Klebsiella michiganensis*, *Klebsiella aerogenes*, *P. aeruginosa*, *Salmonella enterica* and *Serratia marcescens*) were retrieved from the NCBI database [11]. The BioProject accession numbers for all datasets included in this study are provided in the Data Summary. Among the *E*. c*oli* genomes, the majority originated from the One Health Enteric Project, which investigated AMR transmission associated with food-animal production in Ecuadorian communities [12]. From this project, 879 human-derived *E. coli* isolates were included in the initial dataset.

Metadata were manually curated by removing records with incomplete or ambiguous information, non-human isolates and genomes lacking corresponding SRA sequencing data. After this filtering step, 1,021 sequencing datasets were downloaded and *de novo* assembled. Genome quality was assessed using three inclusion criteria: (i) completeness ≥90% and contamination ≤5%; (ii) assembly quality with an N50 ≥40,000 bp; and (iii) species confirmation by FastANI v1.34 [13] Average Nucleotide Identity (ANI)(≥90%) using species-specific reference genomes (Table S1). One genome initially classified as *E. coli* was reassigned as *Hafnia paralvei* based on ANI analysis and was therefore excluded from subsequent analyses. Genomes failing to meet any of the inclusion criteria were excluded, resulting in a final dataset of 802 genomes, including 705 of the 879 *E. coli* genomes from the One Health Enteric Project.

For clarity in the figures, isolate identifiers were abbreviated by combining the first letter of the BioSample or genome name with the last three digits of the corresponding accession number (e.g. S945 for SAMN09832945).

### Raw read processing and genome assembly

Raw reads were downloaded using SRA Toolkit v3.1.0 (https://github.com/ncbi/sra-tools) [14], and then, the reads were analysed using FastQC v0.12.1 [15] and MultiQC v1.26 [16]. Short reads were assembled with Unicycler v0.5.1 [17], while 55 long-read *P. aeruginosa* genomes were assembled with Flye v2.9.5 [18]. QUAST v5.2.0 [19] was used to check the quality of the assembly.

### Resistance gene detection and genomic context

ABRicate v1.0.1 [20] was used with the NCBI database [21] to screen all genomes for resistance-conferring genes, focusing on *bla*_KPC_. Genomes positive for *bla*_KPC_ were subsequently annotated using Bakta v1.10.3 [22], and a comprehensive analysis of the genes located around *bla*_KPC_ was performed within 10 kb upstream and downstream of *bla*_KPC_, observing insertion sequences (ISs) within the genomic context. The TnCentral database [23] was integrated into ABRicate to facilitate the detection of transposons.

### Phylogenomic analysis

Two phylogenomic studies were performed utilizing Mashtree v1.4.6 [24], a tool for phylogenetic reconstruction that uses Mash distances. The first analysis included all 802 genomes, including metadata such as *bla*_KPC_ gene status, bacterial order, bacterial species and transposon type. The second analysis focused on the 60 *bla*_KPC_-positive genomes containing Tn*4401* elements.

### Transposon analysis, location and plasmid detection

A custom database was created using Tn*4401* members (Tn*4401a* ‘KT378596.1’, Tn*4401b* ‘KT378596.1’, Tn*4401d* ‘JN974188.1’, Tn*4401i* ‘MH047294.1’ and Tn*4401l* ‘PP567283.1’) for the correct determination of the transposon and added in ABRicate. Once the transposon was detected, the location of the transposon was determined using ABRicate with PlasmidFinder 2.0.1 (database version 2023.01.18) [25], which determined the replicons of each genome. The plasmid sequences corresponding to each replicon (ColRNAI ‘NZ_AP024759.1’, pBK30683 ‘KF954760.1’, pKpQIL-IT ‘JN233705.2’, pKPN-IT ‘JN233704.1’, pKPHS1 ‘CP003223.1’, pKP91 ‘CP000966.1’ and pKPN3 ‘CP000648.1’) were downloaded from NCBI and analysed for the Tn*4401* transposon presence. Sequences of the genomes with plasmids containing the transposon were extracted using BBmap v39.06 [26], subsequently assembled using SPAdes v3.15.5 [27], and the Tn*4401* transposon presence was established using the custom database in ABRicate.

Genomes lacking replicons but predicted to carry plasmids were analysed with PLASMe v1.1 [28]. Plasmid reconstruction of sequences harbouring Tn*4401* was executed to allocate plasmids to each isolate via reference mapping. A plasmid was deemed associated with an isolate if it exhibited greater than 90% coverage. In instances where two plasmids both attained greater than 90% coverage and exhibited a discrepancy of less than 10%, both plasmids were allocated. Conversely, if the disparity exceeded 10%, only the plasmid with the highest coverage was designated. To ascertain the target site duplication (TSD), we employed TETyper v1.1 [29], which was configured to identify 5 bp flanking regions (Table S2). For the replicon pBK30683, which is associated with Tn*4401l*, plasmid assignment to the isolates was considered by default, regardless of the presence of other plasmids, since this replicon was uniquely present in both isolates and the reconstruction of the track revealed its full extent, alongside *bla*_KPC_ and Tn*4401*. For the Tn*4401l* genetic context determination in pBK30683 isolates, PyGenomeVIZ v1.6.1 was used [30].

### Putative plasmid

The putative plasmid determined by PLASMe was extracted from the genomes assembled with Unicycler using BedTools v2.31.1 [31]. To determine the similarity of the putative plasmid to other plasmids, BLASTn [32] in NCBI was performed, and the plasmids with the highest similarity were used to further determine the percentage of coverage and identity using BLASTn 2.15.0+ [33]. Subsequently, the putative plasmid was annotated using Bakta v1.10.3 to search the genetic context of the transposon in the plasmid and visualized using BRIG (blast Ring Image Generator Files) v0.95 [34].

## Results

### Distribution of *bla*_KPC_ among ESKAPE group and *Enterobacteriaceae*

In this study, we analysed the distribution of carbapenemase genes *bla*_KPC-2_ and *bla*_KPC-3_, together with the transposons Tn*4401l* and Tn*4401a* using whole-genome phylogenomic analyses of 802 genomes of ESKAPE and high-risk enterobacteria isolates from human subjects. Clinical metadata, including disease status, were not consistently available across the included datasets. The ESKAPE group included genomes from *K. pneumoniae* (*n*=55), *P. aeruginosa* (*n*=19) and *A. baumannii* (*n*=8), while the isolates from high-risk enterobacteria included *K. aerogenes* (*n*=4), *S. enterica* subsp. *enterica* serovar Infantis (*n*=4), *S. enterica* subsp. enterica serovar Typhi (*n*=1), *K. michiganensis* (*n*=1), *K. variicola* (*n*=2), *S. marcescens* (*n*=2), *K. oxytoca* (*n*=1) and *E. coli* (*n*=705). Among isolates with clinical metadata, *bla*_KPC-2_ and *bla*_KPC-3_ were detected exclusively in isolates from diseased individuals. Moreover, bla_KPC-3_ was found only in *K. pneumoniae* isolates, consistent with its recognized association with carbapenem-resistant *K. pneumoniae* in Ecuador. Most *bla*_KPC_-carrying isolates were also associated with transposons belonging to the Tn*3* family, specifically Tn*4401a* and Tn*4401l* (Fig. 1).

**Fig. 1.**
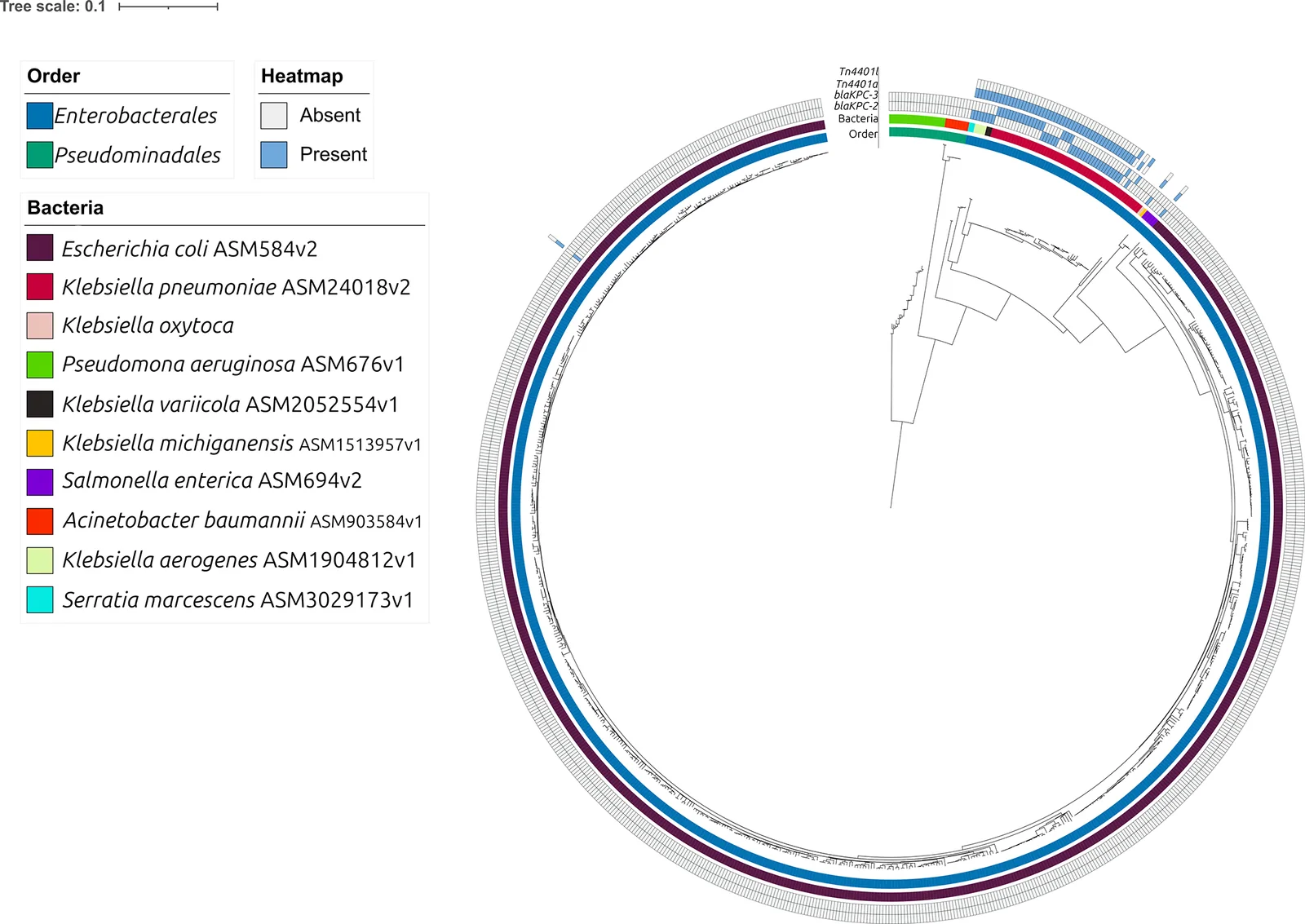
Overview of the genome selection workflow and phylogenetic relationships of the analysed isolates. Circular phylogenetic tree inferred from Mash distances. The inner ring indicates the bacterial order, the outer ring indicates the bacterial species, and the heatmap shows the presence (blue) or absence (grey) of the target genetic element (*bla*_KPC-2_, *bla*_KPC-3_, Tn*4401a* and Tn*4401l*).

### Genomic contexts of Tn*4401*: interplay between ISs and replicon types

Among the *bla*_KPC_-positive isolates, *K. pneumoniae* was the predominant species, accounting for 45% of all isolates carrying *bla*_KPC-2_, followed by *K. aerogenes* (6.66%). Two isolates each of *K. variicola* and *S. marcescens*, and one isolate each of *E. coli*, *K. oxytoca* and *S. enterica*, also harboured *bla*_KPC-2_. In contrast, *bla*_KPC-3_ was detected exclusively in *K. pneumoniae*. MLST revealed that *bla*_KPC-2_-positive *K. pneumoniae* isolates were primarily associated with the high-risk clones ST11, ST258, ST307, ST152 and ST472, whereas *bla*_KPC-3_ was identified in ST45, the single-locus variant ST45-1LV and ST22. Among *K. aerogenes* isolates, ST472 was the predominant sequence type. All *bla*_KPC_-positive isolates carried the Tn*4401* transposon. Tn*4401a* was the predominant variant, accounting for 96.66% of the isolates, and was detected not only in *K. pneumoniae* but also in *E. coli*, *S. enterica*, *K. aerogenes*, *K. oxytoca*, *S. marcescens* and *K. variicola*. In contrast, Tn*4401l* was identified in only two *K. pneumoniae* isolates (3.33%), both carrying *bla*_KPC-3_ on a high-coverage plasmid (Table S2). Notably, these isolates were recovered from nosocomial infections in Guayaquil and Riobamba between 2020 and 2021, representing the first detection of the novel Tn*4401l* variant in this collection. Geographically, most *bla*_KPC_-positive isolates were recovered from the country’s largest metropolitan areas, including Quito, Guayaquil, Cuenca and Riobamba (Fig. 2).

**Fig. 2.**
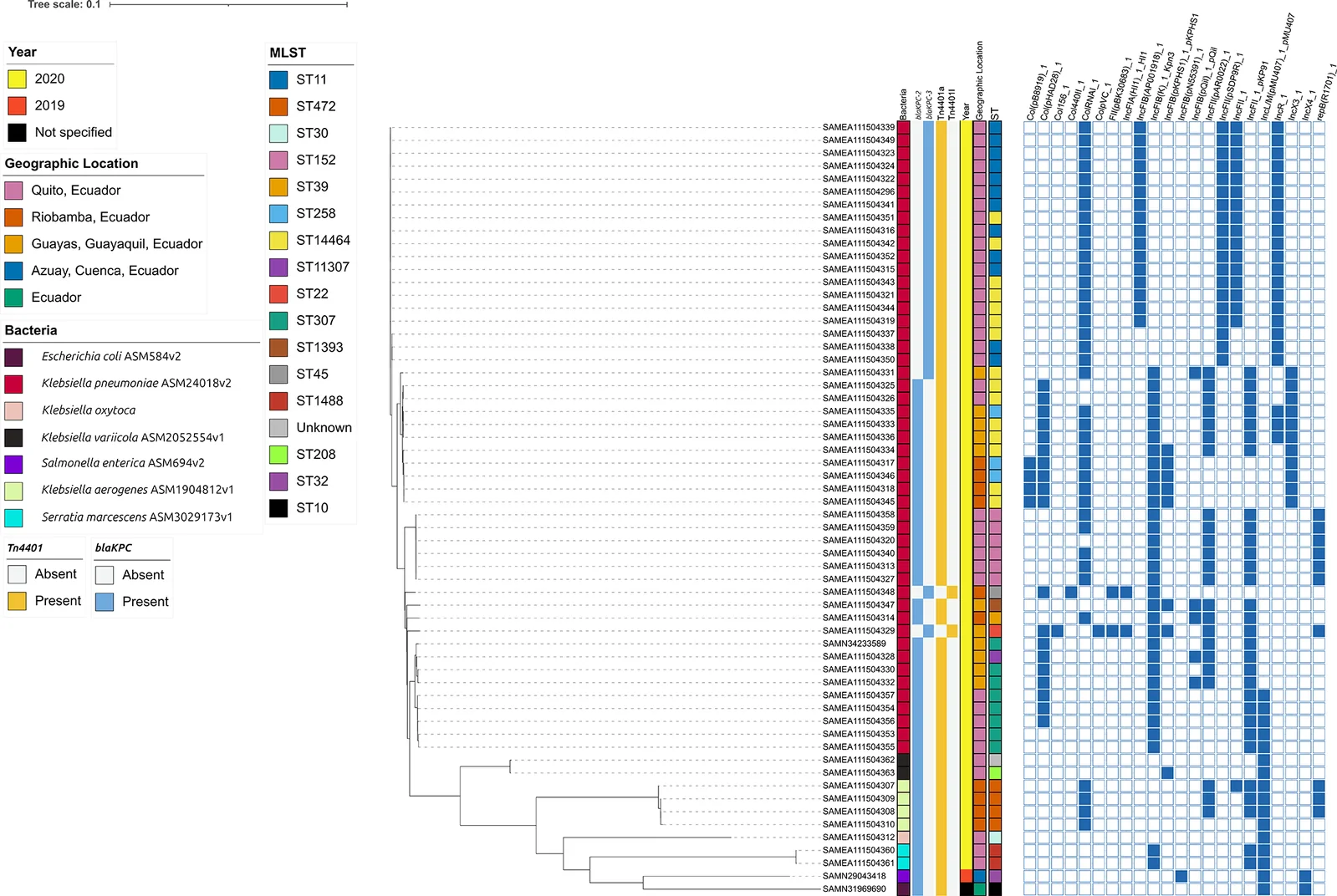
Mash-based phylogenetic tree of *bla*_KPC_-positive isolates recovered in Ecuador. The leftmost column shows the analysed isolates. The subsequent annotation tracks indicate bacterial species, year of isolation, presence of *bla*_KPC_, Tn*4401* variants, geographic origin and MLST. The binary heatmap on the right displays the distribution of plasmid replicon types identified in each isolate.

Analysis of the replicons for the presence of the Tn*4401* transposon showed that only IncFIB(pQil), ColRNAI and FII(pBK30683) contained both the *bla*_KPC_ gene and the transposon. Based on these findings, plasmid reconstruction was performed through reference mapping, which indicated that 32 isolates had high coverage for the ColRNAI plasmid, 5 for IncFIB(pQil), 2 for FII(pBK30683), 2 showed similarity to both IncFIB(pQil) and ColRNAI and 3 were undetermined (Table S2). The remaining 16 isolates revealed 2 putative plasmids, as described below.

To further investigate the genetic context of Tn*4401*, TSDs were characterized and correlated with specific plasmid backbones. A strong association between TSD patterns and plasmid types was observed. The ColRNAI replicon exhibited the greatest TSD diversity, including TGGCT, ATTGA, ATCCA, ATTAT and TGGCG, whereas IncFIB(pQil) was exclusively associated with the ATTGA TSD (Fig. 3). Although both ColRNAI and IncFIB(pQil) shared the ATTGA TSD, FII(pBK30683) was associated with the unique GTTCT TSD, while TGGCT was exclusively detected among ColRNAI plasmids.

**Fig. 3.**
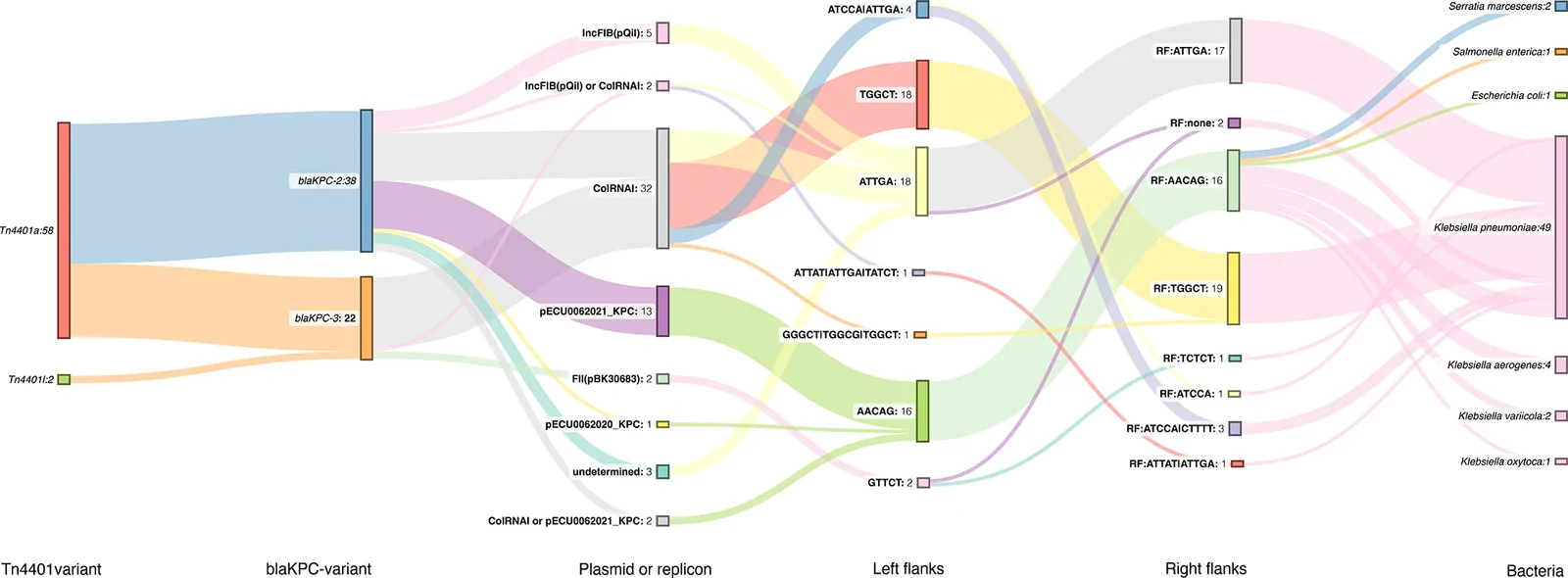
The Sankey flow diagram illustrates the presence of carbapenemase-carrying transposon variants (Tn*4401a* and Tn*4401l*) in enterobacterial isolates and their relationship with *bla*_KPC-2_ and *bla*_KPC-3_, distinct plasmid replicon types, TSD sequence insertion, immediate left flank and right flank sequences upstream and downstream, respectively, of Tn*4401*, and host bacterial species. The diameters of the lines are proportional to the frequency of plasmids. Notably, both putative plasmids, pECU0072020 and pECU0062021, carry Tn*44011a*.

Notably, ColRNAI plasmids were identified exclusively in *K. pneumoniae*. In contrast, plasmids represented by pECU0062021_KPC were detected across multiple bacterial species while consistently maintaining the same AACAG TSD on both the left and right flanking regions, suggesting conservation of the transposition event despite differences in bacterial host.

Significantly, our findings indicate that while most TSDs were identical, exceptions were observed between the left and right flank TSDs, and multiple potential associated TSDs were identified. The identified TSDs could vary not only by plasmid but also by bacterium. Several sequences from the left flank (GTTCT, TGGCT, GGGCT, TGGCG, ATTAT, TATCT and ATCCA) and right flank (ATTGA, TGGCT, TCTCT, ATCCA, CTTTT, ATTGA and ATTAT) were exclusively associated with *K. pneumoniae*. Conversely, the sequence AACAG was associated with multiple species within the *Klebsiella* complex, as well as other bacterial species such as *S. marcescens*, *S. enterica* and *E. coli* (Figs 3 and 4).

**Fig. 4.**
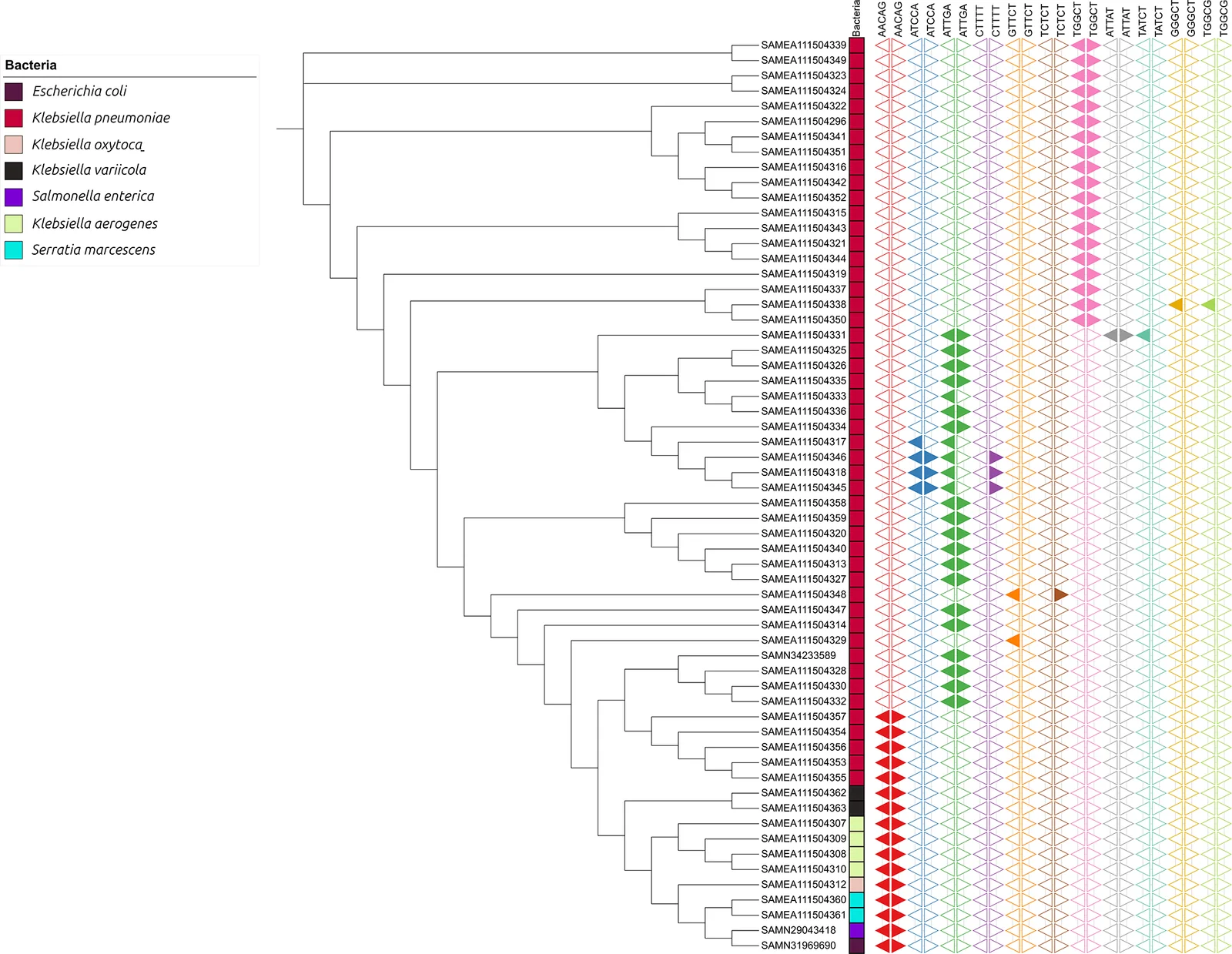
Phylogenomic analysis of *bla*_KPC_ Tn*4401*-carrying genomes, depicting the distribution of flanking sequences across different bacterial hosts. The analysis shows the TSD, with arrows pointing left representing the left flanking sequences and arrows pointing right representing the right flanking sequences upstream and downstream of Tn*4401*, respectively.

The FII (pBK30683) (GenBank ID: KF954760.1) plasmid was associated with Tn*4401l*, prompting plasmid reconstruction to confirm its presence. The reconstructed plasmid exhibited high identity and coverage with FII (pBK30683), although with increased length, likely reflecting short-read assembly artefacts. An examination of the two extracted sequences of pBK30683, identified within SAMEA111504348 and SAMEA111504329, revealed a transposon located at the insertion site delineated by TSD GTTCT, spanning positions 20.706–30.643 and 8.144–18.08, respectively. These plasmids exhibited lengths ranging from 170 to 186 kbp, with a G+C content of 54 mol%, similarly to the original pBK30683 with 54.04 mol% GC content. Resistome analysis identified minor variations in the presence and absence of AMR genes. The core resistance genes detected in both the pBK30683 reference and the analysed isolates included *bla*_KPC-3_, *aadA1*, *blaOXA-9*, *blaTEM-150*, *aph(6)-Id*, *aph(3'')-Ib*, *sul2* and *dfrA14*. Additionally, variants of *aac(6')-Ib* were observed, such as *aac(6')-Ib-W104R* in SAMEA111504329 and *aac(6')-Ib-AKT* in SAMEA111504348 (Table S3).

The genomic context of Tn*4401l* comprises several functional elements associated with bacterial adaptation, including genes involved in replication control, conjugation and plasmid SOS inhibition. The flanking regions were enriched with metal resistance genes such as *NcrBCY* and *ChrB* (Table S4). Although the reference transposon carried *bla*_KPC-2_, all isolates analysed in this study harboured *bla*_KPC-3_ (Fig. S1).

### Emergence of novel plasmids linked to *bla*_KPC_ transmission through Tn*4401* in *K. pneumoniae*

Additionally, genome assembly followed by PLASMe prediction allowed the identification of two putative plasmids carrying Tn*4401a* in the Ecuadorian clinical isolates. The first plasmid, pECU0072020, was ~76.6 kbp in length and was obtained from a *K. aerogenes* biosample (SAMEA111504310). The second plasmid, pECU0062021, was approximately 66.8 kbp in length and was obtained from a *K. oxytoca* biosample (SAMEA111504312). Both plasmids share a conserved core of genes involved in replication, maintenance and horizontal transfer, supporting their potential ability to persist and move between different species. However, they differ in the repertoire of resistant genes and the organization of their mobile elements (Fig. 5). All samples that presented this novel plasmid carried *bla*_KPC-2_ and were found in seven bacteria that include *K. pneumoniae*, *S. enterica* subsp*. enterica* serovar Infantis, *K. aerogenes*, *K. oxytoca*, *S. marcescens*, *K. variicola and E. coli*, all isolated from clinical samples from 2019 to 2020.

**Fig. 5.**
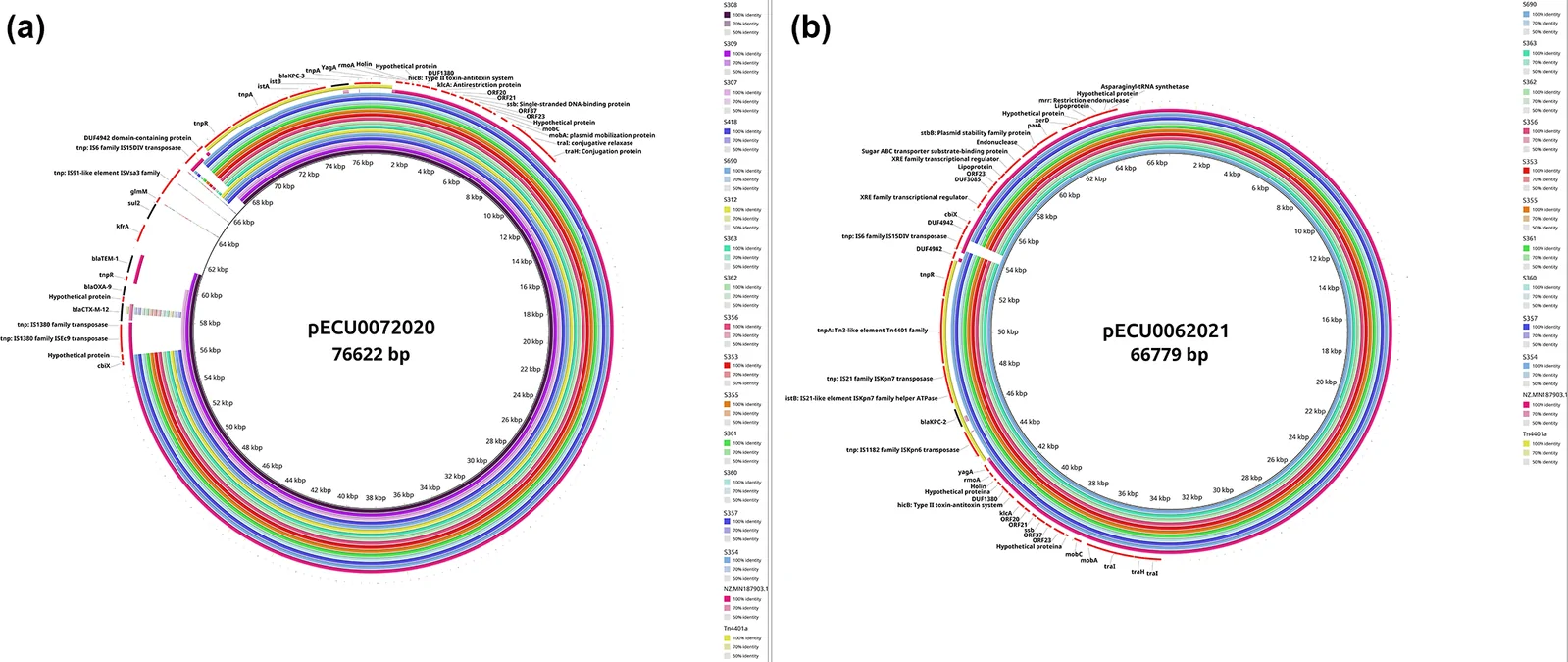
Comparative genomic analysis of the two putative *bla*_KPC_-carrying plasmids reconstructed in this study. Circular alignments of (**a**) pECU0072020 (76,622 bp) and (**b**) pECU0062021 (66,779 bp) against representative plasmid sequences containing Tn*4401*. Concentric rings correspond to individual plasmid alignments, with shading indicating nucleotide identity (100%, 70% and 50%). The outermost track displays the predicted coding sequences, highlighting the *bla*_KPC_-containing Tn*4401* transposon together with genes involved in transposition, conjugation, plasmid maintenance and AMR. Conserved backbone regions are evident across aligned plasmids, whereas structural differences are mainly restricted to accessory regions flanking the transposon.

The largest plasmid, pECU0072020, displayed a complex genetic structure with multiple antibiotic resistance genes, including *bla*_KPC-2_, *bla*_TEM-1_, *bla*_CTX-M-12_, *bla*_OXA-9_, *sul2* and *kfrA* (Fig. 5a), conferring resistance to beta-lactams and sulphonamides. It also carries several mobile genetic elements, such as transposases (*tnpA* and *tnpR*) and ISs (*IS6*, *IS91* and *IS1380*), along with the transposon Tn*4401a*. These genes reside within the IS element cluster/transposon region ‘*tnpR - tnpA - istA/istB - bla*_KPC-2_ *- tnpA*’, which closely resembles the typical Tn*4401* and NTEKPC-II structure. Additional genes related to plasmid maintenance (*glmM* and *hicB*) and hypothetical proteins of unknown function are also present (Fig. 6a).

**Fig. 6.**
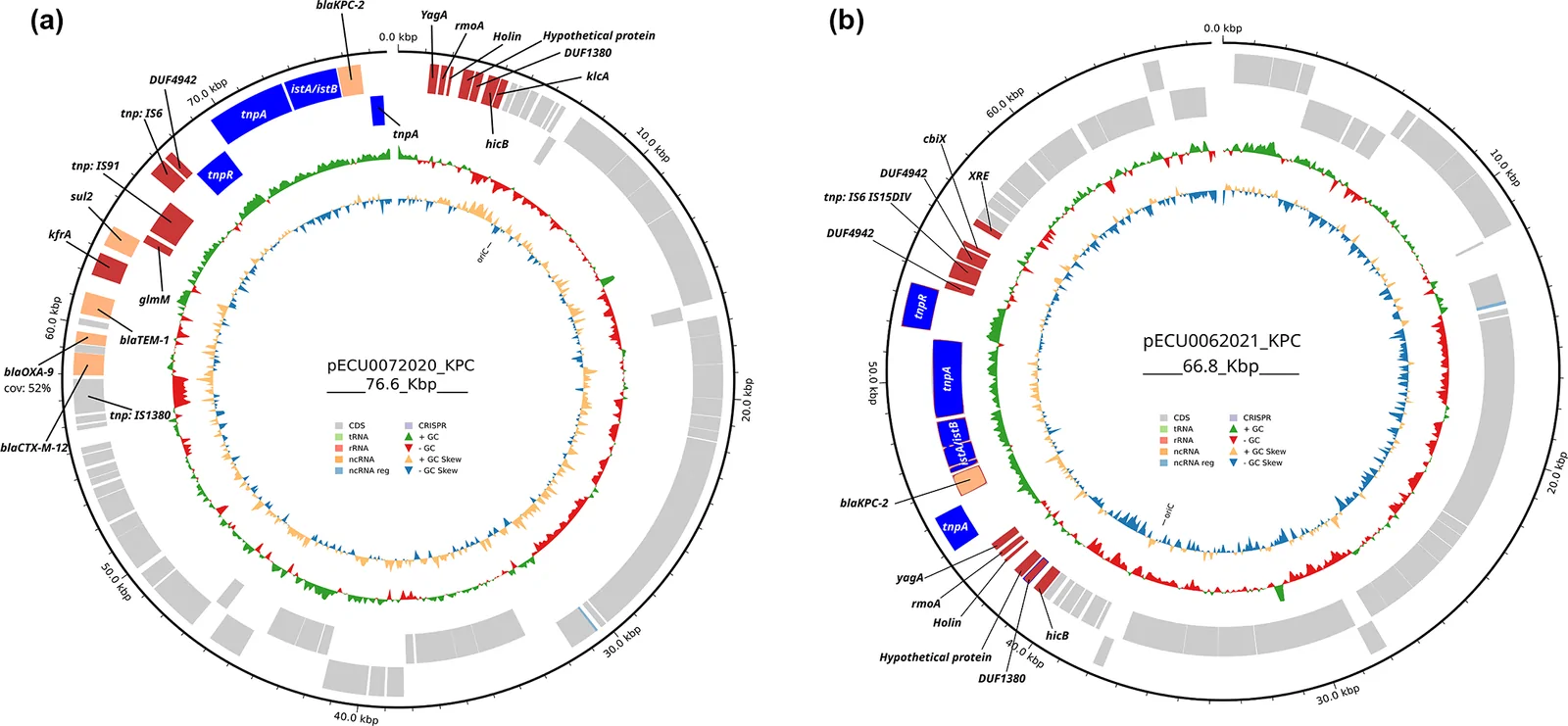
Genetic context of two putative plasmids identified in Ecuadorian clinical isolates harbouring *bla*_KPC-2_. (**a**) pECU0072020 (76.6 kbp, *K. aerogenes*). (b) pECU0062021 (66.8 kbp, *K. oxytoca*).

In contrast, the shorter plasmid, pECU0062021, primarily harbours *bla*_KPC-2_, with 53.16 mol% G+C content. This resistant gene resides within the cluster region organized as ‘*tnpA-bla*_KPC-2_ *-istA/istB- tnpA, tnpR*’, corresponding to a Tn*4401* structure. It also harbours a *hicAB* toxin–antitoxin system, which contributes to plasmid stability and mobility across *Klebsiella* (Fig. 6b). However, it lacks additional accessory resistance genes, limiting its antimicrobial profile to KPC-mediated carbapenem resistance.

Since pECU0072020 harbours a considerably larger repertoire of resistance genes and has no match in current public databases, subsequent analyses focused on this plasmid. The comparison of pECU0072020 with 11 highly similar reference plasmids (Table 1) showed that, in terms of coverage (Fig. S2a), some plasmids, such as OQ821027.1 and CP118215.1, share more than 90% of their sequence, while others, such as OQ821057.1–OQ821070.1, showed partial coverage (~58–62%). However, CP118215.1 carried *bla*_KPC-3_ (Table 1). In parallel, identity analysis (Fig. S2b) revealed that the shared regions have an identity greater than 99%, indicating that, although the total coverage varies, the homologous fragments are highly conserved. These results suggest that pECU0072020 is a mosaic multidrug-resistant plasmid with a unique organization that differs from the previously described plasmids.

**Table 1.** Features of plasmids similar to pECU0072020

ID	Length	Location	Micro-organism	Tn*4401*	*bla*_KPC_	Other AMR genes
NZ_MN187903.1	68453	Argentina	*Citrobacter freundii* strain Cf164	No	No	*bla*_TEM-150_, *bla*_CTX-M-8_
CP011656.1	129196	USA: Virginia	*C. freundii* strain CAV1741	Tn*4401b*	*bla*_KPC-2_	*bla*_TEM-1_, *aac(6’)-Ib4*, *aadA1*, *sul1*, *dfrA3b*, *bla*_SHV-30_
CP037737.1	85575	USA: Charlottesville	*C. freundii* strain CAV1857	Tn*4401b*	*bla*_KPC-2_	*aadA1*, *sul1*, *dfrA3b*, *bla*_SHV-30_, *bla*_TEM-1_
CP094900.1	74544	USA	*K. pneumoniae* strain 2017HL-00503	Tn*4401b*	*bla*_KPC-3_	*bla*_TEM-1_
CP103692.1	81842	USA: Houston	*K. oxytoca* strain ST34	Tn*4401b*	*bla*_KPC-2_	*ant(2’’)-Ia*, *bla*_SHV-105_, *qnrS1*
CP118215.1	70483	USA	*K. oxytoca* strain 2020CK-00213	Tn*4401b*	*bla*_KPC-3_	No
OQ821027.1	66638	Canada	*Enterobacter hormaechei* subsp. *steigerwaltii* strain 04ACRGNY2016	Tn*4401a*	*bla*_KPC-2_	No
OQ821057.1	109773	Canada	*K. pneumoniae* strain 11A1213CRGN071	Tn*4401b*	*bla*_KPC-3_	*dfrA7*, *sul1*, *qnrB2*, *sul1*, *tet(A)*, *ant(2’’)-Ia*, *bla*_SHV-30_
OQ821068.1	109773	Canada	*E. hormaechei* subsp. *steigerwaltii* strain 11A14CRGN027	Tn*4401b*	*bla*_KPC-3_	*dfrA7*, *sul1*, *qnrB2*, *tet(A)*, *ant(2’’)-Ia*, *bla*_SHV-30_
OQ821069.1	109773	Canada	*E. hormaechei* subsp. *steigerwaltii* strain 11A14CRGN034org1	Tn*4401b*	*bla*_KPC-3_	*dfrA7*, *sul1*, *qnrB2*, *tet(A)*, *ant(2’’)-Ia*, *bla*_SHV-30_
OQ821070.1	104052	Canada	*K. pneumoniae* strain 11A14CRGN034org2	Tn*4401b*	*bla*_KPC-3_	*dfrA7*, *sul1*, *tet(A)*, *ant(2’’)-Ia*, *bla*_SHV-30_

Comparison against the NCBI Nucleotide database revealed that the plasmid most closely related to pECU0072020 shared the highest sequence similarity with plasmids identified in clinical isolates of *Klebsiella*, *Citrobacter* and *Enterobacter*. These related plasmids were reported from Canada and the USA, with a single isolate record originating from Argentina (Table 1). Among these similar plasmids, the presence of the Tn*4401* transposon was variable. However, when present, Tn*4401b* was the predominant isoform and was associated with *bla*_KPC-2_ and *bla*_KPC-3_ (Table 1). In contrast, pECU0072020 harbours the Tn*4401a* isoform, indicating a distinct structural configuration compared to the majority of related plasmids.

## Discussion

The emergence of carbapenem resistance genes, specifically *bla*_KPC-2_ and *bla*_KPC-3_, presents a significant challenge in the control of nosocomial infections [2]. These genes, which are responsible for coding carbapenemases, have been thoroughly documented in high-risk *Enterobacteriaceae*, mainly in *K. pneumoniae*, and are associated with resistance to an array of *β*-lactams, including penicillins, carbapenems, cephalosporins, cephamycins and monobactams [10, 35]. Tn*4401*, a transposon classified within the Tn*3* family, has been identified as a crucial mechanism for the mobilization of *bla*_KPC_. Although most global studies have correlated *bla*_KPC_ with Tn*4401a* or its variants in *K. pneumoniae*, contemporary research indicates the emergence of smaller variants, as evidenced by isolates collected in the Caribbean region [36, 37].

In this study, we analysed the first genomic overview of *bla*_KPC*-*2_ and *bla*_KPC*-*3_ distribution, as well as the presence of the transposon elements across 1,021 genomes of ESKAPE pathogens and other high-risk *Enterobacteriaceae* obtained from clinical isolates and healthy individuals of Ecuador [10, 37]. We employed whole-genome-based phylogenomic analyses to assess the relationship between these mobile genetic elements and the spread of resistance in different bacterial lineages. Our comparative analysis between the isolates reported in the One Health study demonstrated that *bla*_KPC_-carrying isolates were not present in the One Health study but could be found in clinical samples. These data suggest that the epidemiology of carbapenemase-producing bacteria in Ecuador may be primarily nosocomial, which could have been potentially driven by the selective pressures of hospital antibiotic use. This aligns with evidence from Colombia and Brazil, where hospital environments act as the primary hubs for *bla*_KPC_ dissemination [38]. This further exemplifies why guidelines for the proper use of antibiotics should be revised and published to prevent the further spread of antibiotic-resistant bacteria.

The analysis performed using Mash distance revealed that 60 of the isolates analysed had either *bla*_KPC-2_ or *bla*_KPC-3_ present, with 40 samples carrying *bla*_KPC-2_ and the remaining 20 carrying *bla*_KPC-3_, demonstrating that *bla*_KPC-2_ has a higher prevalence throughout the samples. Although *K. pneumoniae* represented most *bla*_KPC_-positive isolates, *bla*_KPC-2_ was not restricted to this species. Instead, it was also identified in *K. aerogenes*, *K. variicola*, *K. oxytoca*, *S. marcescens*, *S. enterica* and *E. coli*, highlighting the broad host range of this resistance determinant. Furthermore, it was observed that the isolates harbouring *bla*_KPC-2_ were dominated by major *K. pneumoniae* clones ST11, ST258, ST307, ST152 and ST472. In contrast, clinical isolates carrying *bla*_KPC-3_ were restricted to *K. pneumoniae* of ST22, ST45 and ST45-1LV, all recognized as high-risk clones associated with outbreaks worldwide [10, 36–38]. Our findings complement previous studies conducted in various regions, including the USA, Spain, China and Brazil [39–42], which also demonstrate that *bla*_KPC-2_ and *bla*_KPC-3_ are primarily present in transposable element Tn*4401a*. Here, we also demonstrate that *bla*_KPC-3_ is carried in Ecuador by a novel variant Tn*440l*, reinforcing the importance of genomic surveillance in understanding the mechanisms underlying the global dissemination of carbapenem resistance.

The analysis of mobile genetic elements involved in the dissemination of the *bla*_KPC-2_ and *bla*_KPC-3_ genes showed the relationships between the analysed bacterial genomes, TSD sequences, flanking regions, plasmid replicons and identified Tn*4401* transposons. Different TSD sequences were identified, suggesting multiple insertion events of the transposon in various genomic and plasmidic loci. Regarding the replicons, Tn*4401a* was found in a variety of plasmids containing *bla*_KPC-2_, whereas Tn*4401l*, harbouring *bla*_KPC-3_, was associated with plasmid pBK30683. Our findings also indicate that the flanking regions upstream and downstream of Tn*4401* are not necessarily identical to each other. This demonstrates that the TSD sequences can be diverse, not only within a specific plasmid, but also across different bacterial species. In Ecuador, it has been observed that this transposon can distribute itself within several different bacterial species, having been found in *S. enterica*, *K. pneumoniae*, *K. variicola*, *K. oxytoca*, *K. aerogenes*, *S. marcescens* and *E. coli*. These findings highlight the ability of transposons to disseminate into different species and adapt to different plasmids through varied TSD sequences. The interspecies transmission of this transposon and of NTEs has been reported globally [43], including nosocomial transmission events within a single patient [44].

In Ecuador, the conjugative plasmid pBK30683 (IncFIA) was one of the plasmids carrying *bla*_KPC-3_. While pBK30683 consistently harboured this gene, *bla*_KPC-3_ was also found in other plasmid backbones, indicating multiple vehicles for its dissemination [45]. However, in this study, we describe *bla*_KPC-3_ carried in a Tn*4401l* variant, which has not been formally published but is available in a public database (ID: PP567283.1). Furthermore, we identified an unusual TSD distinct from the common 5 bp TSD (GTTCT) described in different studies [43, 44]. Moreover, several of the *bla*_KPC-2_ strains in Ecuador were also associated with an unusual TSD sequence, AACAG, which differs from the more common TSDs described for other Tn*4401a* variants, indicating variability in target sites across plasmids. This observation is consistent with the findings reported by Cuzon *et al*. [44], who demonstrated that TSD variability depends on the transposon. These findings showed the diversity of transposon structures and insertion patterns associated with *bla*_KPC_ in Ecuador.

Considering that *bla*_KPC-3_ has recently been reported in Ecuador [10], this study highlights the presence of this gene within the country in previous years. This suggests that this gene is highly distributed in clinical isolates throughout the country and that there is a need for an increased monitoring of genetic outbreaks. The presence of *bla*_KPC_ associated with different TSDs and within different transposons could potentially permit the distribution of carbapenemase-resistance genes in Ecuador. In this sense, it is of utmost importance to determine the origin of local outbreaks, as understanding their transmission dynamics would enable the implementation of targeted strategies, including genomic surveillance, strengthened infection control measures and antimicrobial stewardship programmes to reduce their spread. It was found that the TSD was shared across 20 different samples, many of them being from different species, indicating that the transposon was capable of being transferred across species [45]. This implies that carbapenem resistance genes could be potentially transferred to a larger pool of species, raising concern regarding the possibility of genuine epidemiological expansion, which could be linked to local outbreaks or horizontal transfer events, causing a higher number of antibiotic-resistant strains and infections across the country.

Another key finding was the identification of two putative plasmids, pECU0072020 and pECU0062021, carrying *bla*_KPC-2_ within Tn*4401a*. While pECU0062021 harboured only *bla*_KPC-2_ and minimal accessory genes, pECU0072020 has a complex mosaic structure with multiple resistance determinants (*bla*_TEM-1_, *bla*_CTX-M-12_, *bla*_OXA-9_ and *sul2*). This plasmid organization, combined with the lack of a match in the current existing database, suggests the emergence of a novel epidemic plasmid carrying *bla*_KPC-2_ in Ecuador. Additionally, the possibility that there are more plasmids that have not been identified across more bacterial species within and outside of Ecuador underscores the importance of integrating genomic epidemiology into national antimicrobial surveillance to detect emerging variants, find transmission pathways and create targeted control measures.

This study had some limitations. First, most of the genomes analysed were from public databases rather than collected through a prospective surveillance protocol, which reduced the representativity of isolates across Ecuador. Second, the disparity between the number of clinical and non-clinical isolates influences the observed prevalence of *bla*_KPC_ in hospital versus community environments, limiting the approach to determine the spread within the community. The temporal distribution of the available genomes was also uneven, which limited our ability to assess longitudinal trends. Furthermore, our findings are based on genomic analyses without phenotypic validation; therefore, we cannot confirm the reconstructed plasmids. Finally, because genomic surveillance in Ecuador remains limited and publicly available genomes are concentrated in a few major cities, the current dataset does not allow robust assessment of geographic patterns in the distribution of *bla*_KPC_ variants or associated Tn*4401* transposons. Despite these constraints, our study provides the first comprehensive genomic overview of *bla*_KPC_ dissemination in Ecuadorian clinical settings and underscores the need for expanded national genomic surveillance.

## Supplementary material

10.1099/mic.0.001772 Supplementary Material 1.

## Abbreviations

AMR: antimicrobial resistance
ANI: average nucleotide identity
IS: insertion sequences
NTE: non-Tn4401 elements
TSD: extended-spectrum beta-lactamases
TSD: target site duplication
