# Supplementary material for: Genomic characterization of blaKPC transmission via Tn4401a and Tn4401l transposons across Enterobacteriaceae and ESKAPE in Ecuador
Source: Microbiology (Reading). 2026 Oct 15;172(10):001772. doi: 10.1099/mic.0.001772 (PMC13671725; doi:10.1099/mic.0.001772)
Supplement: Supplementary file 1 [file mic-172-01772-s001.pdf]

Supplementary Information

Genomic characterization of *bla*$_{KPC}$ transmission via *Tn4401a* and *Tn4401l* transposons across *Enterobacteriaceae* and ESKAPE in Ecuador

Isaías Mejia-Limones[1]., María Benites-Villegas[1]., María Cáceres-Valdiviezo[1]., Derly Andrade-Molina[1*]

[1]Omics Science Laboratory, Faculty of Health Science, Universidad Espíritu Santo, Ecuador.

***Co-correspondence:** dmandrademolina@uees.edu.ec

Supplementary Table 1. Metadata and general characteristics of genomes

ID	SRA Run	Bioproject	Organism	Recollected	Submmiter	Collection date	Geo loc name	Isolation source	Host	Purpose of sampling	Lat ion	Hospital	Completeness_General	Contamination	1st Criterion	FastANI Reference	# contigs	Total length	GC (%)	N50	2nd Criterion	FastANI (%)	3rd Criterion	Failed criteria
SAMN33842142	SRR26135156	PRJNA946810	*Pseudomonas aeruginosa*	Gabriela Vasco	Universidad Central del Ecuador,	2022-01-31	Quito, Ecuador	Rectal swab	Homo sapiens	Diseased	0.28671686 S 78.55016137 W	Hospital Padre Carollo "Un Canto a la Vida"	86.64	3.03	fail	Pseudomona_ASM676v1	7	7219627	65.75	1591517	pass	98.616	pass	1
SAMN33842141	SRR26135157	PRJNA946810	*Pseudomonas aeruginosa*	Gabriela Vasco	Universidad Central del Ecuador,	2021-12-04	Quito, Ecuador	Tracheal discharge	Homo sapiens	Diseased	0.28671686 S 78.55016137 W	Hospital Padre Carollo "Un Canto a la Vida"	92.84	4.22	pass	Pseudomona_ASM676v1	16	7659171	65.91	1572104	pass	98.7088	pass	0
SAMN33842140	SRR26135158	PRJNA946810	*Pseudomonas aeruginosa*	Gabriela Vasco	Universidad Central del Ecuador,	2021-12-14	Quito, Ecuador	Rectal swab	Homo sapiens	Diseased	0.28671686 S 78.55016137 W	Hospital Padre Carollo "Un Canto a la Vida"	90.18	1.23	pass	Pseudomona_ASM676v1	1	6975849	65.86	6975849	pass	98.695	pass	0
SAMN33842139	SRR26135159	PRJNA946810	*Pseudomonas aeruginosa*	Gabriela Vasco	Universidad Central del Ecuador,	2021-11-24	Quito, Ecuador	Rectal swab	Homo sapiens	Diseased	0.28671686 S 78.55016137 W	Hospital Padre Carollo "Un Canto a la Vida"	67.87	3.46	fail	Pseudomona_ASM676v1	36	4741285	66.74	268703	pass	99.1326	pass	1
SAMN33845195	SRR26135160	PRJNA946810	*Pseudomonas aeruginosa*	Gabriela Vasco	Universidad Central del Ecuador,	2021-12-14	Quito, Ecuador	Rectal swab	Homo sapiens	Diseased	0.28671686 S 78.55016137 W	Hospital Padre Carollo "Un Canto a la Vida"	88.44	0.72	fail	Pseudomona_ASM676v1	3	7042644	65.84	4942797	pass	98.7227	pass	1
SAMN33845194	SRR26135161	PRJNA946810	*Pseudomonas aeruginosa*	Gabriela Vasco	Universidad Central del Ecuador,	2021-12-14	Quito, Ecuador	Rectal swab	Homo sapiens	Diseased	0.28671686 S 78.55016137 W	Hospital Padre Carollo "Un Canto a la Vida"	89.94	0.72	fail	Pseudomona_ASM676v1	1	6971932	65.86	6971932	pass	98.7	pass	1
SAMN33842186	SRR26135162	PRJNA946810	*Pseudomonas aeruginosa*	Gabriela Vasco	Universidad Central del Ecuador,	2022-10-26	Quito, Ecuador	Rectal swab	Homo sapiens	Diseased	0.28671686 S 78.55016137 W	Hospital Padre Carollo "Un Canto a la Vida"	90.31	1.9	pass	Pseudomona_ASM676v1	33	5908185	66.46	323296	pass	99.2471	pass	0
SAMN33842185	SRR26135163	PRJNA946810	*Pseudomonas aeruginosa*	Gabriela Vasco	Universidad Central del Ecuador,	2022-10-26	Quito, Ecuador	wound	Homo sapiens	Diseased	0.28671686 S 78.55016137 W	Hospital Padre Carollo "Un Canto a la Vida"	96.36	2.29	pass	Pseudomona_ASM676v1	1	6456439	66.42	6456439	pass	99.293	pass	0
SAMN33842184	SRR26135164	PRJNA946810	*Pseudomonas aeruginosa*	Gabriela Vasco	Universidad Central del Ecuador,	2022-09-18	Quito, Ecuador	urine	Homo sapiens	Diseased	0.28671686 S 78.55016137 W	Hospital Padre Carollo "Un Canto a la Vida"	90.3	2.19	pass	Pseudomona_ASM676v1	19	6237920	66.3	538245	pass	99.0844	pass	0
SAMN33842183	SRR26135165	PRJNA946810	*Pseudomonas aeruginosa*	Gabriela Vasco	Universidad Central del Ecuador,	2022-09-19	Quito, Ecuador	Rectal swab	Homo sapiens	Diseased	0.28671686 S 78.55016137 W	Hospital Padre Carollo "Un Canto a la Vida"	72.97	0.88	fail	Pseudomona_ASM676v1	58	5063570	66.46	107258	pass	98.9628	pass	1
SAMN33842138	SRR26135166	PRJNA946810	*Pseudomonas aeruginosa*	Gabriela Vasco	Universidad Central del Ecuador,	2021-11-15	Quito, Ecuador	Rectal swab	Homo sapiens	Diseased	0.28671686 S 78.55016137 W	Hospital Padre Carollo "Un Canto a la Vida"	89.61	1.34	fail	Pseudomona_ASM676v1	1	6432124	66.37	6432124	pass	99.2422	pass	1
SAMN33842182	SRR26135167	PRJNA946810	*Pseudomonas aeruginosa*	Gabriela Vasco	Universidad Central del Ecuador,	2022-09-19	Quito, Ecuador	Rectal swab	Homo sapiens	Diseased	0.28671686 S 78.55016137 W	Hospital Padre Carollo "Un Canto a la Vida"	74.5	3.4	fail	Pseudomona_ASM676v1	24	6109883	66.37	600555	pass	99.0527	pass	1
SAMN33842181	SRR26135168	PRJNA946810	*Pseudomonas aeruginosa*	Gabriela Vasco	Universidad Central del Ecuador,	2022-09-19	Quito, Ecuador	wound	Homo sapiens	Diseased	0.28671686 S 78.55016137 W	Hospital Padre Carollo "Un Canto a la Vida"	95.09	1.26	pass	Pseudomona_ASM676v1	2	6480238	66.35	3792232	pass	99.1546	pass	0
SAMN33842180	SRR26135169	PRJNA946810	*Pseudomonas aeruginosa*	Gabriela Vasco	Universidad Central del Ecuador,	2022-09-13	Quito, Ecuador	Rectal swab	Homo sapiens	Diseased	0.28671686 S 78.55016137 W	Hospital Padre Carollo "Un Canto a la Vida"	62.09	0.99	fail	Pseudomona_ASM676v1	61	4017662	66.42	80398	pass	98.5219	pass	1
SAMN33842179	SRR26135170	PRJNA946810	*Pseudomonas aeruginosa*	Gabriela Vasco	Universidad Central del Ecuador,	2022-09-13	Quito, Ecuador	Rectal swab	Homo sapiens	Diseased	0.28671686 S 78.55016137 W	Hospital Padre Carollo "Un Canto a la Vida"	9.29	0.21	fail	Pseudomona_ASM676v1	26	861696	67.89	37472	fail	96.6772	pass	2

ID	SRA Run	Bioproject	Organism	Recollected	Submmiter	Collection date	Geo loc name	Isolation source	Host	Purpose of sampling	Lat ion	Hospital	Completeness_General	Contamination	1st Criterion	FastANI Reference	# contigs	Total length	GC (%)	N50	2nd Criterion	FastANI (%)	3rd Criterion	Failed criteria
SAMN33842178	SRR26135171	PRJNA946810	*Pseudomonas aeruginosa*	Gabriela Vasco	Universidad Central del Ecuador,	2022-09-13	Quito, Ecuador	Femur	Homo sapiens	Diseased	0.28671686 S 78.55016137 W	Hospital Padre Carollo "Un Canto a la Vida"	94.52	0.66	pass	Pseudomona_ASM676v1	6	6410340	66.49	3687367	pass	98.7712	pass	0
SAMN33842177	SRR26135172	PRJNA946810	*Pseudomonas aeruginosa*	Gabriela Vasco	Universidad Central del Ecuador,	2022-09-13	Quito, Ecuador	wound	Homo sapiens	Diseased	0.28671686 S 78.55016137 W	Hospital Padre Carollo "Un Canto a la Vida"	94.23	1.36	pass	Pseudomona_ASM676v1	14	6238816	66.47	665682	pass	98.7612	pass	0
SAMN33842176	SRR26135173	PRJNA946810	*Pseudomonas aeruginosa*	Gabriela Vasco	Universidad Central del Ecuador,	2022-09-13	Quito, Ecuador	acetabulum	Homo sapiens	Diseased	0.28671686 S 78.55016137 W	Hospital Padre Carollo "Un Canto a la Vida"	96.12	0.71	pass	Pseudomona_ASM676v1	42	6372066	66.48	207966	pass	98.7457	pass	0
SAMN33842175	SRR26135174	PRJNA946810	*Pseudomonas aeruginosa*	Gabriela Vasco	Universidad Central del Ecuador,	2022-08-26	Quito, Ecuador	urine	Homo sapiens	Diseased	0.28671686 S 78.55016137 W	Hospital Padre Carollo "Un Canto a la Vida"	70.64	0.24	fail	Pseudomona_ASM676v1	52	5107270	65.7	105889	pass	98.4493	pass	1
SAMN33842174	SRR26135175	PRJNA946810	*Pseudomonas aeruginosa*	Gabriela Vasco	Universidad Central del Ecuador,	2022-08-26	Quito, Ecuador	Rectal swab	Homo sapiens	Diseased	0.28671686 S 78.55016137 W	Hospital Padre Carollo "Un Canto a la Vida"	93.59	0.8	pass	Pseudomona_ASM676v1	33	6786949	65.8	318696	pass	98.6959	pass	0
SAMN33842173	SRR26135176	PRJNA946810	*Pseudomonas aeruginosa*	Gabriela Vasco	Universidad Central del Ecuador,	2022-08-26	Quito, Ecuador	Rectal swab	Homo sapiens	Diseased	0.28671686 S 78.55016137 W	Hospital Padre Carollo "Un Canto a la Vida"	95.9	0.32	pass	Pseudomona_ASM676v1	11	7160404	65.77	1357878	pass	98.7112	pass	0
SAMN33842137	SRR26135177	PRJNA946810	*Pseudomonas aeruginosa*	Gabriela Vasco	Universidad Central del Ecuador,	2021-11-22	Quito, Ecuador	Abdominal discharge	Homo sapiens	Diseased	0.28671686 S 78.55016137 W	Hospital Padre Carollo "Un Canto a la Vida"	82.41	2	fail	Pseudomona_ASM676v1	37	5440848	66.25	268296	pass	99.2155	pass	1
SAMN33842172	SRR26135178	PRJNA946810	*Pseudomonas aeruginosa*	Gabriela Vasco	Universidad Central del Ecuador,	2022-08-09	Quito, Ecuador	wound	Homo sapiens	Diseased	0.28671686 S 78.55016137 W	Hospital Padre Carollo "Un Canto a la Vida"	84.03	0.59	fail	Pseudomona_ASM676v1	49	6076090	65.69	173329	pass	98.6386	pass	1
SAMN33842171	SRR26135179	PRJNA946810	*Pseudomonas aeruginosa*	Gabriela Vasco	Universidad Central del Ecuador,	2022-05-17	Quito, Ecuador	Rectal swab	Homo sapiens	Diseased	0.28671686 S 78.55016137 W	Hospital Padre Carollo "Un Canto a la Vida"	98.78	29.39	fail	Pseudomona_ASM676v1	48	9376875	58.09	477546	pass	98.7267	pass	1
SAMN33842170	SRR26135180	PRJNA946810	*Pseudomonas aeruginosa*	Gabriela Vasco	Universidad Central del Ecuador,	2022-05-16	Quito, Ecuador	blood	Homo sapiens	Diseased	0.28671686 S 78.55016137 W	Hospital Padre Carollo "Un Canto a la Vida"	33.51	0.83	fail	Pseudomona_ASM676v1	73	2395588	65.36	37863	fail	98.3305	pass	2
SAMN33842169	SRR26135181	PRJNA946810	*Pseudomonas aeruginosa*	Gabriela Vasco	Universidad Central del Ecuador,	2022-07-13	Quito, Ecuador	urine	Homo sapiens	Diseased	0.28671686 S 78.55016137 W	Hospital Padre Carollo "Un Canto a la Vida"	62.95	0.29	fail	Pseudomona_ASM676v1	76	4281791	66.09	64935	pass	98.972	pass	1
SAMN33842168	SRR26135182	PRJNA946810	*Pseudomonas aeruginosa*	Gabriela Vasco	Universidad Central del Ecuador,	2022-07-16	Quito, Ecuador	Rectal swab	Homo sapiens	Diseased	0.28671686 S 78.55016137 W	Hospital Padre Carollo "Un Canto a la Vida"	58.51	1.32	fail	Pseudomona_ASM676v1	55	3923614	66.09	80670	pass	98.8566	pass	1
SAMN33842167	SRR26135183	PRJNA946810	*Pseudomonas aeruginosa*	Gabriela Vasco	Universidad Central del Ecuador,	2022-07-16	Quito, Ecuador	Rectal swab	Homo sapiens	Diseased	0.28671686 S 78.55016137 W	Hospital Padre Carollo "Un Canto a la Vida"	95.42	2.14	pass	Pseudomona_ASM676v1	13	6439699	66.32	929144	pass	99.1607	pass	0
SAMN33842166	SRR26135184	PRJNA946810	*Pseudomonas aeruginosa*	Gabriela Vasco	Universidad Central del Ecuador,	2022-07-16	Quito, Ecuador	Rectal swab	Homo sapiens	Diseased	0.28671686 S 78.55016137 W	Hospital Padre Carollo "Un Canto a la Vida"	20.22	0.34	fail	Pseudomona_ASM676v1	33	1328834	66.51	52732	pass	97.8822	pass	1
SAMN33842165	SRR26135185	PRJNA946810	*Pseudomonas aeruginosa*	Gabriela Vasco	Universidad Central del Ecuador,	2022-07-16	Quito, Ecuador	Rectal swab	Homo sapiens	Diseased	0.28671686 S 78.55016137 W	Hospital Padre Carollo "Un Canto a la Vida"	40.03	1.01	fail	Pseudomona_ASM676v1	63	2851292	66.3	54337	pass	98.8552	pass	1

ID	SRA Run	Bioproject	Organism	Recollected	Submmiter	Collection date	Geo loc name	Isolation source	Host	Purpose of sampling	Lat ion	Hospital	Completeness_General	Contamination	1st Criterion	FastANI Reference	# contigs	Total length	GC (%)	N50	2nd Criterion	FastANI (%)	3rd Criterion	Failed criteria
SAMN33842164	SRR26135186	PRJNA946810	*Pseudomonas aeruginosa*	Gabriela Vasco	Universidad Central del Ecuador,	2022-07-16	Quito, Ecuador	Rectal swab	Homo sapiens	Diseased	0.28671686 S 78.55016137 W	Hospital Padre Carollo "Un Canto a la Vida"	96.78	0.89	pass	Pseudomona_ASM676v1	32	6354230	66.32	289950	pass	99.1769	pass	0
SAMN33842163	SRR26135187	PRJNA946810	*Pseudomonas aeruginosa*	Gabriela Vasco	Universidad Central del Ecuador,	2022-05-20	Quito, Ecuador	Tracheal discharge	Homo sapiens	Diseased	0.28671686 S 78.55016137 W	Hospital Padre Carollo "Un Canto a la Vida"	91.67	0.44	pass	Pseudomona_ASM676v1	35	6750376	65.74	325541	pass	98.6578	pass	0
SAMN33842136	SRR26135188	PRJNA946810	*Pseudomonas aeruginosa*	Gabriela Vasco	Universidad Central del Ecuador,	2021-11-11	Quito, Ecuador	Abdominal Wound	Homo sapiens	Diseased	0.28671686 S 78.55016137 W	Hospital Padre Carollo "Un Canto a la Vida"	94.94	1.29	pass	Pseudomona_ASM676v1	1	6432884	66.37	6432884	pass	99.2476	pass	0
SAMN33842162	SRR26135189	PRJNA946810	*Pseudomonas aeruginosa*	Gabriela Vasco	Universidad Central del Ecuador,	2022-05-24	Quito, Ecuador	Rectal swab	Homo sapiens	Diseased	0.28671686 S 78.55016137 W	Hospital Padre Carollo "Un Canto a la Vida"	73.9	1.29	fail	Pseudomona_ASM676v1	45	5497805	65.91	159536	pass	98.5833	pass	1
SAMN33842161	SRR26135190	PRJNA946810	*Pseudomonas aeruginosa*	Gabriela Vasco	Universidad Central del Ecuador,	2022-03-21	Quito, Ecuador	Tracheal discharge	Homo sapiens	Diseased	0.28671686 S 78.55016137 W	Hospital Padre Carollo "Un Canto a la Vida"	91.08	2.76	pass	Pseudomona_ASM676v1	2	6796536	65.97	6780563	pass	98.5099	pass	0
SAMN33842160	SRR26135191	PRJNA946810	*Pseudomonas aeruginosa*	Gabriela Vasco	Universidad Central del Ecuador,	2022-03-24	Quito, Ecuador	Rectal swab	Homo sapiens	Diseased	0.28671686 S 78.55016137 W	Hospital Padre Carollo "Un Canto a la Vida"	89.2	1.43	fail	Pseudomona_ASM676v1	4	7061906	66.03	6727357	pass	98.5325	pass	1
SAMN33842159	SRR26135192	PRJNA946810	*Pseudomonas aeruginosa*	Gabriela Vasco	Universidad Central del Ecuador,	2022-03-24	Quito, Ecuador	Rectal swab	Homo sapiens	Diseased	0.28671686 S 78.55016137 W	Hospital Padre Carollo "Un Canto a la Vida"	87.24	0.4	fail	Pseudomona_ASM676v1	2	6854106	65.98	6838132	pass	98.5027	pass	1
SAMN33842158	SRR26135193	PRJNA946810	*Pseudomonas aeruginosa*	Gabriela Vasco	Universidad Central del Ecuador,	2022-03-03	Quito, Ecuador	Rectal swab	Homo sapiens	Diseased	0.28671686 S 78.55016137 W	Hospital Padre Carollo "Un Canto a la Vida"	89.04	1.5	fail	Pseudomona_ASM676v1	2	7036899	65.75	6601712	pass	99.2291	pass	1
SAMN33842157	SRR26135194	PRJNA946810	*Pseudomonas aeruginosa*	Gabriela Vasco	Universidad Central del Ecuador,	2022-03-03	Quito, Ecuador	Rectal swab	Homo sapiens	Diseased	0.28671686 S 78.55016137 W	Hospital Padre Carollo "Un Canto a la Vida"	79.42	2.28	fail	Pseudomona_ASM676v1	13	6920091	65.78	3460516	pass	99.1502	pass	1
SAMN33842156	SRR26135195	PRJNA946810	*Pseudomonas aeruginosa*	Gabriela Vasco	Universidad Central del Ecuador,	2022-03-09	Quito, Ecuador	Wound	Homo sapiens	Diseased	0.28671686 S 78.55016137 W	Hospital Padre Carollo "Un Canto a la Vida"	96.55	3.75	pass	Pseudomona_ASM676v1	6	7010602	65.73	1994562	pass	99.2654	pass	0
SAMN33842155	SRR26135196	PRJNA946810	*Pseudomonas aeruginosa*	Gabriela Vasco	Universidad Central del Ecuador,	2022-03-10	Quito, Ecuador	Rectal swab	Homo sapiens	Diseased	0.28671686 S 78.55016137 W	Hospital Padre Carollo "Un Canto a la Vida"	81.86	2.78	fail	Pseudomona_ASM676v1	13	6953086	66.08	631069	pass	99.0964	pass	1
SAMN33842154	SRR26135197	PRJNA946810	*Pseudomonas aeruginosa*	Gabriela Vasco	Universidad Central del Ecuador,	2022-03-10	Quito, Ecuador	Rectal swab	Homo sapiens	Diseased	0.28671686 S 78.55016137 W	Hospital Padre Carollo "Un Canto a la Vida"	85.24	3.77	fail	Pseudomona_ASM676v1	17	6627672	66.11	537759	pass	99.093	pass	1
SAMN33842153	SRR26135198	PRJNA946810	*Pseudomonas aeruginosa*	Gabriela Vasco	Universidad Central del Ecuador,	2022-03-10	Quito, Ecuador	Rectal swab	Homo sapiens	Diseased	0.28671686 S 78.55016137 W	Hospital Padre Carollo "Un Canto a la Vida"	84.25	4.26	fail	Pseudomona_ASM676v1	18	6654518	66.15	571648	pass	99.0714	pass	1
SAMN33842152	SRR26135200	PRJNA946810	*Pseudomonas aeruginosa*	Gabriela Vasco	Universidad Central del Ecuador,	2022-03-07	Quito, Ecuador	Blood	Homo sapiens	Diseased	0.28671686 S 78.55016137 W	Hospital Padre Carollo "Un Canto a la Vida"	82.24	3.34	fail	Pseudomona_ASM676v1	43	6562755	66.25	225191	pass	99.0851	pass	1
SAMN33842151	SRR26135201	PRJNA946810	*Pseudomonas aeruginosa*	Gabriela Vasco	Universidad Central del Ecuador,	2022-02-11	Quito, Ecuador	Tracheostome secretion	Homo sapiens	Diseased	0.28671686 S 78.55016137 W	Hospital Padre Carollo "Un Canto a la Vida"	88.64	0.56	fail	Pseudomona_ASM676v1	1	6564799	66.35	6564799	pass	99.1878	pass	1

ID	SRA Run	Bioproject	Organism	Recollected	Submmiter	Collection date	Geo loc name	Isolation source	Host	Purpose of sampling	Lat ion	Hospital	Completeness_General	Contamination	1st Criterion	FastANI Reference	# contigs	Total length	GC (%)	N50	2nd Criterion	FastANI (%)	3rd Criterion	Failed criteria
SAMN33842150	SRR26135202	PRJNA946810	*Pseudomonas aeruginosa*	Gabriela Vasco	Universidad Central del Ecuador,	2022-02-14	Quito, Ecuador	Rectal swab	Homo sapiens	Diseased	0.28671686 S 78.55016137 W	Hospital Padre Carollo "Un Canto a la Vida"	70.54	2.86	fail	Pseudomona_ASM676v1	16	6416322	66.34	856635	pass	99.0497	pass	1
SAMN33842149	SRR26135203	PRJNA946810	*Pseudomonas aeruginosa*	Gabriela Vasco	Universidad Central del Ecuador,	2022-02-14	Quito, Ecuador	Rectal swab	Homo sapiens	Diseased	0.28671686 S 78.55016137 W	Hospital Padre Carollo "Un Canto a la Vida"	54.23	2.54	fail	Pseudomona_ASM676v1	42	4169639	66.65	130235	pass	98.9039	pass	1
SAMN33842148	SRR26135204	PRJNA946810	*Pseudomonas aeruginosa*	Gabriela Vasco	Universidad Central del Ecuador,	2022-01-18	Quito, Ecuador	Sputum	Homo sapiens	Diseased	0.28671686 S 78.55016137 W	Hospital Padre Carollo "Un Canto a la Vida"	89.57	2.01	fail	Pseudomona_ASM676v1	1	6526160	66.29	6526160	pass	99.1742	pass	1
SAMN33842147	SRR26135205	PRJNA946810	*Pseudomonas aeruginosa*	Gabriela Vasco	Universidad Central del Ecuador,	2022-01-18	Quito, Ecuador	Rectal swab	Homo sapiens	Diseased	0.28671686 S 78.55016137 W	Hospital Padre Carollo "Un Canto a la Vida"	91.36	3.35	pass	Pseudomona_ASM676v1	8	6555316	66.34	757647	pass	99.1171	pass	0
SAMN33842146	SRR26135206	PRJNA946810	*Pseudomonas aeruginosa*	Gabriela Vasco	Universidad Central del Ecuador,	2022-01-18	Quito, Ecuador	Rectal swab	Homo sapiens	Diseased	0.28671686 S 78.55016137 W	Hospital Padre Carollo "Un Canto a la Vida"	92.56	2.54	pass	Pseudomona_ASM676v1	6	6788838	66.18	2978559	pass	99.1249	pass	0
SAMN33842145	SRR26135207	PRJNA946810	*Pseudomonas aeruginosa*	Gabriela Vasco	Universidad Central del Ecuador,	2022-01-18	Quito, Ecuador	Rectal swab	Homo sapiens	Diseased	0.28671686 S 78.55016137 W	Hospital Padre Carollo "Un Canto a la Vida"	89.55	2.57	fail	Pseudomona_ASM676v1	8	6796865	66.1	3785714	pass	99.1322	pass	1
SAMN33842144	SRR26135208	PRJNA946810	*Pseudomonas aeruginosa*	Gabriela Vasco	Universidad Central del Ecuador,	2022-01-18	Quito, Ecuador	Rectal swab	Homo sapiens	Diseased	0.28671686 S 78.55016137 W	Hospital Padre Carollo "Un Canto a la Vida"	65.72	2.13	fail	Pseudomona_ASM676v1	35	5539481	66.07	211947	pass	98.7899	pass	1
SAMN33842143	SRR26135209	PRJNA946810	*Pseudomonas aeruginosa*	Gabriela Vasco	Universidad Central del Ecuador,	2022-01-28	Quito, Ecuador	Sputum	Homo sapiens	Diseased	0.28671686 S 78.55016137 W	Hospital Padre Carollo "Un Canto a la Vida"	77.65	0.33	fail	Pseudomona_ASM676v1	38	5710438	65.86	186452	pass	98.5774	pass	1
SAMEA111504297	ERS13616659	PRJEB56918	*Pseudomonas aeruginosa*	Teodoro Maldonado Carbo Hospital	EBI	2020-08-13	Guayas, Guayaquil, Ecuador	unspecified diagnostic sample	Homo sapiens	Diseased	2.203816 S 79.897453 W	Teodoro Maldonado Carbo Hospital	99.72	0.3	pass	Pseudomona_ASM676v1	357	6465562	66.35	29174	fail	98.6766	pass	1
SAMEA111504298	ERS13616660	PRJNA514245	*Pseudomonas aeruginosa*	Eugenio Espejo Hospital	EBI	2020-08-21	Quito, Ecuador	aspirate	Homo sapiens	Diseased	0.180653 S 78.467834 W	Eugenio Espejo Hospital	98.73	0.2	pass	Pseudomona_ASM676v1	269	6251728	66.6	38032	fail	99.2333	pass	1
SAMEA111504299	ERS13616661	PRJNA514245	*Acinetobacter baumannii*	Eugenio Espejo Hospital	EBI	2020-08-05	Quito, Ecuador	aspirate	Homo sapiens	Diseased	0.180653 S 78.467834 W	Eugenio Espejo Hospital	100	0.32	pass	Acinetobacter_ASM903584v1	103	4003976	38.99	147031	pass	97.9682	pass	0
SAMEA111504300	ERS13616662	PRJNA514245	*Acinetobacter baumannii*	Eugenio Espejo Hospital	EBI	2020-08-13	Quito, Ecuador	blood	Homo sapiens	Diseased	0.180653 S 78.467834 W	Eugenio Espejo Hospital	100	0.38	pass	Acinetobacter_ASM903584v1	94	4068310	38.9	102358	pass	97.7252	pass	0
SAMEA111504301	ERS13616663	PRJNA514245	*Acinetobacter baumannii*	Eugenio Espejo Hospital	EBI	2020-08-13	Quito, Ecuador	aspirate	Homo sapiens	Diseased	0.180653 S 78.467834 W	Eugenio Espejo Hospital	100	0.32	pass	Acinetobacter_ASM903584v1	92	4008085	38.99	157519	pass	97.916	pass	0
SAMEA111504304	ERS13616666	PRJNA514245	*Acinetobacter baumannii*	Eugenio Espejo Hospital	EBI	2020-08-16	Quito, Ecuador	blood	Homo sapiens	Diseased	0.180653 S 78.467834 W	Eugenio Espejo Hospital	100	0.32	pass	Acinetobacter_ASM903584v1	94	4008871	39	147031	pass	97.9351	pass	0
SAMEA111504302	ERS13616664	PRJNA514245	*Acinetobacter baumannii*	Eugenio Espejo Hospital	EBI	2020-08-13	Quito, Ecuador	aspirate	Homo sapiens	Diseased	0.180653 S 78.467834 W	Eugenio Espejo Hospital	100	0.32	pass	Acinetobacter_ASM903584v1	90	4008387	38.98	157189	pass	97.905	pass	0
SAMEA111504303	ERS13616665	PRJNA514245	*Acinetobacter baumannii*	Carlos Andrade Marín Hospital	EBI	2020-08-14	Quito, Ecuador	swab (superficial and non-surgical)	Homo sapiens	Diseased	0.180653 S 78.467834 W	Carlos Andrade Marín Hospital	100	0.54	pass	Acinetobacter_ASM903584v1	104	4099432	38.93	83863	pass	97.7546	pass	0
SAMEA111504305	ERS13616667	PRJNA514245	*Acinetobacter baumannii*	Eugenio Espejo Hospital	EBI	2020-08-20	Quito, Ecuador	aspirate	Homo sapiens	Diseased	0.180653 S 78.467834 W	Eugenio Espejo Hospital	100	0.31	pass	Acinetobacter_ASM903584v1	102	4007284	39	110967	pass	97.8932	pass	0
SAMEA111504306	ERS13616668	PRJNA514245	*Acinetobacter baumannii*	Eugenio Espejo Hospital	EBI	2020-08-25	Quito, Ecuador	aspirate	Homo sapiens	Diseased	0.180653 S 78.467834 W	Eugenio Espejo Hospital	100	0.3	pass	Acinetobacter_ASM903584v1	129	4001411	39.01	88365	pass	97.9114	pass	0
SAMEA111504354	ERR10437331	PRJEB56918	*Klebsiella pneumoniae*	Eugenio Espejo Hospital	National Center for Biotechnology Information	2020-08-22	Quito, Ecuador	blood	Homo sapiens	Diseased	0.180653 S 78.467834 W	Eugenio Espejo Hospital	97.91	0.13	pass	K_pneumoniae_ASM24018v2	96	5525529	57.17	109070	pass	99.0357	pass	0

ID	SRA Run	Bioproject	Organism	Recollected	Submmiter	Collection date	Geo loc name	Isolation source	Host	Purpose of sampling	Lat ion	Hospital	Completeness_General	Contamination	1st Criterion	FastANI Reference	# contigs	Total length	GC (%)	N50	2nd Criterion	FastANI (%)	3rd Criterion	Failed criteria
SAMEA111504359	ERR10437357	PRJEB56918	*Klebsiella pneumoniae*	Eugenio Espejo Hospital	National Center for Biotechnology Information	2020-08-25	Quito, Ecuador	aspirate	Homo sapiens	Diseased	0.180653 S 78.467834 W	Eugenio Espejo Hospital	98.96	0.02	pass	K_pneumoniae_ASM24018v2	126	5375616	57.5	98152	pass	99.0496	pass	0
SAMEA111504343	ERR10437269	PRJEB56918	*Klebsiella pneumoniae*	Carlos Andrade Marín Hospital	National Center for Biotechnology Information	2020-08-17	Quito, Ecuador	blood	Homo sapiens	Diseased	0.180653 S 78.467834 W	Carlos Andrade Marín Hospital	98.91	0.83	pass	K_pneumoniae_ASM24018v2	135	5582050	57.24	129806	pass	99.6448	pass	0
SAMEA111504317	ERR10437162	PRJEB56918	*Klebsiella pneumoniae*	Riobamba Teaching Hospital	National Center for Biotechnology Information	2020-08-07	Riobamba, Ecuador	blood	Homo sapiens	Diseased		Riobamba Teaching Hospital	98.85	0.47	pass	K_pneumoniae_ASM24018v2	120	5639139	57.04	122989	pass	99.454	pass	0
SAMEA111504352	ERR10437338	PRJEB56918	*Klebsiella pneumoniae*	Carlos Andrade Marín Hospital	National Center for Biotechnology Information	2020-08-20	Quito, Ecuador	aspirate	Homo sapiens	Diseased	0.180653 S 78.467834 W	Carlos Andrade Marín Hospital	98.91	0.83	pass	K_pneumoniae_ASM24018v2	135	5581461	57.24	98903	pass	99.6404	pass	0
SAMEA111504355	ERR10437335	PRJEB56918	*Klebsiella pneumoniae*	Eugenio Espejo Hospital	National Center for Biotechnology Information	2020-08-23	Quito, Ecuador	urine	Homo sapiens	Diseased	0.180653 S 78.467834 W	Eugenio Espejo Hospital	96.35	0.17	pass	K_pneumoniae_ASM24018v2	81	5491665	57.19	162033	pass	99.0606	pass	0
SAMEA111504342	ERR10437267	PRJEB56918	*Klebsiella pneumoniae*	Carlos Andrade Marín Hospital	National Center for Biotechnology Information	2020-08-17	Quito, Ecuador	aspirate	Homo sapiens	Diseased	0.180653 S 78.467834 W	Carlos Andrade Marín Hospital	98.91	0.83	pass	K_pneumoniae_ASM24018v2	146	5578903	57.24	98899	pass	99.6671	pass	0
SAMEA111504328	ERR10437211	PRJEB56918	*Klebsiella pneumoniae*	Teodoro Maldonado Carbo Hospital	National Center for Biotechnology Information	2020-08-13	Guayas, Guayaquil, Ecuador	blood	Homo sapiens	Diseased	2.203816 S 79.897453 W	Teodoro Maldonado Carbo Hospital	97.6	0.13	pass	K_pneumoniae_ASM24018v2	121	5406701	57.34	86844	pass	99.097	pass	0
SAMEA111504322	ERR10437194	PRJEB56918	*Klebsiella pneumoniae*	Carlos Andrade Marín Hospital	National Center for Biotechnology Information	2020-08-11	Quito, Ecuador	urine	Homo sapiens	Diseased	0.180653 S 78.467834 W	Carlos Andrade Marín Hospital	98.91	0.8	pass	K_pneumoniae_ASM24018v2	131	5580648	57.24	162737	pass	99.6736	pass	0
SAMEA111504313	ERR10437152	PRJEB56918	*Klebsiella pneumoniae*	Eugenio Espejo Hospital	National Center for Biotechnology Information	2020-08-03	Quito, Ecuador	aspirate	Homo sapiens	Diseased	0.180653 S 78.467834 W	Eugenio Espejo Hospital	98.96	0.02	pass	K_pneumoniae_ASM24018v2	106	5381062	57.47	123337	pass	98.9483	pass	0
SAMEA111504323	ERR10437192	PRJEB56918	*Klebsiella pneumoniae*	Carlos Andrade Marín Hospital	National Center for Biotechnology Information	2020-08-11	Quito, Ecuador	aspirate	Homo sapiens	Diseased	0.180653 S 78.467834 W	Carlos Andrade Marín Hospital	98.91	0.8	pass	K_pneumoniae_ASM24018v2	129	5581198	57.25	134445	pass	99.6744	pass	0
SAMEA111504358	ERR10437352	PRJEB56918	*Klebsiella pneumoniae*	Eugenio Espejo Hospital	National Center for Biotechnology Information	2020-08-24	Quito, Ecuador	aspirate	Homo sapiens	Diseased	0.180653 S 78.467834 W	Eugenio Espejo Hospital	98.85	0.03	pass	K_pneumoniae_ASM24018v2	125	5362897	57.54	120360	pass	99.0198	pass	0
SAMEA111504357	ERR10437353	PRJEB56918	*Klebsiella pneumoniae*	Eugenio Espejo Hospital	National Center for Biotechnology Information	2020-08-24	Quito, Ecuador	aspirate	Homo sapiens	Diseased	0.180653 S 78.467834 W	Eugenio Espejo Hospital	99.04	0.14	pass	K_pneumoniae_ASM24018v2	105	5571393	57.22	99359	pass	99.0723	pass	0
SAMEA111504356	ERR10437351	PRJEB56918	*Klebsiella pneumoniae*	Eugenio Espejo Hospital	National Center for Biotechnology Information	2020-08-23	Quito, Ecuador	blood	Homo sapiens	Diseased	0.180653 S 78.467834 W	Eugenio Espejo Hospital	98.37	0.12	pass	K_pneumoniae_ASM24018v2	112	5497422	57.22	96302	pass	99.0676	pass	0
SAMEA111504353	ERR10437337	PRJEB56918	*Klebsiella pneumoniae*	Eugenio Espejo Hospital	National Center for Biotechnology Information	2020-08-21	Quito, Ecuador	blood	Homo sapiens	Diseased	0.180653 S 78.467834 W	Eugenio Espejo Hospital	96.24	0.16	pass	K_pneumoniae_ASM24018v2	93	5489807	57.19	162015	pass	99.0882	pass	0
SAMEA111504351	ERR10437323	PRJEB56918	*Klebsiella pneumoniae*	Carlos Andrade Marín Hospital	National Center for Biotechnology Information	2020-08-20	Quito, Ecuador	aspirate	Homo sapiens	Diseased	0.180653 S 78.467834 W	Carlos Andrade Marín Hospital	98.91	0.84	pass	K_pneumoniae_ASM24018v2	129	5581892	57.25	124817	pass	99.6286	pass	0
SAMEA111504350	ERR10437319	PRJEB56918	*Klebsiella pneumoniae*	Carlos Andrade Marín Hospital	National Center for Biotechnology Information	2020-08-20	Quito, Ecuador	swab (wound)	Homo sapiens	Diseased	0.180653 S 78.467834 W	Carlos Andrade Marín Hospital	98.91	0.08	pass	K_pneumoniae_ASM24018v2	106	5429039	57.42	145192	pass	99.6802	pass	0
SAMEA111504348	ERR10437314	PRJEB56918	*Klebsiella pneumoniae*	Riobamba Teaching Hospital	National Center for Biotechnology Information	2020-08-19	Riobamba, Ecuador	unspecified diagnostic sample	Homo sapiens	Diseased		Riobamba Teaching Hospital	99.02	0.12	pass	K_pneumoniae_ASM24018v2	141	5429880	57.35	102400	pass	99.0844	pass	0
SAMEA111504344	ERR10437324	PRJEB56918	*Klebsiella pneumoniae*	Carlos Andrade Marín Hospital	National Center for Biotechnology Information	2020-08-17	Quito, Ecuador	blood	Homo sapiens	Diseased	0.180653 S 78.467834 W	Carlos Andrade Marín Hospital	98.91	0.83	pass	K_pneumoniae_ASM24018v2	118	5584099	57.24	169580	pass	99.6327	pass	0
SAMEA111504349	ERR10437312	PRJEB56918	*Klebsiella pneumoniae*	Carlos Andrade Marín Hospital	National Center for Biotechnology Information	2020-08-20	Quito, Ecuador	aspirate	Homo sapiens	Diseased	0.180653 S 78.467834 W	Carlos Andrade Marín Hospital	98.91	0.85	pass	K_pneumoniae_ASM24018v2	152	5580149	57.25	102639	pass	99.6276	pass	0

ID	SRA Run	Bioproject	Organism	Recollected	Submitter	Collection date	Geo loc name	Isolation source	Host	Purpose of sampling	Lat ion	Hospital	Completeness_General	Contamination	1st Criterion	FastANI Reference	# contigs	Total length	GC (%)	N50	2nd Criterion	FastANI (%)	3rd Criterion	Failed criteria
SAMEA111504345	ERR10437302	PRJEB56918	*Klebsiella pneumoniae*	Riobamba Teaching Hospital	National Center for Biotechnology Information	2020-08-17	Riobamba, Ecuador	blood	Homo sapiens	Diseased		Riobamba Teaching Hospital	98.88	0.44	pass	K_pneumoniae_ASM24018v2	126	5640060	57.04	128831	pass	99.4604	pass	0
SAMEA111504346	ERR10437301	PRJEB56918	*Klebsiella pneumoniae*	Riobamba Teaching Hospital	National Center for Biotechnology Information	2020-08-18	Riobamba, Ecuador	blood	Homo sapiens	Diseased		Riobamba Teaching Hospital	98.88	0.47	pass	K_pneumoniae_ASM24018v2	127	5636243	57.04	111485	pass	99.4509	pass	0
SAMEA111504338	ERR10437276	PRJEB56918	*Klebsiella pneumoniae*	Carlos Andrade Marín Hospital	National Center for Biotechnology Information	2020-08-14	Quito, Ecuador	unspecified diagnostic sample	Homo sapiens	Diseased	0.180653 S 78.467834 W	Carlos Andrade Marín Hospital	98.91	0.08	pass	K_pneumoniae_ASM24018v2	105	5434475	57.42	145405	pass	99.7074	pass	0
SAMEA111504347	ERR10437299	PRJEB56918	*Klebsiella pneumoniae*	Teodoro Maldonado Carbo Hospital	National Center for Biotechnology Information	2020-08-18	Guayas, Guayaquil, Ecuador	sputum	Homo sapiens	Diseased	2.203816 S 79.897453 W	Teodoro Maldonado Carbo Hospital	98.98	0.46	pass	K_pneumoniae_ASM24018v2	173	5602674	57.08	118236	pass	99.006	pass	0
SAMEA111504341	ERR10437268	PRJEB56918	*Klebsiella pneumoniae*	Carlos Andrade Marín Hospital	National Center for Biotechnology Information	2020-08-17	Quito, Ecuador	aspirate	Homo sapiens	Diseased	0.180653 S 78.467834 W	Carlos Andrade Marín Hospital	98.91	0.83	pass	K_pneumoniae_ASM24018v2	144	5580989	57.24	124736	pass	99.6518	pass	0
SAMEA111504340	ERR10437266	PRJEB56918	*Klebsiella pneumoniae*	Eugenio Espejo Hospital	National Center for Biotechnology Information	2020-08-16	Quito, Ecuador	blood	Homo sapiens	Diseased	0.180653 S 78.467834 W	Eugenio Espejo Hospital	98.96	0.02	pass	K_pneumoniae_ASM24018v2	121	5376402	57.48	120290	pass	98.9867	pass	0
SAMEA111504336	ERR10437259	PRJEB56918	*Klebsiella pneumoniae*	Teodoro Maldonado Carbo Hospital	National Center for Biotechnology Information	2020-08-13	Guayas, Guayaquil, Ecuador	blood	Homo sapiens	Diseased	2.203816 S 79.897453 W	Teodoro Maldonado Carbo Hospital	98.98	0.13	pass	K_pneumoniae_ASM24018v2	163	5659217	57.02	94813	pass	99.5054	pass	0
SAMEA111504337	ERR10437258	PRJEB56918	*Klebsiella pneumoniae*	Carlos Andrade Marín Hospital	National Center for Biotechnology Information	2020-08-14	Quito, Ecuador	unspecified diagnostic sample	Homo sapiens	Diseased	0.180653 S 78.467834 W	Carlos Andrade Marín Hospital	98.99	0.08	pass	K_pneumoniae_ASM24018v2	124	5442721	57.41	124813	pass	99.698	pass	0
SAMEA111504339	ERR10437254	PRJEB56918	*Klebsiella pneumoniae*	Carlos Andrade Marín Hospital	National Center for Biotechnology Information	2020-08-16	Quito, Ecuador	aspirate	Homo sapiens	Diseased	0.180653 S 78.467834 W	Carlos Andrade Marín Hospital	98.99	0.8	pass	K_pneumoniae_ASM24018v2	150	5580185	57.25	95563	pass	99.6574	pass	0
SAMEA111504333	ERR10437245	PRJEB56918	*Klebsiella pneumoniae*	Teodoro Maldonado Carbo Hospital	National Center for Biotechnology Information	2020-08-13	Guayas, Guayaquil, Ecuador	feces	Homo sapiens	Diseased	2.203816 S 79.897453 W	Teodoro Maldonado Carbo Hospital	98.98	0.13	pass	K_pneumoniae_ASM24018v2	153	5659714	57	94180	pass	99.4653	pass	0
SAMEA111504335	ERR10437246	PRJEB56918	*Klebsiella pneumoniae*	Teodoro Maldonado Carbo Hospital	National Center for Biotechnology Information	2020-08-13	Guayas, Guayaquil, Ecuador	aspirate	Homo sapiens	Diseased	2.203816 S 79.897453 W	Teodoro Maldonado Carbo Hospital	98.98	0.15	pass	K_pneumoniae_ASM24018v2	159	5644874	57.01	86242	pass	99.5619	pass	0
SAMEA111504334	ERR10437243	PRJEB56918	*Klebsiella pneumoniae*	Teodoro Maldonado Carbo Hospital	National Center for Biotechnology Information	2020-08-13	Guayas, Guayaquil, Ecuador	aspirate	Homo sapiens	Diseased	2.203816 S 79.897453 W	Teodoro Maldonado Carbo Hospital	98.78	0.48	pass	K_pneumoniae_ASM24018v2	163	5757909	57.01	99262	pass	99.4863	pass	0
SAMEA111504327	ERR10437235	PRJEB56918	*Klebsiella pneumoniae*	Eugenio Espejo Hospital	National Center for Biotechnology Information	2020-08-13	Quito, Ecuador	blood	Homo sapiens	Diseased	0.180653 S 78.467834 W	Eugenio Espejo Hospital	98.93	0.02	pass	K_pneumoniae_ASM24018v2	103	5379058	57.47	145653	pass	99.0544	pass	0
SAMEA111504329	ERR10437223	PRJEB56918	*Klebsiella pneumoniae*	Teodoro Maldonado Carbo Hospital	National Center for Biotechnology Information	2020-08-13	Guayas, Guayaquil, Ecuador	aspirate	Homo sapiens	Diseased	2.203816 S 79.897453 W	Teodoro Maldonado Carbo Hospital	98.79	0.69	pass	K_pneumoniae_ASM24018v2	229	5629478	57.16	66108	pass	98.9378	pass	0
SAMEA111504325	ERR10437196	PRJEB56918	*Klebsiella pneumoniae*	Carlos Andrade Marín Hospital	National Center for Biotechnology Information	2020-08-13	Quito, Ecuador	urine	Homo sapiens	Diseased	0.180653 S 78.467834 W	Carlos Andrade Marín Hospital	98.92	0.09	pass	K_pneumoniae_ASM24018v2	139	5578965	57.2	92599	pass	99.4715	pass	0
SAMEA111504331	ERR10437231	PRJEB56918	*Klebsiella pneumoniae*	Teodoro Maldonado Carbo Hospital	National Center for Biotechnology Information	2020-08-13	Guayas, Guayaquil, Ecuador	urine	Homo sapiens	Diseased	2.203816 S 79.897453 W	Teodoro Maldonado Carbo Hospital	98.93	0.06	pass	K_pneumoniae_ASM24018v2	159	5565936	57.29	94087	pass	99.5923	pass	0
SAMEA111504326	ERR10437213	PRJEB56918	*Klebsiella pneumoniae*	Carlos Andrade Marín Hospital	National Center for Biotechnology Information	2020-08-13	Quito, Ecuador	urine	Homo sapiens	Diseased	0.180653 S 78.467834 W	Carlos Andrade Marín Hospital	98.92	0.05	pass	K_pneumoniae_ASM24018v2	115	5575239	57.21	149489	pass	99.5105	pass	0
SAMEA111504320	ERR10437181	PRJEB56918	*Klebsiella pneumoniae*	Eugenio Espejo Hospital	National Center for Biotechnology Information	2020-08-09	Quito, Ecuador	aspirate	Homo sapiens	Diseased	0.180653 S 78.467834 W	Eugenio Espejo Hospital	98.85	0.02	pass	K_pneumoniae_ASM24018v2	96	5375919	57.48	187217	pass	98.9996	pass	0
SAMEA111504321	ERR10437193	PRJEB56918	*Klebsiella pneumoniae*	Carlos Andrade Marín Hospital	National Center for Biotechnology Information	2020-08-11	Quito, Ecuador	aspirate	Homo sapiens	Diseased	0.180653 S 78.467834 W	Carlos Andrade Marín Hospital	98.91	0.83	pass	K_pneumoniae_ASM24018v2	128	5582652	57.24	134445	pass	99.6757	pass	0

ID	SRA Run	Bioproject	Organism	Recollected	Submmiter	Collection date	Geo loc name	Isolation source	Host	Purpose of sampling	Lat ion	Hospital	Completeness_General	Contamination	1st Criterion	FastANI Reference	# contigs	Total length	GC (%)	N50	2nd Criterion	FastANI (%)	3rd Criterion	Failed criteria
SAMEA111504330	ERR10437233	PRJEB56918	*Klebsiella pneumoniae*	Teodoro Maldonado Carbo Hospital	National Center for Biotechnology Information	2020-08-13	Guayas, Guayaquil, Ecuador	unspecified diagnostic sample	Homo sapiens	Diseased	2.203816 S 79.897453 W	Teodoro Maldonado Carbo Hospital	96.12	0.15	pass	K_pneumoniae_ASM24018v2	104	5364017	57.28	132425	pass	99.1035	pass	0
SAMEA111504318	ERR10437175	PRJEB56918	*Klebsiella pneumoniae*	Riobamba Teaching Hospital	National Center for Biotechnology Information	2020-08-07	Riobamba, Ecuador	blood	Homo sapiens	Diseased		Riobamba Teaching Hospital	98.88	0.47	pass	K_pneumoniae_ASM24018v2	124	5640650	57.04	122992	pass	99.4594	pass	0
SAMEA111504319	ERR10437168	PRJEB56918	*Klebsiella pneumoniae*	Instituto Nacional de Investigación en Salud Pública	National Center for Biotechnology Information	2020-08-09	Quito, Ecuador	unspecified diagnostic sample	Homo sapiens	Diseased	0.180653 S 78.467834 W	Instituto Nacional de Investigación en Salud Pública	98.91	0.73	pass	K_pneumoniae_ASM24018v2	157	5563900	57.25	79612	pass	99.666	pass	0
SAMEA111504314	ERR10437163	PRJEB56918	*Klebsiella pneumoniae*	Riobamba Teaching Hospital	National Center for Biotechnology Information	2020-08-06	Riobamba, Ecuador	blood	Homo sapiens	Diseased		Riobamba Teaching Hospital	98.93	0.18	pass	K_pneumoniae_ASM24018v2	125	5525134	57.29	122621	pass	98.9417	pass	0
SAMEA111504315	ERR10437160	PRJEB56918	*Klebsiella pneumoniae*	Carlos Andrade Marín Hospital	National Center for Biotechnology Information	2020-08-07	Quito, Ecuador	aspirate	Homo sapiens	Diseased	0.180653 S 78.467834 W	Carlos Andrade Marín Hospital	98.99	0.8	pass	K_pneumoniae_ASM24018v2	135	5580624	57.25	130081	pass	99.6661	pass	0
SAMEA111504316	ERR10437161	PRJEB56918	*Klebsiella pneumoniae*	Carlos Andrade Marín Hospital	National Center for Biotechnology Information	2020-08-07	Quito, Ecuador	aspirate	Homo sapiens	Diseased	0.180653 S 78.467834 W	Carlos Andrade Marín Hospital	98.91	0.8	pass	K_pneumoniae_ASM24018v2	142	5581859	57.24	110454	pass	99.6716	pass	0
SAMEA111504332	ERR10437234	PRJEB56918	*Klebsiella pneumoniae*	Teodoro Maldonado Carbo Hospital	National Center for Biotechnology Information	2020-08-13	Guayas, Guayaquil, Ecuador	blood	Homo sapiens	Diseased	2.203816 S 79.897453 W	Teodoro Maldonado Carbo Hospital	96.23	0.22	pass	K_pneumoniae_ASM24018v2	89	5445435	57.18	181273	pass	99.011	pass	0
SAMEA111504324	ERR10437201	PRJEB56918	*Klebsiella pneumoniae*	Carlos Andrade Marín Hospital	National Center for Biotechnology Information	2020-08-11	Quito, Ecuador	aspirate	Homo sapiens	Diseased	0.180653 S 78.467834 W	Carlos Andrade Marín Hospital	98.91	0.8	pass	K_pneumoniae_ASM24018v2	128	5581019	57.24	162737	pass	99.6504	pass	0
SAMN34232747	SRR25759565	PRJNA956653	*Klebsiella pneumoniae*	Hospital de niños Dr Roberto Gilbert	UEES	2020-10-14	Guayas, Guayaquil, Ecuador	urine	Homo sapiens	Diseased	2.176570 S 79.883580 W	Hospital de niños Dr Roberto Gilbert	93.07	1.83	pass	K_pneumoniae_ASM24018v2	955	5288092	57.64	8373	fail	99.0635	pass	1
SAMN34233589	SRR25759564	PRJNA956653	*Klebsiella pneumoniae*	Hospital de niños Dr Roberto Gilbert	UEES	2020-11-10	Guayas, Guayaquil, Ecuador	blood	Homo sapiens	Diseased	2.176570 S 79.883580 W	Hospital de niños Dr Roberto Gilbert	96.93	0.19	pass	K_pneumoniae_ASM24018v2	307	5464660	57.49	42246	pass	99.0733	pass	0
SAMN34233590	SRR25759561	PRJNA956653	*Klebsiella pneumoniae*	Hospital de niños Dr Roberto Gilbert	UEES	2020-11-10	Guayas, Guayaquil, Ecuador	blood	Homo sapiens	Diseased	2.176570 S 79.883580 W	Hospital de niños Dr Roberto Gilbert	99.04	0.11	pass	K_pneumoniae_ASM24018v2	68	5352490	57.45	242403	pass	99.0701	pass	0
SAMN34233591	SRR25759563	PRJNA956653	*Klebsiella pneumoniae*	Hospital de niños Dr Roberto Gilbert	UEES	2020-11-10	Guayas, Guayaquil, Ecuador	blood	Homo sapiens	Diseased	2.176570 S 79.883580 W	Hospital de niños Dr Roberto Gilbert	97.51	0.1	pass	K_pneumoniae_ASM24018v2	251	5234157	57.78	40064	pass	99.1152	pass	0
SAMN34233592	SRR25759569	PRJNA956653	*Klebsiella pneumoniae*	Hospital de niños Dr Roberto Gilbert	UEES	2021-10-14	Guayas, Guayaquil, Ecuador	blood	Homo sapiens	Diseased	2.176570 S 79.883580 W	Hospital de niños Dr Roberto Gilbert	96.82	0.48	pass	K_pneumoniae_ASM24018v2	554	5241550	57.52	17366	fail	98.9983	pass	1
SAMN34226817	SRR25759572	PRJNA956653	*Klebsiella pneumoniae*	Hospital de niños Dr Roberto Gilbert	UEES	2020-10-06	Guayas, Guayaquil, Ecuador	blood	Homo sapiens	Diseased	2.176570 S 79.883580 W	Hospital de niños Dr Roberto Gilbert	98.92	0.13	pass	K_pneumoniae_ASM24018v2	77	5300023	57.61	179530	pass	99.0794	pass	0
SAMN34233593	SRR25759568	PRJNA956653	*Klebsiella pneumoniae*	Hospital de niños Dr Roberto Gilbert	UEES	2020-11-14	Guayas, Guayaquil, Ecuador	blood	Homo sapiens	Diseased	2.176570 S 79.883580 W	Hospital de niños Dr Roberto Gilbert	96.17	0.52	pass	K_pneumoniae_ASM24018v2	463	5466568	57.53	23726	fail	99.0558	pass	1
SAMN34233594	SRR25759571	PRJNA956653	*Klebsiella pneumoniae*	Hospital de niños Dr Roberto Gilbert	UEES	2020-11-14	Guayas, Guayaquil, Ecuador	blood	Homo sapiens	Diseased	2.176570 S 79.883580 W	Hospital de niños Dr Roberto Gilbert	100	0.24	pass	K_pneumoniae_ASM24018v2	458	5442625	57.57	24112	fail	99.1431	pass	1
SAMN34226819	SRR24202601	PRJNA956653	*Klebsiella pneumoniae*	Hospital de niños Dr Roberto Gilbert	UEES	2020-10-06	Guayas, Guayaquil, Ecuador	blood	Homo sapiens	Diseased	2.176570 S 79.883580 W	Hospital de niños Dr Roberto Gilbert	99.04	0.1	pass	K_pneumoniae_ASM24018v2	68	5396520	57.23	198695	pass	99.0666	pass	0
SAMN34233596	SRR25759566	PRJNA956653	*Klebsiella pneumoniae*	Hospital de niños Dr Roberto Gilbert	UEES	2021-01-07	Guayas, Guayaquil, Ecuador	blood	Homo sapiens	Diseased	2.176570 S 79.883580 W	Hospital de niños Dr Roberto Gilbert	98.59	3.53	pass	K_pneumoniae_ASM24018v2	1100	5071605	58	6521	fail	99.0961	pass	1
SAMN34232744	SRR25759562	PRJNA956653	*Klebsiella pneumoniae*	Hospital de niños Dr Roberto Gilbert	UEES	2020-07-10	Guayas, Guayaquil, Ecuador	blood	Homo sapiens	Diseased	2.176570 S 79.883580 W	Hospital de niños Dr Roberto Gilbert	98.89	0.1	pass	K_pneumoniae_ASM24018v2	209	5291799	57.58	51782	pass	99.0592	pass	0

ID	SRA Run	Bioproject	Organism	Recollected	Submitter	Collection date	Geo loc name	Isolation source	Host	Purpose of sampling	Lat ion	Hospital	Completeness_General	Contamination	1st Criterion	FastANI Reference	# contigs	Total length	GC (%)	N50	2nd Criterion	FastANI (%)	3rd Criterion	Failed criteria
SAMN34232745	SRR25759570	PRJNA956653	*Klebsiella pneumoniae*	Hospital de niños Dr Roberto Gilbert	UEES	2020-07-10	Guayas, Guayaquil, Ecuador	blood	Homo sapiens	Diseased	2.176570 S 79.883580 W	Hospital de niños Dr Roberto Gilbert	99.04	0.1	pass	K_pneumoniae_ASM24018v2	63	5396772	57.23	261581	pass	99.0881	pass	0
SAMN34232746	SRR25759560	PRJNA956653	*Klebsiella pneumoniae*	Hospital de niños Dr Roberto Gilbert	UEES	2020-10-14	Guayas, Guayaquil, Ecuador	urine	Homo sapiens	Diseased	2.176570 S 79.883580 W	Hospital de niños Dr Roberto Gilbert	98.1	0.71	pass	K_pneumoniae_ASM24018v2	490	5105676	57.86	17951	fail	99.1285	pass	1
SAMN34233595	SRR25759567	PRJNA956653	*Klebsiella pneumoniae*	Hospital de niños Dr Roberto Gilbert	UEES	2022-01-11	Guayas, Guayaquil, Ecuador	blood	Homo sapiens	Diseased	2.176570 S 79.883580 W	Hospital de niños Dr Roberto Gilbert	76.49	2.06	fail	K_pneumoniae_ASM24018v2	1505	4156587	58.51	3250	fail	98.8602	pass	2
SAMEA111504312	ERR10437141	PRJEB56918	*Klebsiella oxytoca*	Carlos Andrade Marín Hospital	Centre for Genomic Epidemiology, National Foods INS	2020-08-15	Quito, Ecuador	swab (wound)	Homo sapiens	Diseased		Carlos Andrade Marin Hospital	#N/A	#N/A	#N/A	#N/A	#N/A	#N/A	#N/A	#N/A	#N/A	#N/A	#N/A	#N/A
SAMEA111504310	ERS13616672	PRJEB56918	*Klebsiella aerogenes*	Riobamba Teaching Hospital	Centre for Genomic Epidemiology, National Food Institute, Technical University of Denmark (DTU), Denmark	2020-08-17	Riobamba, Ecuador	blood	Homo sapiens	Diseased		Riobamba Teaching Hospital	97.12	0.14	pass	K_aerogenes_ASM1904812v1	53	5139631	55.08	163014	pass	95.9258	pass	0
SAMEA111504309	ERS13616671	PRJNA514245	*Klebsiella aerogenes*	Riobamba Teaching Hospital	Centre for Genomic Epidemiology, National Food Institute, Technical University of Denmark (DTU), Denmark	2020-08-17	Riobamba, Ecuador	blood	Homo sapiens	Diseased		Riobamba Teaching Hospital	98.96	0.41	pass	K_aerogenes_ASM1904812v1	71	5350540	55.03	172532	pass	95.9224	pass	0
SAMEA111504308	ERS13616670	PRJNA514245	*Klebsiella aerogenes*	Riobamba Teaching Hospital	Centre for Genomic Epidemiology, National Food Institute, Technical University of Denmark (DTU), Denmark	2020-08-11	Riobamba, Ecuador	blood	Homo sapiens	Diseased		Riobamba Teaching Hospital	98.94	0.62	pass	K_aerogenes_ASM1904812v1	62	5347257	55.03	228969	pass	95.9491	pass	0
SAMEA111504307	ERS13616669	PRJNA514245	*Klebsiella aerogenes*	Riobamba Teaching Hospital	Centre for Genomic Epidemiology, National Food Institute, Technical University of Denmark (DTU), Denmark	2020-08-06	Riobamba, Ecuador	blood	Homo sapiens	Diseased		Riobamba Teaching Hospital	98.38	1.27	pass	K_aerogenes_ASM1904812v1	66	5326258	54.98	258886	pass	95.914	pass	0
SAMEA111504363	ERS13616725	PRJNA514245	*Klebsiella variicola*	Eugenio Espejo Hospital	Centre for Genomic Epidemiology, National Food Institute, Technical University of Denmark (DTU), Denmark	2020-08-23	Quito, Ecuador	aspirate	Homo sapiens	Diseased	0.180653 S 78.467834 W	Eugenio Espejo Hospital	98.89	1.35	pass	K_variicola_ASM2052554v1	79	5640733	57.26	111272	pass	99.0815	pass	0

ID	SRA Run	Bioproject	Organism	Recollected	Submmiter	Collection date	Geo loc name	Isolation source	Host	Purpose of sampling	Lat ion	Hospital	Completeness_General	Contamination	1st Criterion	FastANI Reference	# contigs	Total length	GC (%)	N50	2nd Criterion	FastANI (%)	3rd Criterion	Failed criteria
SAMEA111504362	ERS13616724	PRJNA514245	*Klebsiella variicola*	Eugenio Espejo Hospital	Centre for Genomic Epidemiology, National Food Institute, Technical University of Denmark (DTU), Denmark	2020-08-12	Quito, Ecuador	aspirate	Homo sapiens	Diseased	0.180653 S 78.467834 W	Eugenio Espejo Hospital	96.2	0.4	pass	K_variicola_ASM2052554v1	72	5451872	57.45	125782	pass	99.0798	pass	0
SAMN29043418	SRR19650860	PRJNA849201	*Salmonella enterica*	Fernando Villavicencio. Viviana Alban	USFQ	2019	Azuay, Cuenca, Ecuador	wound secretion	Homo sapiens	Diseased	2.89741 S 79.0045 W		98.98	0.25	pass	Salmonella_ASM694v2	62	5011001	52.07	192562	pass	98.7471	pass	0
SAMN37705295	SRR26302937	PRJNA1024947	*Salmonella typhi*	National Institute of Public Health Research, Dr. Leopoldo Izquieta Perez	National Institute of Public Health Research INSPI-Ecuador	2023-06-01	Guayas, Guayaquil, Ecuador	blood	Homo sapiens	Diseased	2.17486570 S 79.94092966 W	Hospital del IEES ceibos	89.43	1.72	fail	Salmonella_ASM694v2	1319	3997068	51.98	3727	fail	97.8948	pass	2
SAMN37705294	SRR26302938	PRJNA1024947	*Salmonella typhi*	National Institute of Public Health Research, Dr. Leopoldo Izquieta Perez	National Institute of Public Health Research INSPI-Ecuador	2023-06-01	Guayas, Guayaquil, Ecuador	blood	Homo sapiens	Diseased	2.17486570 S 79.94092966 W	Hospital del IEES ceibos	97.19	0.12	pass	Salmonella_ASM694v2	106	4656855	52.09	70763	pass	98.2154	pass	0
SAMEA7302380	ERR4844965	PRJEB37560	*Salmonella enterica*	UNIETAR	FISABIO-Public Health Research	2018-07-13	Quito, Ecuador	feces	Homo sapiens	Diseased			98.81	0.05	pass	Salmonella_ASM694v2	67	4917289	52.13	149907	pass	98.7767	pass	0
SAMEA7302379	ERR4844964	PRJEB37560	*Salmonella enterica*	UNIETAR	FISABIO-Public Health Research	2018-08-24	Quito, Ecuador	feces	Homo sapiens	Diseased			100	0.04	pass	Salmonella_ASM694v2	69	5038611	51.98	183794	pass	98.6865	pass	0
SAMEA7302378	ERR4844963	PRJEB37560	*Salmonella enterica*	UNIETAR	FISABIO-Public Health Research	2018-09-17	Quito, Ecuador	feces	Homo sapiens	Diseased			98.81	0.08	pass	Salmonella_ASM694v2	60	4963066	52.16	201505	pass	98.7202	pass	0
SAMEA111504296	ERS13616658	PRJEB56918	*uncultured bacterium*	Carlos Andrade Marín Hospital	EBI	2020-08-10	Quito, Ecuador	blood	Homo sapiens	Diseased	0.180653 S 78.467834 W	Carlos Andrade Marín Hospital	98.91	0.83	pass	K_pneumoniae_ASM24018v2	132	5581412	57.25	133917	pass	99.6367	pass	0
SAMEA111504360	ERS13616722	PRJNA514245	*Serratia marcescens*	Eugenio Espejo Hospital	EBI	2020-08-04	Quito, Ecuador	unspecified diagnostic sample	Homo sapiens	Diseased	0.180653 S 78.467834 W	Eugenio Espejo Hospital	96.58	0.38	pass	Serratia_ASM3029173v1	91	5211596	59.77	137800	pass	95.2099	pass	0
SAMEA111504361	ERS13616723	PRJNA514245	*Serratia marcescens*	Eugenio Espejo Hospital	EBI	2020-08-16	Quito, Ecuador	blood	Homo sapiens	Diseased	0.180653 S 78.467834 W	Eugenio Espejo Hospital	97.23	0.36	pass	Serratia_ASM3029173v1	79	5259213	59.78	172497	pass	95.24	pass	0
SAMN09832923	SRR7693770	PRJNA486009	*Escherichia coli*	Instituto de Microbiologia, Universidad San Francisco de Quito	Georgia Institute of Technology	2015-06-13	Borbon, Ecuador	diarrhea	Homo sapiens	Diseased	1.08 N 78.99 W		100	0.11	pass	E_coli_ASM584v2	185	4876303	50.6	91273	pass	99.075	pass	0
SAMN09833074	SRR7693677	PRJNA514245	*Escherichia coli*	Instituto de Microbiologia, Universidad San Francisco de Quito	Georgia Institute of Technology	2014-09-03	Quito, Ecuador	feces	Homo sapiens	Diseased	0.18 S 78.46 W		99.81	0.03	pass	E_coli_ASM584v2	206	5121482	50.69	74226	pass	99.4548	pass	0
SAMN09832987	SRR7693791	PRJNA486009	*Escherichia coli*	Instituto de Microbiologia, Universidad San Francisco de Quito	Georgia Institute of Technology	2015-08-02	Ecuador	diarrhea	Homo sapiens	Diseased	1.8312 S 78.1834 W		100	0.42	pass	E_coli_ASM584v2	152	5024272	50.47	82882	pass	98.3519	pass	0
SAMN09833072	SRR7693675	PRJNA514245	*Escherichia coli*	Instituto de Microbiologia, Universidad San Francisco de Quito	Georgia Institute of Technology	2014-09-03	Quito, Ecuador	feces	Homo sapiens	Diseased	0.18 S 78.46 W		99.89	0.84	pass	E_coli_ASM584v2	106	5043293	50.53	168357	pass	96.6796	pass	0
SAMN09833117	SRR7693617	PRJNA486009	*Escherichia coli*	Instituto de Microbiologia, Universidad San Francisco de Quito	Georgia Institute of Technology	2014-09-01	Quito, Ecuador	feces	Homo sapiens	Diseased	0.18 S 78.46 W		100	0.03	pass	E_coli_ASM584v2	396	4910529	50.7	28101	fail	99.1686	pass	1

ID	SRA Run	Bioproject	Organism	Recollected	Submmiter	Collection date	Geo loc name	Isolation source	Host	Purpose of sampling	Lat ion	Hospital	Completeness_General	Contamination	1st Criterion	FastANI Reference	# contigs	Total length	GC (%)	N50	2nd Criterion	FastANI (%)	3rd Criterion	Failed criteria
SAMN09833144	SRR7693646	PRJNA514245	*Escherichia coli*	Instituto de Microbiologia, Universidad San Francisco de Quito	Georgia Institute of Technology	2015-04-20	Selva Alegre, Ecuador	feces	Homo sapiens	Diseased	0.33 N 78.19 W		100	0.19	pass	E_coli_ASM584v2	165	4909978	50.53	98205	pass	99.1142	pass	0
SAMN09832911	SRR7693797	PRJNA486009	*Escherichia coli*	Instituto de Microbiologia, Universidad San Francisco de Quito	Georgia Institute of Technology	2015-04-13	Las Peñas, Ecuador	diarrhea	Homo sapiens	Diseased	1.8312 S 78.1834 W		100	0.3	pass	E_coli_ASM584v2	175	4976878	50.56	112075	pass	99.3454	pass	0
SAMN17228541	SRR13370409	PRJNA514245	*Escherichia coli*	Instituto de Microbiologia, Universidad San Francisco de Quito	Georgia Institute of Technology	2014-04-28	Esmeraldas, Ecuador	feces	Homo sapiens	Diseased	0.96 N 79.65 W	centro forence de esmeraldas	100	0.86	pass	E_coli_ASM584v2	231	5253081	50.61	87633	pass	97.3788	pass	0
SAMN09833122	SRR7693851	PRJNA486009	*Escherichia coli*	Instituto de Microbiologia, Universidad San Francisco de Quito	Georgia Institute of Technology	2014-09-02	Quito, Ecuador	feces	Homo sapiens	Diseased	0.18 S 78.46 W		99.49	0.22	pass	E_coli_ASM584v2	256	4641574	50.79	39282	fail	98.3344	pass	1
SAMN09833102	SRR7693606	PRJNA514245	*Escherichia coli*	Instituto de Microbiologia, Universidad San Francisco de Quito	Georgia Institute of Technology	2014-09-23	Quito, Ecuador	feces	Homo sapiens	Diseased	0.18 S 78.46 W		100	0.18	pass	E_coli_ASM584v2	145	4912461	50.69	97695	pass	98.8445	pass	0
SAMN09832922	SRR7693771	PRJNA486009	*Escherichia coli*	Instituto de Microbiologia, Universidad San Francisco de Quito	Georgia Institute of Technology	2014-06-02	Borbon, Ecuador	diarrhea	Homo sapiens	Diseased	1.08 N 78.99 W		100	0.19	pass	E_coli_ASM584v2	169	4664361	50.89	73441	pass	99.2445	pass	0
SAMN09832913	SRR7693799	PRJNA486009	*Escherichia coli*	Instituto de Microbiologia, Universidad San Francisco de Quito	Georgia Institute of Technology	2015-05-04	Borbon, Ecuador	diarrhea	Homo sapiens	Diseased	1.08 N 78.99 W		100	0.17	pass	E_coli_ASM584v2	149	4971793	50.73	115877	pass	99.5378	pass	0
SAMN09832908	SRR7693837	PRJNA486009	*Escherichia coli*	Instituto de Microbiologia, Universidad San Francisco de Quito	Georgia Institute of Technology	2015-04-11	Borbon, Ecuador	diarrhea	Homo sapiens	Diseased	1.08 N 78.99 W		100	0.09	pass	E_coli_ASM584v2	128	4825616	50.65	97148	pass	98.3928	pass	0
SAMN09833021	SRR7693839	PRJNA486009	*Escherichia coli*	Instituto de Microbiologia, Universidad San Francisco de Quito	Georgia Institute of Technology	2014-07-22	Esmeraldas, Ecuador	diarrhea	Homo sapiens	Diseased	0.96 N 79.65 W		100	0.66	pass	E_coli_ASM584v2	189	5081927	50.57	98429	pass	96.6537	pass	0
SAMN09833099	SRR7693753	PRJNA514245	*Escherichia coli*	Instituto de Microbiologia, Universidad San Francisco de Quito	Georgia Institute of Technology	2014-09-22	Quito, Ecuador	feces	Homo sapiens	Diseased	0.18 S 78.46 W		97.76	0.69	pass	E_coli_ASM584v2	357	5180493	50.67	44796	pass	96.74	pass	0
SAMN09833001	SRR7693784	PRJNA486009	*Escherichia coli*	Instituto de Microbiologia, Universidad San Francisco de Quito	Georgia Institute of Technology	2014-06-24	Esmeraldas, Ecuador	diarrhea	Homo sapiens	Diseased	0.96 N 79.65 W		100	0.63	pass	E_coli_ASM584v2	203	5288630	50.41	93281	pass	99.4906	pass	0
SAMN09832929	SRR7693767	PRJNA486009	*Escherichia coli*	Instituto de Microbiologia, Universidad San Francisco de Quito	Georgia Institute of Technology	2015-07-05	Borbon, Ecuador	diarrhea	Homo sapiens	Diseased	1.08 N 78.99 W		100	0.3	pass	E_coli_ASM584v2	194	4952258	50.57	63817	pass	99.1921	pass	0
SAMN09833132	SRR7693662	PRJNA486009	*Escherichia coli*	Instituto de Microbiologia, Universidad San Francisco de Quito	Georgia Institute of Technology	2015-09-20	Ecuador	feces	Homo sapiens	Diseased	1.83 S 78.18 W		99.89	0.92	pass	E_coli_ASM584v2	209	4834855	50.48	70360	pass	96.6962	pass	0

ID	SRA Run	Bioproject	Organism	Recollected	Submmiter	Collection date	Geo loc name	Isolation source	Host	Purpose of sampling	Lat ion	Hospital	Completeness_General	Contamination	1st Criterion	FastANI Reference	# contigs	Total length	GC (%)	N50	2nd Criterion	FastANI (%)	3rd Criterion	Failed criteria
SAMN09832910	SRR7693796	PRJNA486009	*Escherichia coli*	Instituto de Microbiologia, Universidad San Francisco de Quito	Georgia Institute of Technology	2015-04-13	Las Peñas, Ecuador	diarrhea	Homo sapiens	Diseased	1.08 N 79.15 W		100	0.35	pass	E_coli_ASM584v2	144	5058636	50.47	179574	pass	99.4052	pass	0
SAMN09832928	SRR7693768	PRJNA486009	*Escherichia coli*	Instituto de Microbiologia, Universidad San Francisco de Quito	Georgia Institute of Technology	2015-07-03	Borbon, Ecuador	diarrhea	Homo sapiens	Diseased	1.08 N 78.99 W		100	0.37	pass	E_coli_ASM584v2	449	4730443	50.99	24323	fail	98.3698	pass	1
SAMN09833114	SRR7693616	PRJNA486009	*Escherichia coli*	Instituto de Microbiologia, Universidad San Francisco de Quito	Georgia Institute of Technology	2014-08-26	Quito, Ecuador	feces	Homo sapiens	Diseased	0.18 S 78.46 W		62.44	22.16	fail	E_coli_ASM584v2	2967	4608342	49.97	2361	fail	95.2578	pass	2
SAMN09832898	SRR7693765	PRJNA486009	*Escherichia coli*	Instituto de Microbiologia, Universidad San Francisco de Quito	Georgia Institute of Technology	2014-05-16	Borbon, Ecuador	diarrhea	Homo sapiens	Diseased	1.08 N 78.99 W		100	0.49	pass	E_coli_ASM584v2	76	4899687	50.75	197206	pass	96.7469	pass	0
SAMN09833075	SRR7693678	PRJNA514245	*Escherichia coli*	Instituto de Microbiologia, Universidad San Francisco de Quito	Georgia Institute of Technology	2014-05-09	Quito, Ecuador	feces	Homo sapiens	Diseased	0.18 S 78.46 W		100	0.04	pass	E_coli_ASM584v2	229	5155890	50.66	55449	pass	98.3435	pass	0
SAMN09832917	SRR7693795	PRJNA486009	*Escherichia coli*	Instituto de Microbiologia, Universidad San Francisco de Quito	Georgia Institute of Technology	2015-05-22	Borbon, Ecuador	diarrhea	Homo sapiens	Diseased	1.08 N 78.99 W		98.64	0.14	pass	E_coli_ASM584v2	103	5037911	50.48	158474	pass	97.2662	pass	0
SAMN09833059	SRR7693664	PRJNA514245	*Escherichia coli*	Instituto de Microbiologia, Universidad San Francisco de Quito	Georgia Institute of Technology	2014-06-07	Esmeraldas, Ecuador	feces	Homo sapiens	Diseased	0.96 N 79.65 W		100	0.34	pass	E_coli_ASM584v2	220	5323542	50.58	60311	pass	97.1704	pass	0
SAMN09833082	SRR7693719	PRJNA514245	*Escherichia coli*	Instituto de Microbiologia, Universidad San Francisco de Quito	Georgia Institute of Technology	2014-05-12	Quito, Ecuador	feces	Homo sapiens	Diseased	0.18 S 78.46 W		99.61	0.18	pass	E_coli_ASM584v2	332	4673269	51.05	32538	fail	98.3787	pass	1
SAMN09833050	SRR7693671	PRJNA486009	*Escherichia coli*	Instituto de Microbiologia, Universidad San Francisco de Quito	Georgia Institute of Technology	2014-04-23	Esmeraldas, Ecuador	feces	Homo sapiens	Diseased	0.96 N 79.65 W		97.9	0.09	pass	E_coli_ASM584v2	229	5232367	50.42	112572	pass	97.8555	pass	0
SAMN09833107	SRR7693709	PRJNA514245	*Escherichia coli*	Instituto de Microbiologia, Universidad San Francisco de Quito	Georgia Institute of Technology	2014-05-19	Quito, Ecuador	feces	Homo sapiens	Diseased	0.18 S 78.46 W		100	0.01	pass	E_coli_ASM584v2	378	4440643	50.87	25437	fail	98.2444	pass	1
SAMN09832925	SRR7693776	PRJNA486009	*Escherichia coli*	Instituto de Microbiologia, Universidad San Francisco de Quito	Georgia Institute of Technology	2015-06-20	Borbon, Ecuador	diarrhea	Homo sapiens	Diseased	1.08 N 78.99 W		100	0.02	pass	E_coli_ASM584v2	119	4892088	50.61	119226	pass	99.406	pass	0
SAMN09833151	SRR7693722	PRJNA514245	*Escherichia coli*	Instituto de Microbiologia, Universidad San Francisco de Quito	Georgia Institute of Technology	2015-06-23	Santo domingo, Ecuador	feces	Homo sapiens	Diseased	0.25 S 79.17 W		99.97	0.86	pass	E_coli_ASM584v2	114	5197692	50.62	200224	pass	96.7895	pass	0
SAMN09833090	SRR7693747	PRJNA514245	*Escherichia coli*	Instituto de Microbiologia, Universidad San Francisco de Quito	Georgia Institute of Technology	2014-09-18	Quito, Ecuador	feces	Homo sapiens	Diseased	0.18 S 78.46 W		100	0.2	pass	E_coli_ASM584v2	193	5021502	50.43	86305	pass	98.4048	pass	0

ID	SRA Run	Bioproject	Organism	Recollected	Submmiter	Collection date	Geo loc name	Isolation source	Host	Purpose of sampling	Lat ion	Hospital	Completeness_General	Contamination	1st Criterion	FastANI Reference	# contigs	Total length	GC (%)	N50	2nd Criterion	FastANI (%)	3rd Criterion	Failed criteria
SAMN09833110	SRR7693620	PRJNA486009	*Escherichia coli*	Instituto de Microbiologia, Universidad San Francisco de Quito	Georgia Institute of Technology	2014-05-19	Quito, Ecuador	feces	Homo sapiens	Diseased	0.18 S 78.46 W		99.76	0.25	pass	E_coli_ASM584v2	760	4971845	50.56	12756	fail	99.0709	pass	1
SAMN09833093	SRR7693744	PRJNA514245	*Escherichia coli*	Instituto de Microbiologia, Universidad San Francisco de Quito	Georgia Institute of Technology	2014-09-18	Quito, Ecuador	feces	Homo sapiens	Diseased	0.18 S 78.46 W		100	0.51	pass	E_coli_ASM584v2	418	4800193	50.79	23653	fail	98.4688	pass	1
SAMN09833126	SRR7693826	PRJNA486009	*Escherichia coli*	Instituto de Microbiologia, Universidad San Francisco de Quito	Georgia Institute of Technology	2015-08-15	Ecuador	feces	Homo sapiens	Diseased	1.83 S 78.18 W		100	0.11	pass	E_coli_ASM584v2	153	5202963	50.42	150111	pass	97.3078	pass	0
SAMN09833121	SRR7693854	PRJNA486009	*Escherichia coli*	Instituto de Microbiologia, Universidad San Francisco de Quito	Georgia Institute of Technology	2014-09-02	Quito, Ecuador	feces	Homo sapiens	Diseased	0.18 S 78.46 W		100	0.09	pass	E_coli_ASM584v2	238	4807964	50.64	56118	pass	99.1209	pass	0
SAMN09833047	SRR7693596	PRJNA486009	*Escherichia coli*	Instituto de Microbiologia, Universidad San Francisco de Quito	Georgia Institute of Technology	2014-05-28	Esmeraldas, Ecuador	feces	Homo sapiens	Diseased	0.96 N 79.65 W		100	0.62	pass	E_coli_ASM584v2	279	5133321	50.71	65307	pass	97.4109	pass	0
SAMN09833003	SRR7693782	PRJNA486009	*Escherichia coli*	Instituto de Microbiologia, Universidad San Francisco de Quito	Georgia Institute of Technology	2014-06-24	Esmeraldas, Ecuador	diarrhea	Homo sapiens	Diseased	0.96 N 79.65 W		100	0.14	pass	E_coli_ASM584v2	96	5028768	50.34	147845	pass	97.1012	pass	0
SAMN09833018	SRR7693811	PRJNA486009	*Escherichia coli*	Instituto de Microbiologia, Universidad San Francisco de Quito	Georgia Institute of Technology	2014-07-18	Esmeraldas, Ecuador	diarrhea	Homo sapiens	Diseased	0.96 N 79.65 W		99.18	0.56	pass	E_coli_ASM584v2	175	5060198	50.87	73267	pass	96.7281	pass	0
SAMN09833113	SRR7693621	PRJNA486009	*Escherichia coli*	Instituto de Microbiologia, Universidad San Francisco de Quito	Georgia Institute of Technology	2014-08-26	Quito, Ecuador	feces	Homo sapiens	Diseased	0.18 S 78.46 W		95.82	1.21	pass	E_coli_ASM584v2	161	5203269	50.55	105936	pass	96.7893	pass	0
SAMN09833084	SRR7693717	PRJNA514245	*Escherichia coli*	Instituto de Microbiologia, Universidad San Francisco de Quito	Georgia Institute of Technology	2014-09-17	Quito, Ecuador	feces	Homo sapiens	Diseased	0.18 S 78.46 W		100	0.04	pass	E_coli_ASM584v2	220	5171126	50.63	113713	pass	99.435	pass	0
SAMN09833148	SRR7693634	PRJNA514245	*Escherichia coli*	Instituto de Microbiologia, Universidad San Francisco de Quito	Georgia Institute of Technology	2015-06-18	Santo domingo, Ecuador	feces	Homo sapiens	Diseased	0.25 S 79.17 W		100	0.12	pass	E_coli_ASM584v2	235	4870496	50.64	55599	pass	99.3596	pass	0
SAMN09833145	SRR7693647	PRJNA514245	*Escherichia coli*	Instituto de Microbiologia, Universidad San Francisco de Quito	Georgia Institute of Technology	2015-04-20	Selva Alegre, Ecuador	feces	Homo sapiens	Diseased	0.33 N 78.19 W		100	0.19	pass	E_coli_ASM584v2	164	4844699	50.62	73375	pass	99.0914	pass	0
SAMN09833081	SRR7693720	PRJNA514245	*Escherichia coli*	Instituto de Microbiologia, Universidad San Francisco de Quito	Georgia Institute of Technology	2014-09-16	Quito, Ecuador	feces	Homo sapiens	Diseased	0.18 S 78.46 W		99.89	0.83	pass	E_coli_ASM584v2	187	4993833	50.68	88814	pass	96.7315	pass	0
SAMN09833005	SRR7693780	PRJNA486009	*Escherichia coli*	Instituto de Microbiologia, Universidad San Francisco de Quito	Georgia Institute of Technology	2014-04-28	Esmeraldas, Ecuador	diarrhea	Homo sapiens	Diseased	0.96 N 79.65 W		100	1.05	pass	E_coli_ASM584v2	144	4986947	50.84	104498	pass	96.754	pass	0

ID	SRA Run	Bioproject	Organism	Recollected	Submmiter	Collection date	Geo loc name	Isolation source	Host	Purpose of sampling	Lat ion	Hospital	Completeness_General	Contamination	1st Criterion	FastANI Reference	# contigs	Total length	GC (%)	N50	2nd Criterion	FastANI (%)	3rd Criterion	Failed criteria
SAMN09833136	SRR7693658	PRJNA514245	*Escherichia coli*	Instituto de Microbiologia, Universidad San Francisco de Quito	Georgia Institute of Technology	2014-11-12	Ecuador	feces	Homo sapiens	Diseased	1.83 S 78.18 W		99.97	0.57	pass	E_coli_ASM584v2	157	5268031	50.47	115788	pass	99.4612	pass	0
SAMN09833131	SRR7693624	PRJNA486009	*Escherichia coli*	Instituto de Microbiologia, Universidad San Francisco de Quito	Georgia Institute of Technology	2015-08-18	Ecuador	feces	Homo sapiens	Diseased	1.83 S 78.18 W		98.78	0.03	pass	E_coli_ASM584v2	119	4770629	50.9	109406	pass	98.8988	pass	0
SAMN09833009	SRR7693787	PRJNA486009	*Escherichia coli*	Instituto de Microbiologia, Universidad San Francisco de Quito	Georgia Institute of Technology	2014-07-10	Esmeraldas, Ecuador	diarrhea	Homo sapiens	Diseased	0.96 N 79.65 W		100	0.53	pass	E_coli_ASM584v2	139	5250333	50.65	155123	pass	97.221	pass	0
SAMN09833063	SRR7693704	PRJNA514245	*Escherichia coli*	Instituto de Microbiologia, Universidad San Francisco de Quito	Georgia Institute of Technology	2014-06-12	Esmeraldas, Ecuador	feces	Homo sapiens	Diseased	0.96 N 79.65 W		99.49	0.44	pass	E_coli_ASM584v2	226	5045599	50.72	81173	pass	99.1314	pass	0
SAMN09833022	SRR7693842	PRJNA486009	*Escherichia coli*	Instituto de Microbiologia, Universidad San Francisco de Quito	Georgia Institute of Technology	2014-07-22	Esmeraldas, Ecuador	feces	Homo sapiens	Diseased	0.96 N 79.65 W		100	0.2	pass	E_coli_ASM584v2	158	5333994	50.29	92362	pass	97.1147	pass	0
SAMN09833135	SRR7693659	PRJNA514245	*Escherichia coli*	Instituto de Microbiologia, Universidad San Francisco de Quito	Georgia Institute of Technology	2014-10-15	Ecuador	feces	Homo sapiens	Diseased	1.83 S 78.18 W		100	0.16	pass	E_coli_ASM584v2	180	4821410	50.69	72124	pass	99.1435	pass	0
SAMN09833106	SRR7693708	PRJNA514245	*Escherichia coli*	Instituto de Microbiologia, Universidad San Francisco de Quito	Georgia Institute of Technology	2014-05-18	Quito, Ecuador	feces	Homo sapiens	Diseased	0.18 S 78.46 W		100	0	pass	E_coli_ASM584v2	388	4402445	50.87	23885	fail	98.3496	pass	1
SAMN09833053	SRR7693670	PRJNA514245	*Escherichia coli*	Instituto de Microbiologia, Universidad San Francisco de Quito	Georgia Institute of Technology	2014-06-03	Esmeraldas, Ecuador	feces	Homo sapiens	Diseased	0.96 N 79.65 W		100	0.17	pass	E_coli_ASM584v2	134	5046897	50.67	151584	pass	98.5795	pass	0
SAMN09833024	SRR7693844	PRJNA486009	*Escherichia coli*	Instituto de Microbiologia, Universidad San Francisco de Quito	Georgia Institute of Technology	2014-07-24	Esmeraldas, Ecuador	feces	Homo sapiens	Diseased	0.96 N 79.65 W		100	0.38	pass	E_coli_ASM584v2	170	4913515	50.51	64575	pass	98.2805	pass	0
SAMN09832989	SRR7693790	PRJNA486009	*Escherichia coli*	Instituto de Microbiologia, Universidad San Francisco de Quito	Georgia Institute of Technology	2015-08-02	Ecuador	diarrhea	Homo sapiens	Diseased	1.8312 S 78.1834 W		100	0.04	pass	E_coli_ASM584v2	177	4779481	50.85	64169	pass	99.1976	pass	0
SAMN09833069	SRR7693696	PRJNA514245	*Escherichia coli*	Instituto de Microbiologia, Universidad San Francisco de Quito	Georgia Institute of Technology	2014-09-08	Quito, Ecuador	feces	Homo sapiens	Diseased	0.18 S 78.46 W		100	0.42	pass	E_coli_ASM584v2	370	4823848	50.72	29636	fail	98.3907	pass	1
SAMN09832924	SRR7693777	PRJNA486009	*Escherichia coli*	Instituto de Microbiologia, Universidad San Francisco de Quito	Georgia Institute of Technology	2015-06-17	Borbon, Ecuador	diarrhea	Homo sapiens	Diseased	1.08 N 78.99 W		100	0.07	pass	E_coli_ASM584v2	153	5040647	50.44	92187	pass	99.4608	pass	0
SAMN09833065	SRR7693698	PRJNA514245	*Escherichia coli*	Instituto de Microbiologia, Universidad San Francisco de Quito	Georgia Institute of Technology	2014-06-12	Esmeraldas, Ecuador	feces	Homo sapiens	Diseased	0.96 N 79.65 W		100	0.09	pass	E_coli_ASM584v2	206	4777830	50.58	78281	pass	99.0958	pass	0

ID	SRA Run	Bioproject	Organism	Recollected	Submmiter	Collection date	Geo loc name	Isolation source	Host	Purpose of sampling	Lat ion	Hospital	Completeness_General	Contamination	1st Criterion	FastANI Reference	# contigs	Total length	GC (%)	N50	2nd Criterion	FastANI (%)	3rd Criterion	Failed criteria
SAMN09833077	SRR7693680	PRJNA514245	*Escherichia coli*	Instituto de Microbiologia, Universidad San Francisco de Quito	Georgia Institute of Technology	2014-10-11	Quito, Ecuador	feces	Homo sapiens	Diseased	0.18 S 78.46 W		100	0.19	pass	E_coli_ASM584v2	192	5250742	50.42	77107	pass	97.1468	pass	0
SAMN09833055	SRR7693668	PRJNA486009	*Escherichia coli*	Instituto de Microbiologia, Universidad San Francisco de Quito	Georgia Institute of Technology	2014-06-04	Esmeraldas, Ecuador	feces	Homo sapiens	Diseased	0.96 N 79.65 W		97.53	0.22	pass	E_coli_ASM584v2	345	5030395	50.81	28741	fail	99.4684	pass	1
SAMN09832916	SRR7693794	PRJNA486009	*Escherichia coli*	Instituto de Microbiologia, Universidad San Francisco de Quito	Georgia Institute of Technology	2015-05-21	Borbon, Ecuador	diarrhea	Homo sapiens	Diseased	1.08 N 78.99 W		100	0.37	pass	E_coli_ASM584v2	155	5170483	50.63	92292	pass	97.1734	pass	0
SAMN09833057	SRR7693666	PRJNA486009	*Escherichia coli*	Instituto de Microbiologia, Universidad San Francisco de Quito	Georgia Institute of Technology	2014-06-05	Esmeraldas, Ecuador	feces	Homo sapiens	Diseased	0.96 N 79.65 W		100	0	pass	E_coli_ASM584v2	418	4532607	50.88	23246	fail	98.3274	pass	1
SAMN09833025	SRR7693843	PRJNA486009	*Escherichia coli*	Instituto de Microbiologia, Universidad San Francisco de Quito	Georgia Institute of Technology	2014-07-24	Esmeraldas, Ecuador	feces	Homo sapiens	Diseased	0.96 N 79.65 W		100	0.11	pass	E_coli_ASM584v2	103	4869521	50.51	189652	pass	96.7698	pass	0
SAMN09832900	SRR7693829	PRJNA486009	*Escherichia coli*	Instituto de Microbiologia, Universidad San Francisco de Quito	Georgia Institute of Technology	2015-01-11	Borbon, Ecuador	diarrhea	Homo sapiens	Diseased	1.08 N 78.99 W		100	0.08	pass	E_coli_ASM584v2	134	5033204	50.55	112512	pass	98.4102	pass	0
SAMN09833064	SRR7693699	PRJNA514245	*Escherichia coli*	Instituto de Microbiologia, Universidad San Francisco de Quito	Georgia Institute of Technology	2014-06-12	Esmeraldas, Ecuador	feces	Homo sapiens	Diseased	0.96 N 79.65 W		100	0.08	pass	E_coli_ASM584v2	287	4779359	50.58	40367	pass	99.1398	pass	0
SAMN09833100	SRR7693710	PRJNA514245	*Escherichia coli*	Instituto de Microbiologia, Universidad San Francisco de Quito	Georgia Institute of Technology	2014-09-23	Quito, Ecuador	feces	Homo sapiens	Diseased	0.18 S 78.46 W		100	0.05	pass	E_coli_ASM584v2	73	4561878	50.59	180548	pass	99.4513	pass	0
SAMN09833139	SRR7693642	PRJNA514245	*Escherichia coli*	Instituto de Microbiologia, Universidad San Francisco de Quito	Georgia Institute of Technology	2014-12-14	Selva Alegre, Ecuador	feces	Homo sapiens	Diseased	0.33 N 78.19 W		100	0	pass	E_coli_ASM584v2	93	4935704	50.72	150678	pass	99.0674	pass	0
SAMN09833029	SRR7693847	PRJNA486009	*Escherichia coli*	Instituto de Microbiologia, Universidad San Francisco de Quito	Georgia Institute of Technology	2014-08-04	Esmeraldas, Ecuador	feces	Homo sapiens	Diseased	0.96 N 79.65 W		100	19.33	fail	E_coli_ASM584v2	476	6409330	51.21	55295	pass	97.1429	pass	1
SAMN09832926	SRR7693775	PRJNA486009	*Escherichia coli*	Instituto de Microbiologia, Universidad San Francisco de Quito	Georgia Institute of Technology	2015-06-24	Borbon, Ecuador	diarrhea	Homo sapiens	Diseased	1.08 N 78.99 W		100	0.06	pass	E_coli_ASM584v2	210	4739285	50.74	56357	pass	99.1279	pass	0
SAMN09833116	SRR7693618	PRJNA486009	*Escherichia coli*	Instituto de Microbiologia, Universidad San Francisco de Quito	Georgia Institute of Technology	2014-08-28	Quito, Ecuador	feces	Homo sapiens	Diseased	0.18 S 78.46 W		85.67	6.83	fail	E_coli_ASM584v2	2308	4453527	49.84	2869	fail	97.8007	pass	2
SAMN09833002	SRR7693783	PRJNA486009	*Escherichia coli*	Instituto de Microbiologia, Universidad San Francisco de Quito	Georgia Institute of Technology	2014-06-24	Esmeraldas, Ecuador	diarrhea	Homo sapiens	Diseased	0.96 N 79.65 W		100	0.44	pass	E_coli_ASM584v2	221	5265935	50.42	82442	pass	99.4859	pass	0

ID	SRA Run	Bioproject	Organism	Recollected	Submitter	Collection date	Geo loc name	Isolation source	Host	Purpose of sampling	Lat ion	Hospital	Completeness_General	Contamination	1st Criterion	FastANI Reference	# contigs	Total length	GC (%)	N50	2nd Criterion	FastANI (%)	3rd Criterion	Failed criteria
SAMN09833006	SRR7693779	PRJNA486009	*Escherichia coli*	Instituto de Microbiologia, Universidad San Francisco de Quito	Georgia Institute of Technology	2014-06-27	Esmeraldas, Ecuador	diarrhea	Homo sapiens	Diseased	0.96 N 79.65 W		100	0.58	pass	E_coli_ASM584v2	176	5144673	50.6	149125	pass	97.3287	pass	0
SAMN09832912	SRR7693798	PRJNA486009	*Escherichia coli*	Instituto de Microbiologia, Universidad San Francisco de Quito	Georgia Institute of Technology	2015-04-17	Ecuador	diarrhea	Homo sapiens	Diseased	1.8312 S 78.1834 W		100	0.04	pass	E_coli_ASM584v2	145	4984037	50.5	150355	pass	98.429	pass	0
SAMN09833115	SRR7693615	PRJNA486009	*Escherichia coli*	Instituto de Microbiologia, Universidad San Francisco de Quito	Georgia Institute of Technology	2014-08-27	Quito, Ecuador	feces	Homo sapiens	Diseased	0.18 S 78.46 W		100	0.61	pass	E_coli_ASM584v2	88	5091048	50.67	223802	pass	96.8183	pass	0
SAMN09833070	SRR7693673	PRJNA514245	*Escherichia coli*	Instituto de Microbiologia, Universidad San Francisco de Quito	Georgia Institute of Technology	2014-09-08	Quito, Ecuador	feces	Homo sapiens	Diseased	0.18 S 78.46 W		100	0.5	pass	E_coli_ASM584v2	405	4913585	50.68	28746	fail	98.3881	pass	1
SAMN09833111	SRR7693619	PRJNA486009	*Escherichia coli*	Instituto de Microbiologia, Universidad San Francisco de Quito	Georgia Institute of Technology	2014-08-06	Quito, Ecuador	feces	Homo sapiens	Diseased	0.18 S 78.46 W		100	0.02	pass	E_coli_ASM584v2	173	5056043	50.76	104495	pass	99.4878	pass	0
SAMN09833133	SRR7693661	PRJNA486009	*Escherichia coli*	Instituto de Microbiologia, Universidad San Francisco de Quito	Georgia Institute of Technology	2015-09-25	Ecuador	feces	Homo sapiens	Diseased	1.83 S 78.18 W		100	0.2	pass	E_coli_ASM584v2	115	4638912	50.81	114180	pass	99.1736	pass	0
SAMN09833105	SRR7693707	PRJNA514245	*Escherichia coli*	Instituto de Microbiologia, Universidad San Francisco de Quito	Georgia Institute of Technology	2014-05-18	Quito, Ecuador	feces	Homo sapiens	Diseased	0.18 S 78.46 W		100	0	pass	E_coli_ASM584v2	443	4440705	51	19763	fail	98.3637	pass	1
SAMN09833052	SRR7693669	PRJNA514245	*Escherichia coli*	Instituto de Microbiologia, Universidad San Francisco de Quito	Georgia Institute of Technology	2014-06-02	Esmeraldas, Ecuador	feces	Homo sapiens	Diseased	0.96 N 79.65 W		100	0.39	pass	E_coli_ASM584v2	426	5056210	50.93	26311	fail	99.2177	pass	1
SAMN09833020	SRR7693840	PRJNA486009	*Escherichia coli*	Instituto de Microbiologia, Universidad San Francisco de Quito	Georgia Institute of Technology	2014-07-21	Esmeraldas, Ecuador	feces	Homo sapiens	Diseased	0.96 N 79.65 W		100	0.03	pass	E_coli_ASM584v2	131	4996052	50.67	141200	pass	98.5608	pass	0
SAMN09833079	SRR7693682	PRJNA514245	*Escherichia coli*	Instituto de Microbiologia, Universidad San Francisco de Quito	Georgia Institute of Technology	2014-05-11	Quito, Ecuador	feces	Homo sapiens	Diseased	0.18 S 78.46 W		100	0.2	pass	E_coli_ASM584v2	237	5323984	50.43	58326	pass	97.1372	pass	0
SAMN09832985	SRR7693838	PRJNA486009	*Escherichia coli*	Instituto de Microbiologia, Universidad San Francisco de Quito	Georgia Institute of Technology	2015-07-18	Ecuador	Diarrhea	Homo sapiens	Diseased	1.8312 S 78.1834 W		100	0.48	pass	E_coli_ASM584v2	254	4982350	50.71	59443	pass	99.1635	pass	0
SAMN09833137	SRR7693657	PRJNA514245	*Escherichia coli*	Instituto de Microbiologia, Universidad San Francisco de Quito	Georgia Institute of Technology	2014-12-12	Colon Eloy, ecuador	feces	Homo sapiens	Diseased	0.73 N 79.06 W		100	0.09	pass	E_coli_ASM584v2	171	5176385	50.63	118736	pass	99.4715	pass	0
SAMN09833112	SRR7693622	PRJNA486009	*Escherichia coli*	Instituto de Microbiologia, Universidad San Francisco de Quito	Georgia Institute of Technology	2014-08-26	Quito, Ecuador	feces	Homo sapiens	Diseased	0.18 S 78.46 W		100	0	pass	E_coli_ASM584v2	122	5064603	50.64	143291	pass	98.407	pass	0

ID	SRA Run	Bioproject	Organism	Recollected	Submmiter	Collection date	Geo loc name	Isolation source	Host	Purpose of sampling	Lat ion	Hospital	Completeness_General	Contamination	1st Criterion	FastANI Reference	# contigs	Total length	GC (%)	N50	2nd Criterion	FastANI (%)	3rd Criterion	Failed criteria
SAMN09833108	SRR7693625	PRJNA514245	*Escherichia coli*	Instituto de Microbiologia, Universidad San Francisco de Quito	Georgia Institute of Technology	2014-05-19	Quito, Ecuador	feces	Homo sapiens	Diseased	0.18 S 78.46 W		100	0.06	pass	E_coli_ASM584v2	213	5110060	50.75	73680	pass	99.4811	pass	0
SAMN09833118	SRR7693613	PRJNA486009	*Escherichia coli*	Instituto de Microbiologia, Universidad San Francisco de Quito	Georgia Institute of Technology	2014-09-02	Quito, Ecuador	feces	Homo sapiens	Diseased	0.18 S 78.46 W		100	0.06	pass	E_coli_ASM584v2	174	5220719	50.63	95505	pass	99.4304	pass	0
SAMN09833038	SRR7693822	PRJNA486009	*Escherichia coli*	Instituto de Microbiologia, Universidad San Francisco de Quito	Georgia Institute of Technology	2014-09-05	Esmeraldas, Ecuador	Diarrhea	Homo sapiens	Diseased	0.96 N 79.65 W		99.83	0.74	pass	E_coli_ASM584v2	100	4798076	50.63	94110	pass	96.7292	pass	0
SAMN09832921	SRR7693772	PRJNA486009	*Escherichia coli*	Instituto de Microbiologia, Universidad San Francisco de Quito	Georgia Institute of Technology	2015-06-01	Maldonado, Ecuador	Diarrhea	Homo sapiens	Diseased	1.06 N 78.91 W		100	0.61	pass	E_coli_ASM584v2	177	5063070	50.61	97293	pass	96.6584	pass	0
SAMN09833138	SRR7693648	PRJNA514245	*Escherichia coli*	Instituto de Microbiologia, Universidad San Francisco de Quito	Georgia Institute of Technology	2014-12-12	Colon Eloy, ecuador	feces	Homo sapiens	Diseased	0.73 N 79.06 W		100	0.29	pass	E_coli_ASM584v2	229	4985710	50.58	83963	pass	98.9784	pass	0
SAMN09833103	SRR7693608	PRJNA514245	*Escherichia coli*	Instituto de Microbiologia, Universidad San Francisco de Quito	Georgia Institute of Technology	2014-09-23	Quito, Ecuador	feces	Homo sapiens	Diseased	0.18 S 78.46 W		99.89	0.89	pass	E_coli_ASM584v2	120	5088516	50.56	142798	pass	96.7277	pass	0
SAMN09833034	SRR7693813	PRJNA486009	*Escherichia coli*	Instituto de Microbiologia, Universidad San Francisco de Quito	Georgia Institute of Technology	2014-08-22	Esmeraldas, Ecuador	Diarrhea	Homo sapiens	Diseased	0.96 N 79.65 W		81.57	14	fail	E_coli_ASM584v2	3016	6100264	50.21	3031	fail	96.4319	pass	2
SAMN09832907	SRR7693834	PRJNA486009	*Escherichia coli*	Instituto de Microbiologia, Universidad San Francisco de Quito	Georgia Institute of Technology	2015-04-07	Borbon, Ecuador	Diarrhea	Homo sapiens	Diseased	1.08 N 78.99 W		99.85	0.24	pass	E_coli_ASM584v2	305	5247525	50.42	61426	pass	98.3722	pass	0
SAMN09833098	SRR7693754	PRJNA514245	*Escherichia coli*	Instituto de Microbiologia, Universidad San Francisco de Quito	Georgia Institute of Technology	2014-09-22	Quito, Ecuador	feces	Homo sapiens	Diseased	0.18 S 78.46 W		100	0.16	pass	E_coli_ASM584v2	200	4842343	50.52	80547	pass	99.1006	pass	0
SAMN09833083	SRR7693718	PRJNA514245	*Escherichia coli*	Instituto de Microbiologia, Universidad San Francisco de Quito	Georgia Institute of Technology	2014-09-16	Quito, Ecuador	feces	Homo sapiens	Diseased	0.18 S 78.46 W		100	0.11	pass	E_coli_ASM584v2	240	5011523	50.52	82232	pass	98.3581	pass	0
SAMN09832979	SRS3669977	PRJNA486009	*Escherichia coli*	Instituto de Microbiologia\, Universidad San Francisco de Quito	Georgia Institute of Technology	2015-05-04	Ecuador	feces	Homo sapiens	Diseased	1.83 S 78.18 W		100	0.06	pass	E_coli_ASM584v2	203	5198310	50.7	108060	pass	97.3708	pass	0
SAMN09832960	SRS3669947	PRJNA486009	*Escherichia coli*	Instituto de Microbiologia\, Universidad San Francisco de Quito	Georgia Institute of Technology	2014-10-03	Borbon, Ecuador	feces	Homo sapiens	Diseased	1.08 N 78.99 W		100	0.07	pass	E_coli_ASM584v2	137	4833504	50.91	96601	pass	99.2004	pass	0
SAMN09833042	SRR7693593	PRJNA486009	*Escherichia coli*	Instituto de Microbiologia\, Universidad San Francisco de Quito	Georgia Institute of Technology	2014-05-15	Esmeraldas, Ecuador	feces	Homo sapiens	Diseased	0.96 N 79.65 W		100	0.05	pass	E_coli_ASM584v2	184	4922968	50.6	72219	pass	99.2194	pass	0

ID	SRA Run	Bioproject	Organism	Recollected	Submmiter	Collection date	Geo loc name	Isolation source	Host	Purpose of sampling	Lat ion	Hospital	Completeness_General	Contamination	1st Criterion	FastANI Reference	# contigs	Total length	GC (%)	N50	2nd Criterion	FastANI (%)	3rd Criterion	Failed criteria
SAMN09832954	SRS3669937	PRJNA486009	*Escherichia coli*	Instituto de Microbiologia\, Universidad San Francisco de Quito	Georgia Institute of Technology	2014-08-29	Borbon, Ecuador	feces	Homo sapiens	Diseased	1.08 N 78.99 W		100	0.2	pass	E_coli_ASM584v2	117	4892419	50.77	119370	pass	99.0938	pass	0
SAMN09832990	SRR7693736	PRJNA486009	*Escherichia coli*	Instituto de Microbiologia\, Universidad San Francisco de Quito	Georgia Institute of Technology	2015-01-12	Ecuador	feces	Homo sapiens	Diseased			99.96	0.09	pass	E_coli_ASM584v2	252	5062701	50.61	79349	pass	98.3462	pass	0
SAMN09832942	SRR7693694	PRJNA486009	*Escherichia coli*	Instituto de Microbiologia\, Universidad San Francisco de Quito	Georgia Institute of Technology	2014-06-18	Borbon, Ecuador	feces	Homo sapiens	Diseased	1.08 N 78.99 W		100	0.13	pass	E_coli_ASM584v2	82	4728919	50.66	185027	pass	99.547	pass	0
SAMN09832969	SRS3669961	PRJNA486009	*Escherichia coli*	Instituto de Microbiologia\, Universidad San Francisco de Quito	Georgia Institute of Technology	2015-01-15	Ecuador	feces	Homo sapiens	Diseased	1.83 S 78.18 W		100	0.28	pass	E_coli_ASM584v2	211	5237894	50.58	103421	pass	97.279	pass	0
SAMN09832959	SRS3669958	PRJNA486009	*Escherichia coli*	Instituto de Microbiologia\, Universidad San Francisco de Quito	Georgia Institute of Technology	2014-10-01	Borbon, Ecuador	feces	Homo sapiens	Diseased	1.08 N 78.99 W		100	0.13	pass	E_coli_ASM584v2	365	5072150	50.81	34545	fail	98.8773	pass	1
SAMN09832948	SRS3669902	PRJNA486009	*Escherichia coli*	Instituto de Microbiologia\, Universidad San Francisco de Quito	Georgia Institute of Technology	2014-07-30	Colon Eloy, Ecuador	feces	Homo sapiens	Diseased	0.73 N 79.06 W		100	0.07	pass	E_coli_ASM584v2	250	4871155	50.64	65524	pass	99.0761	pass	0
SAMN09832953	SRS3669970	PRJNA486009	*Escherichia coli*	Instituto de Microbiologia\, Universidad San Francisco de Quito	Georgia Institute of Technology	2014-08-25	Ecuador	feces	Homo sapiens	Diseased	1.06 N 78.91 W		100	0.1	pass	E_coli_ASM584v2	72	4657076	50.6	154849	pass	98.3599	pass	0
SAMN09833040	SRS3669989	PRJNA486009	*Escherichia coli*	Instituto de Microbiologia\, Universidad San Francisco de Quito	Georgia Institute of Technology	2014-05-14	Esmeraldas, Ecuador	feces	Homo sapiens	Diseased	0.96 N 79.65 W		100	0.47	pass	E_coli_ASM584v2	102	5040028	50.69	174051	pass	96.7866	pass	0
SAMN09832977	SRS3669936	PRJNA486009	*Escherichia coli*	Instituto de Microbiologia\, Universidad San Francisco de Quito	Georgia Institute of Technology	2015-04-30	Ecuador	feces	Homo sapiens	Diseased	1.83 S 78.18 W		100	0.03	pass	E_coli_ASM584v2	240	4816983	50.58	46599	pass	98.4296	pass	0
SAMN09832946	SRS3669897	PRJNA486009	*Escherichia coli*	Instituto de Microbiologia\, Universidad San Francisco de Quito	Georgia Institute of Technology	2014-06-27	Borbon, Ecuador	feces	Homo sapiens	Diseased	1.08 N 78.99 W		100	0.17	pass	E_coli_ASM584v2	93	4577327	50.65	108852	pass	98.9246	pass	0
SAMN09832945	SRS3669900	PRJNA486009	*Escherichia coli*	Instituto de Microbiologia\, Universidad San Francisco de Quito	Georgia Institute of Technology	2014-06-18	Borbon, Ecuador	feces	Homo sapiens	Diseased	1.08 N 78.99 W		100	0.03	pass	E_coli_ASM584v2	26	4385933	50.65	412442	pass	92.9081	pass	0
SAMN09832991	SRR7693737	PRJNA486009	*Escherichia coli*	Instituto de Microbiologia\, Universidad San Francisco de Quito	Georgia Institute of Technology	2014-06-17	Esmeraldas, Ecuador	feces	Homo sapiens	Diseased	0.96 N 79.65 W		100	0.29	pass	E_coli_ASM584v2	155	5200994	50.65	92288	pass	97.2777	pass	0
SAMN09832958	SRS3669959	PRJNA486009	*Escherichia coli*	Instituto de Microbiologia\, Universidad San Francisco de Quito	Georgia Institute of Technology	2014-09-30	Borbon, Ecuador	feces	Homo sapiens	Diseased	1.08 N 78.99 W		100	0.38	pass	E_coli_ASM584v2	357	4856951	50.68	32989	fail	98.2992	pass	1

ID	SRA Run	Bioproject	Organism	Recollected	Submmiter	Collection date	Geo loc name	Isolation source	Host	Purpose of sampling	Lat ion	Hospital	Completeness_General	Contamination	1st Criterion	FastANI Reference	# contigs	Total length	GC (%)	N50	2nd Criterion	FastANI (%)	3rd Criterion	Failed criteria
SAMN09832943	SRS3669998	PRJNA486009	*Escherichia coli*	Instituto de Microbiologia\, Universidad San Francisco de Quito	Georgia Institute of Technology	2014-06-18	Borbon, Ecuador	feces	Homo sapiens	Diseased	1.08 N 78.99 W		99.78	0.25	pass	E_coli_ASM584v2	253	4989495	50.63	58017	pass	99.4276	pass	0
SAMN09832997	SRS3669845	PRJNA486009	*Escherichia coli*	Instituto de Microbiologia\, Universidad San Francisco de Quito	Georgia Institute of Technology	2014-06-20	Esmeraldas, Ecuador	feces	Homo sapiens	Diseased	0.96 N 79.65 W		99.89	0.81	pass	E_coli_ASM584v2	126	5118534	50.55	113392	pass	96.9822	pass	0
SAMN09832966	SRS3669934	PRJNA486009	*Escherichia coli*	Instituto de Microbiologia\, Universidad San Francisco de Quito	Georgia Institute of Technology	missing	Ecuador	feces	Homo sapiens	Diseased	1.83 S 78.18 W		100	0.06	pass	E_coli_ASM584v2	86	4876143	50.72	158343	pass	99.0829	pass	0
SAMN09832957	SRS3669903	PRJNA486009	*Escherichia coli*	Instituto de Microbiologia\, Universidad San Francisco de Quito	Georgia Institute of Technology	2014-09-30	Borbon, Ecuador	feces	Homo sapiens	Diseased	1.08 N 78.99 W		100	0.17	pass	E_coli_ASM584v2	157	4749814	50.62	78000	pass	98.4028	pass	0
SAMN09832994	SRR7693740	PRJNA486009	*Escherichia coli*	Instituto de Microbiologia\, Universidad San Francisco de Quito	Georgia Institute of Technology	2014-06-17	Esmeraldas, Ecuador	feces	Homo sapiens	Diseased	0.96 N 79.65 W		100	0.08	pass	E_coli_ASM584v2	107	5186619	50.6	179981	pass	98.3713	pass	0
SAMN09832890	SRR7693759	PRJNA486009	*Escherichia coli*	Instituto de Microbiologia\, Universidad San Francisco de Quito	Georgia Institute of Technology	2014-04-24	Las Peñas, Ecuador	feces	Homo sapiens	Diseased	1.08 N 79.15 W		100	0.22	pass	E_coli_ASM584v2	188	5148180	50.62	92931	pass	99.1754	pass	0
SAMN09832951	SRS3669979	PRJNA486009	*Escherichia coli*	Instituto de Microbiologia\, Universidad San Francisco de Quito	Georgia Institute of Technology	2014-08-14	Santo Domingo, Ecuador	feces	Homo sapiens	Diseased	0.25 S 79.17 W		100	0.29	pass	E_coli_ASM584v2	138	5043046	50.52	157408	pass	99.3897	pass	0
SAMN09832961	SRS3669946	PRJNA486009	*Escherichia coli*	Instituto de Microbiologia\, Universidad San Francisco de Quito	Georgia Institute of Technology	2014-10-07	Ecuador	feces	Homo sapiens	Diseased	1.06 N 78.91 W		100	0.5	pass	E_coli_ASM584v2	174	5304296	50.64	123087	pass	97.1901	pass	0
SAMN09832947	SRS3669898	PRJNA486009	*Escherichia coli*	Instituto de Microbiologia\, Universidad San Francisco de Quito	Georgia Institute of Technology	2014-06-27	Borbon, Ecuador	feces	Homo sapiens	Diseased	1.08 N 78.99 W		100	0.17	pass	E_coli_ASM584v2	100	4568315	50.68	102385	pass	98.9552	pass	0
SAMN09832955	SRS3669963	PRJNA486009	*Escherichia coli*	Instituto de Microbiologia\, Universidad San Francisco de Quito	Georgia Institute of Technology	2014-09-24	Borbon, Ecuador	feces	Homo sapiens	Diseased	1.08 N 78.99 W		95.93	0.85	pass	E_coli_ASM584v2	110	5069379	50.8	124350	pass	96.7705	pass	0
SAMN09832932	SRR7693729	PRJNA486009	*Escherichia coli*	Instituto de Microbiologia\, Universidad San Francisco de Quito	Georgia Institute of Technology	missing	Borbon, Ecuador	feces	Homo sapiens	Diseased	1.08 N 78.99 W		100	0.09	pass	E_coli_ASM584v2	72	4705566	50.81	154679	pass	98.4467	pass	0
SAMN09832963	SRS3669950	PRJNA486009	*Escherichia coli*	Instituto de Microbiologia\, Universidad San Francisco de Quito	Georgia Institute of Technology	2015-08-22	Ecuador	feces	Homo sapiens	Diseased	1.83 S 78.18 W		64.8	4.38	fail	E_coli_ASM584v2	2573	3696887	49.71	1730	fail	96.0707	pass	2
SAMN09832984	SRS3669956	PRJNA486009	*Escherichia coli*	Instituto de Microbiologia\, Universidad San Francisco de Quito	Georgia Institute of Technology	missing	Ecuador	feces	Homo sapiens	Diseased	1.83 S 78.18 W		100	0.79	pass	E_coli_ASM584v2	155	5446685	50.49	88605	pass	97.1962	pass	0

ID	SRA Run	Bioproject	Organism	Recollected	Submmiter	Collection date	Geo loc name	Isolation source	Host	Purpose of sampling	Lat ion	Hospital	Completeness_General	Contamination	1st Criterion	FastANI Reference	# contigs	Total length	GC (%)	N50	2nd Criterion	FastANI (%)	3rd Criterion	Failed criteria
SAMN09832950	SRS3669982	PRJNA486009	*Escherichia coli*	Instituto de Microbiologia\, Universidad San Francisco de Quito	Georgia Institute of Technology	2014-08-01	Borbon, Ecuador	feces	Homo sapiens	Diseased	1.08 N 78.99 W		100	0.24	pass	E_coli_ASM584v2	195	4904342	50.56	72846	pass	99.1088	pass	0
SAMN09832930	SRR7693731	PRJNA486009	*Escherichia coli*	Instituto de Microbiologia\, Universidad San Francisco de Quito	Georgia Institute of Technology	2015-07-07	Borbon, Ecuador	feces	Homo sapiens	Diseased	1.08 N 78.99 W		100	0.2	pass	E_coli_ASM584v2	149	4832930	50.82	88777	pass	99.5542	pass	0
SAMN09832933	SRR7693730	PRJNA486009	*Escherichia coli*	Instituto de Microbiologia\, Universidad San Francisco de Quito	Georgia Institute of Technology	2015-08-05	Borbon, Ecuador	feces	Homo sapiens	Diseased	1.08 N 78.99 W		100	0.1	pass	E_coli_ASM584v2	241	4697176	50.72	49387	pass	99.1563	pass	0
SAMN09832992	SRR7693738	PRJNA486009	*Escherichia coli*	Instituto de Microbiologia\, Universidad San Francisco de Quito	Georgia Institute of Technology	2014-06-17	Esmeraldas, Ecuador	feces	Homo sapiens	Diseased	0.96 N 79.65 W		100	0.12	pass	E_coli_ASM584v2	170	5303455	50.42	65208	pass	97.1334	pass	0
SAMN09832935	SRR7693728	PRJNA486009	*Escherichia coli*	Instituto de Microbiologia\, Universidad San Francisco de Quito	Georgia Institute of Technology	2015-09-01	Borbon, Ecuador	feces	Homo sapiens	Diseased	1.08 N 78.99 W		100	0.8	pass	E_coli_ASM584v2	48	4775835	50.71	188808	pass	96.7809	pass	0
SAMN09832893	SRS3669832	PRJNA486009	*Escherichia coli*	Instituto de Microbiologia\, Universidad San Francisco de Quito	Georgia Institute of Technology	2014-10-30	Borbon, Ecuador	feces	Homo sapiens	Diseased	1.08 N 78.99 W		98.84	0.04	pass	E_coli_ASM584v2	305	4993874	50.9	39585	fail	98.4007	pass	1
SAMN09832896	SRR7693761	PRJNA486009	*Escherichia coli*	Instituto de Microbiologia\, Universidad San Francisco de Quito	Georgia Institute of Technology	2014-02-24	Borbon, Ecuador	feces	Homo sapiens	Diseased	1.08 N 78.99 W		100	0.3	pass	E_coli_ASM584v2	161	5130518	50.5	152417	pass	99.3593	pass	0
SAMN09832894	SRR7693763	PRJNA486009	*Escherichia coli*	Instituto de Microbiologia\, Universidad San Francisco de Quito	Georgia Institute of Technology	2014-11-09	Borbon, Ecuador	feces	Homo sapiens	Diseased	1.08 N 78.99 W		100	0.2	pass	E_coli_ASM584v2	208	4794966	50.48	57443	pass	98.4247	pass	0
SAMN09832967	SRS3669935	PRJNA486009	*Escherichia coli*	Instituto de Microbiologia\, Universidad San Francisco de Quito	Georgia Institute of Technology	2015-01-14	Ecuador	feces	Homo sapiens	Diseased	1.83 S 78.18 W		100	0.09	pass	E_coli_ASM584v2	107	4701868	50.56	130995	pass	99.4858	pass	0
SAMN09832976	SRS3669938	PRJNA486009	*Escherichia coli*	Instituto de Microbiologia\, Universidad San Francisco de Quito	Georgia Institute of Technology	2015-04-15	Ecuador	feces	Homo sapiens	Diseased	1.83 S 78.18 W		100	0.2	pass	E_coli_ASM584v2	247	5083555	50.7	52368	pass	98.2689	pass	0
SAMN09832998	SRR7693733	PRJNA486009	*Escherichia coli*	Instituto de Microbiologia\, Universidad San Francisco de Quito	Georgia Institute of Technology	2014-06-23	Borbon, Ecuador	feces	Homo sapiens	Diseased	1.08 N 78.99 W		100	0.01	pass	E_coli_ASM584v2	228	5183519	50.54	76452	pass	99.0087	pass	0
SAMN09832934	SRS3669931	PRJNA486009	*Escherichia coli*	Instituto de Microbiologia\, Universidad San Francisco de Quito	Georgia Institute of Technology	2015-08-27	Borbon, Ecuador	feces	Homo sapiens	Diseased	1.08 N 78.99 W		100	0.06	pass	E_coli_ASM584v2	377	4528325	50.78	23499	fail	98.2539	pass	1
SAMN09832897	SRR7693760	PRJNA486009	*Escherichia coli*	Instituto de Microbiologia\, Universidad San Francisco de Quito	Georgia Institute of Technology	2014-02-24	Borbon, Ecuador	feces	Homo sapiens	Diseased	1.08 N 78.99 W		100	0.06	pass	E_coli_ASM584v2	106	5138184	50.64	139900	pass	97.2564	pass	0

ID	SRA Run	Bioproject	Organism	Recollected	Submmiter	Collection date	Geo loc name	Isolation source	Host	Purpose of sampling	Lat ion	Hospital	Completeness_General	Contamination	1st Criterion	FastANI Reference	# contigs	Total length	GC (%)	N50	2nd Criterion	FastANI (%)	3rd Criterion	Failed criteria
SAMN09832968	SRS3669957	PRJNA486009	*Escherichia coli*	Instituto de Microbiologia\, Universidad San Francisco de Quito	Georgia Institute of Technology	2015-01-15	Ecuador	feces	Homo sapiens	Diseased	1.83 S 78.18 W		100	0.19	pass	E_coli_ASM584v2	247	5525255	50.47	88833	pass	97.1748	pass	0
SAMN09832996	SRS3669848	PRJNA486009	*Escherichia coli*	Instituto de Microbiologia\, Universidad San Francisco de Quito	Georgia Institute of Technology	2014-06-20	Esmeraldas, Ecuador	feces	Homo sapiens	Diseased	0.96 N 79.65 W		100	0.08	pass	E_coli_ASM584v2	126	5220537	50.61	134101	pass	98.3376	pass	0
SAMN09832975	SRS3669904	PRJNA486009	*Escherichia coli*	Instituto de Microbiologia\, Universidad San Francisco de Quito	Georgia Institute of Technology	2015-04-11	Ecuador	feces	Homo sapiens	Diseased	1.83 S 78.18 W		100	0.01	pass	E_coli_ASM584v2	177	5111810	50.76	97632	pass	97.3013	pass	0
SAMN09832944	SRS3669899	PRJNA486009	*Escherichia coli*	Instituto de Microbiologia\, Universidad San Francisco de Quito	Georgia Institute of Technology	2014-06-18	Borbon, Ecuador	feces	Homo sapiens	Diseased	1.08 N 78.99 W		100	0.1	pass	E_coli_ASM584v2	111	4918308	50.48	175519	pass	98.3655	pass	0
SAMN09832952	SRS3669981	PRJNA486009	*Escherichia coli*	Instituto de Microbiologia\, Universidad San Francisco de Quito	Georgia Institute of Technology	2014-08-14	Santo Domingo, Ecuador	feces	Homo sapiens	Diseased	0.25 S 79.17 W		100	0.33	pass	E_coli_ASM584v2	123	5078051	50.51	152414	pass	99.4016	pass	0
SAMN09832978	SRS3669978	PRJNA486009	*Escherichia coli*	Instituto de Microbiologia\, Universidad San Francisco de Quito	Georgia Institute of Technology	2015-05-01	Ecuador	feces	Homo sapiens	Diseased	1.83 S 78.18 W		100	0.08	pass	E_coli_ASM584v2	138	4887604	50.45	137350	pass	98.4884	pass	0
SAMEA111504311	ERS13616673	PRJNA514245	*Escherichia coli*	Teodoro Maldonado Carbo Hospital	EBI	2020-08-13	Ecuador	drainage	Homo sapiens	Diseased	2.203816 S 79.897453 W	Teodoro Maldonado Carbo Hospital	99.9	0.81	pass	E_coli_ASM584v2	131	5001527	50.7	100766	pass	96.8793	pass	0
SAMN50147144	SRR34673899	PRJNA1293794	*Escherichia coli*	UEES	UEES	2020	Guayas, Guayaquil, Ecuador	urine	Homo sapiens	Diseased	2.176570 S 79.883580 W	Hospital de niños Dr Roberto Gilbert	94.4	4.42	pass	E_coli_ASM584v2	1030	4584384	51.04	6439	fail	98.2635	pass	1
SAMN50147142	SRR34673901	PRJNA1293794	*Escherichia coli*	UEES	UEES	2020	Guayas, Guayaquil, Ecuador	urine	Homo sapiens	Diseased	2.176570 S 79.883580 W	Hospital de niños Dr Roberto Gilbert	97.2	1.59	pass	E_coli_ASM584v2	839	4527411	51.21	7977	fail	96.8985	pass	1
SAMN50147143	SRR34673900	PRJNA1293794	*Escherichia coli*	UEES	UEES	2020	Guayas, Guayaquil, Ecuador	urine	Homo sapiens	Diseased	2.176570 S 79.883580 W	Hospital de niños Dr Roberto Gilbert	91.95	3.68	pass	E_coli_ASM584v2	1038	4733086	50.9	6290	fail	96.7752	pass	1
SAMN50147145	SRR34673898	PRJNA1293794	*Escherichia coli*	UEES	UEES	2020	Guayas, Guayaquil, Ecuador	urine	Homo sapiens	Diseased	2.176570 S 79.883580 W	Hospital de niños Dr Roberto Gilbert	91.84	2.27	pass	E_coli_ASM584v2	1261	4676314	51.35	5004	fail	97.2754	pass	1
SAMN09833096	SRR7693749	PRJNA514245	*Escherichia coli*	Instituto de Microbiologia, Universidad San Francisco de Quito	Georgia Institute of Technology	2014-09-19	Quito, Ecuador	feces	Homo sapiens	Surveillance	0.18 S 78.46 W		100	0.07	pass	E_coli_ASM584v2	196	4909181	50.58	63247	pass	99.2029	pass	0
SAMN09833078	SRR7693681	PRJNA514245	*Escherichia coli*	Instituto de Microbiologia, Universidad San Francisco de Quito	Georgia Institute of Technology	2014-09-12	Quito, Ecuador	feces	Homo sapiens	Surveillance	0.18 S 78.46 W		100	0.38	pass	E_coli_ASM584v2	190	5037526	50.8	95763	pass	99.0553	pass	0
SAMN09833097	SRR7693748	PRJNA514245	*Escherichia coli*	Instituto de Microbiologia, Universidad San Francisco de Quito	Georgia Institute of Technology	2014-09-19	Quito, Ecuador	feces	Homo sapiens	Surveillance	0.18 S 78.46 W		100	0.2	pass	E_coli_ASM584v2	187	5024843	50.83	87166	pass	99.5023	pass	0

ID	SRA Run	Bioproject	Organism	Recollected	Submmiter	Collection date	Geo loc name	Isolation source	Host	Purpose of sampling	Lat ion	Hospital	Completeness_General	Contamination	1st Criterion	FastANI Reference	# contigs	Total length	GC (%)	N50	2nd Criterion	FastANI (%)	3rd Criterion	Failed criteria
SAMN09833056	SRR7693665	PRJNA486009	*Escherichia coli*	Instituto de Microbiologia, Universidad San Francisco de Quito	Georgia Institute of Technology	2014-06-05	Esmeraldas, Ecuador	feces	Homo sapiens	Surveillance	0.96 N 79.65 W		98.47	0.24	pass	E_coli_ASM584v2	333	5038933	50.59	30823	fail	99.4186	pass	1
SAMN09832970	SRR7693766	PRJNA486009	*Escherichia coli*	Instituto de Microbiologia, Universidad San Francisco de Quito	Georgia Institute of Technology	2015-02-24	Ecuador	diarrhea	Homo sapiens	Surveillance	1.8312 S 78.1834 W		100	0.14	pass	E_coli_ASM584v2	204	5075538	50.63	66707	pass	99.1545	pass	0
SAMN09833109	SRR7693628	PRJNA486009	*Escherichia coli*	Instituto de Microbiologia, Universidad San Francisco de Quito	Georgia Institute of Technology	2014-05-19	Quito, Ecuador	feces	Homo sapiens	Surveillance	0.18 S 78.46 W		100	0.22	pass	E_coli_ASM584v2	304	5148256	50.52	47514	pass	98.2935	pass	0
SAMN09833119	SRR7693612	PRJNA486009	*Escherichia coli*	Instituto de Microbiologia, Universidad San Francisco de Quito	Georgia Institute of Technology	2014-09-02	Quito, Ecuador	feces	Homo sapiens	Surveillance	0.18 S 78.46 W		100	0.16	pass	E_coli_ASM584v2	239	4656783	50.81	52637	pass	99.4305	pass	0
SAMN09832920	SRR7693773	PRJNA486009	*Escherichia coli*	Instituto de Microbiologia, Universidad San Francisco de Quito	Georgia Institute of Technology	2015-05-28	Borbon, Ecuador	stool	Homo sapiens	Surveillance	1.08 N 78.99 W		100	0.17	pass	E_coli_ASM584v2	338	5038881	50.79	39648	fail	99.5441	pass	1
SAMN09833146	SRR7693633	PRJNA514245	*Escherichia coli*	Instituto de Microbiologia, Universidad San Francisco de Quito	Georgia Institute of Technology	2015-04-20	Selva Alegre, Ecuador	feces	Homo sapiens	Surveillance	0.33 N 78.19 W		100	0.21	pass	E_coli_ASM584v2	175	5018873	50.67	111067	pass	99.4335	pass	0
SAMN09833016	SRR7693807	PRJNA486009	*Escherichia coli*	Instituto de Microbiologia, Universidad San Francisco de Quito	Georgia Institute of Technology	2014-07-18	Esmeraldas, Ecuador	stool	Homo sapiens	Surveillance	0.96 N 79.65 W		100	0.08	pass	E_coli_ASM584v2	145	4943580	50.56	106116	pass	98.3799	pass	0
SAMN09833128	SRR7693821	PRJNA486009	*Escherichia coli*	Instituto de Microbiologia, Universidad San Francisco de Quito	Georgia Institute of Technology	2015-08-17	Ecuador	feces	Homo sapiens	Surveillance	1.83 S 78.18 W		100	0.02	pass	E_coli_ASM584v2	193	4874627	50.74	69629	pass	99.1532	pass	0
SAMN09833073	SRR7693676	PRJNA514245	*Escherichia coli*	Instituto de Microbiologia, Universidad San Francisco de Quito	Georgia Institute of Technology	2014-09-04	Quito, Ecuador	feces	Homo sapiens	Surveillance	0.18 S 78.46 W		100	0.04	pass	E_coli_ASM584v2	194	5170145	50.74	107724	pass	99.4568	pass	0
SAMN09833125	SRR7693850	PRJNA486009	*Escherichia coli*	Instituto de Microbiologia, Universidad San Francisco de Quito	Georgia Institute of Technology	2015-08-14	Ecuador	feces	Homo sapiens	Surveillance	1.83 S 78.18 W		100	0.37	pass	E_coli_ASM584v2	158	4756144	50.74	81462	pass	98.7913	pass	0
SAMN09832938	SRR7693723	PRJNA486009	*Escherichia coli*	Instituto de Microbiologia, Universidad San Francisco de Quito	Georgia Institute of Technology	2015-09-17	Borbon, ecuador	feces	Homo sapiens	Surveillance	1.08 N 78.99 W		100	0.07	pass	E_coli_ASM584v2	124	4703846	50.64	99541	pass	98.4373	pass	0
SAMN09833049	SRR7693601	PRJNA486009	*Escherichia coli*	Instituto de Microbiologia, Universidad San Francisco de Quito	Georgia Institute of Technology	2014-06-02	Esmeraldas, Ecuador	feces	Homo sapiens	Surveillance	0.96 N 79.65 W		100	0.15	pass	E_coli_ASM584v2	471	5111248	50.93	21325	fail	97.2828	pass	1
SAMN09832988	SRR7693800	PRJNA486009	*Escherichia coli*	Instituto de Microbiologia, Universidad San Francisco de Quito	Georgia Institute of Technology	2015-08-02	Ecuador	stool	Homo sapiens	Surveillance	1.8312 S 78.1834 W		99.35	6.24	fail	E_coli_ASM584v2	2472	4763755	50.18	2636	fail	98.2054	pass	2

ID	SRA Run	Bioproject	Organism	Recollected	Submmiter	Collection date	Geo loc name	Isolation source	Host	Purpose of sampling	Lat ion	Hospital	Completeness_General	Contamination	1st Criterion	FastANI Reference	# contigs	Total length	GC (%)	N50	2nd Criterion	FastANI (%)	3rd Criterion	Failed criteria
SAMN09833129	SRR7693824	PRJNA486009	*Escherichia coli*	Instituto de Microbiologia, Universidad San Francisco de Quito	Georgia Institute of Technology	2015-08-22	Ecuador	feces	Homo sapiens	Surveillance	1.83 S 78.18 W		100	0.11	pass	E_coli_ASM584v2	213	4746039	50.7	73502	pass	99.1174	pass	0
SAMN09833124	SRR7693849	PRJNA486009	*Escherichia coli*	Instituto de Microbiologia, Universidad San Francisco de Quito	Georgia Institute of Technology	2015-08-14	Esmeraldas, Ecuador	feces	Homo sapiens	Surveillance	0.96 N 79.65 W		100	0.22	pass	E_coli_ASM584v2	290	5027015	50.4	53196	pass	98.2868	pass	0
SAMN09832901	SRR7693828	PRJNA486009	*Escherichia coli*	Instituto de Microbiologia, Universidad San Francisco de Quito	Georgia Institute of Technology	2015-01-20	Borbon, Ecuador	stool	Homo sapiens	Surveillance	1.08 N 78.99 W		100	0.08	pass	E_coli_ASM584v2	214	5069054	50.67	71085	pass	98.2957	pass	0
SAMN09833085	SRR7693716	PRJNA514245	*Escherichia coli*	Instituto de Microbiologia, Universidad San Francisco de Quito	Georgia Institute of Technology	2014-09-16	Quito, Ecuador	feces	Homo sapiens	Surveillance	0.18 S 78.46 W		100	0.17	pass	E_coli_ASM584v2	157	5311679	50.36	135866	pass	97.2286	pass	0
SAMN09833045	SRR7693598	PRJNA486009	*Escherichia coli*	Instituto de Microbiologia, Universidad San Francisco de Quito	Georgia Institute of Technology	2014-05-19	Esmeraldas, Ecuador	feces	Homo sapiens	Surveillance	0.96 N 79.65 W		100	0.07	pass	E_coli_ASM584v2	299	5032985	50.66	42057	pass	98.9151	pass	0
SAMN09833017	SRR7693808	PRJNA486009	*Escherichia coli*	Instituto de Microbiologia, Universidad San Francisco de Quito	Georgia Institute of Technology	2014-07-18	Esmeraldas, Ecuador	stool	Homo sapiens	Surveillance	0.96 N 79.65 W		100	0.17	pass	E_coli_ASM584v2	70	4861888	50.58	259882	pass	98.3442	pass	0
SAMN09833080	SRR7693721	PRJNA514245	*Escherichia coli*	Instituto de Microbiologia, Universidad San Francisco de Quito	Georgia Institute of Technology	2014-09-11	Quito, Ecuador	feces	Homo sapiens	Surveillance	0.18 S 78.46 W		100	0.08	pass	E_coli_ASM584v2	107	4908479	50.52	173247	pass	99.5526	pass	0
SAMN09833030	SRR7693817	PRJNA486009	*Escherichia coli*	Instituto de Microbiologia, Universidad San Francisco de Quito	Georgia Institute of Technology	2014-04-30	Esmeraldas, Ecuador	stool	Homo sapiens	Surveillance	0.96 N 79.65 W		100	0.36	pass	E_coli_ASM584v2	146	4994199	50.68	89993	pass	98.8447	pass	0
SAMN09833088	SRR7693713	PRJNA514245	*Escherichia coli*	Instituto de Microbiologia, Universidad San Francisco de Quito	Georgia Institute of Technology	2014-09-17	Quito, Ecuador	feces	Homo sapiens	Surveillance	0.18 S 78.46 W		98.91	0.17	pass	E_coli_ASM584v2	245	5192022	50.58	50378	pass	97.3307	pass	0
SAMN09833076	SRR7693679	PRJNA514245	*Escherichia coli*	Instituto de Microbiologia, Universidad San Francisco de Quito	Georgia Institute of Technology	2014-09-10	Quito, Ecuador	feces	Homo sapiens	Surveillance	0.18 S 78.46 W		100	0.41	pass	E_coli_ASM584v2	213	5180840	50.68	107370	pass	98.339	pass	0
SAMN09832918	SRR7693801	PRJNA486009	*Escherichia coli*	Instituto de Microbiologia, Universidad San Francisco de Quito	Georgia Institute of Technology	2015-05-27	Borbon, Ecuador	stool	Homo sapiens	Surveillance	1.08 N 78.99 W		100	0.57	pass	E_coli_ASM584v2	181	5052404	50.6	93929	pass	96.71	pass	0
SAMN09833094	SRR7693751	PRJNA514245	*Escherichia coli*	Instituto de Microbiologia, Universidad San Francisco de Quito	Georgia Institute of Technology	2014-09-19	Quito, Ecuador	feces	Homo sapiens	Surveillance	0.18 S 78.46 W		83.65	13.75	fail	E_coli_ASM584v2	2952	4706430	49.93	2121	fail	97.2501	pass	2
SAMN09833095	SRR7693750	PRJNA514245	*Escherichia coli*	Instituto de Microbiologia, Universidad San Francisco de Quito	Georgia Institute of Technology	2014-09-19	Quito, Ecuador	feces	Homo sapiens	Surveillance	0.18 S 78.46 W		100	0.51	pass	E_coli_ASM584v2	289	5190966	50.39	53814	pass	98.3238	pass	0

ID	SRA Run	Bioproject	Organism	Recollected	Submmiter	Collection date	Geo loc name	Isolation source	Host	Purpose of sampling	Lat ion	Hospital	Completeness_General	Contamination	1st Criterion	FastANI Reference	# contigs	Total length	GC (%)	N50	2nd Criterion	FastANI (%)	3rd Criterion	Failed criteria
SAMN09833123	SRR7693852	PRJNA486009	*Escherichia coli*	Instituto de Microbiologia, Universidad San Francisco de Quito	Georgia Institute of Technology	2015-08-11	Ecuador	feces	Homo sapiens	Surveillance	1.83 S 78.18 W		100	0.45	pass	E_coli_ASM584v2	204	5000451	50.78	72985	pass	98.7374	pass	0
SAMN09832974	SRR7693786	PRJNA486009	*Escherichia coli*	Instituto de Microbiologia, Universidad San Francisco de Quito	Georgia Institute of Technology	2015-02-27	Ecuador	stool	Homo sapiens	Surveillance	1.8312 S 78.1834 W		100	0.13	pass	E_coli_ASM584v2	132	4973327	50.61	126345	pass	98.2874	pass	0
SAMN09833120	SRR7693853	PRJNA486009	*Escherichia coli*	Instituto de Microbiologia, Universidad San Francisco de Quito	Georgia Institute of Technology	2014-09-02	Quito, Ecuador	feces	Homo sapiens	Surveillance	0.18 S 78.46 W		100	0.11	pass	E_coli_ASM584v2	142	4973709	50.52	114689	pass	98.4024	pass	0
SAMN09833036	SRR7693815	PRJNA486009	*Escherichia coli*	Instituto de Microbiologia, Universidad San Francisco de Quito	Georgia Institute of Technology	2014-09-24	Esmeraldas, Ecuador	stool	Homo sapiens	Surveillance	0.96 N 79.65 W		99.79	0.4	pass	E_coli_ASM584v2	361	4944480	50.71	30666	fail	99.4214	pass	1
SAMN09833101	SRR7693711	PRJNA514245	*Escherichia coli*	Instituto de Microbiologia, Universidad San Francisco de Quito	Georgia Institute of Technology	2014-09-23	Quito, Ecuador	feces	Homo sapiens	Surveillance	0.18 S 78.46 W		100	0.02	pass	E_coli_ASM584v2	207	5087344	50.8	65608	pass	99.5241	pass	0
SAMN09833019	SRR7693802	PRJNA486009	*Escherichia coli*	Instituto de Microbiologia, Universidad San Francisco de Quito	Georgia Institute of Technology	2015-05-27	Borbon, Ecuador	stool	Homo sapiens	Surveillance	1.08 N 78.99 W		100	0.12	pass	E_coli_ASM584v2	72	4621915	50.81	193657	pass	98.4355	pass	0
SAMN09833141	SRR7693643	PRJNA486009	*Escherichia coli*	Instituto de Microbiologia, Universidad San Francisco de Quito	Georgia Institute of Technology	2015-02-20	Ecuador	feces	Homo sapiens	Surveillance	1.83 S 78.18 W		100	0.43	pass	E_coli_ASM584v2	282	5357930	50.44	46972	pass	97.2536	pass	0
SAMN09832903	SRR7693830	PRJNA486009	*Escherichia coli*	Instituto de Microbiologia, Universidad San Francisco de Quito	Georgia Institute of Technology	2015-01-24	Borbon, Ecuador	stool	Homo sapiens	Surveillance	1.08 N 78.99 W		100	0.07	pass	E_coli_ASM584v2	121	4806368	50.58	128899	pass	99.5601	pass	0
SAMN09833149	SRR7693635	PRJNA514245	*Escherichia coli*	Instituto de Microbiologia, Universidad San Francisco de Quito	Georgia Institute of Technology	2014-10-15	Ecuador	feces	Homo sapiens	Surveillance	1.83 S 78.18 W		100	0.42	pass	E_coli_ASM584v2	195	5059001	50.75	94475	pass	99.0883	pass	0
SAMN09832971	SRR7693769	PRJNA486009	*Escherichia coli*	Instituto de Microbiologia, Universidad San Francisco de Quito	Georgia Institute of Technology	2015-02-24	Ecuador	stool	Homo sapiens	Surveillance	1.8312 S 78.1834 W		100	0.14	pass	E_coli_ASM584v2	177	5080144	50.62	81087	pass	99.1446	pass	0
SAMN09833061	SRR7693702	PRJNA514245	*Escherichia coli*	Instituto de Microbiologia, Universidad San Francisco de Quito	Georgia Institute of Technology	2014-06-10	Esmeraldas, Ecuador	feces	Homo sapiens	Surveillance	0.96 N 79.65 W		100	0.23	pass	E_coli_ASM584v2	212	5263822	50.63	65385	pass	97.3046	pass	0
SAMN09833033	SRR7693820	PRJNA486009	*Escherichia coli*	Instituto de Microbiologia, Universidad San Francisco de Quito	Georgia Institute of Technology	2014-08-19	Esmeraldas, Ecuador	stool	Homo sapiens	Surveillance	0.96 N 79.65 W		100	0.21	pass	E_coli_ASM584v2	134	5423675	50.52	127176	pass	97.225	pass	0
SAMN09833092	SRR7693745	PRJNA514245	*Escherichia coli*	Instituto de Microbiologia, Universidad San Francisco de Quito	Georgia Institute of Technology	2014-09-19	Quito, Ecuador	feces	Homo sapiens	Surveillance	0.18 S 78.46 W		100	0.21	pass	E_coli_ASM584v2	145	4960175	50.62	187247	pass	98.3642	pass	0

ID	SRA Run	Bioproject	Organism	Recollected	Submmiter	Collection date	Geo loc name	Isolation source	Host	Purpose of sampling	Lat ion	Hospital	Completeness_General	Contamination	1st Criterion	FastANI Reference	# contigs	Total length	GC (%)	N50	2nd Criterion	FastANI (%)	3rd Criterion	Failed criteria
SAMN09833026	SRR7693846	PRJNA486009	*Escherichia coli*	Instituto de Microbiologia, Universidad San Francisco de Quito	Georgia Institute of Technology	2014-07-29	Esmeraldas, Ecuador	feces	Homo sapiens	Surveillance	0.96 N 79.65 W		100	0.41	pass	E_coli_ASM584v2	294	5589096	50.39	73604	pass	97.2569	pass	0
SAMN09833147	SRR7693649	PRJNA514245	*Escherichia coli*	Instituto de Microbiologia, Universidad San Francisco de Quito	Georgia Institute of Technology	2015-06-18	Santo domingo, Ecuador	feces	Homo sapiens	Surveillance	0.25 S 79.17 W		100	0.07	pass	E_coli_ASM584v2	229	5137296	50.63	58793	pass	98.3104	pass	0
SAMN09832905	SRR7693832	PRJNA486009	*Escherichia coli*	Instituto de Microbiologia, Universidad San Francisco de Quito	Georgia Institute of Technology	2015-02-11	Maldonado, Ecuador	stool	Homo sapiens	Surveillance	1.06 N 78.91 W		100	0.15	pass	E_coli_ASM584v2	128	5281580	50.59	111883	pass	97.2659	pass	0
SAMN09833048	SRR7693602	PRJNA486009	*Escherichia coli*	Instituto de Microbiologia, Universidad San Francisco de Quito	Georgia Institute of Technology	2014-06-02	Esmeraldas, Ecuador	feces	Homo sapiens	Surveillance	0.96 N 79.65 W		100	0.04	pass	E_coli_ASM584v2	378	4803979	50.82	27578	fail	99.1198	pass	1
SAMN09832906	SRR7693835	PRJNA486009	*Escherichia coli*	Instituto de Microbiologia, Universidad San Francisco de Quito	Georgia Institute of Technology	2015-03-27	Borbon, Ecuador	stool	Homo sapiens	Surveillance	1.08 N 78.99 W		97.41	0.08	pass	E_coli_ASM584v2	243	5092560	50.67	54286	pass	99.4402	pass	0
SAMN09833011	SRR7693806	PRJNA486009	*Escherichia coli*	Instituto de Microbiologia, Universidad San Francisco de Quito	Georgia Institute of Technology	2014-04-29	Esmeraldas, Ecuador	stool	Homo sapiens	Surveillance	0.96 N 79.65 W		100	0.64	pass	E_coli_ASM584v2	312	5441556	50.33	62695	pass	97.2417	pass	0
SAMN09833142	SRR7693644	PRJNA514245	*Escherichia coli*	Instituto de Microbiologia, Universidad San Francisco de Quito	Georgia Institute of Technology	2015-02-21	Ecuador	feces	Homo sapiens	Surveillance	1.83 S 78.18 W		100	0.11	pass	E_coli_ASM584v2	94	4805286	50.65	153690	pass	99.5094	pass	0
SAMN09833091	SRR7693746	PRJNA514245	*Escherichia coli*	Instituto de Microbiologia, Universidad San Francisco de Quito	Georgia Institute of Technology	2014-05-15	Quito, Ecuador	feces	Homo sapiens	Surveillance	0.18 S 78.46 W		100	0.18	pass	E_coli_ASM584v2	146	4820038	50.59	134842	pass	98.2998	pass	0
SAMN09833134	SRR7693660	PRJNA486009	*Escherichia coli*	Instituto de Microbiologia, Universidad San Francisco de Quito	Georgia Institute of Technology	2015-09-25	Ecuador	feces	Homo sapiens	Surveillance	1.83 S 78.18 W		100	0.47	pass	E_coli_ASM584v2	257	4932458	50.99	44216	pass	98.8219	pass	0
SAMN09832927	SRR7693774	PRJNA486009	*Escherichia coli*	Instituto de Microbiologia, Universidad San Francisco de Quito	Georgia Institute of Technology	missing	Borbon, Ecuador	stool	Homo sapiens	Surveillance	1.08 N 78.99 W		100	0.17	pass	E_coli_ASM584v2	124	4894906	50.54	166365	pass	98.3494	pass	0
SAMN09833066	SRR7693701	PRJNA514245	*Escherichia coli*	Instituto de Microbiologia, Universidad San Francisco de Quito	Georgia Institute of Technology	2014-06-17	Esmeraldas, Ecuador	feces	Homo sapiens	Surveillance	0.96 N 79.65 W		100	0.12	pass	E_coli_ASM584v2	510	5380613	50.71	28441	fail	97.2429	pass	1
SAMN09832902	SRR7693831	PRJNA486009	*Escherichia coli*	Instituto de Microbiologia, Universidad San Francisco de Quito	Georgia Institute of Technology	2015-01-21	Borbon, Ecuador	stool	Homo sapiens	Surveillance	1.08 N 78.99 W		100	0.45	pass	E_coli_ASM584v2	342	4784155	50.67	32989	fail	98.2957	pass	1
SAMN09833150	SRR7693727	PRJNA514245	*Escherichia coli*	Instituto de Microbiologia, Universidad San Francisco de Quito	Georgia Institute of Technology	2015-06-18	Santo domingo, Ecuador	feces	Homo sapiens	Surveillance	0.25 S 79.17 W		100	0.05	pass	E_coli_ASM584v2	109	4717220	50.62	117329	pass	99.4543	pass	0

ID	SRA Run	Bioproject	Organism	Recollected	Submmiter	Collection date	Geo loc name	Isolation source	Host	Purpose of sampling	Lat ion	Hospital	Completeness_General	Contamination	1st Criterion	FastANI Reference	# contigs	Total length	GC (%)	N50	2nd Criterion	FastANI (%)	3rd Criterion	Failed criteria
SAMN09833027	SRR7693845	PRJNA486009	*Escherichia coli*	Instituto de Microbiologia, Universidad San Francisco de Quito	Georgia Institute of Technology	2014-04-30	Esmeraldas, Ecuador	feces	Homo sapiens	Surveillance	0.96 N 79.65 W		100	0.14	pass	E_coli_ASM584v2	175	5052434	50.6	78425	pass	99.156	pass	0
SAMN09833130	SRR7693626	PRJNA486009	*Escherichia coli*	Instituto de Microbiologia, Universidad San Francisco de Quito	Georgia Institute of Technology	2014-10-15	Ecuador	feces	Homo sapiens	Surveillance	1.83 S 78.18 W		100	0.31	pass	E_coli_ASM584v2	235	5138664	50.65	114310	pass	98.4111	pass	0
SAMN09833087	SRR7693714	PRJNA514245	*Escherichia coli*	Instituto de Microbiologia, Universidad San Francisco de Quito	Georgia Institute of Technology	2014-09-18	Quito, Ecuador	feces	Homo sapiens	Surveillance	0.18 S 78.46 W		100	0.12	pass	E_coli_ASM584v2	211	5047709	50.52	104455	pass	98.3735	pass	0
SAMN09832936	SRR7693725	PRJNA486009	*Escherichia coli*	Instituto de Microbiologia, Universidad San Francisco de Quito	Georgia Institute of Technology	2015-09-05	Borbon, ecuador	feces	Homo sapiens	Surveillance	1.08 N 78.99 W		99.86	0.01	pass	E_coli_ASM584v2	355	4910133	50.59	37753	fail	99.147	pass	1
SAMN09833068	SRR7693697	PRJNA514245	*Escherichia coli*	Instituto de Microbiologia, Universidad San Francisco de Quito	Georgia Institute of Technology	2014-09-04	Quito, Ecuador	feces	Homo sapiens	Surveillance	0.18 S 78.46 W		100	0	pass	E_coli_ASM584v2	139	4927557	50.55	85562	pass	97.2439	pass	0
SAMN09833054	SRR7693667	PRJNA486009	*Escherichia coli*	Instituto de Microbiologia, Universidad San Francisco de Quito	Georgia Institute of Technology	missing	Esmeraldas, Ecuador	feces	Homo sapiens	Surveillance	0.96 N 79.65 W		100	0.78	pass	E_coli_ASM584v2	166	5189338	50.61	131190	pass	96.7731	pass	0
SAMN09833086	SRR7693715	PRJNA514245	*Escherichia coli*	Instituto de Microbiologia, Universidad San Francisco de Quito	Georgia Institute of Technology	2014-09-18	Quito, Ecuador	feces	Homo sapiens	Surveillance	0.18 S 78.46 W		97.5	0.92	pass	E_coli_ASM584v2	305	4925784	50.71	38169	fail	98.786	pass	1
SAMN09833007	SRR7693778	PRJNA486009	*Escherichia coli*	Instituto de Microbiologia, Universidad San Francisco de Quito	Georgia Institute of Technology	2014-07-01	Esmeraldas, Ecuador	stool	Homo sapiens	Surveillance	0.96 N 79.65 W		100	0.08	pass	E_coli_ASM584v2	88	4518530	50.63	102845	pass	99.5524	pass	0
SAMN09833013	SRR7693804	PRJNA486009	*Escherichia coli*	Instituto de Microbiologia, Universidad San Francisco de Quito	Georgia Institute of Technology	2014-07-16	Esmeraldas, Ecuador	stool	Homo sapiens	Surveillance	0.96 N 79.65 W		100	0.02	pass	E_coli_ASM584v2	163	5060275	50.73	118928	pass	99.4793	pass	0
SAMN09833071	SRR7693674	PRJNA514245	*Escherichia coli*	Instituto de Microbiologia, Universidad San Francisco de Quito	Georgia Institute of Technology	2014-09-03	Quito, Ecuador	feces	Homo sapiens	Surveillance	0.18 S 78.46 W		100	0.35	pass	E_coli_ASM584v2	276	5195367	50.39	71305	pass	99.4026	pass	0
SAMN09832914	SRR7693792	PRJNA486009	*Escherichia coli*	Instituto de Microbiologia, Universidad San Francisco de Quito	Georgia Institute of Technology	2015-05-15	Borbon, Ecuador	Stool	Homo sapiens	Surveillance	1.08 N 78.99 W		99.88	0.12	pass	E_coli_ASM584v2	151	4983960	50.64	85837	pass	99.4848	pass	0
SAMN09833143	SRR7693645	PRJNA514245	*Escherichia coli*	Instituto de Microbiologia, Universidad San Francisco de Quito	Georgia Institute of Technology	2015-04-17	Selva Alegre, Ecuador	feces	Homo sapiens	Surveillance	0.33 N 78.19 W		100	0.11	pass	E_coli_ASM584v2	215	5089671	50.71	65791	pass	99.4595	pass	0
SAMN09833127	SRR7693827	PRJNA486009	*Escherichia coli*	Instituto de Microbiologia, Universidad San Francisco de Quito	Georgia Institute of Technology	2015-08-15	Ecuador	feces	Homo sapiens	Surveillance	1.83 S 78.18 W		100	0.15	pass	E_coli_ASM584v2	197	4883740	50.69	62988	pass	98.3733	pass	0

ID	SRA Run	Bioproject	Organism	Recollected	Submmiter	Collection date	Geo loc name	Isolation source	Host	Purpose of sampling	Lat ion	Hospital	Completeness_General	Contamination	1st Criterion	FastANI Reference	# contigs	Total length	GC (%)	N50	2nd Criterion	FastANI (%)	3rd Criterion	Failed criteria
SAMN09833004	SRR7693781	PRJNA486009	*Escherichia coli*	Instituto de Microbiologia, Universidad San Francisco de Quito	Georgia Institute of Technology	2014-06-27	Esmeraldas, Ecuador	Stool	Homo sapiens	Surveillance	0.96 N 79.65 W		100	0.23	pass	E_coli_ASM584v2	129	4982191	50.73	122943	pass	99.0854	pass	0
SAMN09833089	SRR7693712	PRJNA514245	*Escherichia coli*	Instituto de Microbiologia, Universidad San Francisco de Quito	Georgia Institute of Technology	2014-09-23	Quito, Ecuador	feces	Homo sapiens	Surveillance	0.18 S 78.46 W		98.9	0	pass	E_coli_ASM584v2	112	4763846	50.52	131647	pass	98.3102	pass	0
SAMN09833152	SRR7693735	PRJNA514245	*Escherichia coli*	Instituto de Microbiologia, Universidad San Francisco de Quito	Georgia Institute of Technology	2015-07-17	Selva Alegre, Ecuador	feces	Homo sapiens	Surveillance	0.33 N 78.19 W		100	0.11	pass	E_coli_ASM584v2	119	5201318	50.62	119087	pass	98.3489	pass	0
SAMN09833140	SRR7693789	PRJNA514245	*Escherichia coli*	Instituto de Microbiologia, Universidad San Francisco de Quito	Georgia Institute of Technology	missing	Ecuador	feces	Homo sapiens	Surveillance	1.83 S 78.18 W		100	0.11	pass	E_coli_ASM584v2	99	4921552	50.69	133312	pass	98.3102	pass	0
SAMN09832904	SRR7693833	PRJNA486009	*Escherichia coli*	Instituto de Microbiologia, Universidad San Francisco de Quito	Georgia Institute of Technology	2015-01-26	Borbon, Ecuador	Stool	Homo sapiens	Surveillance	1.08 N 78.99 W		98.75	0	pass	E_coli_ASM584v2	258	5232544	50.49	48296	pass	97.2445	pass	0
SAMN09833067	SRR7693700	PRJNA514245	*Escherichia coli*	Instituto de Microbiologia, Universidad San Francisco de Quito	Georgia Institute of Technology	2014-06-17	Esmeraldas, Ecuador	feces	Homo sapiens	Surveillance	0.96 N 79.65 W		100	0.73	pass	E_coli_ASM584v2	162	5060245	50.9	108003	pass	96.8041	pass	0
SAMN09832915	SRR7693793	PRJNA486009	*Escherichia coli*	Instituto de Microbiologia, Universidad San Francisco de Quito	Georgia Institute of Technology	2015-05-20	Borbon, Ecuador	Stool	Homo sapiens	Surveillance	1.08 N 78.99 W		100	0.33	pass	E_coli_ASM584v2	195	4893957	50.57	103173	pass	98.3418	pass	0
SAMN09832939	SRR7693724	PRJNA486009	*Escherichia coli*	Instituto de Microbiologia, Universidad San Francisco de Quito	Georgia Institute of Technology	2015-09-28	Borbon, ecuador	feces	Homo sapiens	Surveillance	1.08 N 78.99 W		99.07	0.1	pass	E_coli_ASM584v2	283	4709904	50.75	37977	fail	99.4529	pass	1
SAMN09832973	SRR7693764	PRJNA486009	*Escherichia coli*	Instituto de Microbiologia, Universidad San Francisco de Quito	Georgia Institute of Technology	2015-02-27	Ecuador	Stool	Homo sapiens	Surveillance	1.8312 S 78.1834 W		100	0.42	pass	E_coli_ASM584v2	399	4804778	50.76	25702	fail	98.4068	pass	1
SAMN09833000	SRR7693785	PRJNA486009	*Escherichia coli*	Instituto de Microbiologia, Universidad San Francisco de Quito	Georgia Institute of Technology	2014-06-24	Esmeraldas, Ecuador	Stool	Homo sapiens	Surveillance	0.96 N 79.65 W		100	0.63	pass	E_coli_ASM584v2	176	5308870	50.39	107999	pass	99.3773	pass	0
SAMN09833104	SRR7693706	PRJNA514245	*Escherichia coli*	Instituto de Microbiologia, Universidad San Francisco de Quito	Georgia Institute of Technology	2014-09-24	Quito, Ecuador	feces	Homo sapiens	Surveillance	0.18 S 78.46 W		100	0.6	pass	E_coli_ASM584v2	336	4904573	50.88	32650	fail	99.162	pass	1
SAMN09832982	SRR7693825	PRJNA486009	*Escherichia coli*	Instituto de Microbiologia, Universidad San Francisco de Quito	Georgia Institute of Technology	2015-06-27	Ecuador	Stool	Homo sapiens	Surveillance	1.8312 S 78.1834 W		100	0.1	pass	E_coli_ASM584v2	108	4850593	50.51	169902	pass	96.7786	pass	0
SAMN09833037	SRR7693816	PRJNA486009	*Escherichia coli*	Instituto de Microbiologia, Universidad San Francisco de Quito	Georgia Institute of Technology	2014-09-04	Esmeraldas, Ecuador	Stool	Homo sapiens	Surveillance	0.96 N 79.65 W		98.88	1.06	pass	E_coli_ASM584v2	270	5020600	50.76	40542	pass	96.7343	pass	0

ID	SRA Run	Bioproject	Organism	Recollected	Submmiter	Collection date	Geo loc name	Isolation source	Host	Purpose of sampling	Lat ion	Hospital	Completeness_General	Contamination	1st Criterion	FastANI Reference	# contigs	Total length	GC (%)	N50	2nd Criterion	FastANI (%)	3rd Criterion	Failed criteria
SAMN09832993	SRR7693739	PRJNA486009	*Escherichia coli*	Instituto de Microbiologia\, Universidad San Francisco de Quito	Georgia Institute of Technology	2014-06-17	Esmeraldas, Ecuador	feces	Homo sapiens	Surveillance	0.96 N 79.65 W		100	0.4	pass	E_coli_ASM584v2	204	5062724	50.8	86809	pass	99.0981	pass	0
SAMN09832980	SRS3669932	PRJNA486009	*Escherichia coli*	Instituto de Microbiologia\, Universidad San Francisco de Quito	Georgia Institute of Technology	2015-06-06	Ecuador	feces	Homo sapiens	Surveillance	1.83 S 78.18 W		100	0.05	pass	E_coli_ASM584v2	224	4853818	50.62	52729	pass	99.0718	pass	0
SAMN09832981	SRS3669985	PRJNA486009	*Escherichia coli*	Instituto de Microbiologia\, Universidad San Francisco de Quito	Georgia Institute of Technology	2015-06-09	Ecuador	feces	Homo sapiens	Surveillance	1.83 S 78.18 W		100	0.17	pass	E_coli_ASM584v2	115	5061763	50.46	122545	pass	97.124	pass	0
SAMN09832964	SRS3669951	PRJNA486009	*Escherichia coli*	Instituto de Microbiologia\, Universidad San Francisco de Quito	Georgia Institute of Technology	2015-08-22	Ecuador	feces	Homo sapiens	Surveillance	1.83 S 78.18 W		100	0.16	pass	E_coli_ASM584v2	302	4890745	50.66	49811	pass	99.2025	pass	0
SAMN09832965	SRS3669952	PRJNA486009	*Escherichia coli*	Instituto de Microbiologia\, Universidad San Francisco de Quito	Georgia Institute of Technology	2015-09-11	Ecuador	feces	Homo sapiens	Surveillance	1.83 S 78.18 W		99.82	0.54	pass	E_coli_ASM584v2	120	4873544	50.54	185292	pass	96.676	pass	0
SAMN09832895	SRR7693762	PRJNA486009	*Escherichia coli*	Instituto de Microbiologia\, Universidad San Francisco de Quito	Georgia Institute of Technology	2014-12-03	Colon Eloy, Ecuador	feces	Homo sapiens	Surveillance	0.73 N 79.06 W		97.07	5.21	fail	E_coli_ASM584v2	1248	5362307	50.44	7407	fail	99.3298	pass	2
SAMN09832940	SRS3669896	PRJNA486009	*Escherichia coli*	Instituto de Microbiologia\, Universidad San Francisco de Quito	Georgia Institute of Technology	2015-10-01	Borbon, Ecuador	feces	Homo sapiens	Surveillance	1.08 N 78.99 W		100	0.03	pass	E_coli_ASM584v2	182	5148606	50.71	96386	pass	99.4546	pass	0
SAMN09832995	SRR7693741	PRJNA486009	*Escherichia coli*	Instituto de Microbiologia\, Universidad San Francisco de Quito	Georgia Institute of Technology	2014-06-20	Esmeraldas, Ecuador	feces	Homo sapiens	Surveillance	0.96 N 79.65 W		100	0.02	pass	E_coli_ASM584v2	180	5101306	50.73	95505	pass	99.4604	pass	0
SAMN09832892	SRR7693757	PRJNA486009	*Escherichia coli*	Instituto de Microbiologia\, Universidad San Francisco de Quito	Georgia Institute of Technology	2014-10-10	Borbon, Ecuador	feces	Homo sapiens	Surveillance	1.08 N 78.99 W		100	0.73	pass	E_coli_ASM584v2	159	5314028	50.68	121128	pass	97.3898	pass	0
SAMN09832972	SRS3669987	PRJNA486009	*Escherichia coli*	Instituto de Microbiologia\, Universidad San Francisco de Quito	Georgia Institute of Technology	2015-02-27	Ecuador	feces	Homo sapiens	Surveillance			97.78	0.02	pass	E_coli_ASM584v2	140	4713591	50.67	93470	pass	99.56	pass	0
SAMN09832931	SRR7693732	PRJNA486009	*Escherichia coli*	Instituto de Microbiologia\, Universidad San Francisco de Quito	Georgia Institute of Technology	2015-07-09	Borbon, Ecuador	feces	Homo sapiens	Surveillance	1.08 N 78.99 W		100	0.49	pass	E_coli_ASM584v2	348	4806706	50.55	27329	fail	99.1721	pass	1
SAMN09832962	SRS3669949	PRJNA486009	*Escherichia coli*	Instituto de Microbiologia\, Universidad San Francisco de Quito	Georgia Institute of Technology	2014-10-10	Borbon, Ecuador	feces	Homo sapiens	Surveillance	1.08 N 78.99 W		100	0.25	pass	E_coli_ASM584v2	250	5143564	50.54	69695	pass	98.318	pass	0
SAMN09832891	SRR7693758	PRJNA486009	*Escherichia coli*	Instituto de Microbiologia\, Universidad San Francisco de Quito	Georgia Institute of Technology	2014-10-10	Borbon, Ecuador	feces	Homo sapiens	Surveillance	1.08 N 78.99 W		100	1.44	pass	E_coli_ASM584v2	81	5171219	50.77	216248	pass	96.7497	pass	0

ID	SRA Run	Bioproject	Organism	Recollected	Submmiter	Collection date	Geo loc name	Isolation source	Host	Purpose of sampling	Lat ion	Hospital	Completeness_General	Contamination	1st Criterion	FastANI Reference	# contigs	Total length	GC (%)	N50	2nd Criterion	FastANI (%)	3rd Criterion	Failed criteria
SAMN09832999	SRR7693734	PRJNA486009	*Escherichia coli*	Instituto de Microbiologia\, Universidad San Francisco de Quito	Georgia Institute of Technology	2014-06-24	Borbon, Ecuador	feces	Homo sapiens	Surveillance	1.08 N 78.99 W		100	0.04	pass	E_coli_ASM584v2	129	5108467	50.68	147073	pass	97.3213	pass	0
SAMN09832941	SRS3669895	PRJNA486009	*Escherichia coli*	Instituto de Microbiologia\, Universidad San Francisco de Quito	Georgia Institute of Technology	2014-06-15	Borbon, Ecuador	feces	Homo sapiens	Surveillance	1.08 N 78.99 W		99.97	0.56	pass	E_coli_ASM584v2	187	5268568	50.48	99769	pass	99.4524	pass	0
SAMN09832956	SRR7693695	PRJNA486009	*Escherichia coli*	Instituto de Microbiologia\, Universidad San Francisco de Quito	Georgia Institute of Technology	2014-09-24	Borbon, Ecuador	feces	Homo sapiens	Surveillance	1.08 N 78.99 W		100	0.26	pass	E_coli_ASM584v2	253	5509390	50.5	120237	pass	97.1688	pass	0
SAMN09832986	SRS3669838	PRJNA486009	*Escherichia coli*	Instituto de Microbiologia\, Universidad San Francisco de Quito	Georgia Institute of Technology	2015-07-27	Ecuador	feces	Homo sapiens	Surveillance	1.83 S 78.18 W		100	0.07	pass	E_coli_ASM584v2	112	4792454	50.76	123742	pass	99.4114	pass	0
SAMN09832983	SRS3669833	PRJNA486009	*Escherichia coli*	Instituto de Microbiologia\, Universidad San Francisco de Quito	Georgia Institute of Technology	2015-06-27	Ecuador	feces	Homo sapiens	Surveillance	1.83 S 78.18 W		100	0.32	pass	E_coli_ASM584v2	241	5330272	50.42	107307	pass	97.274	pass	0
SAMN09832937	SRR7693726	PRJNA486009	*Escherichia coli*	Instituto de Microbiologia\, Universidad San Francisco de Quito	Georgia Institute of Technology	2015-09-05	Borbon, Ecuador	feces	Homo sapiens	Surveillance	1.08 N 78.99 W		100	0.19	pass	E_coli_ASM584v2	123	4616643	50.82	68693	pass	98.4111	pass	0
SAMN09832949	SRS3669901	PRJNA486009	*Escherichia coli*	Instituto de Microbiologia\, Universidad San Francisco de Quito	Georgia Institute of Technology	2014-07-01	Colon Eloy, Ecuador	feces	Homo sapiens	Surveillance	0.73 N 79.06 W		100	0.14	pass	E_coli_ASM584v2	245	4805673	50.65	49408	pass	99.1393	pass	0
SAMN09832899	SRS3669983	PRJNA486009	*Escherichia coli*	Instituto de Microbiologia\, Universidad San Francisco de Quito	Georgia Institute of Technology	2014-12-10	Colon Eloy, Ecuador	feces	Homo sapiens	Surveillance	0.73 N 79.06 W		100	0.27	pass	E_coli_ASM584v2	113	4586168	50.72	94467	pass	98.9109	pass	0
SAMN30414715	SRR21141464	PRJNA861272	*Escherichia coli*	Jay Graham Laboratory	University of California - Berkeley	2018	Quito, Ecuador	feces	Homo sapiens	Surveillance			100	0.06	pass	E_coli_ASM584v2	230	4906656	50.89	57810	pass	98.4487	pass	0
SAMN30416340	SRR21143494	PRJNA861272	*Escherichia coli*	Jay Graham Laboratory	University of California - Berkeley	2021	Quito, Ecuador	feces	Homo sapiens	Surveillance			100	0.12	pass	E_coli_ASM584v2	175	4711858	50.82	57999	pass	98.4352	pass	0
SAMN30416357	SRR21143414	PRJNA861272	*Escherichia coli*	Jay Graham Laboratory	University of California - Berkeley	2021	Quito, Ecuador	feces	Homo sapiens	Surveillance			100	1.08	pass	E_coli_ASM584v2	145	5085389	50.93	95026	pass	96.785	pass	0
SAMN30414062	SRR21141421	PRJNA861272	*Escherichia coli*	Jay Graham Laboratory	University of California - Berkeley	2021	Quito, Ecuador	feces	Homo sapiens	Surveillance			100	0.03	pass	E_coli_ASM584v2	318	5390782	50.6	53621	pass	97.2977	pass	0
SAMN30414773	SRR21142255	PRJNA861272	*Escherichia coli*	Jay Graham Laboratory	University of California - Berkeley	2018	Quito, Ecuador	feces	Homo sapiens	Surveillance			100	0.07	pass	E_coli_ASM584v2	181	5403646	50.43	180300	pass	97.1863	pass	0
SAMN30413808	SRR21141834	PRJNA861272	*Escherichia coli*	Jay Graham Laboratory	University of California - Berkeley	2021	Quito, Ecuador	feces	Homo sapiens	Surveillance			100	0.02	pass	E_coli_ASM584v2	222	4524458	50.84	39870	fail	97.9438	pass	1
SAMN30413989	SRR21141821	PRJNA861272	*Escherichia coli*	Jay Graham Laboratory	University of California - Berkeley	2021	Quito, Ecuador	feces	Homo sapiens	Surveillance			100	0.05	pass	E_coli_ASM584v2	137	5071546	50.58	138431	pass	98.408	pass	0
SAMN30413806	SRR21141440	PRJNA861272	*Escherichia coli*	Jay Graham Laboratory	University of California - Berkeley	2021	Quito, Ecuador	feces	Homo sapiens	Surveillance			99	0.76	pass	E_coli_ASM584v2	75	4700027	55.62	151154	pass	81.6563	fail	1
SAMN30414724	SRR21141870	PRJNA861272	*Escherichia coli*	Jay Graham Laboratory	University of California - Berkeley	2018	Quito, Ecuador	feces	Homo sapiens	Surveillance			100	0.15	pass	E_coli_ASM584v2	120	4990883	50.63	160658	pass	98.4784	pass	0

ID	SRA Run	Bioproject	Organism	Recollected	Submmiter	Collection date	Geo loc name	Isolation source	Host	Purpose of sampling	Lat ion	Hospital	Completeness_General	Contamination	1st Criterion	FastANI Reference	# contigs	Total length	GC (%)	N50	2nd Criterion	FastANI (%)	3rd Criterion	Failed criteria
SAMN30416121	SRR21143646	PRJNA861272	*Escherichia coli*	Jay Graham Laboratory	University of California - Berkeley	2021	Quito, Ecuador	feces	Homo sapiens	Surveillance			100	0.24	pass	E_coli_ASM584v2	310	5427764	50.9	51235	pass	97.3092	pass	0
SAMN30413959	SRR21141439	PRJNA861272	*Escherichia coli*	Jay Graham Laboratory	University of California - Berkeley	2021	Quito, Ecuador	feces	Homo sapiens	Surveillance			100	0.33	pass	E_coli_ASM584v2	185	5259060	50.5	166146	pass	97.2021	pass	0
SAMN30416471	SRR21143710	PRJNA861272	*Escherichia coli*	Jay Graham Laboratory	University of California - Berkeley	2021	Quito, Ecuador	feces	Homo sapiens	Surveillance			100	0.36	pass	E_coli_ASM584v2	207	5226287	50.54	88573	pass	97.269	pass	0
SAMN30414780	SRR21142247	PRJNA861272	*Escherichia coli*	Jay Graham Laboratory	University of California - Berkeley	2018	Quito, Ecuador	feces	Homo sapiens	Surveillance			100	0.57	pass	E_coli_ASM584v2	140	5262151	50.54	174660	pass	97.8592	pass	0
SAMN30413814	SRR21141639	PRJNA861272	*Escherichia coli*	Jay Graham Laboratory	University of California - Berkeley	2021	Quito, Ecuador	feces	Homo sapiens	Surveillance			100	0.53	pass	E_coli_ASM584v2	259	4825253	51.07	39089	fail	99.3898	pass	1
SAMN30414437	SRR21142372	PRJNA861272	*Escherichia coli*	Jay Graham Laboratory	University of California - Berkeley	2021	Quito, Ecuador	feces	Homo sapiens	Surveillance			100	0.21	pass	E_coli_ASM584v2	252	4909246	50.83	52173	pass	98.8565	pass	0
SAMN30414642	SRR21142304	PRJNA861272	*Escherichia coli*	Jay Graham Laboratory	University of California - Berkeley	2018	Quito, Ecuador	feces	Homo sapiens	Surveillance			100	0.04	pass	E_coli_ASM584v2	101	5142676	50.6	153711	pass	97.2244	pass	0
SAMN30414169	SRR21141590	PRJNA861272	*Escherichia coli*	Jay Graham Laboratory	University of California - Berkeley	2021	Quito, Ecuador	feces	Homo sapiens	Surveillance			100	0.08	pass	E_coli_ASM584v2	149	4721829	50.83	74136	pass	98.9355	pass	0
SAMN30414158	SRR21141603	PRJNA861272	*Escherichia coli*	Jay Graham Laboratory	University of California - Berkeley	2021	Quito, Ecuador	feces	Homo sapiens	Surveillance			100	0.24	pass	E_coli_ASM584v2	357	5374909	50.89	41820	pass	99.0082	pass	0
SAMN30414334	SRR21141727	PRJNA861272	*Escherichia coli*	Jay Graham Laboratory	University of California - Berkeley	2021	Quito, Ecuador	feces	Homo sapiens	Surveillance			100	0.01	pass	E_coli_ASM584v2	311	4631376	51.11	29066	fail	98.974	pass	1
SAMN30414425	SRR21141978	PRJNA861272	*Escherichia coli*	Jay Graham Laboratory	University of California - Berkeley	2021	Quito, Ecuador	feces	Homo sapiens	Surveillance			100	0.14	pass	E_coli_ASM584v2	216	5025405	50.8	78617	pass	97.2088	pass	0
SAMN30414338	SRR21141723	PRJNA861272	*Escherichia coli*	Jay Graham Laboratory	University of California - Berkeley	2021	Quito, Ecuador	feces	Homo sapiens	Surveillance			100	0.9	pass	E_coli_ASM584v2	243	5365255	50.54	50907	pass	97.1902	pass	0
SAMN30414549	SRR21141936	PRJNA861272	*Escherichia coli*	Jay Graham Laboratory	University of California - Berkeley	2021	Quito, Ecuador	feces	Homo sapiens	Surveillance			100	1.87	pass	E_coli_ASM584v2	228	5336118	50.62	47647	pass	97.8984	pass	0
SAMN30414073	SRR21141409	PRJNA861272	*Escherichia coli*	Jay Graham Laboratory	University of California - Berkeley	2021	Quito, Ecuador	feces	Homo sapiens	Surveillance			100	0.48	pass	E_coli_ASM584v2	255	4957764	50.5	43132	pass	98.4341	pass	0
SAMN30414627	SRR21141882	PRJNA861272	*Escherichia coli*	Jay Graham Laboratory	University of California - Berkeley	2018	Quito, Ecuador	feces	Homo sapiens	Surveillance			100	0.04	pass	E_coli_ASM584v2	94	4772134	50.76	136542	pass	99.5445	pass	0
SAMN30413997	SRR21141813	PRJNA861272	*Escherichia coli*	Jay Graham Laboratory	University of California - Berkeley	2021	Quito, Ecuador	feces	Homo sapiens	Surveillance			100	0.16	pass	E_coli_ASM584v2	89	4949608	50.62	146184	pass	98.3024	pass	0
SAMN30414409	SRR21141995	PRJNA861272	*Escherichia coli*	Jay Graham Laboratory	University of California - Berkeley	2021	Quito, Ecuador	feces	Homo sapiens	Surveillance			100	0.17	pass	E_coli_ASM584v2	148	4801140	50.57	79267	pass	99.5907	pass	0
SAMN30414670	SRR21142241	PRJNA861272	*Escherichia coli*	Jay Graham Laboratory	University of California - Berkeley	2018	Quito, Ecuador	feces	Homo sapiens	Surveillance			100	0.96	pass	E_coli_ASM584v2	268	5147207	50.78	62089	pass	97.0621	pass	0
SAMN30416428	SRR21143366	PRJNA861272	*Escherichia coli*	Jay Graham Laboratory	University of California - Berkeley	2021	Quito, Ecuador	feces	Homo sapiens	Surveillance			100	0.18	pass	E_coli_ASM584v2	102	5051494	50.47	147496	pass	97.1009	pass	0
SAMN30414135	SRR21142156	PRJNA861272	*Escherichia coli*	Jay Graham Laboratory	University of California - Berkeley	2021	Quito, Ecuador	feces	Homo sapiens	Surveillance			100	0.02	pass	E_coli_ASM584v2	89	4794230	50.61	136295	pass	98.3809	pass	0
SAMN30416398	SRR21143791	PRJNA861272	*Escherichia coli*	Jay Graham Laboratory	University of California - Berkeley	2021	Quito, Ecuador	feces	Homo sapiens	Surveillance			99.73	0	pass	E_coli_ASM584v2	166	5205573	50.67	100399	pass	98.5129	pass	0
SAMN30414145	SRR21142145	PRJNA514245	*Escherichia coli*	Jay Graham Laboratory	University of California - Berkeley	2021	Quito, Ecuador	feces	Homo sapiens	Surveillance			100	0.22	pass	E_coli_ASM584v2	294	5115675	50.67	50400	pass	97.1253	pass	0
SAMN30414370	SRR21142087	PRJNA861272	*Escherichia coli*	Jay Graham Laboratory	University of California - Berkeley	2021	Quito, Ecuador	feces	Homo sapiens	Surveillance			100	0.09	pass	E_coli_ASM584v2	176	4734992	50.74	57174	pass	99.1584	pass	0
SAMN30414011	SRR21142197	PRJNA861272	*Escherichia coli*	Jay Graham Laboratory	University of California - Berkeley	2021	Quito, Ecuador	feces	Homo sapiens	Surveillance			100	0.26	pass	E_coli_ASM584v2	102	4934707	50.74	117478	pass	98.4309	pass	0

ID	SRA Run	Bioproject	Organism	Recollected	Submitter	Collection date	Geo loc name	Isolation source	Host	Purpose of sampling	Lat ion	Hospital	Completeness_General	Contamination	1st Criterion	FastANI Reference	# contigs	Total length	GC (%)	N50	2nd Criterion	FastANI (%)	3rd Criterion	Failed criteria
SAMN30413894	SRR21141703	PRJNA861272	*Escherichia coli*	Jay Graham Laboratory	University of California - Berkeley	2021	Quito, Ecuador	feces	Homo sapiens	Surveillance			100	0.1	pass	E_coli_ASM584v2	106	4846986	50.72	138172	pass	99.4055	pass	0
SAMN30413982	SRR21141830	PRJNA861272	*Escherichia coli*	Jay Graham Laboratory	University of California - Berkeley	2021	Quito, Ecuador	feces	Homo sapiens	Surveillance			100	0.06	pass	E_coli_ASM584v2	142	4746826	50.92	72662	pass	98.4909	pass	0
SAMN30413917	SRR21141678	PRJNA861272	*Escherichia coli*	Jay Graham Laboratory	University of California - Berkeley	2021	Quito, Ecuador	feces	Homo sapiens	Surveillance			100	0.11	pass	E_coli_ASM584v2	63	4832613	50.51	287816	pass	97.2161	pass	0
SAMN30413795	SRR21141539	PRJNA861272	*Escherichia coli*	Jay Graham Laboratory	University of California - Berkeley	2021	Quito, Ecuador	feces	Homo sapiens	Surveillance			100	0.13	pass	E_coli_ASM584v2	87	5068156	50.65	198179	pass	98.341	pass	0
SAMN30414781	SRR21142214	PRJNA861272	*Escherichia coli*	Jay Graham Laboratory	University of California - Berkeley	2018	Quito, Ecuador	feces	Homo sapiens	Surveillance			100	1.38	pass	E_coli_ASM584v2	120	5280792	50.55	228742	pass	97.8619	pass	0
SAMN30414767	SRR21142262	PRJNA861272	*Escherichia coli*	Jay Graham Laboratory	University of California - Berkeley	2018	Quito, Ecuador	feces	Homo sapiens	Surveillance			100	0.37	pass	E_coli_ASM584v2	165	4985382	50.56	119022	pass	99.0644	pass	0
SAMN30414626	SRR21141883	PRJNA861272	*Escherichia coli*	Jay Graham Laboratory	University of California - Berkeley	2018	Quito, Ecuador	feces	Homo sapiens	Surveillance			100	0.2	pass	E_coli_ASM584v2	211	4967532	50.65	68928	pass	98.4222	pass	0
SAMN30414226	SRR21141751	PRJNA861272	*Escherichia coli*	Jay Graham Laboratory	University of California - Berkeley	2021	Quito, Ecuador	feces	Homo sapiens	Surveillance			99.84	0.06	pass	E_coli_ASM584v2	253	4925728	50.74	43469	pass	98.4129	pass	0
SAMN30416421	SRR21143375	PRJNA861272	*Escherichia coli*	Jay Graham Laboratory	University of California - Berkeley	2021	Quito, Ecuador	feces	Homo sapiens	Surveillance			85.63	2.84	fail	E_coli_ASM584v2	2531	3958020	49.89	1905	fail	97.0019	pass	2
SAMN30414089	SRR21141806	PRJNA861272	*Escherichia coli*	Jay Graham Laboratory	University of California - Berkeley	2021	Quito, Ecuador	feces	Homo sapiens	Surveillance			100	0.06	pass	E_coli_ASM584v2	185	4933583	50.7	114517	pass	98.4276	pass	0
SAMN30414590	SRR21142329	PRJNA861272	*Escherichia coli*	Jay Graham Laboratory	University of California - Berkeley	2018	Quito, Ecuador	feces	Homo sapiens	Surveillance			95.74	0.08	pass	E_coli_ASM584v2	539	4707011	50.6	15417	fail	98.2328	pass	1
SAMN30416443	SRR21143741	PRJNA861272	*Escherichia coli*	Jay Graham Laboratory	University of California - Berkeley	2021	Quito, Ecuador	feces	Homo sapiens	Surveillance			100	0.05	pass	E_coli_ASM584v2	197	5037280	50.55	83557	pass	98.3309	pass	0
SAMN30413942	SRR21141650	PRJNA861272	*Escherichia coli*	Jay Graham Laboratory	University of California - Berkeley	2021	Quito, Ecuador	feces	Homo sapiens	Surveillance			100	0.11	pass	E_coli_ASM584v2	270	5162104	50.72	42440	pass	97.1983	pass	0
SAMN30416375	SRR21143394	PRJNA861272	*Escherichia coli*	Jay Graham Laboratory	University of California - Berkeley	2021	Quito, Ecuador	feces	Homo sapiens	Surveillance			100	0.29	pass	E_coli_ASM584v2	286	5580920	50.75	55498	pass	97.3013	pass	0
SAMN30414196	SRR21142040	PRJNA861272	*Escherichia coli*	Jay Graham Laboratory	University of California - Berkeley	2021	Quito, Ecuador	feces	Homo sapiens	Surveillance			100	1.27	pass	E_coli_ASM584v2	300	5213784	50.79	36773	fail	97.8829	pass	1
SAMN30414373	SRR21142084	PRJNA514245	*Escherichia coli*	Jay Graham Laboratory	University of California - Berkeley	2021	Quito, Ecuador	feces	Homo sapiens	Surveillance			99.29	0.73	pass	E_coli_ASM584v2	518	4718271	51.08	17622	fail	98.5072	pass	1
SAMN30414668	SRR21142244	PRJNA861272	*Escherichia coli*	Jay Graham Laboratory	University of California - Berkeley	2018	Quito, Ecuador	feces	Homo sapiens	Surveillance			100	0.52	pass	E_coli_ASM584v2	148	5093329	50.52	158422	pass	98.4689	pass	0
SAMN30416385	SRR21143806	PRJNA861272	*Escherichia coli*	Jay Graham Laboratory	University of California - Berkeley	2021	Quito, Ecuador	feces	Homo sapiens	Surveillance			100	0.08	pass	E_coli_ASM584v2	156	4775082	50.82	78621	pass	99.4497	pass	0
SAMN30416086	SRR21143916	PRJNA861272	*Escherichia coli*	Jay Graham Laboratory	University of California - Berkeley	2021	Quito, Ecuador	feces	Homo sapiens	Surveillance			100	0.79	pass	E_coli_ASM584v2	213	4923029	50.56	48001	pass	98.4521	pass	0
SAMN30414641	SRR21142305	PRJNA861272	*Escherichia coli*	Jay Graham Laboratory	University of California - Berkeley	2018	Quito, Ecuador	feces	Homo sapiens	Surveillance			100	0.09	pass	E_coli_ASM584v2	97	5139848	50.59	207371	pass	97.2146	pass	0
SAMN30414765	SRR21142264	PRJNA861272	*Escherichia coli*	Jay Graham Laboratory	University of California - Berkeley	2018	Quito, Ecuador	feces	Homo sapiens	Surveillance			100	0.73	pass	E_coli_ASM584v2	168	5305206	50.59	148485	pass	97.2852	pass	0
SAMN30414347	SRR21141713	PRJNA861272	*Escherichia coli*	Jay Graham Laboratory	University of California - Berkeley	2021	Quito, Ecuador	feces	Homo sapiens	Surveillance			100	1.59	pass	E_coli_ASM584v2	201	5155214	50.67	63320	pass	97.9088	pass	0
SAMN30414276	SRR21141568	PRJNA861272	*Escherichia coli*	Jay Graham Laboratory	University of California - Berkeley	2021	Quito, Ecuador	feces	Homo sapiens	Surveillance			100	0.14	pass	E_coli_ASM584v2	323	4763703	51.02	26221	fail	98.9008	pass	1
SAMN30414264	SRR21141581	PRJNA861272	*Escherichia coli*	Jay Graham Laboratory	University of California - Berkeley	2021	Quito, Ecuador	feces	Homo sapiens	Surveillance			100	0.61	pass	E_coli_ASM584v2	288	5340732	50.64	54131	pass	97.2994	pass	0

ID	SRA Run	Bioproject	Organism	Recollected	Submmiter	Collection date	Geo loc name	Isolation source	Host	Purpose of sampling	Lat ion	Hospital	Completeness_General	Contamination	1st Criterion	FastANI Reference	# contigs	Total length	GC (%)	N50	2nd Criterion	FastANI (%)	3rd Criterion	Failed criteria
SAMN30414543	SRR21141943	PRJNA861272	*Escherichia coli*	Jay Graham Laboratory	University of California - Berkeley	2021	Quito, Ecuador	feces	Homo sapiens	Surveillance			100	0.15	pass	E_coli_ASM584v2	192	4690217	50.95	52764	pass	98.9262	pass	0
SAMN30414775	SRR21142253	PRJNA861272	*Escherichia coli*	Jay Graham Laboratory	University of California - Berkeley	2018	Quito, Ecuador	feces	Homo sapiens	Surveillance			100	0.07	pass	E_coli_ASM584v2	167	5426247	50.44	174276	pass	97.1939	pass	0
SAMN30413957	SRR21141442	PRJNA861272	*Escherichia coli*	Jay Graham Laboratory	University of California - Berkeley	2021	Quito, Ecuador	feces	Homo sapiens	Surveillance			98.37	0.07	pass	E_coli_ASM584v2	80	4884801	50.38	184703	pass	98.3233	pass	0
SAMN30413837	SRR21141576	PRJNA861272	*Escherichia coli*	Jay Graham Laboratory	University of California - Berkeley	2021	Quito, Ecuador	feces	Homo sapiens	Surveillance			100	0.14	pass	E_coli_ASM584v2	156	5093154	50.68	71921	pass	98.3022	pass	0
SAMN30414350	SRR21142109	PRJNA861272	*Escherichia coli*	Jay Graham Laboratory	University of California - Berkeley	2021	Quito, Ecuador	feces	Homo sapiens	Surveillance			100	0.12	pass	E_coli_ASM584v2	369	4897037	50.94	25295	fail	98.4308	pass	1
SAMN30414327	SRR21141735	PRJNA861272	*Escherichia coli*	Jay Graham Laboratory	University of California - Berkeley	2021	Quito, Ecuador	feces	Homo sapiens	Surveillance			100	0.26	pass	E_coli_ASM584v2	307	5013048	50.85	33880	fail	97.277	pass	1
SAMN30414297	SRR21142024	PRJNA861272	*Escherichia coli*	Jay Graham Laboratory	University of California - Berkeley	2021	Quito, Ecuador	feces	Homo sapiens	Surveillance			98.34	0.01	pass	E_coli_ASM584v2	302	4717179	50.71	30495	fail	99.6978	pass	2
SAMN30416410	SRR21143778	PRJNA861272	*Escherichia coli*	Jay Graham Laboratory	University of California - Berkeley	2021	Quito, Ecuador	feces	Homo sapiens	Surveillance			100	1.87	pass	E_coli_ASM584v2	158	5279084	50.75	190648	pass	96.7639	pass	0
SAMN30414031	SRR21141647	PRJNA861272	*Escherichia coli*	Jay Graham Laboratory	University of California - Berkeley	2021	Quito, Ecuador	feces	Homo sapiens	Surveillance			100	0.21	pass	E_coli_ASM584v2	213	4754937	50.8	57951	pass	99.2653	pass	0
SAMN30414436	SRR21142373	PRJNA861272	*Escherichia coli*	Jay Graham Laboratory	University of California - Berkeley	2021	Quito, Ecuador	feces	Homo sapiens	Surveillance			99.85	0.17	pass	E_coli_ASM584v2	260	4874377	50.78	42886	pass	98.8447	pass	0
SAMN30416083	SRR21143919	PRJNA861272	*Escherichia coli*	Jay Graham Laboratory	University of California - Berkeley	2021	Quito, Ecuador	feces	Homo sapiens	Surveillance			99.96	0.03	pass	E_coli_ASM584v2	211	4821161	50.78	47277	pass	99.0154	pass	0
SAMN30413803	SRR21141665	PRJNA861272	*Escherichia coli*	Jay Graham Laboratory	University of California - Berkeley	2021	Quito, Ecuador	feces	Homo sapiens	Surveillance			100	0.3	pass	E_coli_ASM584v2	146	5003184	50.73	102046	pass	98.4005	pass	0
SAMN30416382	SRR21143386	PRJNA861272	*Escherichia coli*	Jay Graham Laboratory	University of California - Berkeley	2021	Quito, Ecuador	feces	Homo sapiens	Surveillance			100	0.94	pass	E_coli_ASM584v2	245	5641439	50.69	56762	pass	98.0075	pass	0
SAMN30413973	SRR21141424	PRJNA861272	*Escherichia coli*	Jay Graham Laboratory	University of California - Berkeley	2021	Quito, Ecuador	feces	Homo sapiens	Surveillance			100	0.54	pass	E_coli_ASM584v2	222	5241094	50.61	80218	pass	98.8421	pass	0
SAMN30415831	SRR21143509	PRJNA861272	*Escherichia coli*	Jay Graham Laboratory	University of California - Berkeley	2018	Quito, Ecuador	feces	Homo sapiens	Surveillance			100	0.14	pass	E_coli_ASM584v2	225	5189357	50.58	117183	pass	97.1417	pass	0
SAMN30414162	SRR21141598	PRJNA861272	*Escherichia coli*	Jay Graham Laboratory	University of California - Berkeley	2021	Quito, Ecuador	feces	Homo sapiens	Surveillance			100	0.17	pass	E_coli_ASM584v2	220	4906820	50.87	47846	pass	99.0413	pass	0
SAMN30416452	SRR21143731	PRJNA861272	*Escherichia coli*	Jay Graham Laboratory	University of California - Berkeley	2021	Quito, Ecuador	feces	Homo sapiens	Surveillance			100	0.06	pass	E_coli_ASM584v2	82	4884031	50.66	211016	pass	98.4637	pass	0
SAMN30414199	SRR21142036	PRJNA861272	*Escherichia coli*	Jay Graham Laboratory	University of California - Berkeley	2021	Quito, Ecuador	feces	Homo sapiens	Surveillance			99.76	0.08	pass	E_coli_ASM584v2	332	5241414	50.75	35091	fail	99.2866	pass	1
SAMN30416446	SRR21143738	PRJNA861272	*Escherichia coli*	Jay Graham Laboratory	University of California - Berkeley	2021	Quito, Ecuador	feces	Homo sapiens	Surveillance			100	0.12	pass	E_coli_ASM584v2	138	5000418	50.7	86499	pass	98.3454	pass	0
SAMN30413927	SRR21141667	PRJNA861272	*Escherichia coli*	Jay Graham Laboratory	University of California - Berkeley	2021	Quito, Ecuador	feces	Homo sapiens	Surveillance			100	0.14	pass	E_coli_ASM584v2	223	4814796	50.79	58298	pass	99.4127	pass	0
SAMN30414053	SRR21141623	PRJNA861272	*Escherichia coli*	Jay Graham Laboratory	University of California - Berkeley	2021	Quito, Ecuador	feces	Homo sapiens	Surveillance			100	0.21	pass	E_coli_ASM584v2	79	5104240	50.7	221455	pass	97.2059	pass	0
SAMN30414283	SRR21141560	PRJNA861272	*Escherichia coli*	Jay Graham Laboratory	University of California - Berkeley	2021	Quito, Ecuador	feces	Homo sapiens	Surveillance			100	0.05	pass	E_coli_ASM584v2	218	4864147	50.61	49344	pass	98.882	pass	0
SAMN30416441	SRR21143743	PRJNA861272	*Escherichia coli*	Jay Graham Laboratory	University of California - Berkeley	2021	Quito, Ecuador	feces	Homo sapiens	Surveillance			100	0	pass	E_coli_ASM584v2	137	5065693	50.96	88398	pass	99.4301	pass	0
SAMN30414337	SRR21141724	PRJNA861272	*Escherichia coli*	Jay Graham Laboratory	University of California - Berkeley	2021	Quito, Ecuador	feces	Homo sapiens	Surveillance			98.24	0.37	pass	E_coli_ASM584v2	376	5292044	50.42	39449	fail	97.2768	pass	1

ID	SRA Run	Bioproject	Organism	Recollected	Submmiter	Collection date	Geo loc name	Isolation source	Host	Purpose of sampling	Lat ion	Hospital	Completeness_General	Contamination	1st Criterion	FastANI Reference	# contigs	Total length	GC (%)	N50	2nd Criterion	FastANI (%)	3rd Criterion	Failed criteria
SAMN30414286	SRR21141557	PRJNA861272	*Escherichia coli*	Jay Graham Laboratory	University of California - Berkeley	2021	Quito, Ecuador	feces	Homo sapiens	Surveillance			100	0.16	pass	E_coli_ASM584v2	202	5466925	50.49	175180	pass	97.1353	pass	0
SAMN30415832	SRR21143368	PRJNA861272	*Escherichia coli*	Jay Graham Laboratory	University of California - Berkeley	2018	Quito, Ecuador	feces	Homo sapiens	Surveillance			100	0.14	pass	E_coli_ASM584v2	233	5241556	50.58	114509	pass	97.1259	pass	0
SAMN30413893	SRR21141704	PRJNA861272	*Escherichia coli*	Jay Graham Laboratory	University of California - Berkeley	2021	Quito, Ecuador	feces	Homo sapiens	Surveillance			100	0.11	pass	E_coli_ASM584v2	97	4981676	50.54	139012	pass	98.3908	pass	0
SAMN30413988	SRR21141824	PRJNA861272	*Escherichia coli*	Jay Graham Laboratory	University of California - Berkeley	2021	Quito, Ecuador	feces	Homo sapiens	Surveillance			100	0.07	pass	E_coli_ASM584v2	299	5131538	50.76	53785	pass	99.4828	pass	0
SAMN30414550	SRR21141935	PRJNA861272	*Escherichia coli*	Jay Graham Laboratory	University of California - Berkeley	2021	Quito, Ecuador	feces	Homo sapiens	Surveillance			100	0.63	pass	E_coli_ASM584v2	205	5063430	50.93	56152	pass	97.2652	pass	0
SAMN30414090	SRR21141805	PRJNA861272	*Escherichia coli*	Jay Graham Laboratory	University of California - Berkeley	2021	Quito, Ecuador	feces	Homo sapiens	Surveillance			100	0.16	pass	E_coli_ASM584v2	385	5244079	50.64	38064	fail	97.2336	pass	1
SAMN30415861	SRR21143465	PRJNA861272	*Escherichia coli*	Jay Graham Laboratory	University of California - Berkeley	2018	Quito, Ecuador	feces	Homo sapiens	Surveillance			100	0.2	pass	E_coli_ASM584v2	177	4897821	50.42	94262	pass	99.4242	pass	0
SAMN30416330	SRR21143505	PRJNA861272	*Escherichia coli*	Jay Graham Laboratory	University of California - Berkeley	2021	Quito, Ecuador	feces	Homo sapiens	Surveillance			98.79	0.04	pass	E_coli_ASM584v2	337	4883602	50.79	33804	fail	97.3144	pass	1
SAMN30416377	SRR21143392	PRJNA861272	*Escherichia coli*	Jay Graham Laboratory	University of California - Berkeley	2021	Quito, Ecuador	feces	Homo sapiens	Surveillance			100	0.27	pass	E_coli_ASM584v2	112	4861198	50.5	120858	pass	98.3569	pass	0
SAMN30416467	SRR21143715	PRJNA861272	*Escherichia coli*	Jay Graham Laboratory	University of California - Berkeley	2021	Quito, Ecuador	feces	Homo sapiens	Surveillance			100	0.31	pass	E_coli_ASM584v2	86	4787038	50.65	123917	pass	99.0011	pass	0
SAMN30414352	SRR21142107	PRJNA514245	*Escherichia coli*	Jay Graham Laboratory	University of California - Berkeley	2021	Quito, Ecuador	feces	Homo sapiens	Surveillance			100	0.03	pass	E_coli_ASM584v2	333	4530888	51.11	24835	fail	99.0487	pass	1
SAMN30414329	SRR21141732	PRJNA861272	*Escherichia coli*	Jay Graham Laboratory	University of California - Berkeley	2021	Quito, Ecuador	feces	Homo sapiens	Surveillance			100	0.19	pass	E_coli_ASM584v2	249	5105563	50.65	64249	pass	97.3151	pass	0
SAMN30416369	SRR21143400	PRJNA861272	*Escherichia coli*	Jay Graham Laboratory	University of California - Berkeley	2021	Quito, Ecuador	feces	Homo sapiens	Surveillance			100	0.05	pass	E_coli_ASM584v2	170	4909242	50.62	105603	pass	99.4599	pass	0
SAMN30414210	SRR21141768	PRJNA861272	*Escherichia coli*	Jay Graham Laboratory	University of California - Berkeley	2021	Quito, Ecuador	feces	Homo sapiens	Surveillance			100	0.09	pass	E_coli_ASM584v2	185	4989280	51.02	83337	pass	98.4359	pass	0
SAMN30413993	SRR21141817	PRJNA861272	*Escherichia coli*	Jay Graham Laboratory	University of California - Berkeley	2021	Quito, Ecuador	feces	Homo sapiens	Surveillance			100	0.25	pass	E_coli_ASM584v2	208	5010124	50.56	54545	pass	97.1762	pass	0
SAMN30414386	SRR21141542	PRJNA861272	*Escherichia coli*	Jay Graham Laboratory	University of California - Berkeley	2021	Quito, Ecuador	feces	Homo sapiens	Surveillance			100	0.17	pass	E_coli_ASM584v2	301	4803625	50.86	34746	fail	98.9252	pass	1
SAMN30413898	SRR21141699	PRJNA861272	*Escherichia coli*	Jay Graham Laboratory	University of California - Berkeley	2021	Quito, Ecuador	feces	Homo sapiens	Surveillance			99.01	0.03	pass	E_coli_ASM584v2	244	4920292	50.78	61863	pass	97.1664	pass	0
SAMN30414583	SRR21142338	PRJNA861272	*Escherichia coli*	Jay Graham Laboratory	University of California - Berkeley	2018	Quito, Ecuador	feces	Homo sapiens	Surveillance			100	0.22	pass	E_coli_ASM584v2	248	4930466	50.52	84830	pass	98.376	pass	0
SAMN30414376	SRR21142081	PRJNA514245	*Escherichia coli*	Jay Graham Laboratory	University of California - Berkeley	2021	Quito, Ecuador	feces	Homo sapiens	Surveillance			100	0.16	pass	E_coli_ASM584v2	161	4928742	50.76	65719	pass	97.9238	pass	0
SAMN30414402	SRR21141523	PRJNA861272	*Escherichia coli*	Jay Graham Laboratory	University of California - Berkeley	2021	Quito, Ecuador	feces	Homo sapiens	Surveillance			100	0.51	pass	E_coli_ASM584v2	143	4900542	50.49	94038	pass	98.4352	pass	0
SAMN30414173	SRR21141586	PRJNA861272	*Escherichia coli*	Jay Graham Laboratory	University of California - Berkeley	2021	Quito, Ecuador	feces	Homo sapiens	Surveillance			100	0.04	pass	E_coli_ASM584v2	201	4886262	50.75	57586	pass	98.3793	pass	0
SAMN30414340	SRR21141720	PRJNA861272	*Escherichia coli*	Jay Graham Laboratory	University of California - Berkeley	2021	Quito, Ecuador	feces	Homo sapiens	Surveillance			100	0.17	pass	E_coli_ASM584v2	358	4897625	50.95	31094	fail	98.3407	pass	1
SAMN30413879	SRR21142220	PRJNA861272	*Escherichia coli*	Jay Graham Laboratory	University of California - Berkeley	2021	Quito, Ecuador	feces	Homo sapiens	Surveillance			100	0.12	pass	E_coli_ASM584v2	154	5268872	50.61	111429	pass	98.329	pass	0
SAMN30414480	SRR21142061	PRJNA514245	*Escherichia coli*	Jay Graham Laboratory	University of California - Berkeley	2021	Quito, Ecuador	feces	Homo sapiens	Surveillance			100	0	pass	E_coli_ASM584v2	141	4756923	50.77	73145	pass	99.564	pass	0

ID	SRA Run	Bioproject	Organism	Recollected	Submitter	Collection date	Geo loc name	Isolation source	Host	Purpose of sampling	Lat ion	Hospital	Completeness_General	Contamination	1st Criterion	FastANI Reference	# contigs	Total length	GC (%)	N50	2nd Criterion	FastANI (%)	3rd Criterion	Failed criteria
SAMN30414539	SRR21141947	PRJNA861272	*Escherichia coli*	Jay Graham Laboratory	University of California - Berkeley	2021	Quito, Ecuador	feces	Homo sapiens	Surveillance			98.3	0.01	pass	E_coli_ASM584v2	213	4488749	50.86	44107	pass	99.3879	pass	0
SAMN30413789	SRR21141711	PRJNA861272	*Escherichia coli*	Jay Graham Laboratory	University of California - Berkeley	2021	Quito, Ecuador	feces	Homo sapiens	Surveillance			93.22	8.24	fail	E_coli_ASM584v2	845	5259829	50.76	17584	fail	98.4876	pass	2
SAMN30414049	SRR21141627	PRJNA861272	*Escherichia coli*	Jay Graham Laboratory	University of California - Berkeley	2021	Quito, Ecuador	feces	Homo sapiens	Surveillance			100	0.03	pass	E_coli_ASM584v2	100	5128294	50.37	128159	pass	98.3203	pass	0
SAMN30414201	SRR21142034	PRJNA861272	*Escherichia coli*	Jay Graham Laboratory	University of California - Berkeley	2021	Quito, Ecuador	feces	Homo sapiens	Surveillance			100	0.48	pass	E_coli_ASM584v2	171	5219930	50.69	117855	pass	97.2837	pass	0
SAMN30414774	SRR21142254	PRJNA861272	*Escherichia coli*	Jay Graham Laboratory	University of California - Berkeley	2018	Quito, Ecuador	feces	Homo sapiens	Surveillance			99.96	0.09	pass	E_coli_ASM584v2	193	5422331	50.45	114709	pass	97.2278	pass	0
SAMN30414071	SRR21141411	PRJNA861272	*Escherichia coli*	Jay Graham Laboratory	University of California - Berkeley	2021	Quito, Ecuador	feces	Homo sapiens	Surveillance			100	0.08	pass	E_coli_ASM584v2	144	4916638	50.57	102829	pass	98.4019	pass	0
SAMN30413873	SRR21141880	PRJNA861272	*Escherichia coli*	Jay Graham Laboratory	University of California - Berkeley	2021	Quito, Ecuador	feces	Homo sapiens	Surveillance			100	0.24	pass	E_coli_ASM584v2	342	5498744	50.84	40049	pass	97.3157	pass	0
SAMN30416451	SRR21143732	PRJNA861272	*Escherichia coli*	Jay Graham Laboratory	University of California - Berkeley	2021	Quito, Ecuador	feces	Homo sapiens	Surveillance			100	0.34	pass	E_coli_ASM584v2	200	5603880	50.53	120108	pass	97.2361	pass	0
SAMN30414221	SRR21141756	PRJNA861272	*Escherichia coli*	Jay Graham Laboratory	University of California - Berkeley	2021	Quito, Ecuador	feces	Homo sapiens	Surveillance			100	0.2	pass	E_coli_ASM584v2	131	4862536	50.89	96918	pass	99.5691	pass	0
SAMN30414443	SRR21142365	PRJNA861272	*Escherichia coli*	Jay Graham Laboratory	University of California - Berkeley	2021	Quito, Ecuador	feces	Homo sapiens	Surveillance			100	0.13	pass	E_coli_ASM584v2	259	5046798	50.77	58015	pass	98.3694	pass	0
SAMN30414058	SRR21141618	PRJNA861272	*Escherichia coli*	Jay Graham Laboratory	University of California - Berkeley	2021	Quito, Ecuador	feces	Homo sapiens	Surveillance			100	0.19	pass	E_coli_ASM584v2	151	4753708	50.51	88426	pass	98.8972	pass	0
SAMN30414456	SRR21142351	PRJNA861272	*Escherichia coli*	Jay Graham Laboratory	University of California - Berkeley	2021	Quito, Ecuador	feces	Homo sapiens	Surveillance			100	0.11	pass	E_coli_ASM584v2	202	4798426	50.82	54546	pass	98.9699	pass	0
SAMN30414582	SRR21142339	PRJNA861272	*Escherichia coli*	Jay Graham Laboratory	University of California - Berkeley	2018	Quito, Ecuador	feces	Homo sapiens	Surveillance			100	0.06	pass	E_coli_ASM584v2	221	4972497	50.56	56361	pass	97.2646	pass	0
SAMN30414644	SRR21142302	PRJNA861272	*Escherichia coli*	Jay Graham Laboratory	University of California - Berkeley	2018	Quito, Ecuador	feces	Homo sapiens	Surveillance			98.66	0.04	pass	E_coli_ASM584v2	101	5090086	50.59	134461	pass	97.1417	pass	0
SAMN30414305	SRR21142015	PRJNA861272	*Escherichia coli*	Jay Graham Laboratory	University of California - Berkeley	2021	Quito, Ecuador	feces	Homo sapiens	Surveillance			100	0.17	pass	E_coli_ASM584v2	168	4771634	50.75	78619	pass	98.4163	pass	0
SAMN30413871	SRR21141902	PRJNA861272	*Escherichia coli*	Jay Graham Laboratory	University of California - Berkeley	2021	Quito, Ecuador	feces	Homo sapiens	Surveillance			100	0.02	pass	E_coli_ASM584v2	230	4933559	50.89	39621	fail	97.3753	pass	1
SAMN30414375	SRR21142082	PRJNA861272	*Escherichia coli*	Jay Graham Laboratory	University of California - Berkeley	2021	Quito, Ecuador	feces	Homo sapiens	Surveillance			100	0.39	pass	E_coli_ASM584v2	318	5237204	50.52	43992	pass	97.2177	pass	0
SAMN30413930	SRR21141663	PRJNA861272	*Escherichia coli*	Jay Graham Laboratory	University of California - Berkeley	2021	Quito, Ecuador	feces	Homo sapiens	Surveillance			100	0.31	pass	E_coli_ASM584v2	164	5258793	50.47	122692	pass	97.2586	pass	0
SAMN30414051	SRR21141625	PRJNA861272	*Escherichia coli*	Jay Graham Laboratory	University of California - Berkeley	2021	Quito, Ecuador	feces	Homo sapiens	Surveillance			100	0.04	pass	E_coli_ASM584v2	131	5004810	50.51	102555	pass	98.4624	pass	0
SAMN30413829	SRR21141376	PRJNA861272	*Escherichia coli*	Jay Graham Laboratory	University of California - Berkeley	2021	Quito, Ecuador	feces	Homo sapiens	Surveillance			98.16	0.01	pass	E_coli_ASM584v2	173	4830407	50.76	66540	pass	98.4009	pass	0
SAMN30413834	SRR21142137	PRJNA514245	*Escherichia coli*	Jay Graham Laboratory	University of California - Berkeley	2021	Quito, Ecuador	feces	Homo sapiens	Surveillance			100	0.01	pass	E_coli_ASM584v2	156	5188103	50.53	91297	pass	98.3764	pass	0
SAMN30414224	SRR21141753	PRJNA861272	*Escherichia coli*	Jay Graham Laboratory	University of California - Berkeley	2021	Quito, Ecuador	feces	Homo sapiens	Surveillance			100	0.12	pass	E_coli_ASM584v2	417	4892839	50.89	29974	fail	97.1813	pass	1
SAMN30414778	SRR21142250	PRJNA861272	*Escherichia coli*	Jay Graham Laboratory	University of California - Berkeley	2018	Quito, Ecuador	feces	Homo sapiens	Surveillance			100	0.66	pass	E_coli_ASM584v2	113	5274182	50.53	221545	pass	97.8344	pass	0
SAMN30416361	SRR21143409	PRJNA861272	*Escherichia coli*	Jay Graham Laboratory	University of California - Berkeley	2021	Quito, Ecuador	feces	Homo sapiens	Surveillance			100	0.11	pass	E_coli_ASM584v2	105	4928614	50.7	107917	pass	98.3794	pass	0

ID	SRA Run	Bioproject	Organism	Recollected	Submitter	Collection date	Geo loc name	Isolation source	Host	Purpose of sampling	Lat ion	Hospital	Completeness_General	Contamination	1st Criterion	FastANI Reference	# contigs	Total length	GC (%)	N50	2nd Criterion	FastANI (%)	3rd Criterion	Failed criteria
SAMN30413968	SRR21141430	PRJNA861272	*Escherichia coli*	Jay Graham Laboratory	University of California - Berkeley	2021	Quito, Ecuador	feces	Homo sapiens	Surveillance			100	0.06	pass	E_coli_ASM584v2	114	4862676	50.69	177319	pass	98.9087	pass	0
SAMN30416416	SRR21143381	PRJNA861272	*Escherichia coli*	Jay Graham Laboratory	University of California - Berkeley	2021	Quito, Ecuador	feces	Homo sapiens	Surveillance			97.79	44.52	fail	Acinetobacter_ASM903584v1	530	4558754	37.74	17658	fail	78.7185	fail	3
SAMN30414271	SRR21141573	PRJNA861272	*Escherichia coli*	Jay Graham Laboratory	University of California - Berkeley	2021	Quito, Ecuador	feces	Homo sapiens	Surveillance			100	0.09	pass	E_coli_ASM584v2	169	4985015	50.76	75731	pass	99.1082	pass	0
SAMN30414365	SRR21142093	PRJNA861272	*Escherichia coli*	Jay Graham Laboratory	University of California - Berkeley	2021	Quito, Ecuador	feces	Homo sapiens	Surveillance			99.41	0.06	pass	E_coli_ASM584v2	561	4978893	51.17	16986	fail	97.2583	pass	1
SAMN30416349	SRR21143484	PRJNA861272	*Escherichia coli*	Jay Graham Laboratory	University of California - Berkeley	2021	Quito, Ecuador	feces	Homo sapiens	Surveillance			100	0.41	pass	E_coli_ASM584v2	185	4739148	50.81	61347	pass	99.6315	pass	0
SAMN30414046	SRR21141631	PRJNA861272	*Escherichia coli*	Jay Graham Laboratory	University of California - Berkeley	2021	Quito, Ecuador	feces	Homo sapiens	Surveillance			100	0.16	pass	E_coli_ASM584v2	169	5215672	50.73	120807	pass	98.4006	pass	0
SAMN30414344	SRR21141716	PRJNA861272	*Escherichia coli*	Jay Graham Laboratory	University of California - Berkeley	2021	Quito, Ecuador	feces	Homo sapiens	Surveillance			99.95	0.05	pass	E_coli_ASM584v2	287	4706744	51.03	33014	fail	98.5259	pass	1
SAMN30413947	SRR21141453	PRJNA861272	*Escherichia coli*	Jay Graham Laboratory	University of California - Berkeley	2021	Quito, Ecuador	feces	Homo sapiens	Surveillance			100	0.23	pass	E_coli_ASM584v2	228	5132165	50.89	58930	pass	97.1178	pass	0
SAMN30413953	SRR21141446	PRJNA861272	*Escherichia coli*	Jay Graham Laboratory	University of California - Berkeley	2021	Quito, Ecuador	feces	Homo sapiens	Surveillance			100	0.03	pass	E_coli_ASM584v2	139	5091562	54.08	110206	pass	81.1248	fail	1
SAMN30416429	SRR21143365	PRJNA861272	*Escherichia coli*	Jay Graham Laboratory	University of California - Berkeley	2021	Quito, Ecuador	feces	Homo sapiens	Surveillance			100	0.32	pass	E_coli_ASM584v2	149	5140919	50.53	112210	pass	97.2354	pass	0
SAMN30414133	SRR21142158	PRJNA861272	*Escherichia coli*	Jay Graham Laboratory	University of California - Berkeley	2021	Quito, Ecuador	feces	Homo sapiens	Surveillance			99.6	0.11	pass	E_coli_ASM584v2	124	5046098	50.54	153599	pass	97.2497	pass	0
SAMN30413809	SRR21141822	PRJNA861272	*Escherichia coli*	Jay Graham Laboratory	University of California - Berkeley	2021	Quito, Ecuador	feces	Homo sapiens	Surveillance			100	0.06	pass	E_coli_ASM584v2	113	4672639	50.83	87397	pass	98.4745	pass	0
SAMN30414319	SRR21141743	PRJNA861272	*Escherichia coli*	Jay Graham Laboratory	University of California - Berkeley	2021	Quito, Ecuador	feces	Homo sapiens	Surveillance			100	0.19	pass	E_coli_ASM584v2	178	4916730	50.75	60045	pass	99.107	pass	0
SAMN30416466	SRR21143716	PRJNA861272	*Escherichia coli*	Jay Graham Laboratory	University of California - Berkeley	2021	Quito, Ecuador	feces	Homo sapiens	Surveillance			100	0.34	pass	E_coli_ASM584v2	163	5025472	50.6	105992	pass	99.0803	pass	0
SAMN30414427	SRR21141976	PRJNA861272	*Escherichia coli*	Jay Graham Laboratory	University of California - Berkeley	2021	Quito, Ecuador	feces	Homo sapiens	Surveillance			99.74	0.02	pass	E_coli_ASM584v2	254	4508353	50.93	40956	pass	99.3769	pass	0
SAMN30414723	SRR21141871	PRJNA861272	*Escherichia coli*	Jay Graham Laboratory	University of California - Berkeley	2018	Quito, Ecuador	feces	Homo sapiens	Surveillance			100	0.19	pass	E_coli_ASM584v2	104	4965208	50.61	197711	pass	98.4858	pass	0
SAMN30415839	SRR21143349	PRJNA861272	*Escherichia coli*	Jay Graham Laboratory	University of California - Berkeley	2018	Quito, Ecuador	feces	Homo sapiens	Surveillance			100	0.15	pass	E_coli_ASM584v2	109	4862697	50.63	126937	pass	98.9272	pass	0
SAMN30414078	SRR21141404	PRJNA861272	*Escherichia coli*	Jay Graham Laboratory	University of California - Berkeley	2021	Quito, Ecuador	feces	Homo sapiens	Surveillance			100	0.09	pass	E_coli_ASM584v2	197	5059937	50.79	73991	pass	97.1529	pass	0
SAMN30415830	SRR21143431	PRJNA861272	*Escherichia coli*	Jay Graham Laboratory	University of California - Berkeley	2018	Quito, Ecuador	feces	Homo sapiens	Surveillance			100	0.16	pass	E_coli_ASM584v2	153	4835629	50.63	90105	pass	98.9431	pass	0
SAMN30414066	SRR21141417	PRJNA861272	*Escherichia coli*	Jay Graham Laboratory	University of California - Berkeley	2021	Quito, Ecuador	feces	Homo sapiens	Surveillance			99.36	0.49	pass	E_coli_ASM584v2	295	5087845	50.84	35407	fail	96.7767	pass	1
SAMN30413905	SRR21141691	PRJNA861272	*Escherichia coli*	Jay Graham Laboratory	University of California - Berkeley	2021	Quito, Ecuador	feces	Homo sapiens	Surveillance			100	0.56	pass	E_coli_ASM584v2	177	4822568	50.97	49116	pass	98.0544	pass	0
SAMN30414660	SRR21142284	PRJNA861272	*Escherichia coli*	Jay Graham Laboratory	University of California - Berkeley	2018	Quito, Ecuador	feces	Homo sapiens	Surveillance			100	0.12	pass	E_coli_ASM584v2	86	5228242	50.49	213907	pass	97.3102	pass	0
SAMN30414421	SRR21141982	PRJNA861272	*Escherichia coli*	Jay Graham Laboratory	University of California - Berkeley	2021	Quito, Ecuador	feces	Homo sapiens	Surveillance			100	0.04	pass	E_coli_ASM584v2	413	4883521	51.06	25169	fail	98.2812	pass	1
SAMN30413798	SRR21142221	PRJNA861272	*Escherichia coli*	Jay Graham Laboratory	University of California - Berkeley	2021	Quito, Ecuador	feces	Homo sapiens	Surveillance			67.35	48.93	fail	E_coli_ASM584v2	1849	6245494	50.59	9584	fail	97.3076	pass	2

ID	SRA Run	Bioproject	Organism	Recollected	Submitter	Collection date	Geo loc name	Isolation source	Host	Purpose of sampling	Lat ion	Hospital	Completeness_General	Contamination	1st Criterion	FastANI Reference	# contigs	Total length	GC (%)	N50	2nd Criterion	FastANI (%)	3rd Criterion	Failed criteria
SAMN30414171	SRR21141588	PRJNA861272	*Escherichia coli*	Jay Graham Laboratory	University of California - Berkeley	2021	Quito, Ecuador	feces	Homo sapiens	Surveillance			100	0.04	pass	E_coli_ASM584v2	76	4638567	50.72	134777	pass	99.549	pass	0
SAMN30414719	SRR21141459	PRJNA861272	*Escherichia coli*	Jay Graham Laboratory	University of California - Berkeley	2018	Quito, Ecuador	feces	Homo sapiens	Surveillance			100	0.06	pass	E_coli_ASM584v2	146	4920726	50.83	111730	pass	98.4542	pass	0
SAMN30414446	SRR21142362	PRJNA861272	*Escherichia coli*	Jay Graham Laboratory	University of California - Berkeley	2021	Quito, Ecuador	feces	Homo sapiens	Surveillance			100	0.09	pass	E_coli_ASM584v2	156	4939736	50.81	76584	pass	98.4898	pass	0
SAMN30416348	SRR21143485	PRJNA861272	*Escherichia coli*	Jay Graham Laboratory	University of California - Berkeley	2021	Quito, Ecuador	feces	Homo sapiens	Surveillance			100	0.07	pass	E_coli_ASM584v2	277	4929487	50.82	46408	pass	98.4904	pass	0
SAMN30414075	SRR21141407	PRJNA861272	*Escherichia coli*	Jay Graham Laboratory	University of California - Berkeley	2021	Quito, Ecuador	feces	Homo sapiens	Surveillance			99.92	0.62	pass	E_coli_ASM584v2	545	5560995	50.87	22363	fail	97.2008	pass	1
SAMN30416415	SRR21143382	PRJNA861272	*Escherichia coli*	Jay Graham Laboratory	University of California - Berkeley	2021	Quito, Ecuador	feces	Homo sapiens	Surveillance			100	0.05	pass	E_coli_ASM584v2	176	5368889	50.42	141094	pass	97.1789	pass	0
SAMN30416419	SRR21143377	PRJNA861272	*Escherichia coli*	Jay Graham Laboratory	University of California - Berkeley	2021	Quito, Ecuador	feces	Homo sapiens	Surveillance			100	0.29	pass	E_coli_ASM584v2	221	5282809	50.53	50346	pass	98.3185	pass	0
SAMN30414366	SRR21142092	PRJNA861272	*Escherichia coli*	Jay Graham Laboratory	University of California - Berkeley	2021	Quito, Ecuador	feces	Homo sapiens	Surveillance			99.68	0.1	pass	E_coli_ASM584v2	479	5346721	51.02	22058	fail	97.2953	pass	1
SAMN30414374	SRR21142083	PRJNA861272	*Escherichia coli*	Jay Graham Laboratory	University of California - Berkeley	2021	Quito, Ecuador	feces	Homo sapiens	Surveillance			99.54	0.03	pass	E_coli_ASM584v2	217	5259731	50.61	68490	pass	97.1546	pass	0
SAMN30414431	SRR21141971	PRJNA861272	*Escherichia coli*	Jay Graham Laboratory	University of California - Berkeley	2021	Quito, Ecuador	feces	Homo sapiens	Surveillance			100	0.27	pass	E_coli_ASM584v2	231	5103365	50.75	61304	pass	98.5167	pass	0
SAMN30414411	SRR21141993	PRJNA861272	*Escherichia coli*	Jay Graham Laboratory	University of California - Berkeley	2021	Quito, Ecuador	feces	Homo sapiens	Surveillance			100	0.21	pass	E_coli_ASM584v2	158	4848907	51.02	56388	pass	98.4109	pass	0
SAMN30414105	SRR21141789	PRJNA861272	*Escherichia coli*	Jay Graham Laboratory	University of California - Berkeley	2021	Quito, Ecuador	feces	Homo sapiens	Surveillance			99.97	0.18	pass	E_coli_ASM584v2	182	4611177	50.77	58256	pass	99.3318	pass	0
SAMN30413822	SRR21142174	PRJNA861272	*Escherichia coli*	Jay Graham Laboratory	University of California - Berkeley	2021	Quito, Ecuador	feces	Homo sapiens	Surveillance			100	0.1	pass	E_coli_ASM584v2	154	4631425	50.88	53694	pass	99.1795	pass	0
SAMN30414000	SRR21141809	PRJNA861272	*Escherichia coli*	Jay Graham Laboratory	University of California - Berkeley	2021	Quito, Ecuador	feces	Homo sapiens	Surveillance			100	0.12	pass	E_coli_ASM584v2	258	5162037	50.86	51887	pass	97.1447	pass	0
SAMN30414088	SRR21141393	PRJNA861272	*Escherichia coli*	Jay Graham Laboratory	University of California - Berkeley	2021	Quito, Ecuador	feces	Homo sapiens	Surveillance			100	0.3	pass	E_coli_ASM584v2	302	5185328	50.83	37969	fail	97.3063	pass	1
SAMN30414313	SRR21142006	PRJNA861272	*Escherichia coli*	Jay Graham Laboratory	University of California - Berkeley	2021	Quito, Ecuador	feces	Homo sapiens	Surveillance			100	0.25	pass	E_coli_ASM584v2	160	4983553	50.54	73185	pass	98.3626	pass	0
SAMN30414060	SRR21141423	PRJNA861272	*Escherichia coli*	Jay Graham Laboratory	University of California - Berkeley	2021	Quito, Ecuador	feces	Homo sapiens	Surveillance			99.78	0.02	pass	E_coli_ASM584v2	73	4867887	52.01	132778	pass	82.9286	fail	1
SAMN30414464	SRR21142079	PRJNA514245	*Escherichia coli*	Jay Graham Laboratory	University of California - Berkeley	2021	Quito, Ecuador	feces	Homo sapiens	Surveillance			100	0.11	pass	E_coli_ASM584v2	206	4848212	50.7	62635	pass	98.9068	pass	0
SAMN30414292	SRR21142029	PRJNA861272	*Escherichia coli*	Jay Graham Laboratory	University of California - Berkeley	2021	Quito, Ecuador	feces	Homo sapiens	Surveillance			100	0.88	pass	E_coli_ASM584v2	325	5358602	50.64	32893	fail	98.024	pass	1
SAMN30416118	SRR21143649	PRJNA861272	*Escherichia coli*	Jay Graham Laboratory	University of California - Berkeley	2021	Quito, Ecuador	feces	Homo sapiens	Surveillance			100	0.05	pass	E_coli_ASM584v2	80	4919119	51.94	156185	pass	82.3533	fail	1
SAMN30414246	SRR21142129	PRJNA861272	*Escherichia coli*	Jay Graham Laboratory	University of California - Berkeley	2021	Quito, Ecuador	feces	Homo sapiens	Surveillance			100	0.43	pass	E_coli_ASM584v2	290	5436217	50.61	46324	pass	97.1715	pass	0
SAMN30413850	SRR21141527	PRJNA861272	*Escherichia coli*	Jay Graham Laboratory	University of California - Berkeley	2021	Quito, Ecuador	feces	Homo sapiens	Surveillance			100	0.14	pass	E_coli_ASM584v2	154	5091689	50.71	110260	pass	98.3764	pass	0
SAMN30416454	SRR21143729	PRJNA861272	*Escherichia coli*	Jay Graham Laboratory	University of California - Berkeley	2021	Quito, Ecuador	feces	Homo sapiens	Surveillance			100	0.65	pass	E_coli_ASM584v2	176	5516792	50.65	97523	pass	97.3811	pass	0
SAMN30413909	SRR21141686	PRJNA861272	*Escherichia coli*	Jay Graham Laboratory	University of California - Berkeley	2021	Quito, Ecuador	feces	Homo sapiens	Surveillance			99.99	0.08	pass	E_coli_ASM584v2	263	5400834	50.84	53529	pass	97.2079	pass	0

ID	SRA Run	Bioproject	Organism	Recollected	Submmiter	Collection date	Geo loc name	Isolation source	Host	Purpose of sampling	Lat ion	Hospital	Completeness_General	Contamination	1st Criterion	FastANI Reference	# contigs	Total length	GC (%)	N50	2nd Criterion	FastANI (%)	3rd Criterion	Failed criteria
SAMN30416388	SRR21143802	PRJNA861272	*Escherichia coli*	Jay Graham Laboratory	University of California - Berkeley	2021	Quito, Ecuador	feces	Homo sapiens	Surveillance			100	0.45	pass	E_coli_ASM584v2	151	5080651	50.63	88119	pass	98.3546	pass	0
SAMN30414175	SRR21141584	PRJNA861272	*Escherichia coli*	Jay Graham Laboratory	University of California - Berkeley	2021	Quito, Ecuador	feces	Homo sapiens	Surveillance			98.04	0.62	pass	E_coli_ASM584v2	345	4943617	50.7	31440	fail	97.3017	pass	1
SAMN30413999	SRR21141810	PRJNA861272	*Escherichia coli*	Jay Graham Laboratory	University of California - Berkeley	2021	Quito, Ecuador	feces	Homo sapiens	Surveillance			100	0.14	pass	E_coli_ASM584v2	130	4883821	50.73	133795	pass	98.4553	pass	0
SAMN30415864	SRR21143854	PRJNA861272	*Escherichia coli*	Jay Graham Laboratory	University of California - Berkeley	2018	Quito, Ecuador	feces	Homo sapiens	Surveillance			100	0.19	pass	E_coli_ASM584v2	147	4910018	50.4	145148	pass	99.4251	pass	0
SAMN30414343	SRR21141717	PRJNA861272	*Escherichia coli*	Jay Graham Laboratory	University of California - Berkeley	2021	Quito, Ecuador	feces	Homo sapiens	Surveillance			100	0.27	pass	E_coli_ASM584v2	260	4811532	50.74	38092	fail	98.4074	pass	1
SAMN30414290	SRR21142031	PRJNA861272	*Escherichia coli*	Jay Graham Laboratory	University of California - Berkeley	2021	Quito, Ecuador	feces	Homo sapiens	Surveillance			100	0.76	pass	E_coli_ASM584v2	165	4987048	50.86	62684	pass	96.7169	pass	0
SAMN30413963	SRR21141435	PRJNA861272	*Escherichia coli*	Jay Graham Laboratory	University of California - Berkeley	2021	Quito, Ecuador	feces	Homo sapiens	Surveillance			100	0.09	pass	E_coli_ASM584v2	398	4804918	51.12	29710	fail	98.9278	pass	1
SAMN30414183	SRR21141383	PRJNA861272	*Escherichia coli*	Jay Graham Laboratory	University of California - Berkeley	2021	Quito, Ecuador	feces	Homo sapiens	Surveillance			99.95	0.11	pass	E_coli_ASM584v2	238	4936430	50.84	56604	pass	98.4668	pass	0
SAMN30413881	SRR21141471	PRJNA861272	*Escherichia coli*	Jay Graham Laboratory	University of California - Berkeley	2021	Quito, Ecuador	feces	Homo sapiens	Surveillance			100	0	pass	E_coli_ASM584v2	154	4855267	50.73	100598	pass	99.6874	pass	0
SAMN30414084	SRR21141397	PRJNA861272	*Escherichia coli*	Jay Graham Laboratory	University of California - Berkeley	2021	Quito, Ecuador	feces	Homo sapiens	Surveillance			96.99	0	pass	E_coli_ASM584v2	214	4413366	50.86	43293	pass	99.2607	pass	0
SAMN30414362	SRR21142096	PRJNA861272	*Escherichia coli*	Jay Graham Laboratory	University of California - Berkeley	2021	Quito, Ecuador	feces	Homo sapiens	Surveillance			100	0.35	pass	E_coli_ASM584v2	386	5612155	50.69	42356	pass	97.2604	pass	0
SAMN30414777	SRR21142251	PRJNA861272	*Escherichia coli*	Jay Graham Laboratory	University of California - Berkeley	2018	Quito, Ecuador	feces	Homo sapiens	Surveillance			100	0.76	pass	E_coli_ASM584v2	118	5287126	50.54	221544	pass	97.8274	pass	0
SAMN30416090	SRR21143911	PRJNA861272	*Escherichia coli*	Jay Graham Laboratory	University of California - Berkeley	2021	Quito, Ecuador	feces	Homo sapiens	Surveillance			100	0.16	pass	E_coli_ASM584v2	164	5173491	50.49	68193	pass	98.3349	pass	0
SAMN30415833	SRR21143703	PRJNA861272	*Escherichia coli*	Jay Graham Laboratory	University of California - Berkeley	2018	Quito, Ecuador	feces	Homo sapiens	Surveillance			99.14	0.47	pass	E_coli_ASM584v2	295	5146234	50.57	34139	fail	97.0585	pass	1
SAMN30413960	SRR21141438	PRJNA861272	*Escherichia coli*	Jay Graham Laboratory	University of California - Berkeley	2021	Quito, Ecuador	feces	Homo sapiens	Surveillance			100	0.21	pass	E_coli_ASM584v2	189	4957683	50.73	88111	pass	98.4815	pass	0
SAMN30416420	SRR21143376	PRJNA861272	*Escherichia coli*	Jay Graham Laboratory	University of California - Berkeley	2021	Quito, Ecuador	feces	Homo sapiens	Surveillance			100	0.13	pass	E_coli_ASM584v2	205	5184843	50.69	72392	pass	98.3428	pass	0
SAMN30416401	SRR21143788	PRJNA861272	*Escherichia coli*	Jay Graham Laboratory	University of California - Berkeley	2021	Quito, Ecuador	feces	Homo sapiens	Surveillance			100	0.27	pass	E_coli_ASM584v2	170	5236436	50.66	96044	pass	97.2609	pass	0
SAMN30414280	SRR21141563	PRJNA861272	*Escherichia coli*	Jay Graham Laboratory	University of California - Berkeley	2021	Quito, Ecuador	feces	Homo sapiens	Surveillance			100	0.32	pass	E_coli_ASM584v2	216	5032016	50.52	43989	pass	98.3092	pass	0
SAMN30416112	SRR21143656	PRJNA861272	*Escherichia coli*	Jay Graham Laboratory	University of California - Berkeley	2021	Quito, Ecuador	feces	Homo sapiens	Surveillance			99.96	0.13	pass	E_coli_ASM584v2	246	5115641	50.5	59987	pass	98.5095	pass	0
SAMN30416469	SRR21143712	PRJNA861272	*Escherichia coli*	Jay Graham Laboratory	University of California - Berkeley	2021	Quito, Ecuador	feces	Homo sapiens	Surveillance			99.76	0.04	pass	E_coli_ASM584v2	61	5093335	51.96	204681	pass	82.9427	fail	1
SAMN30414262	SRR21141583	PRJNA861272	*Escherichia coli*	Jay Graham Laboratory	University of California - Berkeley	2021	Quito, Ecuador	feces	Homo sapiens	Surveillance			100	0.14	pass	E_coli_ASM584v2	280	4981313	50.74	44389	pass	98.3289	pass	0
SAMN30413987	SRR21141825	PRJNA861272	*Escherichia coli*	Jay Graham Laboratory	University of California - Berkeley	2021	Quito, Ecuador	feces	Homo sapiens	Surveillance			100	0.19	pass	E_coli_ASM584v2	201	4848608	50.73	100018	pass	99.273	pass	0
SAMN30413966	SRR21141432	PRJNA861272	*Escherichia coli*	Jay Graham Laboratory	University of California - Berkeley	2021	Quito, Ecuador	feces	Homo sapiens	Surveillance			100	0.5	pass	E_coli_ASM584v2	132	5107147	50.24	121431	pass	99.0182	pass	0
SAMN30414243	SRR21142132	PRJNA514245	*Escherichia coli*	Jay Graham Laboratory	University of California - Berkeley	2021	Quito, Ecuador	feces	Homo sapiens	Surveillance			99.61	0	pass	E_coli_ASM584v2	176	4609767	50.89	69230	pass	99.3502	pass	0

ID	SRA Run	Bioproject	Organism	Recollected	Submitter	Collection date	Geo loc name	Isolation source	Host	Purpose of sampling	Lat ion	Hospital	Completeness_General	Contamination	1st Criterion	FastANI Reference	# contigs	Total length	GC (%)	N50	2nd Criterion	FastANI (%)	3rd Criterion	Failed criteria
SAMN30414008	SRR21142201	PRJNA861272	*Escherichia coli*	Jay Graham Laboratory	University of California - Berkeley	2021	Quito, Ecuador	feces	Homo sapiens	Surveillance			100	0.11	pass	E_coli_ASM584v2	199	4777985	50.85	55633	pass	99.1397	pass	0
SAMN30415835	SRR21143681	PRJNA861272	*Escherichia coli*	Jay Graham Laboratory	University of California - Berkeley	2018	Quito, Ecuador	feces	Homo sapiens	Surveillance			100	0.15	pass	E_coli_ASM584v2	106	4864037	50.62	132419	pass	98.9309	pass	0
SAMN30414782	SRR21142213	PRJNA861272	*Escherichia coli*	Jay Graham Laboratory	University of California - Berkeley	2018	Quito, Ecuador	feces	Homo sapiens	Surveillance			100	0.12	pass	E_coli_ASM584v2	154	5030809	50.73	112755	pass	98.4254	pass	0
SAMN30413851	SRR21141996	PRJNA861272	*Escherichia coli*	Jay Graham Laboratory	University of California - Berkeley	2021	Quito, Ecuador	feces	Homo sapiens	Surveillance			100	0.01	pass	E_coli_ASM584v2	90	4506967	50.91	115273	pass	99.0922	pass	0
SAMN30414074	SRR21141408	PRJNA861272	*Escherichia coli*	Jay Graham Laboratory	University of California - Berkeley	2021	Quito, Ecuador	feces	Homo sapiens	Surveillance			100	0.19	pass	E_coli_ASM584v2	282	5044733	50.92	32366	fail	97.2887	pass	1
SAMN30414277	SRR21141567	PRJNA861272	*Escherichia coli*	Jay Graham Laboratory	University of California - Berkeley	2021	Quito, Ecuador	feces	Homo sapiens	Surveillance			99.63	0.16	pass	E_coli_ASM584v2	506	5205431	50.95	22915	fail	98.0446	pass	1
SAMN30414656	SRR21142289	PRJNA861272	*Escherichia coli*	Jay Graham Laboratory	University of California - Berkeley	2018	Quito, Ecuador	feces	Homo sapiens	Surveillance			100	0.12	pass	E_coli_ASM584v2	80	5225657	50.49	260214	pass	97.3235	pass	0
SAMN30414587	SRR21142334	PRJNA861272	*Escherichia coli*	Jay Graham Laboratory	University of California - Berkeley	2018	Quito, Ecuador	feces	Homo sapiens	Surveillance			100	0.06	pass	E_coli_ASM584v2	176	5353654	50.36	130393	pass	97.2224	pass	0
SAMN30414584	SRR21142337	PRJNA861272	*Escherichia coli*	Jay Graham Laboratory	University of California - Berkeley	2018	Quito, Ecuador	feces	Homo sapiens	Surveillance			94.96	1.72	pass	E_coli_ASM584v2	766	5348377	50.23	13835	fail	98.711	pass	1
SAMN30414460	SRR21142346	PRJNA861272	*Escherichia coli*	Jay Graham Laboratory	University of California - Berkeley	2021	Quito, Ecuador	feces	Homo sapiens	Surveillance			100	0.01	pass	E_coli_ASM584v2	125	4563976	50.96	72984	pass	99.6133	pass	0
SAMN30413856	SRR21142348	PRJNA861272	*Escherichia coli*	Jay Graham Laboratory	University of California - Berkeley	2021	Quito, Ecuador	feces	Homo sapiens	Surveillance			100	0.07	pass	E_coli_ASM584v2	104	4795704	50.79	101441	pass	98.3999	pass	0
SAMN30414172	SRR21141587	PRJNA861272	*Escherichia coli*	Jay Graham Laboratory	University of California - Berkeley	2021	Quito, Ecuador	feces	Homo sapiens	Surveillance			64.67	11.14	fail	E_coli_ASM584v2	2064	3951054	49.45	3588	fail	94.8315	pass	2
SAMN30413857	SRR21142074	PRJNA514245	*Escherichia coli*	Jay Graham Laboratory	University of California - Berkeley	2021	Quito, Ecuador	feces	Homo sapiens	Surveillance			100	0.06	pass	E_coli_ASM584v2	219	4394972	50.95	40872	pass	98.4115	pass	0
SAMN30416074	SRR21143929	PRJNA861272	*Escherichia coli*	Jay Graham Laboratory	University of California - Berkeley	2021	Quito, Ecuador	feces	Homo sapiens	Surveillance			100	0.2	pass	E_coli_ASM584v2	213	5182598	50.7	52008	pass	96.0985	pass	0
SAMN30414208	SRR21141771	PRJNA861272	*Escherichia coli*	Jay Graham Laboratory	University of California - Berkeley	2021	Quito, Ecuador	feces	Homo sapiens	Surveillance			100	0.04	pass	E_coli_ASM584v2	253	5106117	50.67	57789	pass	99.4606	pass	0
SAMN30414188	SRR21141378	PRJNA861272	*Escherichia coli*	Jay Graham Laboratory	University of California - Berkeley	2021	Quito, Ecuador	feces	Homo sapiens	Surveillance			100	0.32	pass	E_coli_ASM584v2	173	5105057	50.54	69601	pass	97.2472	pass	0
SAMN30414044	SRR21141633	PRJNA861272	*Escherichia coli*	Jay Graham Laboratory	University of California - Berkeley	2021	Quito, Ecuador	feces	Homo sapiens	Surveillance			100	0.32	pass	E_coli_ASM584v2	255	5632458	50.37	78565	pass	96.0046	pass	0
SAMN30416087	SRR21143915	PRJNA861272	*Escherichia coli*	Jay Graham Laboratory	University of California - Berkeley	2021	Quito, Ecuador	feces	Homo sapiens	Surveillance			99.58	1.08	pass	E_coli_ASM584v2	450	5403969	50.75	37236	fail	98.0276	pass	1
SAMN30414036	SRR21141642	PRJNA861272	*Escherichia coli*	Jay Graham Laboratory	University of California - Berkeley	2021	Quito, Ecuador	feces	Homo sapiens	Surveillance			100	0.05	pass	E_coli_ASM584v2	127	5085579	50.57	138922	pass	98.3574	pass	0
SAMN30416075	SRR21143928	PRJNA861272	*Escherichia coli*	Jay Graham Laboratory	University of California - Berkeley	2021	Quito, Ecuador	feces	Homo sapiens	Surveillance			100	0.2	pass	E_coli_ASM584v2	311	5042408	50.99	37816	fail	97.3292	pass	1
SAMN30415840	SRR21143338	PRJNA861272	*Escherichia coli*	Jay Graham Laboratory	University of California - Berkeley	2018	Quito, Ecuador	feces	Homo sapiens	Surveillance			100	0.09	pass	E_coli_ASM584v2	69	4893912	50.69	187263	pass	98.4163	pass	0
SAMN30414002	SRR21142207	PRJNA861272	*Escherichia coli*	Jay Graham Laboratory	University of California - Berkeley	2021	Quito, Ecuador	feces	Homo sapiens	Surveillance			100	0.3	pass	E_coli_ASM584v2	105	5051501	51.83	148908	pass	82.8144	fail	1
SAMN30414176	SRR21141391	PRJNA861272	*Escherichia coli*	Jay Graham Laboratory	University of California - Berkeley	2021	Quito, Ecuador	feces	Homo sapiens	Surveillance			100	0.15	pass	E_coli_ASM584v2	321	5174961	50.68	42604	pass	98.4449	pass	0
SAMN30414478	SRR21142064	PRJNA514245	*Escherichia coli*	Jay Graham Laboratory	University of California - Berkeley	2021	Quito, Ecuador	feces	Homo sapiens	Surveillance			100	0.21	pass	E_coli_ASM584v2	130	4884189	50.72	80696	pass	98.3922	pass	0

ID	SRA Run	Bioproject	Organism	Recollected	Submitter	Collection date	Geo loc name	Isolation source	Host	Purpose of sampling	Lat ion	Hospital	Completeness_General	Contamination	1st Criterion	FastANI Reference	# contigs	Total length	GC (%)	N50	2nd Criterion	FastANI (%)	3rd Criterion	Failed criteria
SAMN30413923	SRR21141671	PRJNA861272	*Escherichia coli*	Jay Graham Laboratory	University of California - Berkeley	2021	Quito, Ecuador	feces	Homo sapiens	Surveillance			100	0.24	pass	E_coli_ASM584v2	179	4887232	50.58	112837	pass	98.9066	pass	0
SAMN30413903	SRR21141693	PRJNA861272	*Escherichia coli*	Jay Graham Laboratory	University of California - Berkeley	2021	Quito, Ecuador	feces	Homo sapiens	Surveillance			100	0.24	pass	E_coli_ASM584v2	160	4558688	50.85	69099	pass	99.3713	pass	0
SAMN30415834	SRR21143692	PRJNA861272	*Escherichia coli*	Jay Graham Laboratory	University of California - Berkeley	2018	Quito, Ecuador	feces	Homo sapiens	Surveillance			100	0.12	pass	E_coli_ASM584v2	249	5235451	50.57	98122	pass	97.1642	pass	0
SAMN30414033	SRR21141645	PRJNA861272	*Escherichia coli*	Jay Graham Laboratory	University of California - Berkeley	2021	Quito, Ecuador	feces	Homo sapiens	Surveillance			99.96	1.4	pass	E_coli_ASM584v2	86	5141295	50.76	190649	pass	96.7093	pass	0
SAMN30414664	SRR21142280	PRJNA861272	*Escherichia coli*	Jay Graham Laboratory	University of California - Berkeley	2018	Quito, Ecuador	feces	Homo sapiens	Surveillance			99.91	0.09	pass	E_coli_ASM584v2	151	5135227	50.61	101723	pass	97.1469	pass	0
SAMN30414307	SRR21142013	PRJNA861272	*Escherichia coli*	Jay Graham Laboratory	University of California - Berkeley	2021	Quito, Ecuador	feces	Homo sapiens	Surveillance			98.79	0	pass	E_coli_ASM584v2	262	4992497	50.6	40638	pass	98.347	pass	0
SAMN30413932	SRR21141661	PRJNA861272	*Escherichia coli*	Jay Graham Laboratory	University of California - Berkeley	2021	Quito, Ecuador	feces	Homo sapiens	Surveillance			100	0.36	pass	E_coli_ASM584v2	353	5113715	51.02	40316	pass	98.3419	pass	0
SAMN30414289	SRR21141552	PRJNA861272	*Escherichia coli*	Jay Graham Laboratory	University of California - Berkeley	2021	Quito, Ecuador	feces	Homo sapiens	Surveillance			100	0.06	pass	E_coli_ASM584v2	170	4865837	50.71	66219	pass	99.1511	pass	0
SAMN30414254	SRR21142120	PRJNA861272	*Escherichia coli*	Jay Graham Laboratory	University of California - Berkeley	2021	Quito, Ecuador	feces	Homo sapiens	Surveillance			100	0.14	pass	E_coli_ASM584v2	154	4686982	50.96	66259	pass	99.0493	pass	0
SAMN30414566	SRR21141918	PRJNA861272	*Escherichia coli*	Jay Graham Laboratory	University of California - Berkeley	2018	Quito, Ecuador	feces	Homo sapiens	Surveillance			99.15	0.03	pass	E_coli_ASM584v2	139	4628033	50.68	71439	pass	99.1273	pass	0
SAMN30416347	SRR21143487	PRJNA861272	*Escherichia coli*	Jay Graham Laboratory	University of California - Berkeley	2021	Quito, Ecuador	feces	Homo sapiens	Surveillance			100	0.2	pass	E_coli_ASM584v2	203	4994619	50.83	54320	pass	97.2841	pass	0
SAMN30414237	SRR21142139	PRJNA514245	*Escherichia coli*	Jay Graham Laboratory	University of California - Berkeley	2021	Quito, Ecuador	feces	Homo sapiens	Surveillance			100	1.41	pass	E_coli_ASM584v2	224	5279499	50.57	65946	pass	97.8461	pass	0
SAMN30413805	SRR21141451	PRJNA861272	*Escherichia coli*	Jay Graham Laboratory	University of California - Berkeley	2021	Quito, Ecuador	feces	Homo sapiens	Surveillance			92.59	8.46	fail	E_coli_ASM584v2	2072	4777456	50.11	4292	fail	98.6448	pass	2
SAMN30414535	SRR21141952	PRJNA861272	*Escherichia coli*	Jay Graham Laboratory	University of California - Berkeley	2021	Quito, Ecuador	feces	Homo sapiens	Surveillance			100	0.17	pass	E_coli_ASM584v2	215	5084039	50.79	67918	pass	97.1753	pass	0
SAMN30414116	SRR21141777	PRJNA861272	*Escherichia coli*	Jay Graham Laboratory	University of California - Berkeley	2021	Quito, Ecuador	feces	Homo sapiens	Surveillance			100	0.12	pass	E_coli_ASM584v2	123	4988259	50.68	157201	pass	99.4585	pass	0
SAMN30413791	SRR21141823	PRJNA861272	*Escherichia coli*	Jay Graham Laboratory	University of California - Berkeley	2021	Quito, Ecuador	feces	Homo sapiens	Surveillance			85.2	9.15	fail	E_coli_ASM584v2	2587	4790535	49.81	2709	fail	96.7185	pass	2
SAMN30416472	SRR21143709	PRJNA861272	*Escherichia coli*	Jay Graham Laboratory	University of California - Berkeley	2021	Quito, Ecuador	feces	Homo sapiens	Surveillance			100	0	pass	E_coli_ASM584v2	114	4799354	50.71	124571	pass	99.4922	pass	0
SAMN30414721	SRR21141457	PRJNA861272	*Escherichia coli*	Jay Graham Laboratory	University of California - Berkeley	2018	Quito, Ecuador	feces	Homo sapiens	Surveillance			100	0.21	pass	E_coli_ASM584v2	122	4989455	50.62	166818	pass	98.5063	pass	0
SAMN30414238	SRR21142138	PRJNA514245	*Escherichia coli*	Jay Graham Laboratory	University of California - Berkeley	2021	Quito, Ecuador	feces	Homo sapiens	Surveillance			99	0.03	pass	E_coli_ASM584v2	322	4942902	50.93	31516	fail	97.4161	pass	1
SAMN30413866	SRR21141926	PRJNA861272	*Escherichia coli*	Jay Graham Laboratory	University of California - Berkeley	2021	Quito, Ecuador	feces	Homo sapiens	Surveillance			100	0.14	pass	E_coli_ASM584v2	195	5123775	50.83	70921	pass	98.2841	pass	0
SAMN30414306	SRR21142014	PRJNA861272	*Escherichia coli*	Jay Graham Laboratory	University of California - Berkeley	2021	Quito, Ecuador	feces	Homo sapiens	Surveillance			99.84	0.94	pass	E_coli_ASM584v2	214	5307435	50.58	63327	pass	97.1442	pass	0
SAMN30414005	SRR21142204	PRJNA861272	*Escherichia coli*	Jay Graham Laboratory	University of California - Berkeley	2021	Quito, Ecuador	feces	Homo sapiens	Surveillance			100	0.36	pass	E_coli_ASM584v2	864	4844654	50.91	12820	fail	98.9502	pass	1
SAMN30414052	SRR21141624	PRJNA861272	*Escherichia coli*	Jay Graham Laboratory	University of California - Berkeley	2021	Quito, Ecuador	feces	Homo sapiens	Surveillance			100	0.55	pass	E_coli_ASM584v2	104	5206484	50.39	200353	pass	97.0656	pass	0
SAMN30414154	SRR21141607	PRJNA861272	*Escherichia coli*	Jay Graham Laboratory	University of California - Berkeley	2021	Quito, Ecuador	feces	Homo sapiens	Surveillance			100	0.16	pass	E_coli_ASM584v2	86	5103450	50.72	226057	pass	97.3077	pass	0

ID	SRA Run	Bioproject	Organism	Recollected	Submitter	Collection date	Geo loc name	Isolation source	Host	Purpose of sampling	Lat ion	Hospital	Completeness_General	Contamination	1st Criterion	FastANI Reference	# contigs	Total length	GC (%)	N50	2nd Criterion	FastANI (%)	3rd Criterion	Failed criteria
SAMN30414568	SRR21141916	PRJNA861272	*Escherichia coli*	Jay Graham Laboratory	University of California - Berkeley	2018	Quito, Ecuador	feces	Homo sapiens	Surveillance			100	0.16	pass	E_coli_ASM584v2	110	4856817	50.68	119331	pass	99.314	pass	0
SAMN30414663	SRR21142281	PRJNA861272	*Escherichia coli*	Jay Graham Laboratory	University of California - Berkeley	2018	Quito, Ecuador	feces	Homo sapiens	Surveillance			100	0.09	pass	E_coli_ASM584v2	139	5180122	50.61	144913	pass	97.164	pass	0
SAMN30414428	SRR21141975	PRJNA861272	*Escherichia coli*	Jay Graham Laboratory	University of California - Berkeley	2021	Quito, Ecuador	feces	Homo sapiens	Surveillance			100	1.01	pass	E_coli_ASM584v2	240	4949874	50.99	39235	fail	96.7606	pass	1
SAMN30414284	SRR21141559	PRJNA861272	*Escherichia coli*	Jay Graham Laboratory	University of California - Berkeley	2021	Quito, Ecuador	feces	Homo sapiens	Surveillance			100	1.03	pass	E_coli_ASM584v2	203	5217778	50.55	54539	pass	98.3112	pass	0
SAMN30416449	SRR21143734	PRJNA861272	*Escherichia coli*	Jay Graham Laboratory	University of California - Berkeley	2021	Quito, Ecuador	feces	Homo sapiens	Surveillance			100	1.09	pass	E_coli_ASM584v2	207	5466750	50.49	80670	pass	97.1507	pass	0
SAMN30414228	SRR21141749	PRJNA861272	*Escherichia coli*	Jay Graham Laboratory	University of California - Berkeley	2021	Quito, Ecuador	feces	Homo sapiens	Surveillance			98.4	0.57	pass	E_coli_ASM584v2	403	5172398	50.89	24243	fail	97.1396	pass	1
SAMN30416089	SRR21143912	PRJNA861272	*Escherichia coli*	Jay Graham Laboratory	University of California - Berkeley	2021	Quito, Ecuador	feces	Homo sapiens	Surveillance			100	0.07	pass	E_coli_ASM584v2	196	4727066	50.78	58773	pass	99.5092	pass	0
SAMN30414180	SRR21141386	PRJNA861272	*Escherichia coli*	Jay Graham Laboratory	University of California - Berkeley	2021	Quito, Ecuador	feces	Homo sapiens	Surveillance			99.58	0.63	pass	E_coli_ASM584v2	227	5604125	51.42	77568	pass	82.3799	fail	1
SAMN30413836	SRR21142115	PRJNA861272	*Escherichia coli*	Jay Graham Laboratory	University of California - Berkeley	2021	Quito, Ecuador	feces	Homo sapiens	Surveillance			100	1.17	pass	E_coli_ASM584v2	127	5029518	50.82	123597	pass	96.7799	pass	0
SAMN30414718	SRR21141461	PRJNA861272	*Escherichia coli*	Jay Graham Laboratory	University of California - Berkeley	2018	Quito, Ecuador	feces	Homo sapiens	Surveillance			98.56	0.86	pass	E_coli_ASM584v2	252	4854747	50.83	37906	fail	98.4154	pass	1
SAMN30414714	SRR21141465	PRJNA861272	*Escherichia coli*	Jay Graham Laboratory	University of California - Berkeley	2018	Quito, Ecuador	feces	Homo sapiens	Surveillance			100	0.3	pass	E_coli_ASM584v2	153	5020085	50.51	97860	pass	99.137	pass	0
SAMN30413943	SRR21141649	PRJNA861272	*Escherichia coli*	Jay Graham Laboratory	University of California - Berkeley	2021	Quito, Ecuador	feces	Homo sapiens	Surveillance			100	0.02	pass	E_coli_ASM584v2	222	4692968	50.78	44304	pass	99.6462	pass	0
SAMN30414072	SRR21141410	PRJNA861272	*Escherichia coli*	Jay Graham Laboratory	University of California - Berkeley	2021	Quito, Ecuador	feces	Homo sapiens	Surveillance			100	0.33	pass	E_coli_ASM584v2	259	5394902	50.21	77130	pass	99.4553	pass	0
SAMN30414710	SRR21141469	PRJNA861272	*Escherichia coli*	Jay Graham Laboratory	University of California - Berkeley	2018	Quito, Ecuador	feces	Homo sapiens	Surveillance			99.82	0.31	pass	E_coli_ASM584v2	256	4956086	50.52	37627	fail	99.1634	pass	1
SAMN30413792	SRR21141392	PRJNA861272	*Escherichia coli*	Jay Graham Laboratory	University of California - Berkeley	2021	Quito, Ecuador	feces	Homo sapiens	Surveillance			93.3	14.92	fail	E_coli_ASM584v2	2598	5820264	50.46	4430	fail	97.4997	pass	2
SAMN30414665	SRR21142279	PRJNA861272	*Escherichia coli*	Jay Graham Laboratory	University of California - Berkeley	2018	Quito, Ecuador	feces	Homo sapiens	Surveillance			100	0.16	pass	E_coli_ASM584v2	141	5139888	50.58	123087	pass	97.1561	pass	0
SAMN30414010	SRR21142198	PRJNA861272	*Escherichia coli*	Jay Graham Laboratory	University of California - Berkeley	2021	Quito, Ecuador	feces	Homo sapiens	Surveillance			100	0.24	pass	E_coli_ASM584v2	110	5066335	50.38	152607	pass	97.1322	pass	0
SAMN30414240	SRR21142135	PRJNA514245	*Escherichia coli*	Jay Graham Laboratory	University of California - Berkeley	2021	Quito, Ecuador	feces	Homo sapiens	Surveillance			100	0.04	pass	E_coli_ASM584v2	295	4815723	50.65	34775	fail	99.6037	pass	1
SAMN30413896	SRR21141701	PRJNA861272	*Escherichia coli*	Jay Graham Laboratory	University of California - Berkeley	2021	Quito, Ecuador	feces	Homo sapiens	Surveillance			100	0.04	pass	E_coli_ASM584v2	196	5466295	50.37	156999	pass	97.2189	pass	0
SAMN30413956	SRR21141443	PRJNA861272	*Escherichia coli*	Jay Graham Laboratory	University of California - Berkeley	2021	Quito, Ecuador	feces	Homo sapiens	Surveillance			100	0.37	pass	E_coli_ASM584v2	255	5546872	50.54	100249	pass	97.3514	pass	0
SAMN30416389	SRR21143801	PRJNA861272	*Escherichia coli*	Jay Graham Laboratory	University of California - Berkeley	2021	Quito, Ecuador	feces	Homo sapiens	Surveillance			100	0.05	pass	E_coli_ASM584v2	160	5199866	50.68	100991	pass	97.2242	pass	0
SAMN30413860	SRR21141512	PRJNA861272	*Escherichia coli*	Jay Graham Laboratory	University of California - Berkeley	2021	Quito, Ecuador	feces	Homo sapiens	Surveillance			100	0.1	pass	E_coli_ASM584v2	201	4903835	50.89	53603	pass	99.1091	pass	0
SAMN30416351	SRR21143482	PRJNA861272	*Escherichia coli*	Jay Graham Laboratory	University of California - Berkeley	2021	Quito, Ecuador	feces	Homo sapiens	Surveillance			100	0.27	pass	E_coli_ASM584v2	199	5253932	50.78	65450	pass	97.8839	pass	0
SAMN30414279	SRR21141564	PRJNA861272	*Escherichia coli*	Jay Graham Laboratory	University of California - Berkeley	2021	Quito, Ecuador	feces	Homo sapiens	Surveillance			98.5	0.08	pass	E_coli_ASM584v2	574	5030189	50.93	18342	fail	97.2158	pass	1

ID	SRA Run	Bioproject	Organism	Recollected	Submitter	Collection date	Geo loc name	Isolation source	Host	Purpose of sampling	Lat ion	Hospital	Completeness_General	Contamination	1st Criterion	FastANI Reference	# contigs	Total length	GC (%)	N50	2nd Criterion	FastANI (%)	3rd Criterion	Failed criteria
SAMN30414147	SRR21141615	PRJNA861272	*Escherichia coli*	Jay Graham Laboratory	University of California - Berkeley	2021	Quito, Ecuador	feces	Homo sapiens	Surveillance			100	0.11	pass	E_coli_ASM584v2	226	4750990	50.88	42410	pass	99.5513	pass	0
SAMN30414628	SRR21141881	PRJNA861272	*Escherichia coli*	Jay Graham Laboratory	University of California - Berkeley	2018	Quito, Ecuador	feces	Homo sapiens	Surveillance			100	0.03	pass	E_coli_ASM584v2	104	4703468	50.75	147296	pass	99.5627	pass	0
SAMN30416399	SRR21143790	PRJNA861272	*Escherichia coli*	Jay Graham Laboratory	University of California - Berkeley	2021	Quito, Ecuador	feces	Homo sapiens	Surveillance			100	0.12	pass	E_coli_ASM584v2	199	5015982	50.66	74976	pass	99.3232	pass	0
SAMN30414138	SRR21142153	PRJNA514245	*Escherichia coli*	Jay Graham Laboratory	University of California - Berkeley	2021	Quito, Ecuador	feces	Homo sapiens	Surveillance			100	0.06	pass	E_coli_ASM584v2	303	5429141	50.47	59682	pass	97.2419	pass	0
SAMN30414326	SRR21141736	PRJNA861272	*Escherichia coli*	Jay Graham Laboratory	University of California - Berkeley	2021	Quito, Ecuador	feces	Homo sapiens	Surveillance			100	0.09	pass	E_coli_ASM584v2	296	4694792	51.01	30060	fail	99.3625	pass	1
SAMN30414371	SRR21142086	PRJNA861272	*Escherichia coli*	Jay Graham Laboratory	University of California - Berkeley	2021	Quito, Ecuador	feces	Homo sapiens	Surveillance			100	0.18	pass	E_coli_ASM584v2	355	5015287	50.81	37433	fail	98.8489	pass	1
SAMN30413807	SRR21141429	PRJNA861272	*Escherichia coli*	Jay Graham Laboratory	University of California - Berkeley	2021	Quito, Ecuador	feces	Homo sapiens	Surveillance			100	0.11	pass	E_coli_ASM584v2	211	5210578	51	64626	pass	98.3587	pass	0
SAMN30413967	SRR21141431	PRJNA861272	*Escherichia coli*	Jay Graham Laboratory	University of California - Berkeley	2021	Quito, Ecuador	feces	Homo sapiens	Surveillance			100	0.14	pass	E_coli_ASM584v2	182	5530055	50.54	105642	pass	97.1876	pass	0
SAMN30414077	SRR21141405	PRJNA861272	*Escherichia coli*	Jay Graham Laboratory	University of California - Berkeley	2021	Quito, Ecuador	feces	Homo sapiens	Surveillance			100	0.38	pass	E_coli_ASM584v2	308	5235992	50.68	49683	pass	98.4015	pass	0
SAMN30416470	SRR21143711	PRJNA861272	*Escherichia coli*	Jay Graham Laboratory	University of California - Berkeley	2021	Quito, Ecuador	feces	Homo sapiens	Surveillance			100	0.08	pass	E_coli_ASM584v2	78	5041331	53.59	198375	pass	81.1351	fail	1
SAMN30414565	SRR21141919	PRJNA861272	*Escherichia coli*	Jay Graham Laboratory	University of California - Berkeley	2018	Quito, Ecuador	feces	Homo sapiens	Surveillance			100	0.1	pass	E_coli_ASM584v2	69	4693053	50.66	160757	pass	99.1235	pass	0
SAMN30413954	SRR21141445	PRJNA861272	*Escherichia coli*	Jay Graham Laboratory	University of California - Berkeley	2021	Quito, Ecuador	feces	Homo sapiens	Surveillance			95.88	0.8	pass	E_coli_ASM584v2	754	5258147	50.12	17268	fail	98.1881	pass	1
SAMN30414265	SRR21141580	PRJNA861272	*Escherichia coli*	Jay Graham Laboratory	University of California - Berkeley	2021	Quito, Ecuador	feces	Homo sapiens	Surveillance			100	1.07	pass	E_coli_ASM584v2	265	5316366	50.62	58693	pass	97.9806	pass	0
SAMN30414645	SRR21142301	PRJNA861272	*Escherichia coli*	Jay Graham Laboratory	University of California - Berkeley	2018	Quito, Ecuador	feces	Homo sapiens	Surveillance			100	0.08	pass	E_coli_ASM584v2	92	5142171	50.6	165883	pass	97.1879	pass	0
SAMN30414161	SRR21141599	PRJNA861272	*Escherichia coli*	Jay Graham Laboratory	University of California - Berkeley	2021	Quito, Ecuador	feces	Homo sapiens	Surveillance			100	0.12	pass	E_coli_ASM584v2	186	4692681	50.91	54338	pass	98.401	pass	0
SAMN30413824	SRR21142152	PRJNA514245	*Escherichia coli*	Jay Graham Laboratory	University of California - Berkeley	2021	Quito, Ecuador	feces	Homo sapiens	Surveillance			100	0.26	pass	E_coli_ASM584v2	225	5116139	50.7	47625	pass	97.2283	pass	0
SAMN30414465	SRR21142078	PRJNA514245	*Escherichia coli*	Jay Graham Laboratory	University of California - Berkeley	2021	Quito, Ecuador	feces	Homo sapiens	Surveillance			98.33	0	pass	E_coli_ASM584v2	291	4681810	50.75	34626	fail	98.8875	pass	1
SAMN30414153	SRR21141608	PRJNA861272	*Escherichia coli*	Jay Graham Laboratory	University of California - Berkeley	2021	Quito, Ecuador	feces	Homo sapiens	Surveillance			100	0.43	pass	E_coli_ASM584v2	165	4988835	50.63	79332	pass	99.0082	pass	0
SAMN30414029	SRR21142177	PRJNA861272	*Escherichia coli*	Jay Graham Laboratory	University of California - Berkeley	2021	Quito, Ecuador	feces	Homo sapiens	Surveillance			100	0.2	pass	E_coli_ASM584v2	181	4758543	50.5	77151	pass	98.929	pass	0
SAMN30416407	SRR21143782	PRJNA861272	*Escherichia coli*	Jay Graham Laboratory	University of California - Berkeley	2021	Quito, Ecuador	feces	Homo sapiens	Surveillance			100	0.32	pass	E_coli_ASM584v2	164	5303428	50.72	154568	pass	97.2647	pass	0
SAMN30416376	SRR21143393	PRJNA861272	*Escherichia coli*	Jay Graham Laboratory	University of California - Berkeley	2021	Quito, Ecuador	feces	Homo sapiens	Surveillance			100	0.41	pass	E_coli_ASM584v2	326	5445779	50.62	52694	pass	97.2355	pass	0
SAMN30414085	SRR21141396	PRJNA861272	*Escherichia coli*	Jay Graham Laboratory	University of California - Berkeley	2021	Quito, Ecuador	feces	Homo sapiens	Surveillance			98.86	0.5	pass	E_coli_ASM584v2	231	5204871	50.59	47458	pass	97.8253	pass	0
SAMN30414342	SRR21141718	PRJNA861272	*Escherichia coli*	Jay Graham Laboratory	University of California - Berkeley	2021	Quito, Ecuador	feces	Homo sapiens	Surveillance			100	0.06	pass	E_coli_ASM584v2	239	4816989	50.85	45208	pass	99.1186	pass	0
SAMN30414148	SRR21141614	PRJNA861272	*Escherichia coli*	Jay Graham Laboratory	University of California - Berkeley	2021	Quito, Ecuador	feces	Homo sapiens	Surveillance			100	0.11	pass	E_coli_ASM584v2	309	5025736	50.95	38593	fail	97.1707	pass	1

ID	SRA Run	Bioproject	Organism	Recollected	Submmiter	Collection date	Geo loc name	Isolation source	Host	Purpose of sampling	Lat ion	Hospital	Completeness_General	Contamination	1st Criterion	FastANI Reference	# contigs	Total length	GC (%)	N50	2nd Criterion	FastANI (%)	3rd Criterion	Failed criteria
SAMN30414059	SRR21141616	PRJNA861272	*Escherichia coli*	Jay Graham Laboratory	University of California - Berkeley	2021	Quito, Ecuador	feces	Homo sapiens	Surveillance			100	0.19	pass	E_coli_ASM584v2	188	4916606	50.61	77846	pass	99.438	pass	0
SAMN30414206	SRR21141773	PRJNA861272	*Escherichia coli*	Jay Graham Laboratory	University of California - Berkeley	2021	Quito, Ecuador	feces	Homo sapiens	Surveillance			100	0.12	pass	E_coli_ASM584v2	186	5031004	50.77	67757	pass	98.3331	pass	0
SAMN30414658	SRR21142287	PRJNA861272	*Escherichia coli*	Jay Graham Laboratory	University of California - Berkeley	2018	Quito, Ecuador	feces	Homo sapiens	Surveillance			100	0.13	pass	E_coli_ASM584v2	77	5220963	50.49	224532	pass	97.2873	pass	0
SAMN30414083	SRR21141398	PRJNA861272	*Escherichia coli*	Jay Graham Laboratory	University of California - Berkeley	2021	Quito, Ecuador	feces	Homo sapiens	Surveillance			100	1.07	pass	E_coli_ASM584v2	194	5148652	50.73	55589	pass	97.8669	pass	0
SAMN30414449	SRR21142358	PRJNA861272	*Escherichia coli*	Jay Graham Laboratory	University of California - Berkeley	2021	Quito, Ecuador	feces	Homo sapiens	Surveillance			100	1	pass	E_coli_ASM584v2	264	5469011	50.86	52853	pass	98.0582	pass	0
SAMN30414541	SRR21141945	PRJNA861272	*Escherichia coli*	Jay Graham Laboratory	University of California - Berkeley	2021	Quito, Ecuador	feces	Homo sapiens	Surveillance			100	0.17	pass	E_coli_ASM584v2	218	5098175	50.7	76802	pass	97.2093	pass	0
SAMN30416425	SRR21143371	PRJNA861272	*Escherichia coli*	Jay Graham Laboratory	University of California - Berkeley	2021	Quito, Ecuador	feces	Homo sapiens	Surveillance			100	0.02	pass	E_coli_ASM584v2	179	5364812	50.53	93861	pass	97.2245	pass	0
SAMN30414013	SRR21142195	PRJNA861272	*Escherichia coli*	Jay Graham Laboratory	University of California - Berkeley	2021	Quito, Ecuador	feces	Homo sapiens	Surveillance			100	0	pass	E_coli_ASM584v2	188	5045187	50.66	81973	pass	98.3042	pass	0
SAMN30414321	SRR21141741	PRJNA861272	*Escherichia coli*	Jay Graham Laboratory	University of California - Berkeley	2021	Quito, Ecuador	feces	Homo sapiens	Surveillance			100	0.02	pass	E_coli_ASM584v2	132	5104328	50.83	90905	pass	97.2826	pass	0
SAMN30413998	SRR21141812	PRJNA861272	*Escherichia coli*	Jay Graham Laboratory	University of California - Berkeley	2021	Quito, Ecuador	feces	Homo sapiens	Surveillance			100	0.57	pass	E_coli_ASM584v2	148	5020634	50.64	106364	pass	97.8829	pass	0
SAMN30414027	SRR21142180	PRJNA861272	*Escherichia coli*	Jay Graham Laboratory	University of California - Berkeley	2021	Quito, Ecuador	feces	Homo sapiens	Surveillance			100	0.16	pass	E_coli_ASM584v2	227	5062231	50.75	59543	pass	97.1292	pass	0
SAMN30414181	SRR21141385	PRJNA861272	*Escherichia coli*	Jay Graham Laboratory	University of California - Berkeley	2021	Quito, Ecuador	feces	Homo sapiens	Surveillance			100	0.17	pass	E_coli_ASM584v2	162	4854381	50.52	76707	pass	98.8399	pass	0
SAMN30413890	SRR21141707	PRJNA861272	*Escherichia coli*	Jay Graham Laboratory	University of California - Berkeley	2021	Quito, Ecuador	feces	Homo sapiens	Surveillance			100	0.59	pass	E_coli_ASM584v2	159	5232349	50.6	103040	pass	98.3664	pass	0
SAMN30414308	SRR21142012	PRJNA861272	*Escherichia coli*	Jay Graham Laboratory	University of California - Berkeley	2021	Quito, Ecuador	feces	Homo sapiens	Surveillance			100	1.11	pass	E_coli_ASM584v2	225	5292999	50.64	53563	pass	97.1947	pass	0
SAMN30416071	SRR21143932	PRJNA861272	*Escherichia coli*	Jay Graham Laboratory	University of California - Berkeley	2021	Quito, Ecuador	feces	Homo sapiens	Surveillance			100	0.08	pass	E_coli_ASM584v2	154	4849347	50.81	68457	pass	98.3296	pass	0
SAMN30414716	SRR21141463	PRJNA861272	*Escherichia coli*	Jay Graham Laboratory	University of California - Berkeley	2018	Quito, Ecuador	feces	Homo sapiens	Surveillance			100	0.06	pass	E_coli_ASM584v2	161	4919316	50.83	111786	pass	98.47	pass	0
SAMN30414288	SRR21141555	PRJNA861272	*Escherichia coli*	Jay Graham Laboratory	University of California - Berkeley	2021	Quito, Ecuador	feces	Homo sapiens	Surveillance			99.6	0.06	pass	E_coli_ASM584v2	241	5152755	50.63	64393	pass	97.2666	pass	0
SAMN30414128	SRR21142164	PRJNA861272	*Escherichia coli*	Jay Graham Laboratory	University of California - Berkeley	2021	Quito, Ecuador	feces	Homo sapiens	Surveillance			100	0.25	pass	E_coli_ASM584v2	214	5210150	50.83	59721	pass	97.311	pass	0
SAMN30414588	SRR21142333	PRJNA861272	*Escherichia coli*	Jay Graham Laboratory	University of California - Berkeley	2018	Quito, Ecuador	feces	Homo sapiens	Surveillance			100	0.08	pass	E_coli_ASM584v2	217	5283028	50.42	90394	pass	97.2271	pass	0
SAMN30413794	SRR21141554	PRJNA861272	*Escherichia coli*	Jay Graham Laboratory	University of California - Berkeley	2021	Quito, Ecuador	feces	Homo sapiens	Surveillance			100	0.1	pass	E_coli_ASM584v2	149	4761384	50.71	76366	pass	98.3833	pass	0
SAMN30414239	SRR21142136	PRJNA514245	*Escherichia coli*	Jay Graham Laboratory	University of California - Berkeley	2021	Quito, Ecuador	feces	Homo sapiens	Surveillance			99.93	0.45	pass	E_coli_ASM584v2	319	5194002	50.75	39901	fail	99.4734	pass	1
SAMN30414070	SRR21141412	PRJNA861272	*Escherichia coli*	Jay Graham Laboratory	University of California - Berkeley	2021	Quito, Ecuador	feces	Homo sapiens	Surveillance			97.94	0.97	pass	E_coli_ASM584v2	1022	4973615	50.69	9468	fail	97.1738	pass	1
SAMN30414763	SRR21142266	PRJNA861272	*Escherichia coli*	Jay Graham Laboratory	University of California - Berkeley	2018	Quito, Ecuador	feces	Homo sapiens	Surveillance			100	1.72	pass	E_coli_ASM584v2	208	5330357	50.6	90118	pass	97.2432	pass	0
SAMN30414274	SRR21141570	PRJNA861272	*Escherichia coli*	Jay Graham Laboratory	University of California - Berkeley	2021	Quito, Ecuador	feces	Homo sapiens	Surveillance			99.05	0.32	pass	E_coli_ASM584v2	350	4922818	50.69	34846	fail	99.069	pass	1

ID	SRA Run	Bioproject	Organism	Recollected	Submmiter	Collection date	Geo loc name	Isolation source	Host	Purpose of sampling	Lat ion	Hospital	Completeness_General	Contamination	1st Criterion	FastANI Reference	# contigs	Total length	GC (%)	N50	2nd Criterion	FastANI (%)	3rd Criterion	Failed criteria
SAMN30414434	SRR21141968	PRJNA861272	*Escherichia coli*	Jay Graham Laboratory	University of California - Berkeley	2021	Quito, Ecuador	feces	Homo sapiens	Surveillance			100	0.06	pass	E_coli_ASM584v2	155	5015925	50.45	93766	pass	99.6192	pass	0
SAMN30413964	SRR21141434	PRJNA861272	*Escherichia coli*	Jay Graham Laboratory	University of California - Berkeley	2021	Quito, Ecuador	feces	Homo sapiens	Surveillance			100	0.01	pass	E_coli_ASM584v2	232	4515419	51.01	41598	pass	99.3095	pass	0
SAMN30414312	SRR21142007	PRJNA861272	*Escherichia coli*	Jay Graham Laboratory	University of California - Berkeley	2021	Quito, Ecuador	feces	Homo sapiens	Surveillance			99.37	0.24	pass	E_coli_ASM584v2	581	5006496	51.08	13524	fail	99.4729	pass	1
SAMN30414054	SRR21141622	PRJNA861272	*Escherichia coli*	Jay Graham Laboratory	University of California - Berkeley	2021	Quito, Ecuador	feces	Homo sapiens	Surveillance			100	0.17	pass	E_coli_ASM584v2	204	4891234	50.61	57328	pass	98.3165	pass	0
SAMN30414056	SRR21141620	PRJNA861272	*Escherichia coli*	Jay Graham Laboratory	University of California - Berkeley	2021	Quito, Ecuador	feces	Homo sapiens	Surveillance			99.11	0.12	pass	E_coli_ASM584v2	271	5130854	50.82	54715	pass	97.2282	pass	0
SAMN30413861	SRR21141501	PRJNA861272	*Escherichia coli*	Jay Graham Laboratory	University of California - Berkeley	2021	Quito, Ecuador	feces	Homo sapiens	Surveillance			100	0.07	pass	E_coli_ASM584v2	139	4758492	50.91	74816	pass	99.018	pass	0
SAMN30414249	SRR21142125	PRJNA861272	*Escherichia coli*	Jay Graham Laboratory	University of California - Berkeley	2021	Quito, Ecuador	feces	Homo sapiens	Surveillance			100	0.14	pass	E_coli_ASM584v2	270	5108546	50.91	40241	pass	97.1654	pass	0
SAMN30414278	SRR21141566	PRJNA861272	*Escherichia coli*	Jay Graham Laboratory	University of California - Berkeley	2021	Quito, Ecuador	feces	Homo sapiens	Surveillance			97.49	1.17	pass	E_coli_ASM584v2	1363	4608034	51.13	6767	fail	99.5845	pass	1
SAMN30414667	SRR21142245	PRJNA861272	*Escherichia coli*	Jay Graham Laboratory	University of California - Berkeley	2018	Quito, Ecuador	feces	Homo sapiens	Surveillance			100	0.25	pass	E_coli_ASM584v2	206	5213976	50.76	115434	pass	97.0336	pass	0
SAMN30414482	SRR21142059	PRJNA514245	*Escherichia coli*	Jay Graham Laboratory	University of California - Berkeley	2021	Quito, Ecuador	feces	Homo sapiens	Surveillance			100	0.17	pass	E_coli_ASM584v2	199	5271199	50.76	98154	pass	97.1401	pass	0
SAMN30413951	SRR21141448	PRJNA861272	*Escherichia coli*	Jay Graham Laboratory	University of California - Berkeley	2021	Quito, Ecuador	feces	Homo sapiens	Surveillance			98.8	0.05	pass	E_coli_ASM584v2	119	4824221	53.88	85609	pass	81.0929	fail	1
SAMN30414514	SRR21141495	PRJNA861272	*Escherichia coli*	Jay Graham Laboratory	University of California - Berkeley	2021	Quito, Ecuador	feces	Homo sapiens	Surveillance			96.52	0.89	pass	E_coli_ASM584v2	346	4850582	50.7	46268	pass	99.3599	pass	0
SAMN30413955	SRR21141444	PRJNA861272	*Escherichia coli*	Jay Graham Laboratory	University of California - Berkeley	2021	Quito, Ecuador	feces	Homo sapiens	Surveillance			100	0.35	pass	E_coli_ASM584v2	233	5186826	50.75	64143	pass	98.8099	pass	0
SAMN30414540	SRR21141946	PRJNA861272	*Escherichia coli*	Jay Graham Laboratory	University of California - Berkeley	2021	Quito, Ecuador	feces	Homo sapiens	Surveillance			100	0.71	pass	E_coli_ASM584v2	316	5352263	50.62	39195	fail	97.1806	pass	1
SAMN30415828	SRR21143949	PRJNA861272	*Escherichia coli*	Jay Graham Laboratory	University of California - Berkeley	2018	Quito, Ecuador	feces	Homo sapiens	Surveillance			100	0.29	pass	E_coli_ASM584v2	118	5279863	50.53	191127	pass	97.2954	pass	0
SAMN30416440	SRR21143353	PRJNA861272	*Escherichia coli*	Jay Graham Laboratory	University of California - Berkeley	2021	Quito, Ecuador	feces	Homo sapiens	Surveillance			98.5	0	pass	E_coli_ASM584v2	391	5023901	50.55	25992	fail	99.4639	pass	1
SAMN30414247	SRR21142128	PRJNA514245	*Escherichia coli*	Jay Graham Laboratory	University of California - Berkeley	2021	Quito, Ecuador	feces	Homo sapiens	Surveillance			100	0.21	pass	E_coli_ASM584v2	189	5034265	50.79	61621	pass	97.3517	pass	0
SAMN30416117	SRR21143651	PRJNA861272	*Escherichia coli*	Jay Graham Laboratory	University of California - Berkeley	2021	Quito, Ecuador	feces	Homo sapiens	Surveillance			100	0.07	pass	E_coli_ASM584v2	79	4927754	50.58	189975	pass	98.4162	pass	0
SAMN30413950	SRR21141449	PRJNA861272	*Escherichia coli*	Jay Graham Laboratory	University of California - Berkeley	2021	Quito, Ecuador	feces	Homo sapiens	Surveillance			99.99	0.81	pass	E_coli_ASM584v2	153	5066195	54.97	86058	pass	81.9126	fail	1
SAMN30414123	SRR21142169	PRJNA861272	*Escherichia coli*	Jay Graham Laboratory	University of California - Berkeley	2021	Quito, Ecuador	feces	Homo sapiens	Surveillance			100	0.19	pass	E_coli_ASM584v2	254	5180766	50.96	49179	pass	97.3331	pass	0
SAMN30414230	SRR21141746	PRJNA861272	*Escherichia coli*	Jay Graham Laboratory	University of California - Berkeley	2021	Quito, Ecuador	feces	Homo sapiens	Surveillance			100	0.23	pass	E_coli_ASM584v2	237	4532358	51.01	35659	fail	99.1243	pass	1
SAMN30413946	SRR21141454	PRJNA861272	*Escherichia coli*	Jay Graham Laboratory	University of California - Berkeley	2021	Quito, Ecuador	feces	Homo sapiens	Surveillance			100	0.05	pass	E_coli_ASM584v2	194	4876865	50.86	55689	pass	98.3519	pass	0
SAMN30414448	SRR21142360	PRJNA861272	*Escherichia coli*	Jay Graham Laboratory	University of California - Berkeley	2021	Quito, Ecuador	feces	Homo sapiens	Surveillance			100	0.29	pass	E_coli_ASM584v2	345	5496816	50.76	47257	pass	97.3499	pass	0
SAMN30414139	SRR21142151	PRJNA514245	*Escherichia coli*	Jay Graham Laboratory	University of California - Berkeley	2021	Quito, Ecuador	feces	Homo sapiens	Surveillance			100	0.43	pass	E_coli_ASM584v2	319	4819554	50.84	37367	fail	99.0709	pass	1

ID	SRA Run	Bioproject	Organism	Recollected	Submmiter	Collection date	Geo loc name	Isolation source	Host	Purpose of sampling	Lat ion	Hospital	Completeness_General	Contamination	1st Criterion	FastANI Reference	# contigs	Total length	GC (%)	N50	2nd Criterion	FastANI (%)	3rd Criterion	Failed criteria
SAMN30414657	SRR21142288	PRJNA861272	*Escherichia coli*	Jay Graham Laboratory	University of California - Berkeley	2018	Quito, Ecuador	feces	Homo sapiens	Surveillance			100	0.13	pass	E_coli_ASM584v2	82	5226540	50.49	242104	pass	97.2874	pass	0
SAMN30413931	SRR21141662	PRJNA861272	*Escherichia coli*	Jay Graham Laboratory	University of California - Berkeley	2021	Quito, Ecuador	feces	Homo sapiens	Surveillance			99.99	0.91	pass	E_coli_ASM584v2	101	5364794	50.44	158481	pass	96.7145	pass	0
SAMN30416413	SRR21143384	PRJNA861272	*Escherichia coli*	Jay Graham Laboratory	University of California - Berkeley	2021	Quito, Ecuador	feces	Homo sapiens	Surveillance			75.4	8.35	fail	E_coli_ASM584v2	1915	3964333	50.2	3381	fail	96.1272	pass	2
SAMN30414303	SRR21142017	PRJNA861272	*Escherichia coli*	Jay Graham Laboratory	University of California - Berkeley	2021	Quito, Ecuador	feces	Homo sapiens	Surveillance			100	0.31	pass	E_coli_ASM584v2	219	4988395	50.68	53127	pass	99.6903	pass	0
SAMN30414345	SRR21141715	PRJNA861272	*Escherichia coli*	Jay Graham Laboratory	University of California - Berkeley	2021	Quito, Ecuador	feces	Homo sapiens	Surveillance			99.69	0.34	pass	E_coli_ASM584v2	324	4866427	51.05	31733	fail	96.8104	pass	1
SAMN30414351	SRR21142108	PRJNA861272	*Escherichia coli*	Jay Graham Laboratory	University of California - Berkeley	2021	Quito, Ecuador	feces	Homo sapiens	Surveillance			99.99	0.09	pass	E_coli_ASM584v2	546	5115090	51.05	19186	fail	95.9899	pass	1
SAMN30414167	SRR21141593	PRJNA861272	*Escherichia coli*	Jay Graham Laboratory	University of California - Berkeley	2021	Quito, Ecuador	feces	Homo sapiens	Surveillance			100	0.06	pass	E_coli_ASM584v2	242	5101849	50.59	92804	pass	99.4303	pass	0
SAMN30414270	SRR21141574	PRJNA861272	*Escherichia coli*	Jay Graham Laboratory	University of California - Berkeley	2021	Quito, Ecuador	feces	Homo sapiens	Surveillance			100	0.05	pass	E_coli_ASM584v2	183	5086753	50.87	99388	pass	98.3715	pass	0
SAMN30414220	SRR21141757	PRJNA861272	*Escherichia coli*	Jay Graham Laboratory	University of California - Berkeley	2021	Quito, Ecuador	feces	Homo sapiens	Surveillance			100	0.16	pass	E_coli_ASM584v2	200	4826408	50.81	42759	pass	98.3924	pass	0
SAMN30414408	SRR21141997	PRJNA861272	*Escherichia coli*	Jay Graham Laboratory	University of California - Berkeley	2021	Quito, Ecuador	feces	Homo sapiens	Surveillance			100	0.18	pass	E_coli_ASM584v2	131	4806364	50.55	93978	pass	99.5958	pass	0
SAMN30416073	SRR21143930	PRJNA861272	*Escherichia coli*	Jay Graham Laboratory	University of California - Berkeley	2021	Quito, Ecuador	feces	Homo sapiens	Surveillance			100	0.27	pass	E_coli_ASM584v2	268	5484136	50.55	48774	pass	96.027	pass	0
SAMN30414092	SRR21141803	PRJNA861272	*Escherichia coli*	Jay Graham Laboratory	University of California - Berkeley	2021	Quito, Ecuador	feces	Homo sapiens	Surveillance			100	0.22	pass	E_coli_ASM584v2	118	5136946	50.63	185531	pass	97.1328	pass	0
SAMN30414424	SRR21141979	PRJNA861272	*Escherichia coli*	Jay Graham Laboratory	University of California - Berkeley	2021	Quito, Ecuador	feces	Homo sapiens	Surveillance			96.76	7.84	fail	E_coli_ASM584v2	1159	5625663	50.87	10649	fail	96.9194	pass	2
SAMN30414198	SRR21142038	PRJNA861272	*Escherichia coli*	Jay Graham Laboratory	University of California - Berkeley	2021	Quito, Ecuador	feces	Homo sapiens	Surveillance			100	0.03	pass	E_coli_ASM584v2	282	4858432	50.75	41743	pass	98.4155	pass	0
SAMN30414323	SRR21141739	PRJNA861272	*Escherichia coli*	Jay Graham Laboratory	University of California - Berkeley	2021	Quito, Ecuador	feces	Homo sapiens	Surveillance			100	0.14	pass	E_coli_ASM584v2	116	4726214	50.57	103861	pass	99.4709	pass	0
SAMN30414548	SRR21141938	PRJNA861272	*Escherichia coli*	Jay Graham Laboratory	University of California - Berkeley	2021	Quito, Ecuador	feces	Homo sapiens	Surveillance			100	0.02	pass	E_coli_ASM584v2	151	4934384	50.84	71540	pass	97.1696	pass	0
SAMN30414287	SRR21141556	PRJNA861272	*Escherichia coli*	Jay Graham Laboratory	University of California - Berkeley	2021	Quito, Ecuador	feces	Homo sapiens	Surveillance			100	0.1	pass	E_coli_ASM584v2	191	4967399	50.56	62186	pass	98.4156	pass	0
SAMN30414144	SRR21142146	PRJNA514245	*Escherichia coli*	Jay Graham Laboratory	University of California - Berkeley	2021	Quito, Ecuador	feces	Homo sapiens	Surveillance			99.39	0.19	pass	E_coli_ASM584v2	320	4590032	50.97	33676	fail	99.6944	pass	2
SAMN30416088	SRR21143913	PRJNA861272	*Escherichia coli*	Jay Graham Laboratory	University of California - Berkeley	2021	Quito, Ecuador	feces	Homo sapiens	Surveillance			100	1.09	pass	E_coli_ASM584v2	175	5105026	50.93	62929	pass	96.7155	pass	0
SAMN30414528	SRR21141960	PRJNA861272	*Escherichia coli*	Jay Graham Laboratory	University of California - Berkeley	2021	Quito, Ecuador	feces	Homo sapiens	Surveillance			100	0.2	pass	E_coli_ASM584v2	250	5281724	50.38	55227	pass	95.9777	pass	0
SAMN30416433	SRR21143361	PRJNA861272	*Escherichia coli*	Jay Graham Laboratory	University of California - Berkeley	2021	Quito, Ecuador	feces	Homo sapiens	Surveillance			100	0.03	pass	E_coli_ASM584v2	121	4900602	50.8	99997	pass	98.3856	pass	0
SAMN30414025	SRR21142182	PRJNA861272	*Escherichia coli*	Jay Graham Laboratory	University of California - Berkeley	2021	Quito, Ecuador	feces	Homo sapiens	Surveillance			99.94	1.01	pass	E_coli_ASM584v2	930	4777829	50.85	10576	fail	99.2568	pass	1
SAMN30414117	SRR21141776	PRJNA861272	*Escherichia coli*	Jay Graham Laboratory	University of California - Berkeley	2021	Quito, Ecuador	feces	Homo sapiens	Surveillance			100	0	pass	E_coli_ASM584v2	137	4991797	50.73	93379	pass	98.4194	pass	0
SAMN30414348	SRR21141712	PRJNA861272	*Escherichia coli*	Jay Graham Laboratory	University of California - Berkeley	2021	Quito, Ecuador	feces	Homo sapiens	Surveillance			98.13	0	pass	E_coli_ASM584v2	203	4892039	50.87	67747	pass	98.4041	pass	0

ID	SRA Run	Bioproject	Organism	Recollected	Submitter	Collection date	Geo loc name	Isolation source	Host	Purpose of sampling	Lat ion	Hospital	Completeness_General	Contamination	1st Criterion	FastANI Reference	# contigs	Total length	GC (%)	N50	2nd Criterion	FastANI (%)	3rd Criterion	Failed criteria
SAMN30415867	SRR21143430	PRJNA861272	*Escherichia coli*	Jay Graham Laboratory	University of California - Berkeley	2018	Quito, Ecuador	feces	Homo sapiens	Surveillance			100	0.06	pass	E_coli_ASM584v2	148	4779699	50.75	106262	pass	99.4024	pass	0
SAMN30414212	SRR21141766	PRJNA861272	*Escherichia coli*	Jay Graham Laboratory	University of California - Berkeley	2021	Quito, Ecuador	feces	Homo sapiens	Surveillance			100	0.14	pass	E_coli_ASM584v2	192	4701480	51.08	63296	pass	99.2382	pass	0
SAMN30413864	SRR21141948	PRJNA861272	*Escherichia coli*	Jay Graham Laboratory	University of California - Berkeley	2021	Quito, Ecuador	feces	Homo sapiens	Surveillance			100	1.49	pass	E_coli_ASM584v2	187	5233426	50.76	74434	pass	97.8054	pass	0
SAMN30414406	SRR21141999	PRJNA861272	*Escherichia coli*	Jay Graham Laboratory	University of California - Berkeley	2021	Quito, Ecuador	feces	Homo sapiens	Surveillance			100	0.56	pass	E_coli_ASM584v2	110	5318003	50.54	239849	pass	98.3153	pass	0
SAMN30414341	SRR21141719	PRJNA861272	*Escherichia coli*	Jay Graham Laboratory	University of California - Berkeley	2021	Quito, Ecuador	feces	Homo sapiens	Surveillance			100	0.11	pass	E_coli_ASM584v2	274	5007360	50.86	41810	pass	99.2889	pass	0
SAMN30413992	SRR21141818	PRJNA861272	*Escherichia coli*	Jay Graham Laboratory	University of California - Berkeley	2021	Quito, Ecuador	feces	Homo sapiens	Surveillance			100	0.9	pass	E_coli_ASM584v2	359	5484673	50.78	39715	fail	97.8986	pass	1
SAMN30416085	SRR21143917	PRJNA861272	*Escherichia coli*	Jay Graham Laboratory	University of California - Berkeley	2021	Quito, Ecuador	feces	Homo sapiens	Surveillance			100	0.18	pass	E_coli_ASM584v2	225	4970681	50.73	45530	pass	98.4048	pass	0
SAMN30416384	SRR21143807	PRJNA861272	*Escherichia coli*	Jay Graham Laboratory	University of California - Berkeley	2021	Quito, Ecuador	feces	Homo sapiens	Surveillance			100	0.26	pass	E_coli_ASM584v2	113	5095306	50.46	133293	pass	98.2743	pass	0
SAMN30416342	SRR21143492	PRJNA861272	*Escherichia coli*	Jay Graham Laboratory	University of California - Berkeley	2021	Quito, Ecuador	feces	Homo sapiens	Surveillance			100	0.25	pass	E_coli_ASM584v2	114	5222414	50.53	309961	pass	97.2574	pass	0
SAMN30413952	SRR21141447	PRJNA861272	*Escherichia coli*	Jay Graham Laboratory	University of California - Berkeley	2021	Quito, Ecuador	feces	Homo sapiens	Surveillance			99.89	1.34	pass	E_coli_ASM584v2	245	5022395	50.96	39228	fail	96.7975	pass	1
SAMN30413872	SRR21141891	PRJNA861272	*Escherichia coli*	Jay Graham Laboratory	University of California - Berkeley	2021	Quito, Ecuador	feces	Homo sapiens	Surveillance			98.98	0.05	pass	E_coli_ASM584v2	328	5104593	50.63	38469	fail	97.2632	pass	1
SAMN30414213	SRR21141765	PRJNA861272	*Escherichia coli*	Jay Graham Laboratory	University of California - Berkeley	2021	Quito, Ecuador	feces	Homo sapiens	Surveillance			100	0.29	pass	E_coli_ASM584v2	207	5086925	50.74	57706	pass	97.2917	pass	0
SAMN30413796	SRR21142052	PRJNA861272	*Escherichia coli*	Jay Graham Laboratory	University of California - Berkeley	2021	Quito, Ecuador	feces	Homo sapiens	Surveillance			100	0.05	pass	E_coli_ASM584v2	144	4786355	50.41	89287	pass	98.9104	pass	0
SAMN30414047	SRR21141630	PRJNA861272	*Escherichia coli*	Jay Graham Laboratory	University of California - Berkeley	2021	Quito, Ecuador	feces	Homo sapiens	Surveillance			100	0.25	pass	E_coli_ASM584v2	160	5270153	50.41	127551	pass	97.2994	pass	0
SAMN30414720	SRR21141458	PRJNA861272	*Escherichia coli*	Jay Graham Laboratory	University of California - Berkeley	2018	Quito, Ecuador	feces	Homo sapiens	Surveillance			99.86	0.13	pass	E_coli_ASM584v2	163	4981646	50.62	86389	pass	98.4684	pass	0
SAMN30413815	SRR21141628	PRJNA861272	*Escherichia coli*	Jay Graham Laboratory	University of California - Berkeley	2021	Quito, Ecuador	feces	Homo sapiens	Surveillance			98.94	0.57	pass	K_pneumoniae_ASM24018v2	148	5650543	57.25	94275	pass	98.9395	pass	0
SAMN30415837	SRR21143625	PRJNA861272	*Escherichia coli*	Jay Graham Laboratory	University of California - Berkeley	2018	Quito, Ecuador	feces	Homo sapiens	Surveillance			100	0.15	pass	E_coli_ASM584v2	111	4854810	50.61	140459	pass	98.8858	pass	0
SAMN30414439	SRR21142369	PRJNA861272	*Escherichia coli*	Jay Graham Laboratory	University of California - Berkeley	2021	Quito, Ecuador	feces	Homo sapiens	Surveillance			99.88	0.78	pass	E_coli_ASM584v2	262	4886036	51.01	39224	fail	96.9905	pass	1
SAMN30416093	SRR21143908	PRJNA861272	*Escherichia coli*	Jay Graham Laboratory	University of California - Berkeley	2021	Quito, Ecuador	feces	Homo sapiens	Surveillance			100	0.04	pass	E_coli_ASM584v2	306	5029445	50.82	34619	fail	97.157	pass	1
SAMN30416111	SRR21143657	PRJNA861272	*Escherichia coli*	Jay Graham Laboratory	University of California - Berkeley	2021	Quito, Ecuador	feces	Homo sapiens	Surveillance			100	0.05	pass	E_coli_ASM584v2	126	4622185	50.89	62337	pass	97.3912	pass	0
SAMN30416422	SRR21143374	PRJNA861272	*Escherichia coli*	Jay Graham Laboratory	University of California - Berkeley	2021	Quito, Ecuador	feces	Homo sapiens	Surveillance			100	0.43	pass	E_coli_ASM584v2	228	5524973	50.54	111512	pass	97.2835	pass	0
SAMN30414170	SRR21141589	PRJNA861272	*Escherichia coli*	Jay Graham Laboratory	University of California - Berkeley	2021	Quito, Ecuador	feces	Homo sapiens	Surveillance			100	0.11	pass	E_coli_ASM584v2	339	5348123	50.6	43210	pass	97.2047	pass	0
SAMN30416094	SRR21143907	PRJNA861272	*Escherichia coli*	Jay Graham Laboratory	University of California - Berkeley	2021	Quito, Ecuador	feces	Homo sapiens	Surveillance			100	0.34	pass	E_coli_ASM584v2	273	5150088	50.78	40271	pass	98.3004	pass	0
SAMN30416430	SRR21143364	PRJNA861272	*Escherichia coli*	Jay Graham Laboratory	University of California - Berkeley	2021	Quito, Ecuador	feces	Homo sapiens	Surveillance			100	0.11	pass	E_coli_ASM584v2	150	5110705	50.65	128641	pass	98.4126	pass	0

ID	SRA Run	Bioproject	Organism	Recollected	Submmiter	Collection date	Geo loc name	Isolation source	Host	Purpose of sampling	Lat ion	Hospital	Completeness_General	Contamination	1st Criterion	FastANI Reference	# contigs	Total length	GC (%)	N50	2nd Criterion	FastANI (%)	3rd Criterion	Failed criteria
SAMN30414671	SRR21142240	PRJNA861272	*Escherichia coli*	Jay Graham Laboratory	University of California - Berkeley	2018	Quito, Ecuador	feces	Homo sapiens	Surveillance			100	0.2	pass	E_coli_ASM584v2	123	5253645	50.77	204355	pass	97.1058	pass	0
SAMN30416119	SRR21143648	PRJNA861272	*Escherichia coli*	Jay Graham Laboratory	University of California - Berkeley	2021	Quito, Ecuador	feces	Homo sapiens	Surveillance			100	0.25	pass	E_coli_ASM584v2	128	4682461	50.76	105795	pass	99.102	pass	0
SAMN30414713	SRR21141466	PRJNA861272	*Escherichia coli*	Jay Graham Laboratory	University of California - Berkeley	2018	Quito, Ecuador	feces	Homo sapiens	Surveillance			100	0.34	pass	E_coli_ASM584v2	156	5001384	50.52	88907	pass	99.1643	pass	0
SAMN30414134	SRR21142157	PRJNA861272	*Escherichia coli*	Jay Graham Laboratory	University of California - Berkeley	2021	Quito, Ecuador	feces	Homo sapiens	Surveillance			100	0.56	pass	E_coli_ASM584v2	171	5135451	50.61	92628	pass	98.86	pass	0
SAMN30414328	SRR21141734	PRJNA861272	*Escherichia coli*	Jay Graham Laboratory	University of California - Berkeley	2021	Quito, Ecuador	feces	Homo sapiens	Surveillance			100	0.8	pass	E_coli_ASM584v2	320	5011119	50.81	43434	pass	99.1756	pass	0
SAMN30414179	SRR21141387	PRJNA861272	*Escherichia coli*	Jay Graham Laboratory	University of California - Berkeley	2021	Quito, Ecuador	feces	Homo sapiens	Surveillance			100	0.36	pass	E_coli_ASM584v2	179	5668495	50.58	127386	pass	97.9482	pass	0
SAMN30414225	SRR21141752	PRJNA861272	*Escherichia coli*	Jay Graham Laboratory	University of California - Berkeley	2021	Quito, Ecuador	feces	Homo sapiens	Surveillance			98.14	0.47	pass	E_coli_ASM584v2	242	4483577	50.96	38210	fail	98.4823	pass	1
SAMN30413949	SRR21141450	PRJNA861272	*Escherichia coli*	Jay Graham Laboratory	University of California - Berkeley	2021	Quito, Ecuador	feces	Homo sapiens	Surveillance			95.51	1.29	pass	E_coli_ASM584v2	415	4915166	50.88	33469	fail	99.5259	pass	1
SAMN30416383	SRR21143385	PRJNA861272	*Escherichia coli*	Jay Graham Laboratory	University of California - Berkeley	2021	Quito, Ecuador	feces	Homo sapiens	Surveillance			99.78	0.02	pass	E_coli_ASM584v2	43	4882051	51.96	388440	pass	82.9703	fail	1
SAMN30414432	SRR21141970	PRJNA861272	*Escherichia coli*	Jay Graham Laboratory	University of California - Berkeley	2021	Quito, Ecuador	feces	Homo sapiens	Surveillance			100	0	pass	E_coli_ASM584v2	293	4867136	50.96	35543	fail	97.3604	pass	1
SAMN30414316	SRR21142003	PRJNA861272	*Escherichia coli*	Jay Graham Laboratory	University of California - Berkeley	2021	Quito, Ecuador	feces	Homo sapiens	Surveillance			98.99	0.08	pass	E_coli_ASM584v2	218	4994139	50.75	51542	pass	98.3669	pass	0
SAMN30416414	SRR21143383	PRJNA861272	*Escherichia coli*	Jay Graham Laboratory	University of California - Berkeley	2021	Quito, Ecuador	feces	Homo sapiens	Surveillance			100	0.28	pass	E_coli_ASM584v2	66	5158428	50.74	163574	pass	98.4255	pass	0
SAMN30413910	SRR21141685	PRJNA861272	*Escherichia coli*	Jay Graham Laboratory	University of California - Berkeley	2021	Quito, Ecuador	feces	Homo sapiens	Surveillance			99.89	0.63	pass	E_coli_ASM584v2	220	5049423	50.76	55428	pass	96.7432	pass	0
SAMN30413800	SRR21141698	PRJNA861272	*Escherichia coli*	Jay Graham Laboratory	University of California - Berkeley	2021	Quito, Ecuador	feces	Homo sapiens	Surveillance			75.38	2.82	fail	E_coli_ASM584v2	1855	3540090	50.16	2875	fail	98.4441	pass	2
SAMN30416409	SRR21143779	PRJNA861272	*Escherichia coli*	Jay Graham Laboratory	University of California - Berkeley	2021	Quito, Ecuador	feces	Homo sapiens	Surveillance			99.93	0.95	pass	E_coli_ASM584v2	147	5157047	50.63	140350	pass	96.7271	pass	0
SAMN30416368	SRR21143401	PRJNA861272	*Escherichia coli*	Jay Graham Laboratory	University of California - Berkeley	2021	Quito, Ecuador	feces	Homo sapiens	Surveillance			100	0.1	pass	E_coli_ASM584v2	202	5104214	50.61	60605	pass	98.371	pass	0
SAMN30416400	SRR21143789	PRJNA861272	*Escherichia coli*	Jay Graham Laboratory	University of California - Berkeley	2021	Quito, Ecuador	feces	Homo sapiens	Surveillance			100	0.21	pass	E_coli_ASM584v2	201	5020468	50.27	69336	pass	99.514	pass	0
SAMN30415866	SRR21143442	PRJNA861272	*Escherichia coli*	Jay Graham Laboratory	University of California - Berkeley	2018	Quito, Ecuador	feces	Homo sapiens	Surveillance			100	0.06	pass	E_coli_ASM584v2	142	4767786	50.74	106262	pass	99.4339	pass	0
SAMN30414174	SRR21141585	PRJNA861272	*Escherichia coli*	Jay Graham Laboratory	University of California - Berkeley	2021	Quito, Ecuador	feces	Homo sapiens	Surveillance			100	0.32	pass	E_coli_ASM584v2	308	5376115	50.81	51282	pass	98.8149	pass	0
SAMN30414364	SRR21142094	PRJNA861272	*Escherichia coli*	Jay Graham Laboratory	University of California - Berkeley	2021	Quito, Ecuador	feces	Homo sapiens	Surveillance			100	0.02	pass	E_coli_ASM584v2	310	4742398	50.89	32571	fail	99.4754	pass	1
SAMN30416370	SRR21143399	PRJNA861272	*Escherichia coli*	Jay Graham Laboratory	University of California - Berkeley	2021	Quito, Ecuador	feces	Homo sapiens	Surveillance			99.91	0.11	pass	E_coli_ASM584v2	204	5170203	50.68	67225	pass	98.4165	pass	0
SAMN30414192	SRR21142044	PRJNA861272	*Escherichia coli*	Jay Graham Laboratory	University of California - Berkeley	2021	Quito, Ecuador	feces	Homo sapiens	Surveillance			100	0.01	pass	E_coli_ASM584v2	136	4937071	50.6	95931	pass	97.2343	pass	0
SAMN30414087	SRR21141394	PRJNA861272	*Escherichia coli*	Jay Graham Laboratory	University of California - Berkeley	2021	Quito, Ecuador	feces	Homo sapiens	Surveillance			100	0.33	pass	E_coli_ASM584v2	304	5652077	50.66	46576	pass	97.2377	pass	0
SAMN30414152	SRR21141609	PRJNA861272	*Escherichia coli*	Jay Graham Laboratory	University of California - Berkeley	2021	Quito, Ecuador	feces	Homo sapiens	Surveillance			100	0.16	pass	E_coli_ASM584v2	201	5420579	50.57	73474	pass	97.2926	pass	0

ID	SRA Run	Bioproject	Organism	Recollected	Submmiter	Collection date	Geo loc name	Isolation source	Host	Purpose of sampling	Lat ion	Hospital	Completeness_General	Contamination	1st Criterion	FastANI Reference	# contigs	Total length	GC (%)	N50	2nd Criterion	FastANI (%)	3rd Criterion	Failed criteria
SAMN30414009	SRR21142199	PRJNA861272	*Escherichia coli*	Jay Graham Laboratory	University of California - Berkeley	2021	Quito, Ecuador	feces	Homo sapiens	Surveillance			100	0.13	pass	E_coli_ASM584v2	378	5244381	50.66	35656	fail	98.8792	pass	1
SAMN30414322	SRR21141740	PRJNA861272	*Escherichia coli*	Jay Graham Laboratory	University of California - Berkeley	2021	Quito, Ecuador	feces	Homo sapiens	Surveillance			100	0.03	pass	E_coli_ASM584v2	164	4890107	50.74	74129	pass	98.4959	pass	0
SAMN30414302	SRR21142018	PRJNA861272	*Escherichia coli*	Jay Graham Laboratory	University of California - Berkeley	2021	Quito, Ecuador	feces	Homo sapiens	Surveillance			100	0.33	pass	E_coli_ASM584v2	152	4841559	50.87	66952	pass	99.3227	pass	0
SAMN30414669	SRR21142242	PRJNA861272	*Escherichia coli*	Jay Graham Laboratory	University of California - Berkeley	2018	Quito, Ecuador	feces	Homo sapiens	Surveillance			100	0.17	pass	E_coli_ASM584v2	198	5179626	50.74	106754	pass	97.0502	pass	0
SAMN30413918	SRR21141677	PRJNA861272	*Escherichia coli*	Jay Graham Laboratory	University of California - Berkeley	2021	Quito, Ecuador	feces	Homo sapiens	Surveillance			100	0.31	pass	E_coli_ASM584v2	201	5240039	50.66	115495	pass	97.1141	pass	0
SAMN30414160	SRR21141600	PRJNA861272	*Escherichia coli*	Jay Graham Laboratory	University of California - Berkeley	2021	Quito, Ecuador	feces	Homo sapiens	Surveillance			98.55	0.03	pass	E_coli_ASM584v2	298	5148945	50.65	44331	pass	97.208	pass	0
SAMN30416381	SRR21143387	PRJNA861272	*Escherichia coli*	Jay Graham Laboratory	University of California - Berkeley	2021	Quito, Ecuador	feces	Homo sapiens	Surveillance			99.55	0.06	pass	E_coli_ASM584v2	193	4422720	51.03	46889	pass	98.4732	pass	0
SAMN30414368	SRR21142090	PRJNA861272	*Escherichia coli*	Jay Graham Laboratory	University of California - Berkeley	2021	Quito, Ecuador	feces	Homo sapiens	Surveillance			84.15	89.59	fail	E_coli_ASM584v2	3037	13969349	52.1	6725	fail	95.5723	pass	2
SAMN30414132	SRR21142159	PRJNA861272	*Escherichia coli*	Jay Graham Laboratory	University of California - Berkeley	2021	Quito, Ecuador	feces	Homo sapiens	Surveillance			100	0.2	pass	E_coli_ASM584v2	195	4779707	50.73	63040	pass	99.1949	pass	0
SAMN30414197	SRR21142039	PRJNA861272	*Escherichia coli*	Jay Graham Laboratory	University of California - Berkeley	2021	Quito, Ecuador	feces	Homo sapiens	Surveillance			100	0.09	pass	E_coli_ASM584v2	267	5167265	50.6	38367	fail	98.3632	pass	1
SAMN30413799	SRR21141709	PRJNA861272	*Escherichia coli*	Jay Graham Laboratory	University of California - Berkeley	2021	Quito, Ecuador	feces	Homo sapiens	Surveillance			100	0.31	pass	E_coli_ASM584v2	85	4655569	50.66	143451	pass	99.3438	pass	0
SAMN30413839	SRR21141553	PRJNA861272	*Escherichia coli*	Jay Graham Laboratory	University of California - Berkeley	2021	Quito, Ecuador	feces	Homo sapiens	Surveillance			100	0.07	pass	E_coli_ASM584v2	85	4793527	50.62	173226	pass	98.393	pass	0
SAMN30414311	SRR21142008	PRJNA861272	*Escherichia coli*	Jay Graham Laboratory	University of California - Berkeley	2021	Quito, Ecuador	feces	Homo sapiens	Surveillance			100	0.71	pass	E_coli_ASM584v2	220	5658451	50.73	50572	pass	98.0273	pass	0
SAMN30414079	SRR21141402	PRJNA861272	*Escherichia coli*	Jay Graham Laboratory	University of California - Berkeley	2021	Quito, Ecuador	feces	Homo sapiens	Surveillance			100	0.48	pass	E_coli_ASM584v2	133	5201918	50.6	128117	pass	97.8612	pass	0
SAMN30414580	SRR21142341	PRJNA861272	*Escherichia coli*	Jay Graham Laboratory	University of California - Berkeley	2018	Quito, Ecuador	feces	Homo sapiens	Surveillance			100	0.02	pass	E_coli_ASM584v2	208	4995074	50.58	65002	pass	97.2844	pass	0
SAMN30413983	SRR21141829	PRJNA861272	*Escherichia coli*	Jay Graham Laboratory	University of California - Berkeley	2021	Quito, Ecuador	feces	Homo sapiens	Surveillance			100	0.28	pass	E_coli_ASM584v2	167	4891614	50.65	72092	pass	99.0459	pass	0
SAMN30414459	SRR21142347	PRJNA861272	*Escherichia coli*	Jay Graham Laboratory	University of California - Berkeley	2021	Quito, Ecuador	feces	Homo sapiens	Surveillance			99.89	0.77	pass	E_coli_ASM584v2	228	5694019	50.57	89751	pass	97.1481	pass	0
SAMN30416455	SRR21143728	PRJNA861272	*Escherichia coli*	Jay Graham Laboratory	University of California - Berkeley	2021	Quito, Ecuador	feces	Homo sapiens	Surveillance			99.75	0.31	pass	Hafnia paralvei_ASM231314v2	96	4812809	48.13	83645	fail	99.0166	fail	1
SAMN30415827	SRR21143637	PRJNA861272	*Escherichia coli*	Jay Graham Laboratory	University of California - Berkeley	2018	Quito, Ecuador	feces	Homo sapiens	Surveillance			100	0.39	pass	E_coli_ASM584v2	298	5222916	50.54	52093	pass	97.2839	pass	0
SAMN30413908	SRR21141688	PRJNA861272	*Escherichia coli*	Jay Graham Laboratory	University of California - Berkeley	2021	Quito, Ecuador	feces	Homo sapiens	Surveillance			100	0.44	pass	E_coli_ASM584v2	308	5497376	50.79	51262	pass	97.2579	pass	0
SAMN30414527	SRR21141961	PRJNA861272	*Escherichia coli*	Jay Graham Laboratory	University of California - Berkeley	2021	Quito, Ecuador	feces	Homo sapiens	Surveillance			100	1.09	pass	E_coli_ASM584v2	192	5062913	50.88	87195	pass	96.7713	pass	0
SAMN30414396	SRR21141530	PRJNA861272	*Escherichia coli*	Jay Graham Laboratory	University of California - Berkeley	2021	Quito, Ecuador	feces	Homo sapiens	Surveillance			100	0	pass	E_coli_ASM584v2	99	5060178	50.62	167854	pass	98.4201	pass	0
SAMN30414662	SRR21142282	PRJNA861272	*Escherichia coli*	Jay Graham Laboratory	University of California - Berkeley	2018	Quito, Ecuador	feces	Homo sapiens	Surveillance			99.91	0.09	pass	E_coli_ASM584v2	146	5166356	50.61	113827	pass	97.1983	pass	0
SAMN30413961	SRR21141437	PRJNA861272	*Escherichia coli*	Jay Graham Laboratory	University of California - Berkeley	2021	Quito, Ecuador	feces	Homo sapiens	Surveillance			99.83	0.02	pass	E_coli_ASM584v2	352	5014648	50.85	39352	fail	99.5459	pass	1

ID	SRA Run	Bioproject	Organism	Recollected	Submitter	Collection date	Geo loc name	Isolation source	Host	Purpose of sampling	Lat ion	Hospital	Completeness_General	Contamination	1st Criterion	FastANI Reference	# contigs	Total length	GC (%)	N50	2nd Criterion	FastANI (%)	3rd Criterion	Failed criteria
SAMN30414474	SRR21142068	PRJNA514245	*Escherichia coli*	Jay Graham Laboratory	University of California - Berkeley	2021	Quito, Ecuador	feces	Homo sapiens	Surveillance			100	0.06	pass	E_coli_ASM584v2	122	4982097	50.91	90853	pass	98.4159	pass	0
SAMN30415865	SRR21143843	PRJNA861272	*Escherichia coli*	Jay Graham Laboratory	University of California - Berkeley	2018	Quito, Ecuador	feces	Homo sapiens	Surveillance			96.37	0.09	pass	E_coli_ASM584v2	274	4859494	50.41	41322	pass	99.5168	pass	0
SAMN30416423	SRR21143373	PRJNA861272	*Escherichia coli*	Jay Graham Laboratory	University of California - Berkeley	2021	Quito, Ecuador	feces	Homo sapiens	Surveillance			100	0.1	pass	E_coli_ASM584v2	86	4885923	50.91	154414	pass	98.3628	pass	0
SAMN30415826	SRR21143638	PRJNA861272	*Escherichia coli*	Jay Graham Laboratory	University of California - Berkeley	2018	Quito, Ecuador	feces	Homo sapiens	Surveillance			96.69	0.03	pass	E_coli_ASM584v2	149	5171671	50.53	129510	pass	97.3182	pass	0
SAMN30414526	SRR21141962	PRJNA861272	*Escherichia coli*	Jay Graham Laboratory	University of California - Berkeley	2021	Quito, Ecuador	feces	Homo sapiens	Surveillance			100	1.6	pass	E_coli_ASM584v2	221	5187157	50.79	60355	pass	97.9862	pass	0
SAMN30416456	SRR21143727	PRJNA861272	*Escherichia coli*	Jay Graham Laboratory	University of California - Berkeley	2021	Quito, Ecuador	feces	Homo sapiens	Surveillance			100	1.72	pass	E_coli_ASM584v2	172	5420282	50.48	103622	pass	97.163	pass	0
SAMN30414143	SRR21142147	PRJNA861272	*Escherichia coli*	Jay Graham Laboratory	University of California - Berkeley	2021	Quito, Ecuador	feces	Homo sapiens	Surveillance			8.3	0.01	fail	E_coli_ASM584v2	395	622192	45.27	1568	fail	98.7436	pass	2
SAMN30414273	SRR21141571	PRJNA861272	*Escherichia coli*	Jay Graham Laboratory	University of California - Berkeley	2021	Quito, Ecuador	feces	Homo sapiens	Surveillance			96.57	0.54	pass	E_coli_ASM584v2	238	4959641	50.95	45057	pass	96.8057	pass	0
SAMN30413838	SRR21141565	PRJNA861272	*Escherichia coli*	Jay Graham Laboratory	University of California - Berkeley	2021	Quito, Ecuador	feces	Homo sapiens	Surveillance			99.9	0.8	pass	E_coli_ASM584v2	95	5031602	50.64	171702	pass	96.8578	pass	0
SAMN30414712	SRR21141467	PRJNA861272	*Escherichia coli*	Jay Graham Laboratory	University of California - Berkeley	2018	Quito, Ecuador	feces	Homo sapiens	Surveillance			100	0.33	pass	E_coli_ASM584v2	158	5007958	50.52	98325	pass	99.1536	pass	0
SAMN30414589	SRR21142330	PRJNA861272	*Escherichia coli*	Jay Graham Laboratory	University of California - Berkeley	2018	Quito, Ecuador	feces	Homo sapiens	Surveillance			100	0.05	pass	E_coli_ASM584v2	244	5297923	50.38	80600	pass	97.1573	pass	0
SAMN30416444	SRR21143740	PRJNA861272	*Escherichia coli*	Jay Graham Laboratory	University of California - Berkeley	2021	Quito, Ecuador	feces	Homo sapiens	Surveillance			100	0.11	pass	E_coli_ASM584v2	116	4814039	50.76	111017	pass	99.1254	pass	0
SAMN30413912	SRR21141683	PRJNA861272	*Escherichia coli*	Jay Graham Laboratory	University of California - Berkeley	2021	Quito, Ecuador	feces	Homo sapiens	Surveillance			100	0.3	pass	E_coli_ASM584v2	85	5114252	51.77	164211	pass	82.4853	fail	1
SAMN30416432	SRR21143362	PRJNA861272	*Escherichia coli*	Jay Graham Laboratory	University of California - Berkeley	2021	Quito, Ecuador	feces	Homo sapiens	Surveillance			100	0.09	pass	E_coli_ASM584v2	140	4809731	50.81	73795	pass	98.3784	pass	0
SAMN30415863	SRR21143865	PRJNA861272	*Escherichia coli*	Jay Graham Laboratory	University of California - Berkeley	2018	Quito, Ecuador	feces	Homo sapiens	Surveillance			100	0.2	pass	E_coli_ASM584v2	132	4920458	50.41	172787	pass	99.4872	pass	0
SAMN30414093	SRR21141802	PRJNA861272	*Escherichia coli*	Jay Graham Laboratory	University of California - Berkeley	2021	Quito, Ecuador	feces	Homo sapiens	Surveillance			100	0.65	pass	E_coli_ASM584v2	518	5435246	50.2	31496	fail	98.323	pass	1
SAMN30414244	SRR21142131	PRJNA514245	*Escherichia coli*	Jay Graham Laboratory	University of California - Berkeley	2021	Quito, Ecuador	feces	Homo sapiens	Surveillance			100	0.04	pass	E_coli_ASM584v2	236	5329965	50.68	78059	pass	97.1393	pass	0
SAMN30414136	SRR21142155	PRJNA861272	*Escherichia coli*	Jay Graham Laboratory	University of California - Berkeley	2021	Quito, Ecuador	feces	Homo sapiens	Surveillance			100	0.02	pass	E_coli_ASM584v2	84	4797331	50.61	131791	pass	98.3994	pass	0
SAMN30414524	SRR21141964	PRJNA861272	*Escherichia coli*	Jay Graham Laboratory	University of California - Berkeley	2021	Quito, Ecuador	feces	Homo sapiens	Surveillance			100	0.09	pass	E_coli_ASM584v2	287	5175315	50.66	72432	pass	99.0524	pass	0
SAMN30414034	SRR21141644	PRJNA861272	*Escherichia coli*	Jay Graham Laboratory	University of California - Berkeley	2021	Quito, Ecuador	feces	Homo sapiens	Surveillance			100	0.26	pass	E_coli_ASM584v2	145	4990883	50.54	125514	pass	99.0032	pass	0
SAMN30415862	SRR21143454	PRJNA861272	*Escherichia coli*	Jay Graham Laboratory	University of California - Berkeley	2018	Quito, Ecuador	feces	Homo sapiens	Surveillance			100	0.18	pass	E_coli_ASM584v2	148	4911253	50.41	131269	pass	99.5117	pass	0
SAMN30414585	SRR21142336	PRJNA861272	*Escherichia coli*	Jay Graham Laboratory	University of California - Berkeley	2018	Quito, Ecuador	feces	Homo sapiens	Surveillance			100	0.05	pass	E_coli_ASM584v2	159	5364676	50.35	146148	pass	97.2203	pass	0
SAMN30414372	SRR21142085	PRJNA861272	*Escherichia coli*	Jay Graham Laboratory	University of California - Berkeley	2021	Quito, Ecuador	feces	Homo sapiens	Surveillance			100	0.08	pass	E_coli_ASM584v2	288	5199282	50.83	42854	pass	99.4545	pass	0
SAMN30414195	SRR21142041	PRJNA861272	*Escherichia coli*	Jay Graham Laboratory	University of California - Berkeley	2021	Quito, Ecuador	feces	Homo sapiens	Surveillance			99.91	0.21	pass	E_coli_ASM584v2	331	5169920	50.71	27704	fail	97.1266	pass	1

ID	SRA Run	Bioproject	Organism	Recollected	Submitter	Collection date	Geo loc name	Isolation source	Host	Purpose of sampling	Lat ion	Hospital	Completeness_General	Contamination	1st Criterion	FastANI Reference	# contigs	Total length	GC (%)	N50	2nd Criterion	FastANI (%)	3rd Criterion	Failed criteria
SAMN30416072	SRR21143931	PRJNA861272	*Escherichia coli*	Jay Graham Laboratory	University of California - Berkeley	2021	Quito, Ecuador	feces	Homo sapiens	Surveillance			100	0.4	pass	E_coli_ASM584v2	243	5322686	50.7	55208	pass	97.2214	pass	0
SAMN30414410	SRR21141994	PRJNA861272	*Escherichia coli*	Jay Graham Laboratory	University of California - Berkeley	2021	Quito, Ecuador	feces	Homo sapiens	Surveillance			100	0.15	pass	E_coli_ASM584v2	200	4815018	50.76	48415	pass	99.2107	pass	0
SAMN30414003	SRR21142206	PRJNA861272	*Escherichia coli*	Jay Graham Laboratory	University of California - Berkeley	2021	Quito, Ecuador	feces	Homo sapiens	Surveillance			100	0.12	pass	E_coli_ASM584v2	84	5118186	50.45	167374	pass	98.3587	pass	0
SAMN30416424	SRR21143372	PRJNA861272	*Escherichia coli*	Jay Graham Laboratory	University of California - Berkeley	2021	Quito, Ecuador	feces	Homo sapiens	Surveillance			100	0.1	pass	E_coli_ASM584v2	135	4804076	50.88	89955	pass	99.3411	pass	0
SAMN30414155	SRR21141606	PRJNA861272	*Escherichia coli*	Jay Graham Laboratory	University of California - Berkeley	2021	Quito, Ecuador	feces	Homo sapiens	Surveillance			99.9	0.79	pass	E_coli_ASM584v2	153	4939402	50.82	82781	pass	96.9058	pass	0
SAMN30414121	SRR21142171	PRJNA861272	*Escherichia coli*	Jay Graham Laboratory	University of California - Berkeley	2021	Quito, Ecuador	feces	Homo sapiens	Surveillance			100	0.39	pass	E_coli_ASM584v2	185	5029791	50.49	55087	pass	97.2167	pass	0
SAMN30414479	SRR21142062	PRJNA514245	*Escherichia coli*	Jay Graham Laboratory	University of California - Berkeley	2021	Quito, Ecuador	feces	Homo sapiens	Surveillance			99.05	0.42	pass	E_coli_ASM584v2	266	4788828	50.84	40990	pass	99.2067	pass	0
SAMN30416431	SRR21143363	PRJNA861272	*Escherichia coli*	Jay Graham Laboratory	University of California - Berkeley	2021	Quito, Ecuador	feces	Homo sapiens	Surveillance			100	0.03	pass	E_coli_ASM584v2	260	5150505	50.71	75249	pass	99.4515	pass	0
SAMN30414296	SRR21142025	PRJNA861272	*Escherichia coli*	Jay Graham Laboratory	University of California - Berkeley	2021	Quito, Ecuador	feces	Homo sapiens	Surveillance			100	0.07	pass	E_coli_ASM584v2	160	4670933	50.86	57368	pass	98.5412	pass	0
SAMN30413817	SRR21141414	PRJNA861272	*Escherichia coli*	Jay Graham Laboratory	University of California - Berkeley	2021	Quito, Ecuador	feces	Homo sapiens	Surveillance			100	0.08	pass	E_coli_ASM584v2	246	5029031	50.83	57943	pass	98.4021	pass	0
SAMN30414358	SRR21142101	PRJNA861272	*Escherichia coli*	Jay Graham Laboratory	University of California - Berkeley	2021	Quito, Ecuador	feces	Homo sapiens	Surveillance			100	0.33	pass	E_coli_ASM584v2	293	5067708	50.71	34462	fail	98.3223	pass	1
SAMN30414291	SRR21142030	PRJNA861272	*Escherichia coli*	Jay Graham Laboratory	University of California - Berkeley	2021	Quito, Ecuador	feces	Homo sapiens	Surveillance			100	0.14	pass	E_coli_ASM584v2	309	4667828	50.96	34749	fail	99.0467	pass	1
SAMN30414091	SRR21141804	PRJNA861272	*Escherichia coli*	Jay Graham Laboratory	University of California - Berkeley	2021	Quito, Ecuador	feces	Homo sapiens	Surveillance			99.85	0.75	pass	E_coli_ASM584v2	195	4968628	50.76	57350	pass	96.851	pass	0
SAMN30416391	SRR21143799	PRJNA861272	*Escherichia coli*	Jay Graham Laboratory	University of California - Berkeley	2021	Quito, Ecuador	feces	Homo sapiens	Surveillance			100	1.79	pass	E_coli_ASM584v2	115	5051752	50.77	123597	pass	96.7679	pass	0
SAMN30414028	SRR21142179	PRJNA861272	*Escherichia coli*	Jay Graham Laboratory	University of California - Berkeley	2021	Quito, Ecuador	feces	Homo sapiens	Surveillance			100	0.1	pass	E_coli_ASM584v2	180	4875982	50.59	65495	pass	99.2829	pass	0
SAMN30416378	SRR21143390	PRJNA861272	*Escherichia coli*	Jay Graham Laboratory	University of California - Berkeley	2021	Quito, Ecuador	feces	Homo sapiens	Surveillance			100	0.12	pass	E_coli_ASM584v2	137	5115808	50.58	114896	pass	95.8576	pass	0
SAMN30414026	SRR21142181	PRJNA861272	*Escherichia coli*	Jay Graham Laboratory	University of California - Berkeley	2021	Quito, Ecuador	feces	Homo sapiens	Surveillance			98.51	0.49	pass	E_coli_ASM584v2	365	4902961	50.91	33322	fail	97.353	pass	1
SAMN30414380	SRR21141548	PRJNA861272	*Escherichia coli*	Jay Graham Laboratory	University of California - Berkeley	2021	Quito, Ecuador	feces	Homo sapiens	Surveillance			98.93	0.01	pass	E_coli_ASM584v2	316	4816727	50.98	30461	fail	97.3286	pass	1
SAMN30414779	SRR21142248	PRJNA861272	*Escherichia coli*	Jay Graham Laboratory	University of California - Berkeley	2018	Quito, Ecuador	feces	Homo sapiens	Surveillance			100	0.66	pass	E_coli_ASM584v2	130	5255365	50.52	178195	pass	97.8594	pass	0
SAMN30414068	SRR21141415	PRJNA861272	*Escherichia coli*	Jay Graham Laboratory	University of California - Berkeley	2021	Quito, Ecuador	feces	Homo sapiens	Surveillance			99.8	0.74	pass	E_coli_ASM584v2	157	5244854	50.73	104857	pass	96.7304	pass	0
SAMN30414157	SRR21141604	PRJNA861272	*Escherichia coli*	Jay Graham Laboratory	University of California - Berkeley	2021	Quito, Ecuador	feces	Homo sapiens	Surveillance			100	0.12	pass	E_coli_ASM584v2	191	5397854	50.64	87723	pass	97.1627	pass	0
SAMN30416371	SRR21143398	PRJNA861272	*Escherichia coli*	Jay Graham Laboratory	University of California - Berkeley	2021	Quito, Ecuador	feces	Homo sapiens	Surveillance			100	0.61	pass	E_coli_ASM584v2	247	5385657	50.42	134589	pass	97.2277	pass	0
SAMN30414159	SRR21141601	PRJNA861272	*Escherichia coli*	Jay Graham Laboratory	University of California - Berkeley	2021	Quito, Ecuador	feces	Homo sapiens	Surveillance			100	1.34	pass	E_coli_ASM584v2	232	5425434	50.8	51790	pass	97.8953	pass	0
SAMN30414385	SRR21141543	PRJNA861272	*Escherichia coli*	Jay Graham Laboratory	University of California - Berkeley	2021	Quito, Ecuador	feces	Homo sapiens	Surveillance			100	0.14	pass	E_coli_ASM584v2	262	4756538	50.99	35479	fail	98.419	pass	1

ID	SRA Run	Bioproject	Organism	Recollected	Submitter	Collection date	Geo loc name	Isolation source	Host	Purpose of sampling	Lat ion	Hospital	Completeness_General	Contamination	1st Criterion	FastANI Reference	# contigs	Total length	GC (%)	N50	2nd Criterion	FastANI (%)	3rd Criterion	Failed criteria
SAMN30414379	SRR21141549	PRJNA861272	*Escherichia coli*	Jay Graham Laboratory	University of California - Berkeley	2021	Quito, Ecuador	feces	Homo sapiens	Surveillance			100	0.26	pass	E_coli_ASM584v2	209	4725308	50.68	40642	pass	98.8297	pass	0
SAMN30416356	SRR21143415	PRJNA861272	*Escherichia coli*	Jay Graham Laboratory	University of California - Berkeley	2021	Quito, Ecuador	feces	Homo sapiens	Surveillance			100	0.05	pass	E_coli_ASM584v2	295	5046311	50.56	43636	pass	98.3057	pass	0
SAMN30414330	SRR21141731	PRJNA861272	*Escherichia coli*	Jay Graham Laboratory	University of California - Berkeley	2021	Quito, Ecuador	feces	Homo sapiens	Surveillance			100	0.06	pass	E_coli_ASM584v2	232	4638945	50.84	52955	pass	99.2667	pass	0
SAMN30414006	SRR21142203	PRJNA861272	*Escherichia coli*	Jay Graham Laboratory	University of California - Berkeley	2021	Quito, Ecuador	feces	Homo sapiens	Surveillance			99.94	0.07	pass	E_coli_ASM584v2	286	5406891	50.61	44885	pass	97.2262	pass	0
SAMN30413882	SRR21141460	PRJNA861272	*Escherichia coli*	Jay Graham Laboratory	University of California - Berkeley	2021	Quito, Ecuador	feces	Homo sapiens	Surveillance			100	0.13	pass	E_coli_ASM584v2	110	5042640	50.63	163104	pass	98.4416	pass	0
SAMN30413948	SRR21141452	PRJNA861272	*Escherichia coli*	Jay Graham Laboratory	University of California - Berkeley	2021	Quito, Ecuador	feces	Homo sapiens	Surveillance			99.92	0.11	pass	E_coli_ASM584v2	344	4978899	50.73	35565	fail	98.3736	pass	1
SAMN30413858	SRR21142063	PRJNA861272	*Escherichia coli*	Jay Graham Laboratory	University of California - Berkeley	2021	Quito, Ecuador	feces	Homo sapiens	Surveillance			99.95	0.05	pass	E_coli_ASM584v2	107	4785638	55.31	104870	pass	81.7913	fail	1
SAMN30414035	SRR21141643	PRJNA861272	*Escherichia coli*	Jay Graham Laboratory	University of California - Berkeley	2021	Quito, Ecuador	feces	Homo sapiens	Surveillance			100	0	pass	E_coli_ASM584v2	115	5166692	50.55	123043	pass	98.4019	pass	0
SAMN30413833	SRR21141748	PRJNA861272	*Escherichia coli*	Jay Graham Laboratory	University of California - Berkeley	2021	Quito, Ecuador	feces	Homo sapiens	Surveillance			100	0.13	pass	E_coli_ASM584v2	87	4789019	50.6	146138	pass	98.4221	pass	0
SAMN30416408	SRR21143780	PRJNA861272	*Escherichia coli*	Jay Graham Laboratory	University of California - Berkeley	2021	Quito, Ecuador	feces	Homo sapiens	Surveillance			100	0.01	pass	E_coli_ASM584v2	189	5109840	50.88	90911	pass	99.4795	pass	0
SAMN30416462	SRR21143720	PRJNA861272	*Escherichia coli*	Jay Graham Laboratory	University of California - Berkeley	2021	Quito, Ecuador	feces	Homo sapiens	Surveillance			100	0.07	pass	E_coli_ASM584v2	104	4651272	50.85	114316	pass	99.5798	pass	0
SAMN30414285	SRR21141558	PRJNA861272	*Escherichia coli*	Jay Graham Laboratory	University of California - Berkeley	2021	Quito, Ecuador	feces	Homo sapiens	Surveillance			100	1.76	pass	E_coli_ASM584v2	205	5260806	50.65	64360	pass	97.8364	pass	0
SAMN30414007	SRR21142202	PRJNA861272	*Escherichia coli*	Jay Graham Laboratory	University of California - Berkeley	2021	Quito, Ecuador	feces	Homo sapiens	Surveillance			99.32	0.14	pass	E_coli_ASM584v2	367	4790939	51.12	34822	fail	97.3634	pass	1
SAMN30413878	SRR21142232	PRJNA861272	*Escherichia coli*	Jay Graham Laboratory	University of California - Berkeley	2021	Quito, Ecuador	feces	Homo sapiens	Surveillance			100	0.1	pass	E_coli_ASM584v2	135	4872441	50.79	89048	pass	99.1362	pass	0
SAMN30416457	SRR21143726	PRJNA861272	*Escherichia coli*	Jay Graham Laboratory	University of California - Berkeley	2021	Quito, Ecuador	feces	Homo sapiens	Surveillance			100	0.64	pass	E_coli_ASM584v2	223	5304781	50.58	77280	pass	97.8842	pass	0
SAMN30414030	SRR21142176	PRJNA861272	*Escherichia coli*	Jay Graham Laboratory	University of California - Berkeley	2021	Quito, Ecuador	feces	Homo sapiens	Surveillance			100	0.14	pass	E_coli_ASM584v2	159	4974181	50.61	94927	pass	99.5452	pass	0
SAMN30415829	SRR21143639	PRJNA861272	*Escherichia coli*	Jay Graham Laboratory	University of California - Berkeley	2018	Quito, Ecuador	feces	Homo sapiens	Surveillance			100	0.26	pass	E_coli_ASM584v2	98	5102963	50.7	197185	pass	97.3091	pass	0
SAMN30414591	SRR21142328	PRJNA861272	*Escherichia coli*	Jay Graham Laboratory	University of California - Berkeley	2018	Quito, Ecuador	feces	Homo sapiens	Surveillance			100	0.32	pass	E_coli_ASM584v2	142	4950622	50.59	110044	pass	98.4005	pass	0
SAMN30416453	SRR21143730	PRJNA861272	*Escherichia coli*	Jay Graham Laboratory	University of California - Berkeley	2021	Quito, Ecuador	feces	Homo sapiens	Surveillance			100	0.26	pass	E_coli_ASM584v2	195	5185707	50.83	142387	pass	97.1288	pass	0
SAMN30413986	SRR21141826	PRJNA861272	*Escherichia coli*	Jay Graham Laboratory	University of California - Berkeley	2021	Quito, Ecuador	feces	Homo sapiens	Surveillance			100	0.03	pass	E_coli_ASM584v2	184	5100930	50.82	54525	pass	97.2157	pass	0
SAMN30414061	SRR21141422	PRJNA861272	*Escherichia coli*	Jay Graham Laboratory	University of California - Berkeley	2021	Quito, Ecuador	feces	Homo sapiens	Surveillance			98.86	0.43	pass	E_coli_ASM584v2	289	5097498	50.6	40431	pass	97.2765	pass	0
SAMN30414501	SRR21141509	PRJNA861272	*Escherichia coli*	Jay Graham Laboratory	University of California - Berkeley	2021	Quito, Ecuador	feces	Homo sapiens	Surveillance			100	0.13	pass	E_coli_ASM584v2	220	5067184	50.97	62852	pass	97.1252	pass	0
SAMN30414569	SRR21141914	PRJNA861272	*Escherichia coli*	Jay Graham Laboratory	University of California - Berkeley	2018	Quito, Ecuador	feces	Homo sapiens	Surveillance			100	0.09	pass	E_coli_ASM584v2	96	4689319	50.67	110099	pass	99.1316	pass	0
SAMN30414447	SRR21142361	PRJNA861272	*Escherichia coli*	Jay Graham Laboratory	University of California - Berkeley	2021	Quito, Ecuador	feces	Homo sapiens	Surveillance			99.99	0.08	pass	E_coli_ASM584v2	241	5248779	50.93	71904	pass	97.3399	pass	0

ID	SRA Run	Bioproject	Organism	Recollected	Submmiter	Collection date	Geo loc name	Isolation source	Host	Purpose of sampling	Lat ion	Hospital	Completeness_General	Contamination	1st Criterion	FastANI Reference	# contigs	Total length	GC (%)	N50	2nd Criterion	FastANI (%)	3rd Criterion	Failed criteria
SAMN30414466	SRR21142077	PRJNA514245	*Escherichia coli*	Jay Graham Laboratory	University of California - Berkeley	2021	Quito, Ecuador	feces	Homo sapiens	Surveillance			100	0.63	pass	E_coli_ASM584v2	202	5014957	50.76	60057	pass	96.6709	pass	0
SAMN30414115	SRR21141778	PRJNA861272	*Escherichia coli*	Jay Graham Laboratory	University of California - Berkeley	2021	Quito, Ecuador	feces	Homo sapiens	Surveillance			100	0.28	pass	E_coli_ASM584v2	240	5068317	50.85	51195	pass	97.3029	pass	0
SAMN30414629	SRR21141879	PRJNA861272	*Escherichia coli*	Jay Graham Laboratory	University of California - Berkeley	2018	Quito, Ecuador	feces	Homo sapiens	Surveillance			100	0.04	pass	E_coli_ASM584v2	110	4685311	50.75	133673	pass	99.5532	pass	0
SAMN30413877	SRR21142243	PRJNA861272	*Escherichia coli*	Jay Graham Laboratory	University of California - Berkeley	2021	Quito, Ecuador	feces	Homo sapiens	Surveillance			100	0.15	pass	E_coli_ASM584v2	95	4797966	50.68	123132	pass	98.3964	pass	0
SAMN30413958	SRR21141441	PRJNA861272	*Escherichia coli*	Jay Graham Laboratory	University of California - Berkeley	2021	Quito, Ecuador	feces	Homo sapiens	Surveillance			100	0.33	pass	E_coli_ASM584v2	182	5217453	50.47	197653	pass	97.2311	pass	0
SAMN30414523	SRR21141965	PRJNA861272	*Escherichia coli*	Jay Graham Laboratory	University of California - Berkeley	2021	Quito, Ecuador	feces	Homo sapiens	Surveillance			100	0.27	pass	E_coli_ASM584v2	167	5095094	50.76	121979	pass	97.265	pass	0
SAMN30414122	SRR21142170	PRJNA861272	*Escherichia coli*	Jay Graham Laboratory	University of California - Berkeley	2021	Quito, Ecuador	feces	Homo sapiens	Surveillance			100	0.05	pass	E_coli_ASM584v2	180	4828679	50.88	74244	pass	98.427	pass	0
SAMN30416116	SRR21143652	PRJNA861272	*Escherichia coli*	Jay Graham Laboratory	University of California - Berkeley	2021	Quito, Ecuador	feces	Homo sapiens	Surveillance			100	0.24	pass	E_coli_ASM584v2	195	5166925	50.74	88563	pass	97.2833	pass	0
SAMN30416092	SRR21143909	PRJNA861272	*Escherichia coli*	Jay Graham Laboratory	University of California - Berkeley	2021	Quito, Ecuador	feces	Homo sapiens	Surveillance			99.31	0	pass	E_coli_ASM584v2	265	4609146	50.65	41401	pass	99.4867	pass	0
SAMN30416390	SRR21143800	PRJNA861272	*Escherichia coli*	Jay Graham Laboratory	University of California - Berkeley	2021	Quito, Ecuador	feces	Homo sapiens	Surveillance			100	1.92	pass	E_coli_ASM584v2	114	5294989	50.47	130126	pass	97.8782	pass	0
SAMN30414438	SRR21142371	PRJNA861272	*Escherichia coli*	Jay Graham Laboratory	University of California - Berkeley	2021	Quito, Ecuador	feces	Homo sapiens	Surveillance			99.85	0	pass	E_coli_ASM584v2	225	4678727	50.73	42090	pass	99.6469	pass	0
SAMN30414441	SRR21142367	PRJNA861272	*Escherichia coli*	Jay Graham Laboratory	University of California - Berkeley	2021	Quito, Ecuador	feces	Homo sapiens	Surveillance			100	0.16	pass	E_coli_ASM584v2	223	4718950	50.7	51454	pass	98.9017	pass	0
SAMN30414717	SRR21141462	PRJNA861272	*Escherichia coli*	Jay Graham Laboratory	University of California - Berkeley	2018	Quito, Ecuador	feces	Homo sapiens	Surveillance			100	0.06	pass	E_coli_ASM584v2	200	4900523	50.8	82799	pass	98.4578	pass	0
SAMN30416091	SRR21143910	PRJNA861272	*Escherichia coli*	Jay Graham Laboratory	University of California - Berkeley	2021	Quito, Ecuador	feces	Homo sapiens	Surveillance			100	0.05	pass	E_coli_ASM584v2	239	4794429	50.96	41644	pass	98.4476	pass	0
SAMN30414435	SRR21142374	PRJNA861272	*Escherichia coli*	Jay Graham Laboratory	University of California - Berkeley	2021	Quito, Ecuador	feces	Homo sapiens	Surveillance			100	1.27	pass	E_coli_ASM584v2	188	5169807	50.91	96244	pass	96.7841	pass	0
SAMN30413790	SRR21141710	PRJNA861272	*Escherichia coli*	Jay Graham Laboratory	University of California - Berkeley	2021	Quito, Ecuador	feces	Homo sapiens	Surveillance			100	0.21	pass	E_coli_ASM584v2	83	4986776	50.63	248599	pass	98.3528	pass	0
SAMN30414137	SRR21142154	PRJNA861272	*Escherichia coli*	Jay Graham Laboratory	University of California - Berkeley	2021	Quito, Ecuador	feces	Homo sapiens	Surveillance			100	0.28	pass	E_coli_ASM584v2	223	5646166	50.51	76688	pass	97.2492	pass	0
SAMN30414542	SRR21141944	PRJNA861272	*Escherichia coli*	Jay Graham Laboratory	University of California - Berkeley	2021	Quito, Ecuador	feces	Homo sapiens	Surveillance			100	1.43	pass	E_coli_ASM584v2	200	4857742	50.53	80841	pass	99.1644	pass	0
SAMN30413904	SRR21141692	PRJNA861272	*Escherichia coli*	Jay Graham Laboratory	University of California - Berkeley	2021	Quito, Ecuador	feces	Homo sapiens	Surveillance			100	0.21	pass	E_coli_ASM584v2	341	5437064	50.54	41170	pass	97.3276	pass	0
SAMN30414275	SRR21141569	PRJNA861272	*Escherichia coli*	Jay Graham Laboratory	University of California - Berkeley	2021	Quito, Ecuador	feces	Homo sapiens	Surveillance			100	0.24	pass	E_coli_ASM584v2	338	5417017	50.63	39335	fail	97.3388	pass	1
SAMN30414534	SRR21141953	PRJNA861272	*Escherichia coli*	Jay Graham Laboratory	University of California - Berkeley	2021	Quito, Ecuador	feces	Homo sapiens	Surveillance			100	0.01	pass	E_coli_ASM584v2	165	4911996	50.76	82438	pass	98.3806	pass	0
SAMN30413985	SRR21141827	PRJNA861272	*Escherichia coli*	Jay Graham Laboratory	University of California - Berkeley	2021	Quito, Ecuador	feces	Homo sapiens	Surveillance			100	0.41	pass	E_coli_ASM584v2	315	5291974	50.69	43135	pass	97.2952	pass	0
SAMN30416468	SRR21143713	PRJNA861272	*Escherichia coli*	Jay Graham Laboratory	University of California - Berkeley	2021	Quito, Ecuador	feces	Homo sapiens	Surveillance			100	0.04	pass	E_coli_ASM584v2	118	4874688	50.72	101721	pass	99.1415	pass	0
SAMN30414711	SRR21141468	PRJNA861272	*Escherichia coli*	Jay Graham Laboratory	University of California - Berkeley	2018	Quito, Ecuador	feces	Homo sapiens	Surveillance			100	0.18	pass	E_coli_ASM584v2	186	4959237	50.53	88907	pass	99.1339	pass	0

ID	SRA Run	Bioproject	Organism	Recollected	Submmiter	Collection date	Geo loc name	Isolation source	Host	Purpose of sampling	Lat ion	Hospital	Completeness_General	Contamination	1st Criterion	FastANI Reference	# contigs	Total length	GC (%)	N50	2nd Criterion	FastANI (%)	3rd Criterion	Failed criteria
SAMN30414086	SRR21141395	PRJNA861272	*Escherichia coli*	Jay Graham Laboratory	University of California - Berkeley	2021	Quito, Ecuador	feces	Homo sapiens	Surveillance			100	0.18	pass	E_coli_ASM584v2	317	5177833	50.71	36100	fail	98.8219	pass	1
SAMN30414509	SRR21141500	PRJNA861272	*Escherichia coli*	Jay Graham Laboratory	University of California - Berkeley	2021	Quito, Ecuador	feces	Homo sapiens	Surveillance			100	0.76	pass	E_coli_ASM584v2	201	4951189	50.8	62494	pass	96.781	pass	0
SAMN30414146	SRR21142144	PRJNA861272	*Escherichia coli*	Jay Graham Laboratory	University of California - Berkeley	2021	Quito, Ecuador	feces	Homo sapiens	Surveillance			100	0.03	pass	E_coli_ASM584v2	255	5386659	50.53	79802	pass	97.1906	pass	0
SAMN30414057	SRR21141619	PRJNA861272	*Escherichia coli*	Jay Graham Laboratory	University of California - Berkeley	2021	Quito, Ecuador	feces	Homo sapiens	Surveillance			99.57	0.12	pass	E_coli_ASM584v2	190	5164595	50.61	88863	pass	98.3664	pass	0
SAMN30414451	SRR21142356	PRJNA861272	*Escherichia coli*	Jay Graham Laboratory	University of California - Berkeley	2021	Quito, Ecuador	feces	Homo sapiens	Surveillance			100	0.13	pass	E_coli_ASM584v2	332	5088569	50.92	35531	fail	97.1648	pass	1
SAMN30414722	SRR21141456	PRJNA861272	*Escherichia coli*	Jay Graham Laboratory	University of California - Berkeley	2018	Quito, Ecuador	feces	Homo sapiens	Surveillance			99.91	0.18	pass	E_coli_ASM584v2	180	4921102	50.61	86188	pass	98.4695	pass	0
SAMN30413991	SRR21141819	PRJNA861272	*Escherichia coli*	Jay Graham Laboratory	University of California - Berkeley	2021	Quito, Ecuador	feces	Homo sapiens	Surveillance			100	1.37	pass	E_coli_ASM584v2	200	5293192	50.73	56337	pass	96.8018	pass	0
SAMN30414450	SRR21142357	PRJNA861272	*Escherichia coli*	Jay Graham Laboratory	University of California - Berkeley	2021	Quito, Ecuador	feces	Homo sapiens	Surveillance			100	1.13	pass	E_coli_ASM584v2	183	5369128	50.58	103150	pass	97.817	pass	0
SAMN30413812	SRR21142189	PRJNA861272	*Escherichia coli*	Jay Graham Laboratory	University of California - Berkeley	2021	Quito, Ecuador	feces	Homo sapiens	Surveillance			100	0.03	pass	E_coli_ASM584v2	179	4755999	50.87	52012	pass	98.4838	pass	0
SAMN30414124	SRR21142168	PRJNA861272	*Escherichia coli*	Jay Graham Laboratory	University of California - Berkeley	2021	Quito, Ecuador	feces	Homo sapiens	Surveillance			100	0.24	pass	E_coli_ASM584v2	199	4827157	50.76	57617	pass	98.3985	pass	0
SAMN30414461	SRR21142345	PRJNA861272	*Escherichia coli*	Jay Graham Laboratory	University of California - Berkeley	2021	Quito, Ecuador	feces	Homo sapiens	Surveillance			100	0.04	pass	E_coli_ASM584v2	264	5151734	50.9	56322	pass	98.4726	pass	0
SAMN30414586	SRR21142335	PRJNA861272	*Escherichia coli*	Jay Graham Laboratory	University of California - Berkeley	2018	Quito, Ecuador	feces	Homo sapiens	Surveillance			100	0.1	pass	E_coli_ASM584v2	176	5358342	50.36	127551	pass	97.2797	pass	0
SAMN30413793	SRR21141377	PRJNA861272	*Escherichia coli*	Jay Graham Laboratory	University of California - Berkeley	2021	Quito, Ecuador	feces	Homo sapiens	Surveillance			100	0.08	pass	E_coli_ASM584v2	383	4972095	51.03	27903	fail	97.2621	pass	1
SAMN30414315	SRR21142004	PRJNA861272	*Escherichia coli*	Jay Graham Laboratory	University of California - Berkeley	2021	Quito, Ecuador	feces	Homo sapiens	Surveillance			99.96	0.08	pass	E_coli_ASM584v2	153	4853089	50.65	64796	pass	97.2667	pass	0
SAMN30414659	SRR21142285	PRJNA861272	*Escherichia coli*	Jay Graham Laboratory	University of California - Berkeley	2018	Quito, Ecuador	feces	Homo sapiens	Surveillance			100	0.13	pass	E_coli_ASM584v2	98	5188804	50.48	163304	pass	97.2654	pass	0
SAMN30413853	SRR21141974	PRJNA861272	*Escherichia coli*	Jay Graham Laboratory	University of California - Berkeley	2021	Quito, Ecuador	feces	Homo sapiens	Surveillance			100	0.1	pass	E_coli_ASM584v2	113	4818487	50.73	107477	pass	99.0597	pass	0
SAMN30414394	SRR21141532	PRJNA861272	*Escherichia coli*	Jay Graham Laboratory	University of California - Berkeley	2021	Quito, Ecuador	feces	Homo sapiens	Surveillance			100	0.88	pass	E_coli_ASM584v2	312	5274442	50.68	37126	fail	97.1034	pass	1
SAMN30414177	SRR21141390	PRJNA861272	*Escherichia coli*	Jay Graham Laboratory	University of California - Berkeley	2021	Quito, Ecuador	feces	Homo sapiens	Surveillance			99.94	0.03	pass	E_coli_ASM584v2	127	4758440	50.83	86213	pass	98.4561	pass	0
SAMN30416405	SRR21143784	PRJNA861272	*Escherichia coli*	Jay Graham Laboratory	University of California - Berkeley	2021	Quito, Ecuador	feces	Homo sapiens	Surveillance			100	0.24	pass	E_coli_ASM584v2	220	5505180	50.54	69096	pass	97.0543	pass	0
SAMN30414082	SRR21141399	PRJNA861272	*Escherichia coli*	Jay Graham Laboratory	University of California - Berkeley	2021	Quito, Ecuador	feces	Homo sapiens	Surveillance			100	1.03	pass	E_coli_ASM584v2	112	5129117	50.68	141121	pass	96.7418	pass	0
SAMN30416350	SRR21143483	PRJNA861272	*Escherichia coli*	Jay Graham Laboratory	University of California - Berkeley	2021	Quito, Ecuador	feces	Homo sapiens	Surveillance			100	0.23	pass	E_coli_ASM584v2	298	5140800	51.02	39554	fail	97.2771	pass	1
SAMN30414149	SRR21141612	PRJNA861272	*Escherichia coli*	Jay Graham Laboratory	University of California - Berkeley	2021	Quito, Ecuador	feces	Homo sapiens	Surveillance			100	0	pass	E_coli_ASM584v2	357	5041688	50.97	29391	fail	99.5016	pass	1
SAMN30413804	SRR21141654	PRJNA861272	*Escherichia coli*	Jay Graham Laboratory	University of California - Berkeley	2021	Quito, Ecuador	feces	Homo sapiens	Surveillance			100	0.18	pass	E_coli_ASM584v2	106	4537486	50.98	80479	pass	98.9756	pass	0
SAMN30413797	SRR21142332	PRJNA861272	*Escherichia coli*	Jay Graham Laboratory	University of California - Berkeley	2021	Quito, Ecuador	feces	Homo sapiens	Surveillance			100	0.09	pass	E_coli_ASM584v2	54	4751438	50.69	262298	pass	98.4787	pass	0

ID	SRA Run	Bioproject	Organism	Recollected	Submitter	Collection date	Geo loc name	Isolation source	Host	Purpose of sampling	Lat ion	Hospital	Completeness_General	Contamination	1st Criterion	FastANI Reference	# contigs	Total length	GC (%)	N50	2nd Criterion	FastANI (%)	3rd Criterion	Failed criteria
SAMN30414395	SRR21141531	PRJNA861272	*Escherichia coli*	Jay Graham Laboratory	University of California - Berkeley	2021	Quito, Ecuador	feces	Homo sapiens	Surveillance			100	0.14	pass	E_coli_ASM584v2	208	5098906	50.77	58923	pass	97.1221	pass	0
SAMN30414630	SRR21141878	PRJNA861272	*Escherichia coli*	Jay Graham Laboratory	University of California - Berkeley	2018	Quito, Ecuador	feces	Homo sapiens	Surveillance			98.85	0.03	pass	E_coli_ASM584v2	175	4879333	50.66	90997	pass	98.4562	pass	0
SAMN30414048	SRR21141629	PRJNA861272	*Escherichia coli*	Jay Graham Laboratory	University of California - Berkeley	2021	Quito, Ecuador	feces	Homo sapiens	Surveillance			100	1.39	pass	E_coli_ASM584v2	102	5245367	50.54	221587	pass	97.8672	pass	0
SAMN30413880	SRR21141482	PRJNA861272	*Escherichia coli*	Jay Graham Laboratory	University of California - Berkeley	2021	Quito, Ecuador	feces	Homo sapiens	Surveillance			100	0.16	pass	E_coli_ASM584v2	226	4824819	50.79	66300	pass	99.4675	pass	0
SAMN30413919	SRR21141675	PRJNA861272	*Escherichia coli*	Jay Graham Laboratory	University of California - Berkeley	2021	Quito, Ecuador	feces	Homo sapiens	Surveillance			100	0.39	pass	E_coli_ASM584v2	170	5197367	50.3	80955	pass	99.6619	pass	0
SAMN30414127	SRR21142165	PRJNA861272	*Escherichia coli*	Jay Graham Laboratory	University of California - Berkeley	2021	Quito, Ecuador	feces	Homo sapiens	Surveillance			100	0.19	pass	E_coli_ASM584v2	162	4907190	50.69	92958	pass	98.3662	pass	0
SAMN30413816	SRR21141617	PRJNA861272	*Escherichia coli*	Jay Graham Laboratory	University of California - Berkeley	2021	Quito, Ecuador	feces	Homo sapiens	Surveillance			100	0.06	pass	E_coli_ASM584v2	136	4848693	50.79	82642	pass	98.4306	pass	0
SAMN30414764	SRR21142265	PRJNA861272	*Escherichia coli*	Jay Graham Laboratory	University of California - Berkeley	2018	Quito, Ecuador	feces	Homo sapiens	Surveillance			100	0.83	pass	E_coli_ASM584v2	152	5327416	50.6	159728	pass	97.229	pass	0
SAMN30414107	SRR21141787	PRJNA861272	*Escherichia coli*	Jay Graham Laboratory	University of California - Berkeley	2021	Quito, Ecuador	feces	Homo sapiens	Surveillance			100	0.01	pass	E_coli_ASM584v2	135	4910516	54.2	80662	pass	81.1277	fail	1
SAMN30416344	SRR21143490	PRJNA861272	*Escherichia coli*	Jay Graham Laboratory	University of California - Berkeley	2021	Quito, Ecuador	feces	Homo sapiens	Surveillance			100	0.02	pass	E_coli_ASM584v2	259	5064841	50.61	46052	pass	98.4059	pass	0
SAMN30414293	SRR21142028	PRJNA861272	*Escherichia coli*	Jay Graham Laboratory	University of California - Berkeley	2021	Quito, Ecuador	feces	Homo sapiens	Surveillance			100	0.05	pass	E_coli_ASM584v2	219	4877536	50.67	45605	pass	97.1478	pass	0
SAMN30414324	SRR21141738	PRJNA861272	*Escherichia coli*	Jay Graham Laboratory	University of California - Berkeley	2021	Quito, Ecuador	feces	Homo sapiens	Surveillance			100	0.18	pass	E_coli_ASM584v2	311	4799237	50.93	29161	fail	99.6818	pass	2
SAMN30414050	SRR21141626	PRJNA861272	*Escherichia coli*	Jay Graham Laboratory	University of California - Berkeley	2021	Quito, Ecuador	feces	Homo sapiens	Surveillance			100	0.03	pass	E_coli_ASM584v2	107	5122322	50.38	129722	pass	98.4154	pass	0
SAMN30414245	SRR21142130	PRJNA514245	*Escherichia coli*	Jay Graham Laboratory	University of California - Berkeley	2021	Quito, Ecuador	feces	Homo sapiens	Surveillance			99.65	0.61	pass	E_coli_ASM584v2	252	5299570	50.66	42163	pass	97.0713	pass	0
SAMN30413859	SRR21142051	PRJNA861272	*Escherichia coli*	Jay Graham Laboratory	University of California - Berkeley	2021	Quito, Ecuador	feces	Homo sapiens	Surveillance			100	0.14	pass	E_coli_ASM584v2	194	5065447	50.83	65400	pass	97.1201	pass	0
SAMN30413801	SRR21141687	PRJNA861272	*Escherichia coli*	Jay Graham Laboratory	University of California - Berkeley	2021	Quito, Ecuador	feces	Homo sapiens	Surveillance			100	0.09	pass	E_coli_ASM584v2	346	5034887	50.5	48838	pass	99.5034	pass	0
SAMN30414229	SRR21141747	PRJNA861272	*Escherichia coli*	Jay Graham Laboratory	University of California - Berkeley	2021	Quito, Ecuador	feces	Homo sapiens	Surveillance			98.87	0.06	pass	E_coli_ASM584v2	356	4792893	50.87	30277	fail	98.4167	pass	1
SAMN30413929	SRR21141664	PRJNA861272	*Escherichia coli*	Jay Graham Laboratory	University of California - Berkeley	2021	Quito, Ecuador	feces	Homo sapiens	Surveillance			100	0.19	pass	E_coli_ASM584v2	138	4960478	50.5	104673	pass	98.314	pass	0
SAMN30414762	SRR21142267	PRJNA861272	*Escherichia coli*	Jay Graham Laboratory	University of California - Berkeley	2018	Quito, Ecuador	feces	Homo sapiens	Surveillance			100	0.85	pass	E_coli_ASM584v2	155	5337895	50.6	159728	pass	97.238	pass	0
SAMN30413916	SRR21141679	PRJNA861272	*Escherichia coli*	Jay Graham Laboratory	University of California - Berkeley	2021	Quito, Ecuador	feces	Homo sapiens	Surveillance			100	0.01	pass	E_coli_ASM584v2	96	4796776	50.68	138874	pass	99.4827	pass	0
SAMN30414043	SRR21141634	PRJNA861272	*Escherichia coli*	Jay Graham Laboratory	University of California - Berkeley	2021	Quito, Ecuador	feces	Homo sapiens	Surveillance			99.04	0.16	pass	E_coli_ASM584v2	122	5239388	50.58	153922	pass	98.3838	pass	0
SAMN30414151	SRR21141610	PRJNA861272	*Escherichia coli*	Jay Graham Laboratory	University of California - Berkeley	2021	Quito, Ecuador	feces	Homo sapiens	Surveillance			100	0.22	pass	E_coli_ASM584v2	323	4943508	50.82	36050	fail	98.4832	pass	1
SAMN30416114	SRR21143654	PRJNA861272	*Escherichia coli*	Jay Graham Laboratory	University of California - Berkeley	2021	Quito, Ecuador	feces	Homo sapiens	Surveillance			100	0.2	pass	E_coli_ASM584v2	175	5077368	50.88	68489	pass	97.2437	pass	0
SAMN30414131	SRR21142160	PRJNA861272	*Escherichia coli*	Jay Graham Laboratory	University of California - Berkeley	2021	Quito, Ecuador	feces	Homo sapiens	Surveillance			100	0.19	pass	E_coli_ASM584v2	192	4758239	50.7	72118	pass	99.214	pass	0

ID	SRA Run	Bioproject	Organism	Recollected	Submmiter	Collection date	Geo loc name	Isolation source	Host	Purpose of sampling	Lat ion	Hospital	Completeness_General	Contamination	1st Criterion	FastANI Reference	# contigs	Total length	GC (%)	N50	2nd Criterion	FastANI (%)	3rd Criterion	Failed criteria
SAMN30413906	SRR21141690	PRJNA861272	*Escherichia coli*	Jay Graham Laboratory	University of California - Berkeley	2021	Quito, Ecuador	feces	Homo sapiens	Surveillance			100	0.25	pass	E_coli_ASM584v2	149	5009775	50.66	75876	pass	98.3415	pass	0
SAMN30413802	SRR21141676	PRJNA861272	*Escherichia coli*	Jay Graham Laboratory	University of California - Berkeley	2021	Quito, Ecuador	feces	Homo sapiens	Surveillance			100	0.15	pass	E_coli_ASM584v2	97	4594814	50.8	131563	pass	99.2922	pass	0
SAMN30414081	SRR21141400	PRJNA861272	*Escherichia coli*	Jay Graham Laboratory	University of California - Berkeley	2021	Quito, Ecuador	feces	Homo sapiens	Surveillance			99.89	0	pass	E_coli_ASM584v2	219	4702207	50.71	59789	pass	99.5214	pass	0
SAMN30413980	SRR21141832	PRJNA861272	*Escherichia coli*	Jay Graham Laboratory	University of California - Berkeley	2021	Quito, Ecuador	feces	Homo sapiens	Surveillance			100	0.01	pass	E_coli_ASM584v2	231	5025602	50.82	62212	pass	98.5181	pass	0
SAMN30414263	SRR21141582	PRJNA861272	*Escherichia coli*	Jay Graham Laboratory	University of California - Berkeley	2021	Quito, Ecuador	feces	Homo sapiens	Surveillance			100	0.23	pass	E_coli_ASM584v2	260	4997266	50.89	38188	fail	97.2975	pass	1
SAMN30414643	SRR21142303	PRJNA861272	*Escherichia coli*	Jay Graham Laboratory	University of California - Berkeley	2018	Quito, Ecuador	feces	Homo sapiens	Surveillance			100	0.09	pass	E_coli_ASM584v2	93	5136032	50.59	165883	pass	97.1703	pass	0
SAMN30416448	SRR21143735	PRJNA861272	*Escherichia coli*	Jay Graham Laboratory	University of California - Berkeley	2021	Quito, Ecuador	feces	Homo sapiens	Surveillance			100	0.08	pass	E_coli_ASM584v2	158	5047297	50.71	94846	pass	98.4612	pass	0
SAMN30415836	SRR21143636	PRJNA861272	*Escherichia coli*	Jay Graham Laboratory	University of California - Berkeley	2018	Quito, Ecuador	feces	Homo sapiens	Surveillance			100	0.15	pass	E_coli_ASM584v2	114	4846500	50.62	106380	pass	98.9362	pass	0
SAMN30414378	SRR21141551	PRJNA861272	*Escherichia coli*	Jay Graham Laboratory	University of California - Berkeley	2021	Quito, Ecuador	feces	Homo sapiens	Surveillance			98.56	0.06	pass	E_coli_ASM584v2	304	4678847	50.84	36566	fail	98.4599	pass	1
SAMN30413835	SRR21142126	PRJNA861272	*Escherichia coli*	Jay Graham Laboratory	University of California - Berkeley	2021	Quito, Ecuador	feces	Homo sapiens	Surveillance			100	0.74	pass	E_coli_ASM584v2	107	5239062	50.62	149913	pass	97.7999	pass	0
SAMN30414063	SRR21141420	PRJNA861272	*Escherichia coli*	Jay Graham Laboratory	University of California - Berkeley	2021	Quito, Ecuador	feces	Homo sapiens	Surveillance			98.85	0.02	pass	E_coli_ASM584v2	393	5142880	50.62	34563	fail	97.2789	pass	1
SAMN30414440	SRR21142368	PRJNA861272	*Escherichia coli*	Jay Graham Laboratory	University of California - Berkeley	2021	Quito, Ecuador	feces	Homo sapiens	Surveillance			100	1.14	pass	E_coli_ASM584v2	318	5423777	50.61	36345	fail	97.2388	pass	1
SAMN30416339	SRR21143495	PRJNA861272	*Escherichia coli*	Jay Graham Laboratory	University of California - Berkeley	2021	Quito, Ecuador	feces	Homo sapiens	Surveillance			100	0.2	pass	E_coli_ASM584v2	68	5011227	50.74	239267	pass	98.395	pass	0
SAMN30414423	SRR21141980	PRJNA861272	*Escherichia coli*	Jay Graham Laboratory	University of California - Berkeley	2021	Quito, Ecuador	feces	Homo sapiens	Surveillance			100	0.16	pass	E_coli_ASM584v2	353	4814339	50.9	26710	fail	98.3814	pass	1
SAMN30414227	SRR21141750	PRJNA861272	*Escherichia coli*	Jay Graham Laboratory	University of California - Berkeley	2021	Quito, Ecuador	feces	Homo sapiens	Surveillance			100	0.41	pass	E_coli_ASM584v2	328	4828990	50.97	33004	fail	98.464	pass	1
SAMN30414231	SRR21141745	PRJNA861272	*Escherichia coli*	Jay Graham Laboratory	University of California - Berkeley	2021	Quito, Ecuador	feces	Homo sapiens	Surveillance			99.62	0	pass	E_coli_ASM584v2	259	4620868	51.08	35393	fail	99.5319	pass	1
SAMN30414150	SRR21141611	PRJNA861272	*Escherichia coli*	Jay Graham Laboratory	University of California - Berkeley	2021	Quito, Ecuador	feces	Homo sapiens	Surveillance			100	0.03	pass	E_coli_ASM584v2	423	5306927	50.8	25067	fail	97.2709	pass	1
SAMN30416442	SRR21143742	PRJNA861272	*Escherichia coli*	Jay Graham Laboratory	University of California - Berkeley	2021	Quito, Ecuador	feces	Homo sapiens	Surveillance			100	0.03	pass	E_coli_ASM584v2	148	5116251	50.67	110040	pass	98.4092	pass	0
SAMN30414232	SRR21141744	PRJNA861272	*Escherichia coli*	Jay Graham Laboratory	University of California - Berkeley	2021	Quito, Ecuador	feces	Homo sapiens	Surveillance			99.87	0.02	pass	E_coli_ASM584v2	127	5021799	50.58	94007	pass	98.3964	pass	0
SAMN30414433	SRR21141969	PRJNA861272	*Escherichia coli*	Jay Graham Laboratory	University of California - Berkeley	2021	Quito, Ecuador	feces	Homo sapiens	Surveillance			97.84	0.05	pass	E_coli_ASM584v2	209	4837955	50.91	42796	pass	97.2031	pass	0
SAMN30413891	SRR21141706	PRJNA861272	*Escherichia coli*	Jay Graham Laboratory	University of California - Berkeley	2021	Quito, Ecuador	feces	Homo sapiens	Surveillance			100	0.12	pass	E_coli_ASM584v2	65	4580206	50.63	187705	pass	99.1834	pass	0
SAMN30414349	SRR21142110	PRJNA861272	*Escherichia coli*	Jay Graham Laboratory	University of California - Berkeley	2021	Quito, Ecuador	feces	Homo sapiens	Surveillance			100	0.17	pass	E_coli_ASM584v2	301	5012622	50.94	32369	fail	98.4243	pass	1
SAMN30414766	SRR21142263	PRJNA861272	*Escherichia coli*	Jay Graham Laboratory	University of California - Berkeley	2018	Quito, Ecuador	feces	Homo sapiens	Surveillance			100	0.69	pass	E_coli_ASM584v2	156	5329888	50.6	159959	pass	97.2663	pass	0
SAMN30414219	SRR21141758	PRJNA861272	*Escherichia coli*	Jay Graham Laboratory	University of California - Berkeley	2018	Quito, Ecuador	feces	Homo sapiens	Surveillance			100	0.23	pass	E_coli_ASM584v2	154	4873818	50.8	91893	pass	99.5139	pass	0

ID	SRA Run	Bioproject	Organism	Recollected	Submmiter	Collection date	Geo loc name	Isolation source	Host	Purpose of sampling	Lat ion	Hospital	Completeness_General	Contamination	1st Criterion	FastANI Reference	# contigs	Total length	GC (%)	N50	2nd Criterion	FastANI (%)	3rd Criterion	Failed criteria
SAMN30413876	SRR21142286	PRJNA861272	*Escherichia coli*	Jay Graham Laboratory	University of California - Berkeley	2018	Quito, Ecuador	feces	Homo sapiens	Surveillance			99.98	0.03	pass	E_coli_ASM584v2	128	4801137	50.56	79312	pass	99.6905	pass	0
SAMN30416110	SRR21143658	PRJNA861272	*Escherichia coli*	Jay Graham Laboratory	University of California - Berkeley	2021	Quito, Ecuador	feces	Homo sapiens	Surveillance			100	0.14	pass	E_coli_ASM584v2	192	4533637	50.84	55544	pass	99.194	pass	0
SAMN30416120	SRR21143647	PRJNA861272	*Escherichia coli*	Jay Graham Laboratory	University of California - Berkeley	2021	Quito, Ecuador	feces	Homo sapiens	Surveillance			100	0.05	pass	E_coli_ASM584v2	282	5395628	50.73	54486	pass	97.2794	pass	0
SAMN30414545	SRR21141941	PRJNA861272	*Escherichia coli*	Jay Graham Laboratory	University of California - Berkeley	2021	Quito, Ecuador	feces	Homo sapiens	Surveillance			100	0.08	pass	E_coli_ASM584v2	187	4918608	50.74	77769	pass	98.4003	pass	0
SAMN30414776	SRR21142252	PRJNA861272	*Escherichia coli*	Jay Graham Laboratory	University of California - Berkeley	2018	Quito, Ecuador	feces	Homo sapiens	Surveillance			100	0.07	pass	E_coli_ASM584v2	183	5419359	50.44	174276	pass	97.2283	pass	0
SAMN30414214	SRR21141764	PRJNA861272	*Escherichia coli*	Jay Graham Laboratory	University of California - Berkeley	2021	Quito, Ecuador	feces	Homo sapiens	Surveillance			100	0.35	pass	E_coli_ASM584v2	212	5194818	50.61	58511	pass	97.1905	pass	0
SAMN30414281	SRR21141562	PRJNA861272	*Escherichia coli*	Jay Graham Laboratory	University of California - Berkeley	2021	Quito, Ecuador	feces	Homo sapiens	Surveillance			100	1.45	pass	E_coli_ASM584v2	161	5092713	50.87	87380	pass	96.7612	pass	0
SAMN30414248	SRR21142127	PRJNA514245	*Escherichia coli*	Jay Graham Laboratory	University of California - Berkeley	2021	Quito, Ecuador	feces	Homo sapiens	Surveillance			100	0.19	pass	E_coli_ASM584v2	231	4959042	50.72	52305	pass	99.119	pass	0
SAMN30414156	SRR21141605	PRJNA861272	*Escherichia coli*	Jay Graham Laboratory	University of California - Berkeley	2021	Quito, Ecuador	feces	Homo sapiens	Surveillance			100	0.13	pass	E_coli_ASM584v2	209	5116055	50.67	65397	pass	98.3631	pass	0
SAMN30414581	SRR21142340	PRJNA861272	*Escherichia coli*	Jay Graham Laboratory	University of California - Berkeley	2018	Quito, Ecuador	feces	Homo sapiens	Surveillance			100	0.05	pass	E_coli_ASM584v2	178	4993355	50.58	62754	pass	97.2641	pass	0
SAMN30413911	SRR21141684	PRJNA861272	*Escherichia coli*	Jay Graham Laboratory	University of California - Berkeley	2021	Quito, Ecuador	feces	Homo sapiens	Surveillance			97.11	0.01	pass	E_coli_ASM584v2	170	5002654	50.65	89731	pass	98.3429	pass	0
SAMN30416447	SRR21143737	PRJNA861272	*Escherichia coli*	Jay Graham Laboratory	University of California - Berkeley	2021	Quito, Ecuador	feces	Homo sapiens	Surveillance			100	0.14	pass	E_coli_ASM584v2	144	4804705	50.79	119319	pass	99.3632	pass	0
SAMN30414381	SRR21141547	PRJNA861272	*Escherichia coli*	Jay Graham Laboratory	University of California - Berkeley	2021	Quito, Ecuador	feces	Homo sapiens	Surveillance			100	0.14	pass	E_coli_ASM584v2	193	4952866	50.77	54146	pass	98.3714	pass	0
SAMN30416115	SRR21143653	PRJNA861272	*Escherichia coli*	Jay Graham Laboratory	University of California - Berkeley	2021	Quito, Ecuador	feces	Homo sapiens	Surveillance			100	0.14	pass	E_coli_ASM584v2	243	4842666	50.88	53869	pass	99.451	pass	0
SAMN30414567	SRR21141917	PRJNA861272	*Escherichia coli*	Jay Graham Laboratory	University of California - Berkeley	2018	Quito, Ecuador	feces	Homo sapiens	Surveillance			98.74	0.05	pass	E_coli_ASM584v2	137	4749620	50.66	87579	pass	99.2916	pass	0
SAMN30414125	SRR21142167	PRJNA861272	*Escherichia coli*	Jay Graham Laboratory	University of California - Berkeley	2021	Quito, Ecuador	feces	Homo sapiens	Surveillance			100	0.37	pass	E_coli_ASM584v2	143	5343949	50.66	99368	pass	97.2627	pass	0
SAMN30414055	SRR21141621	PRJNA861272	*Escherichia coli*	Jay Graham Laboratory	University of California - Berkeley	2021	Quito, Ecuador	feces	Homo sapiens	Surveillance			98.94	0.28	pass	K_pneumoniae_ASM24018v2	111	5285991	57.49	138272	pass	99.0872	pass	0
SAMN30414377	SRR21142080	PRJNA514245	*Escherichia coli*	Jay Graham Laboratory	University of California - Berkeley	2021	Quito, Ecuador	feces	Homo sapiens	Surveillance			100	0	pass	E_coli_ASM584v2	298	4809987	50.94	35969	fail	99.4709	pass	1
SAMN30414304	SRR21142016	PRJNA861272	*Escherichia coli*	Jay Graham Laboratory	University of California - Berkeley	2021	Quito, Ecuador	feces	Homo sapiens	Surveillance			100	0.17	pass	E_coli_ASM584v2	191	4761522	50.78	65844	pass	98.9473	pass	0
SAMN09832919	SRR7693802	PRJNA486009	*Escherichia coli*	Instituto de Microbiologia, Universidad San Francisco de Quito	Georgia Institute of Technology	2015-05-27	Borbon, Ecuador	Stool	Homo sapiens	Surveillance	1.08 N 78.99 W		100	0.12	pass	E_coli_ASM584v2	72	4621915	50.81	193657	pass	98.4355	pass	0
SAMN09833031	SRR7693818	PRJNA486009	*Escherichia coli*	Instituto de Microbiologia, Universidad San Francisco de Quito	Georgia Institute of Technology	2014-08-13	Esmeraldas, Ecuador	Stool	Homo sapiens	Surveillance	0.96 N 79.65 W		100	0.18	pass	E_coli_ASM584v2	124	5045726	50.7	154227	pass	98.5607	pass	0
SAMN31969690	SRR22480110	PRJNA849201	*Escherichia coli*	Instituto Nacional en Investigacion en Salud Publica	Fernando Villavicencio	Not specified	Ecuador	unspecified diagnostic sample	Homo sapiens	Unspecified			99.49	0.03	pass	E_coli_ASM584v2	144	4635783	50.73	54007	pass	99.9768	pass	0

ID	SRA Run	Bioproject	Organism	Recollected	Submmiter	Collection date	Geo loc name	Isolation source	Host	Purpose of sampling	Lat ion	Hospital	Completeness_General	Contamination	1st Criterion	FastANI Reference	# contigs	Total length	GC (%)	N50	2nd Criterion	FastANI (%)	3rd Criterion	Failed criteria
SAMN52395837	#N/A	PRJNA1338115	*Klebsiella michiganensis*	Hospital de niños Dr Roberto Gilbert	UEES	2021	Guayas, Guayaquil, Ecuador	blood	Homo sapiens	Diseased	2.176570 S 79.883580 W	Hospital de niños Dr Roberto Gilbert	96.95	0.7	pass	K_michiganensis_ASM1513957v1	672	6047888	56.08	16487	fail	98.9136	pass	1
SAMN52395838	#N/A	PRJNA1338115	*Klebsiella michiganensis*	Hospital de niños Dr Roberto Gilbert	UEES	2021	Guayas, Guayaquil, Ecuador	blood	Homo sapiens	Diseased	2.176570 S 79.883580 W	Hospital de niños Dr Roberto Gilbert	98.91	0.52	pass	K_michiganensis_ASM1513957v1	260	6196834	55.86	97023	pass	98.9265	pass	0

Supplementary Table 2. Genomic context of Tn4401 variants, replicons and TSDs

Biosample	Bacteria	SRA Run	Tn4401 variant	*blaKPC variant*	Deletion	SNPs_homozygous	Left_flanks	Right_flanks	pKpQIL-IT - coverage (%)	TUM12128 ColRNAI - coverage (%)	pECU0062021_KPC - coverage (%)	pBK30683 - coverage (%)	(pKP91)-pKPC-3-Kp6 - coverage (%)	Plasmid
SAMN34233589	*Klebsiella pneumoniae*	SRR25759564	Tn4401a	*blaKPC-2*	7020-7118	none	ATTGA	ATTGA	96	71	20	51	74	IncFIB(pQil)
SAMEA111504313	*Klebsiella pneumoniae*	ERR10437152	Tn4401a	*blaKPC-2*	7020-7118	none	ATTGA	ATTGA	86	93	17	43	69	ColRNAI
SAMEA111504315	*Klebsiella pneumoniae*	ERR10437160	Tn4401a	*blaKPC-3*	7020-7118	C8015T	TGGCT	TGGCT	31	100	60	23	20	ColRNAI
SAMEA111504316	*Klebsiella pneumoniae*	ERR10437161	Tn4401a	*blaKPC-3*	7020-7118	C8015T	TGGCT	TGGCT	30	100	40	23	20	ColRNAI
SAMEA111504317	*Klebsiella pneumoniae*	ERR10437162	Tn4401a	*blaKPC-2*	7020-7118	none	ATCCA|ATTGA	ATCCA	73	100	26	40	48	ColRNAI
SAMEA111504314	*Klebsiella pneumoniae*	ERR10437163	Tn4401a	*blaKPC-2*	7020-7118	none	ATTGA	ATTGA	100	100	17	61	79	ColRNAI or IncFIB(pQil)
SAMEA111504319	*Klebsiella pneumoniae*	ERR10437168	Tn4401a	*blaKPC-3*	7020-7118	C8015T	TGGCT	TGGCT	37	100	40	24	20	ColRNAI
SAMEA111504318	*Klebsiella pneumoniae*	ERR10437175	Tn4401a	*blaKPC-2*	7020-7118	none	ATCCA|ATTGA	ATCCA|CTTTT	88	100	18	40	48	ColRNAI
SAMEA111504320	*Klebsiella pneumoniae*	ERR10437181	Tn4401a	*blaKPC-2*	7020-7118	none	ATTGA	ATTGA	82	96	17	44	67	ColRNAI
SAMEA111504323	*Klebsiella pneumoniae*	ERR10437192	Tn4401a	*blaKPC-3*	7020-7118	C8015T	TGGCT	TGGCT	30	100	42	24	20	ColRNAI
SAMEA111504321	*Klebsiella pneumoniae*	ERR10437193	Tn4401a	*blaKPC-3*	7020-7118	C8015T	TGGCT	TGGCT	36	100	48	26	25	ColRNAI
SAMEA111504322	*Klebsiella pneumoniae*	ERR10437194	Tn4401a	*blaKPC-3*	7020-7118	C8015T	TGGCT	TGGCT	33	100	41	25	22	ColRNAI
SAMEA111504325	*Klebsiella pneumoniae*	ERR10437196	Tn4401a	*blaKPC-2*	7020-7118	none	ATTGA	ATTGA	80	69	17	41	63	undetermined
SAMEA111504324	*Klebsiella pneumoniae*	ERR10437201	Tn4401a	*blaKPC-3*	7020-7118	C8015T	TGGCT	TGGCT	30	100	49	24	20	ColRNAI
SAMEA111504328	*Klebsiella pneumoniae*	ERR10437211	Tn4401a	*blaKPC-2*	7020-7118	none	ATTGA	ATTGA	98	70	17	51	73	IncFIB(pQil)
SAMEA111504326	*Klebsiella pneumoniae*	ERR10437213	Tn4401a	*blaKPC-2*	7020-7118	none	ATTGA	ATTGA	80	74	20	41	64	undetermined
SAMEA111504329	*Klebsiella pneumoniae*	ERR10437223	Tn4401l	*blaKPC-3*	7008-7075	C8015T	GTTCT	none	83	74	17	100	82	FII(pBK30683)
SAMEA111504331	*Klebsiella pneumoniae*	ERR10437231	Tn4401a	*blaKPC-3*	7020-7118	C8015T	ATTAT|ATTGA|TATCT	ATTAT|ATTGA	94	100	17	48	64	ColRNAI or IncFIB(pQil)
SAMEA111504330	*Klebsiella pneumoniae*	ERR10437233	Tn4401a	*blaKPC-2*	7020-7118	none	ATTGA	ATTGA	98	71	17	55	74	IncFIB(pQil)
SAMEA111504332	*Klebsiella pneumoniae*	ERR10437234	Tn4401a	*blaKPC-2*	7020-7118	none	ATTGA	ATTGA	98	76	18	52	74	IncFIB(pQil)
SAMEA111504327	*Klebsiella pneumoniae*	ERR10437235	Tn4401a	*blaKPC-2*	7020-7118	none	ATTGA	ATTGA	88	93	23	44	69	ColRNAI
SAMEA111504334	*Klebsiella pneumoniae*	ERR10437243	Tn4401a	*blaKPC-2*	7020-7118	none	ATTGA	ATTGA	77	100	17	45	64	ColRNAI
SAMEA111504333	*Klebsiella pneumoniae*	ERR10437245	Tn4401a	*blaKPC-2*	7020-7118|9967-10006	none	ATTGA	none	86	100	17	49	60	ColRNAI
SAMEA111504335	*Klebsiella pneumoniae*	ERR10437246	Tn4401a	*blaKPC-2*	7020-7118	none	ATTGA	ATTGA	84	100	17	50	65	ColRNAI
SAMEA111504339	*Klebsiella pneumoniae*	ERR10437254	Tn4401a	*blaKPC-3*	7020-7118	C8015T	TGGCT	TGGCT	29	100	39	23	20	ColRNAI
SAMEA111504337	*Klebsiella pneumoniae*	ERR10437258	Tn4401a	*blaKPC-3*	7020-7118	C8015T	TGGCT	TGGCT	25	99	41	18	19	ColRNAI
SAMEA111504336	*Klebsiella pneumoniae*	ERR10437259	Tn4401a	*blaKPC-2*	7020-7118	none	ATTGA	ATTGA	89	100	17	44	66	ColRNAI
SAMEA111504340	*Klebsiella pneumoniae*	ERR10437266	Tn4401a	*blaKPC-2*	7020-7118	none	ATTGA	ATTGA	89	93	35	43	68	ColRNAI

Biosample	Bacteria	SRA Run	Tn4401 variant	*blaKPC variant*	Deletion	SNPs_homozygous	Left_flanks	Right_flanks	pKpQIL-IT - coverage (%)	TUM12128 ColRNAI - coverage (%)	pECU0062021_KPC - coverage (%)	pBK30683 - coverage (%)	(pKP91)-pKPC-3-Kp6 - coverage (%)	Plasmid
SAMEA111504342	*Klebsiella pneumoniae*	ERR10437267	Tn4401a	*blaKPC-3*	7020-7118	C8015T	TGGCT	TGGCT	29	100	41	23	20	ColRNAI
SAMEA111504341	*Klebsiella pneumoniae*	ERR10437268	Tn4401a	*blaKPC-3*	7020-7118	C8015T	TGGCT	TGGCT	30	100	44	23	19	ColRNAI
SAMEA111504343	*Klebsiella pneumoniae*	ERR10437269	Tn4401a	*blaKPC-3*	7020-7118	C8015T	TGGCT	TGGCT	32	100	43	23	20	ColRNAI
SAMEA111504338	*Klebsiella pneumoniae*	ERR10437276	Tn4401a	*blaKPC-3*	7020-7118	C8015T	GGGCT|TGGCG|TGGCT	TGGCT	44	100	54	22	37	ColRNAI
SAMEA111504347	*Klebsiella pneumoniae*	ERR10437299	Tn4401a	*blaKPC-2*	7020-7118	none	ATTGA	ATTGA	99	71	19	56	79	IncFIB(pQil)
SAMEA111504346	*Klebsiella pneumoniae*	ERR10437301	Tn4401a	*blaKPC-2*	7020-7118	none	ATCCA|ATTGA	ATCCA|CTTTT	85	100	17	44	46	ColRNAI
SAMEA111504345	*Klebsiella pneumoniae*	ERR10437302	Tn4401a	*blaKPC-2*	7020-7118	none	ATCCA|ATTGA	ATCCA|CTTTT	86	100	32	40	48	ColRNAI
SAMEA111504349	*Klebsiella pneumoniae*	ERR10437312	Tn4401a	*blaKPC-3*	7020-7118	C8015T	TGGCT	TGGCT	30	100	39	24	20	ColRNAI
SAMEA111504348	*Klebsiella pneumoniae*	ERR10437314	Tn4401l	*blaKPC-3*	7008-7075	C8015T	GTTCT	TCTCT	75	86	17	100	64	FII(pBK30683)
SAMEA111504350	*Klebsiella pneumoniae*	ERR10437319	Tn4401a	*blaKPC-3*	7020-7118	C8015T	TGGCT	TGGCT	31	100	44	19	19	ColRNAI
SAMEA111504351	*Klebsiella pneumoniae*	ERR10437323	Tn4401a	*blaKPC-3*	7020-7118	C8015T	TGGCT	TGGCT	32	100	45	25	21	ColRNAI
SAMEA111504344	*Klebsiella pneumoniae*	ERR10437324	Tn4401a	*blaKPC-3*	7020-7118	C8015T	TGGCT	TGGCT	39	100	60	25	23	ColRNAI
SAMEA111504354	*Klebsiella pneumoniae*	ERR10437331	Tn4401a	*blaKPC-2*	7020-7118	none	AACAG	AACAG	79	75	100	43	51	pECU0062021_KPC
SAMEA111504355	*Klebsiella pneumoniae*	ERR10437335	Tn4401a	*blaKPC-2*	7020-7118	none	AACAG	AACAG	83	71	100	43	52	pECU0062021_KPC
SAMEA111504353	*Klebsiella pneumoniae*	ERR10437337	Tn4401a	*blaKPC-2*	7020-7118	none	AACAG	AACAG	79	73	100	46	52	pECU0062021_KPC
SAMEA111504352	*Klebsiella pneumoniae*	ERR10437338	Tn4401a	*blaKPC-3*	7020-7118	C8015T	TGGCT	TGGCT	31	100	41	24	20	ColRNAI
SAMEA111504356	*Klebsiella pneumoniae*	ERR10437351	Tn4401a	*blaKPC-2*	7020-7118	none	AACAG	AACAG	83	89	100	46	52	pECU0062021_KPC
SAMEA111504358	*Klebsiella pneumoniae*	ERR10437352	Tn4401a	*blaKPC-2*	7020-7118	none	ATTGA	ATTGA	86	93	17	44	69	undetermined
SAMEA111504357	*Klebsiella pneumoniae*	ERR10437353	Tn4401a	*blaKPC-2*	7020-7118	none	AACAG	AACAG	71	73	100	43	60	pECU0062021_KPC
SAMEA111504359	*Klebsiella pneumoniae*	ERR10437357	Tn4401a	*blaKPC-2*	7020-7118	none	ATTGA	ATTGA	82	94	29	43	69	ColRNAI
SAMEA111504296	*uncultured bacterium*	ERS13616658	Tn4401a	*blaKPC-3*	7020-7118	C8015T	TGGCT	TGGCT	34	100	45	24	20	ColRNAI
SAMEA111504307	*Klebsiella aerogenes*	ERS13616669	Tn4401a	*blaKPC-2*	7020-7118	none	AACAG	AACAG	74	100	100	50	69	ColRNAI or pECU0062021_KPC
SAMEA111504308	*Klebsiella aerogenes*	ERS13616670	Tn4401a	*blaKPC-2*	7020-7118	none	AACAG	AACAG	84	100	100	54	65	ColRNAI or pECU0062021_KPC
SAMEA111504309	*Klebsiella aerogenes*	ERS13616671	Tn4401a	*blaKPC-2*	7020-7118	none	AACAG	AACAG	69	87	100	50	63	pECU0062021_KPC
SAMEA111504310	*Klebsiella aerogenes*	ERS13616672	Tn4401a	*blaKPC-2*	7020-7118	none	AACAG	AACAG	13	100	100	12	13	pECU0072020_KPC
SAMEA111504312	*Klebsiella oxytoca*	ERR10437141	Tn4401a	*blaKPC-2*	7020-7118	none	AACAG	AACAG	12	49	100	8	12	pECU0062021_KPC
SAMEA111504360	*Serratia marcescens*	ERS13616722	Tn4401a	*blaKPC-2*	7020-7118	none	AACAG	AACAG	80	72	100	48	60	pECU0062021_KPC
SAMEA111504361	*Serratia marcescens*	ERS13616723	Tn4401a	*blaKPC-2*	7020-7118	none	AACAG	AACAG	72	69	100	48	58	pECU0062021_KPC
SAMEA111504362	*Klebsiella variicola*	ERS13616724	Tn4401a	*blaKPC-2*	7020-7118	none	AACAG	AACAG	14	52	100	21	14	pECU0062021_KPC

Biosample	Bacteria	SRA Run	Tn4401 variant	*blaKPC variant*	Deletion	SNPs_homozygous	Left_flanks	Right_flanks	pKpQIL-IT - coverage (%)	TUM12128 ColRNAI - coverage (%)	pECU0062021_KPC - coverage (%)	pBK30683 - coverage (%)	(pKP91)-pKPC-3-Kp6 - coverage (%)	Plasmid
SAMEA111504363	*Klebsiella variicola*	ERS13616725	Tn4401a	*blaKPC-2*	7020-7118	none	AACAG	AACAG	14	51	100	17	14	pECU0062021_KPC
SAMN29043418	*Salmonella enterica*	SRR19650860	Tn4401a	*blaKPC-2*	7020-7118	none	AACAG	AACAG	20	48	100	10	19	pECU0062021_KPC
SAMN31969690	*Escherichia coli*	SRR22480110	Tn4401a	*blaKPC-2*	7020-7118	none	AACAG	AACAG	11	65	100	8	11	pECU0062021_KPC

Supplementary Table 3. Characteristics of pBK30683-like plasmid

ID	AMR genes	Contigs	Total lenght	GC (%)	Tn4401
pBK30683 ("KF954760.1")	blaKPC-3, aac(6')-Ib', aadA1, blaOXA-9, blaTEM-150, aph(6)-Id, aph(3'')-Ib, sul2, dfrA14	1	139941	54.04	Tn*4401I*
SAMEA111504329	blaKPC-3, aph(6)-Id, aph(3'')-Ib, sul2 aadA1, blaOXA-9, dfrA14, blaTEM-150 aac(6')-Ib-W104R	134	186326	53.89	Tn*4401I*
SAMEA111504348	aac(6')-Ib-AKT, aadA1, blaOXA-9, dfrA14 blaTEM-150, aph(6)-Id, aph(3'')-Ib, sul2 blaKPC-3	65	170427	54.04	Tn*4401I*

Supplementary Table 4. Genomic context of Tn4401l transposon

track	location	length	type	gene	pseudo	protein_id	product
SAMEA111504329 (16 items)							
SAMEA111504329	189 - 1,530 (+)	1341	CDS	*tnp*	False	gnl|Bakta|JBHHLJ_001	ISNCY family ISEsa1 transposase
SAMEA111504329	1,785 - 2,196 (-)	411	CDS	*ncrY*	False	gnl|Bakta|JBHHLJ_002	nickel resistance OB fold protein NcrY
SAMEA111504329	2,369 - 3,500 (-)	1131	CDS	*ncrC*	False	gnl|Bakta|JBHHLJ_003	Ni(II)/Co(II) efflux transporter permease subunit NcrC
SAMEA111504329	3,512 - 3,782 (-)	270	CDS	*ncrB*	False	gnl|Bakta|JBHHLJ_004	nickel-sensing transcriptional repressor NcrB
SAMEA111504329	3,887 - 5,186 (-)	1299	CDS	*ncrA*	False	gnl|Bakta|JBHHLJ_005	Metal Resistance
SAMEA111504329	5,419 - 6,178 (-)	759	CDS	*na*	False	gnl|Bakta|JBHHLJ_006	Tat pathway signal sequence
SAMEA111504329	6,231 - 7,152 (-)	921	CDS	*na*	False	gnl|Bakta|JBHHLJ_007	Class I SAM-dependent methyltransferase
SAMEA111504329	7,214 - 7,586 (-)	372	CDS	*na*	False	gnl|Bakta|JBHHLJ_008	DUF2946 domain-containing protein
SAMEA111504329	7,932 - 8,316 (+)	384	CDS	*chrB*	False	gnl|Bakta|JBHHLJ_009	Chromate resistance protein ChrB
SAMEA111504329	8,497 - 9,817 (+)	1320	CDS	*tnp*	False	gnl|Bakta|JBHHLJ_010	IS1182 family ISKpn6 transposase
SAMEA111504329	10,066 - 10,948 (-)	882	CDS	*blaKPC-3*	False	gnl|Bakta|JBHHLJ_011	carbapenem-hydrolyzing class A beta-lactamase KPC-3
SAMEA111504329	11,266 - 12,046 (-)	780	CDS	*istB*	False	gnl|Bakta|JBHHLJ_012	IS21-like element ISKpn7 family helper ATPase IstB
SAMEA111504329	12,042 - 13,068 (-)	1026	CDS	*tnp*	False	gnl|Bakta|JBHHLJ_013	IS21 family ISKpn7 transposase
SAMEA111504329	13,174 - 16,204 (-)	3030	CDS	*tnpA*	False	gnl|Bakta|JBHHLJ_014	Tn3-like element Tn4401 family transposase
SAMEA111504329	16,313 - 18,029 (+)	1716	CDS	*tnpR*	False	gnl|Bakta|JBHHLJ_015	Tn3-like element Tn4401 family resolvase TnpR
SAMEA111504329	18,071 - 18,980 (-)	909	CDS	*tnpA*	False	gnl|Bakta|JBHHLJ_016	TnpA
SAMEA111504348 (38 items)							
SAMEA111504348	13 - 328 (+)	315	CDS	*na*	False	gnl|Bakta|BNJLKM_001	hypothetical protein
SAMEA111504348	699 - 933 (-)	234	CDS	*na*	True	na	hypothetical protein
SAMEA111504348	987 - 1,488 (-)	501	CDS	*na*	False	gnl|Bakta|BNJLKM_003	hypothetical protein
SAMEA111504348	1,566 - 1,884 (-)	318	CDS	*na*	False	gnl|Bakta|BNJLKM_004	hypothetical protein
SAMEA111504348	1,910 - 3,911 (+)	2001	CDS	*na*	False	gnl|Bakta|BNJLKM_005	ParB/Sulfiredoxin domain-containing protein
SAMEA111504348	3,955 - 4,387 (+)	432	CDS	*psiB*	False	gnl|Bakta|BNJLKM_006	conjugation system SOS inhibitor PsiB
SAMEA111504348	4,383 - 5,112 (+)	729	CDS	*na*	False	gnl|Bakta|BNJLKM_007	Plasmid SOS inhibition protein A
SAMEA111504348	5,108 - 5,435 (+)	327	CDS	*na*	False	gnl|Bakta|BNJLKM_008	Theronine dehydrogenase
SAMEA111504348	5,806 - 5,995 (-)	189	CDS	*na*	False	gnl|Bakta|BNJLKM_009	hypothetical protein
SAMEA111504348	6,099 - 6,369 (-)	270	CDS	*na*	False	gnl|Bakta|BNJLKM_010	hypothetical protein
SAMEA111504348	6,387 - 6,999 (+)	612	CDS	*na*	False	gnl|Bakta|BNJLKM_011	IS3 family transposase
SAMEA111504348	7,036 - 7,153 (+)	117	CDS	*na*	False	gnl|Bakta|BNJLKM_012	hypothetical protein
SAMEA111504348	7,109 - 7,292 (+)	183	CDS	*na*	False	gnl|Bakta|BNJLKM_013	hypothetical protein

track	location	length	type	gene	pseudo	protein_id	product
SAMEA111504348	7,246 - 8,209 (+)	963	CDS	*na*	False	gnl|Bakta|BNJLKM_014	ATP-binding protein
SAMEA111504348	8,186 - 10,697 (+)	2511	CDS	*na*	False	gnl|Bakta|BNJLKM_015	Peptidase S8/S53 domain-containing protein
SAMEA111504348	10,697 - 10,841 (+)	144	CDS	*na*	False	gnl|Bakta|BNJLKM_016	hypothetical protein
SAMEA111504348	11,130 - 12,663 (-)	1533	CDS	*tnp*	False	gnl|Bakta|BNJLKM_017	IS3 family ISKpn38 transposase
SAMEA111504348	13,201 - 13,477 (-)	276	CDS	*na*	False	gnl|Bakta|BNJLKM_018	hypothetical protein
SAMEA111504348	13,655 - 14,144 (+)	489	CDS	*na*	False	gnl|Bakta|BNJLKM_019	hypothetical protein
SAMEA111504348	14,347 - 14,770 (-)	423	CDS	*ncrY*	False	gnl|Bakta|BNJLKM_020	nickel resistance OB fold protein NcrY
SAMEA111504348	14,931 - 16,062 (-)	1131	CDS	*ncrC*	False	gnl|Bakta|BNJLKM_021	Ni(II)/Co(II) efflux transporter permease subunit NcrC
SAMEA111504348	16,074 - 16,344 (-)	270	CDS	*ncrB*	False	gnl|Bakta|BNJLKM_022	nickel-sensing transcriptional repressor NcrB
SAMEA111504348	16,340 - 16,484 (-)	144	CDS	*na*	False	gnl|Bakta|BNJLKM_023	hypothetical protein
SAMEA111504348	16,449 - 17,706 (-)	1257	CDS	*ncrA*	False	gnl|Bakta|BNJLKM_024	Metal Resistance
SAMEA111504348	17,870 - 18,185 (+)	315	CDS	*na*	False	gnl|Bakta|BNJLKM_025	hypothetical protein
SAMEA111504348	18,341 - 18,593 (+)	252	CDS	*na*	False	gnl|Bakta|BNJLKM_026	hypothetical protein
SAMEA111504348	18,949 - 19,450 (+)	501	CDS	*na*	False	gnl|Bakta|BNJLKM_027	hypothetical protein
SAMEA111504348	19,776 - 20,109 (-)	333	CDS	*na*	False	gnl|Bakta|BNJLKM_028	DUF2946 domain-containing protein
SAMEA111504348	20,494 - 20,878 (+)	384	CDS	*chrB*	False	gnl|Bakta|BNJLKM_029	Chromate resistance protein ChrB
SAMEA111504348	21,059 - 22,379 (+)	1320	CDS	*tnp*	False	gnl|Bakta|BNJLKM_030	IS1182 family ISKpn6 transposase
SAMEA111504348	22,628 - 23,510 (-)	882	CDS	*blaKPC-3*	False	gnl|Bakta|BNJLKM_031	carbapenem-hydrolyzing class A beta-lactamase KPC-3
SAMEA111504348	23,538 - 23,832 (-)	294	CDS	*na*	False	gnl|Bakta|BNJLKM_032	hypothetical protein
SAMEA111504348	23,828 - 24,608 (-)	780	CDS	*istB*	False	gnl|Bakta|BNJLKM_033	IS21-like element ISKpn7 family helper ATPase IstB
SAMEA111504348	24,604 - 25,630 (-)	1026	CDS	*tnp*	False	gnl|Bakta|BNJLKM_034	IS21 family ISKpn7 transposase
SAMEA111504348	25,736 - 28,766 (-)	3030	CDS	*tnpA*	False	gnl|Bakta|BNJLKM_035	Tn3-like element Tn4401 family transposase
SAMEA111504348	29,037 - 30,591 (+)	1554	CDS	*xerD*	False	gnl|Bakta|BNJLKM_036	Site-specific recombinase XerD
SAMEA111504348	31,227 - 31,560 (+)	333	CDS	*na*	False	gnl|Bakta|BNJLKM_037	Replication initiator protein
SAMEA111504348	31,828 - 32,020 (-)	192	CDS	*na*	False	gnl|Bakta|BNJLKM_038	hypothetical protein
Tn4401I (9 items)							
Tn4401I	52 - 1,768 (-)	1716	CDS	*tnpR*	False	WYM66076.1	transposase
Tn4401I	1,877 - 4,907 (+)	3030	CDS	*tnpA*	False	WYM66077.1	transposase
Tn4401I	5,013 - 6,039 (+)	1026	CDS	*istA*	False	WYM66078.1	mobile element protein
Tn4401I	6,035 - 6,815 (+)	780	CDS	*istB*	False	WYM66079.1	mobile element protein
Tn4401I	7,129 - 8,011 (+)	882	CDS	*blaKPC-2*	False	WYM66080.1	KPC family carbapenem-hydrolyzing protein
Tn4401I	8,260 - 9,580 (-)	1320	CDS	*tnpA*	False	WYM66081.1	transposase

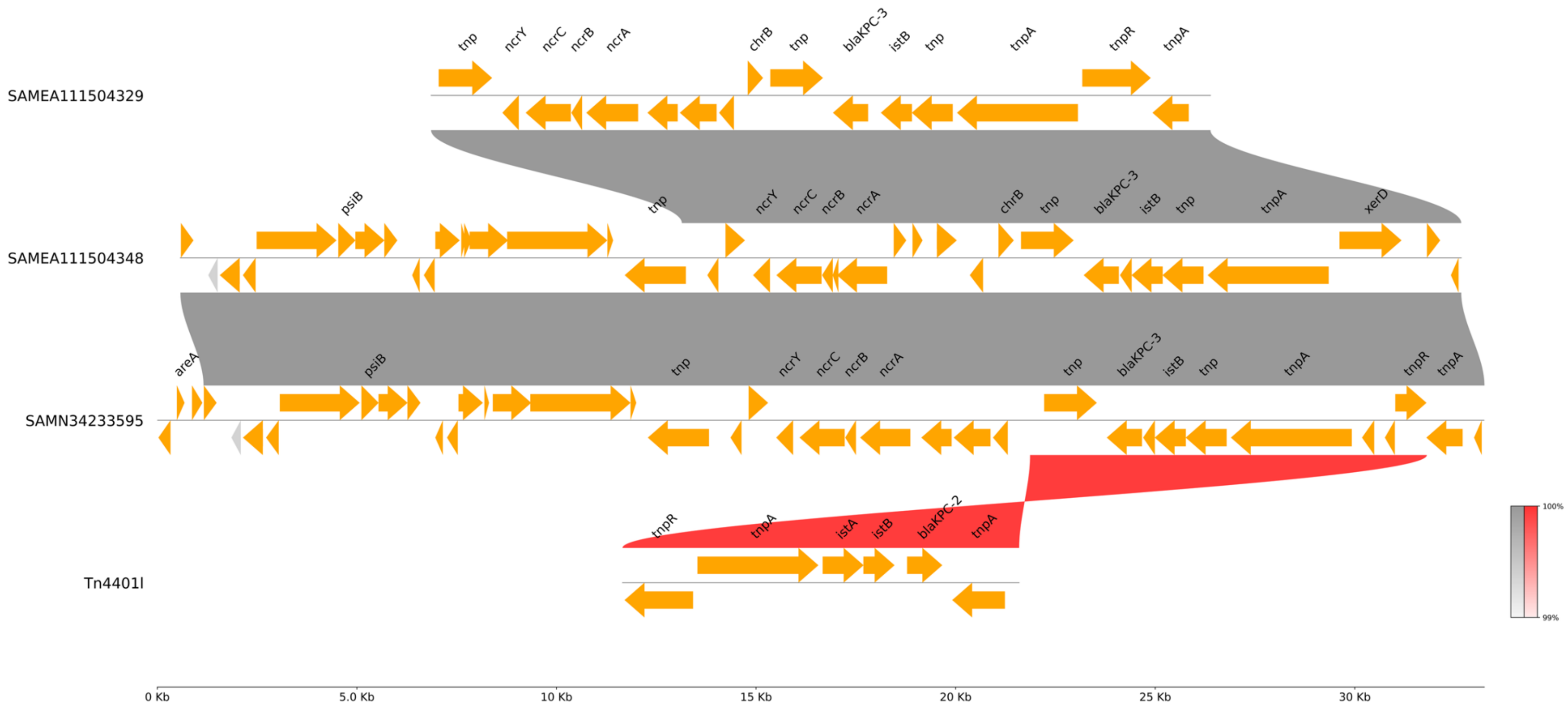

Figure S1. Comparative genomic context of Tn*4401l* harboring *bla*$_{KPC-3}$ in *Klebsiella pneumoniae* isolates.

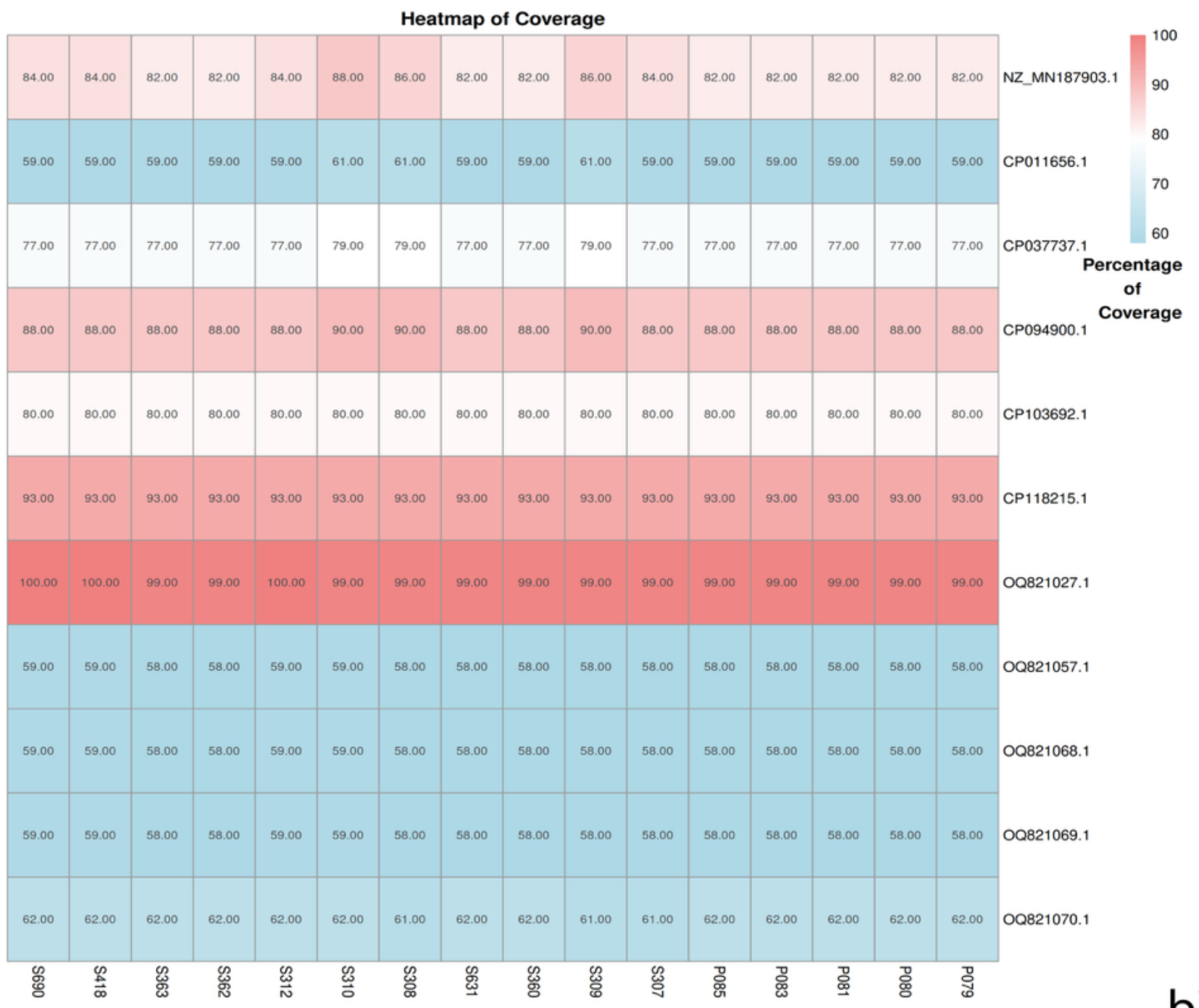

a)

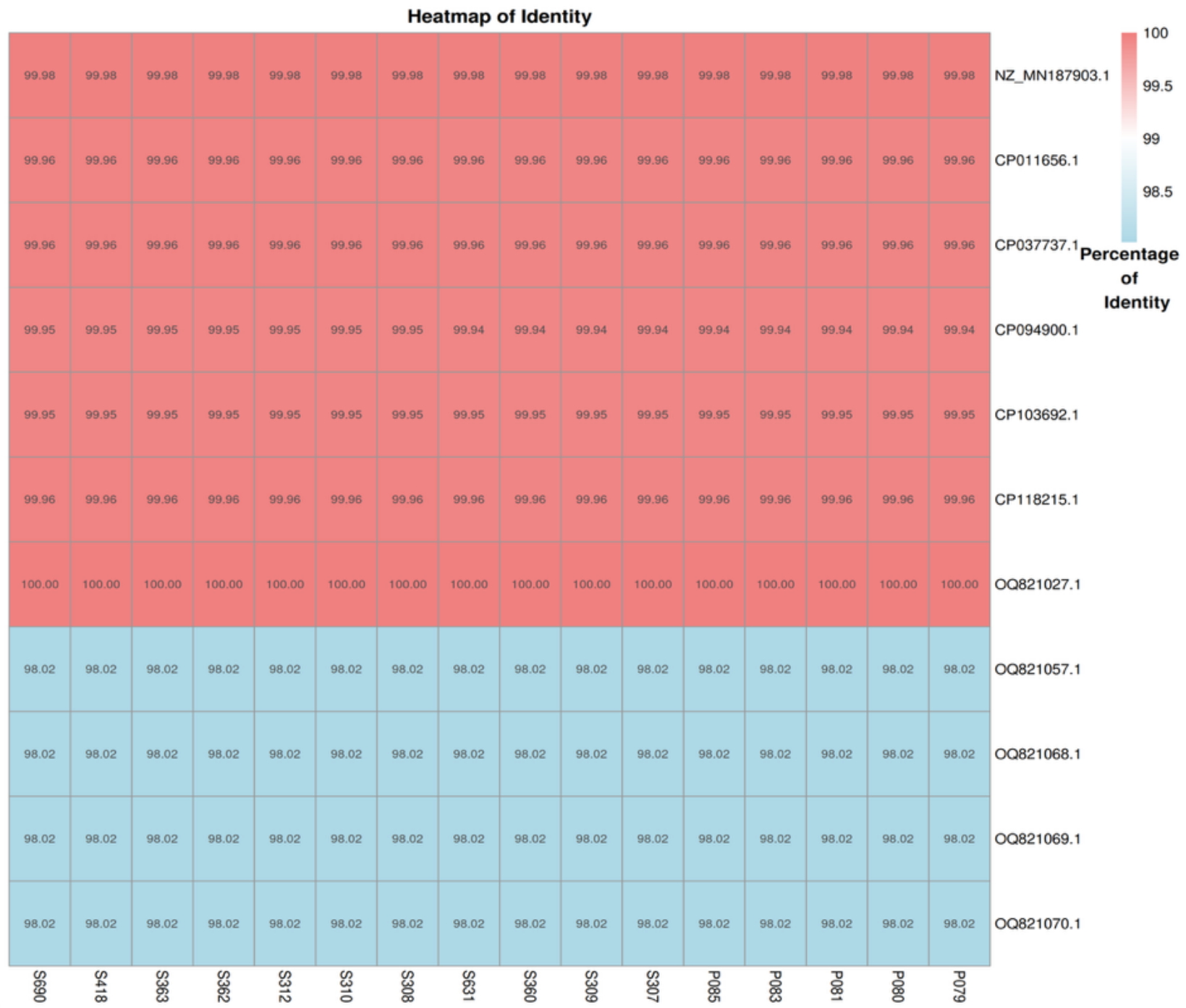

b)

Figure S2. Heatmap comparison of pECU0072020 with eleven related plasmids. (a) Coverage heatmap (query vs. subject). (b) Nucleotide identity heatmap (query vs. subject).
